# Supplementary material for: Synthesis of tertiary alkylphosphonate oligonucleotides through light-driven radical-polar crossover reactions
Source: Nat Commun. 2023 Oct 31;14:6856. doi: 10.1038/s41467-023-42639-y (PMC10618202; doi:10.1038/s41467-023-42639-y)
Supplement: Supplementary file 1 — Supplementary Information [file 41467_2023_42639_MOESM1_ESM.pdf]

**Supplementary Information for**
# Synthesis of tert-alkylphosphonate oligonucleotides through light-driven radical-polar crossover

Kenji Ota, Kazunori Nagao, Dai Hata, Haruki Sugiyama, Yasutomo Segawa, Ryosuke Tokunoh, Tomohiro Seki, Naoya Miyamoto, Yusuke Sasaki, and Hirohisa Ohmiya

## Table of Contents

■ **Supplementary Methods** ■

1. Instrumentation and Chemicals S2
2. Characterization Data for Redox-Active Esters S3–S4
3. General Procedure for Synthesis of Phosphites S4
4. Characterization Data for Phosphites and the Synthetic Intermediates S5–S14
5. General Procedure for Synthesis of *tert*-Alkylphosphonate Oligonucleotides S14
6. Characterization Data for Alkylated Products S14–S23
7. X-ray structures of **3aa** S24–S25
8. Derivatization of *tert*-Alkylphosphonates to the Phosphoramidites S26–S27
9. Characterization Data for Phosphoramidites S27–S29
10. Procedure for Automated Solid-Support Synthesis of Oligonucleotides S30–S47
11. Detection of Alkylsulfonium S48
12. Effects of Additives on Conversion of Redox-Active Esters S48
13. Comparison with Other Carbocation Methods S48–S49
14. The Effects of Chiral Leaving Groups S49
15. Examination of Catalytic Reactions S49–S50
16. Examination of the Addition of Trapping Reagent for Carbocation S50
17. Unsuccessful Substrates S51

■ **NMR Spectra** ■ S52−S190

■ **Supplementary References** ■ S191

■ Supplementary Methods ■

## 1. Instrumentation and Chemicals

NMR spectra were recorded on a Bruker AVANCE NEO 400N spectrometer, operating at 400 MHz for $^{1}$H NMR, 100.6 MHz for $^{13}$C NMR, and 162 MHz for $^{31}$P NMR. Chemical shift values for $^{1}$H and $^{13}$C are referenced to $Me_4Si$ and the residual solvent resonances, respectively. Chemical shifts were reported in $\delta$ ppm. Mass spectra were obtained with Bruker Impact HD mass spectrometer (ESI). TLC analyses were performed on commercial glass plates bearing a 0.25-mm layer of Merck Silica gel $60F_{254}$. Silica gel (Wakosil® 60, 64~210 µm) was used for column chromatography. Biotage Selekt was used for purification. Gilson GX-281 was used as a Preparative HPLC. The column was YMCActus Triant C18 (50×20 mmI.D., 5 µm) with a flow rate of 25 mL/min at room temperature. Melting points were measured on a Stanford Research Systems MPA100. Kessil A160WE 40W Tuna Blue (highest blue and intensity setting) was used as a light source. Techno Sigma UCR-150N was used as a cryocooler. TEKNOS MG9 was used as a fan.

For Prep-HPLC, Waters MS-trigger HPLC systems equipped with YMC-Actus Triart C18 (30 mm ID × 100 mm L, 5 µm) column and Waters Acquity QDa MS detector were used. The eluent was 10mM $NH_4HCO_3$ aq./MeCN. GeneDesign ns8-II oligosynthesizer was used for the automated solid-support synthesis of oligonucleotides. Oligonucleotides were analyzed for characterization by a Waters Xevo G2-XS Qtof UPLC-MS system (ESI-Qtof) with a ACQUITY UPLC OST C18 (1.7 µm, 2.1 x 50 mm) column. The eluent was 240 mM hexafluoroisopropanol/7 mM triethylamine aqueous solution containing 5% MeOH and the methanolic solution (100% MeOH). For distillation of volatile components, a Thermo Scientific SAVANT SPD2010 SpeedVac Concentrator was used. Quantification of oligonucleotides was conducted based on UV absorbance measurement on Unchaind Labs LUNATIC.

All reactions were carried out under nitrogen atmosphere. Materials were obtained from commercial suppliers or prepared according to standard procedures unless otherwise noted. **PTH1** and **PTH3** were prepared by the reported procedure.[1] **PTH2** was prepared by the methylation of benzo[*b*]phenothiazine with iodomethane.[2] **PTH4** was prepared by the reported procedure.[3] $LiBF_4$, $NaBF_4$ and $LiPF_6$ were purchased from Tokyo Chemical Industry Co., stored under nitrogen, and used as received. MeCN, DCM, DMF, THF and toluene were purchased from FUJIFILM Wako Pure Chemical Co., stored under nitrogen, and used as received. Alcohol **S1-1** was prepared by the reported procedure.[4] Alcohol **S1-2** was prepared by the reported procedure.[5] Alcohol **S1-3** was prepared by the reported procedure.[6] Phosphordiamidite **S2-3** was purchased from Tokyo Chemical Industry Co., stored under nitrogen, and used as received. Redox-active esters **2a-2k** were prepared according to the reported procedure.[7] Thymine was purchased from Tokyo Chemical Industry Co., stored under nitrogen, and used as received. 4CzIPN was prepared by the reported procedure.[8] Trifluoroacetic acid (TFA) was purchased from Nacalai Tesque Inc., stored under nitrogen, and used as received. 2-Phenyl-2-propanol (**5a**) was purchased from Tokyo Chemical Industry Co., stored under nitrogen, and used as received. Trimethylsilyl trifluoromethanesulfonate (TMSOTf) was purchased from Tokyo Chemical Industry Co., zinc iodide ($ZnI_2$) was purchased from FUJIFILM Wako Pure Chemical Co., stored under nitrogen, and used as received. Phosphites **1g** and **1h** were purchased from Tokyo Chemical Industry Co., stored under nitrogen, and used as received.

## 2. Characterization Data for Redox-Active Esters

**1,3-Dioxoisoindolin-2-yl 2-(4-Bromophenyl)-2-methylpropanoate (2b)**

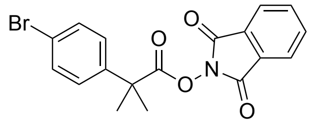

White solid. **M.p.** 125–127 °C. **$^1$H NMR** (400 MHz, $CDCl_3$) $\delta$ 7.88–7.85 (m, 2H), 7.80–7.76 (m, 2H), 7.56–7.52 (m, 2H), 7.39–7.35 (m, 2H), 1.76 (s, 6H). **$^{13}$C NMR** (100.6 MHz, $CDCl_3$) $\delta$ 172.7, 161.9, 141.6, 134.7, 131.8, 128.9, 127.6, 123.9, 121.5, 46.0, 26.7. **HRMS–ESI** (*m/z*): $[M+H]^+$ calcd for $C_{18}H_{15}BrNO_4$, 388.0179; found, 388.0187.

**1,3-Dioxoisoindolin-2-yl 1-Phenylcyclobutane-1-carboxylate (2c)**

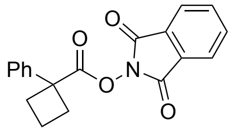

White solid. **M.p.** 93–96 °C. **$^1$H NMR** (400 MHz, $CDCl_3$) $\delta$ 7.86–7.84 (m, 2H), 7.77–7.75 (m, 2H), 7.45–7.40 (m, 4H), 7.32 (m, 1H), 3.11–3.04 (m, 2H), 2.74–2.67 (m, 2H), 2.25 (m, 1H), 2.01 (m, 1H). **$^{13}$C NMR** (100.6 MHz, $CDCl_3$) $\delta$ 172.2, 161.9, 141.1, 134.6, 129.0, 128.6, 127.4, 126.4, 123.8, 50.9, 32.6, 16.8. **HRMS–ESI** (*m/z*): $[M+NH_4]^+$ calcd for $C_{19}H_{19}N_2O_4$, 339.1339; found, 339.1337.

**1,3-Dioxoisoindolin-2-yl 1-Phenylcyclopentane-1-carboxylate (2d)**

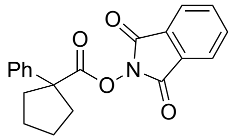

White solid. **M.p.** 84–87 °C. **$^1$H NMR** (400 MHz, $CDCl_3$) $\delta$ 7.85–7.83 (m, 2H), 7.77–7.75 (m, 2H), 7.50–7.48 (m, 2H), 7.42–7.39 (m, 2H), 7.33 (m, 1H), 2.82–2.76 (m, 2H), 2.17–2.10 (m, 2H), 1.95–1.84 (m, 4H).**$^{13}$C NMR** (100.6 MHz, $CDCl_3$) $\delta$ 172.6, 162.0, 141.1, 134.6, 129.0, 128.6, 127.4, 126.8, 123.8, 58.0, 37.1, 23.8. **HRMS–ESI** (*m/z*): $[M+NH_4]^+$ calcd for $C_{20}H_{21}N_2O_4$, 353.1496; found, 353.1501.

**1,3-Dioxoisoindolin-2-yl 4-Phenyltetrahydro-2H-pyran-4-carboxylate (2e)**

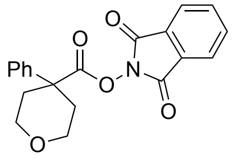

White solid. **M.p.** 122–124 °C. **$^1$H NMR** (400 MHz, $CDCl_3$) $\delta$ 7.88–7.85 (m, 2H), 7.79–7.77 (m, 2H), 7.53–7.51 (m, 2H), 7.47–7.43 (m, 2H), 7.37 (m, 1H), 4.07–4.02 (m, 2H), 3.88–3.81 (m, 2H), 2.65–2.61 (m, 2H), 2.21–2.14 (m, 2H). **$^{13}$C NMR** (100.6 MHz, $CDCl_3$) $\delta$ 171.0, 161.8, 140.7, 134.8, 129.0, 128.9, 128.0, 125.8, 123.9, 65.3, 48.9, 34.8. **HRMS–ESI** (*m/z*): $[M+H]^+$ calcd for $C_{20}H_{18}NO_5$, 352.1179; found, 352.1175.

**1,3-Dioxoisoindolin-2-yl 2,2-Diphenylpropanoate (2g)**

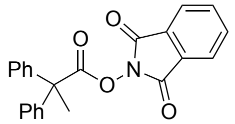


White solid. **M.p.** 114–118 °C. **$^1$H NMR** (400 MHz, $CDCl_3$) $\delta$ 7.88–7.86 (m, 2H), 7.78–7.76 (m, 2H), 7.44–7.30 (m, 10H), 2.17 (s, 3H). **$^{13}$C NMR** (100.6 MHz, $CDCl_3$) $\delta$ 171.9, 161.9, 142.3, 134.7, 129.0, 128.4, 128.0, 127.5, 123.9, 55.8, 27.1. **HRMS–ESI** (*m/z*): $[M+NH_4]^+$ calcd for $C_{23}H_{21}N_2O_4$, 389.1496; found, 389.1506.

**1,3-Dioxoisoindolin-2-yl 2-Methyl-2-phenoxypropanoate (2j)**

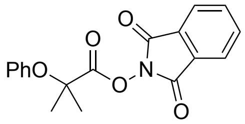


White solid. **M.p.** 74–77 °C. **$^1$H NMR** (400 MHz, $CDCl_3$) $\delta$ 7.92–7.89 (m, 2H), 7.81–7.79 (m, 2H), 7.35–7.31 (m, 2H), 7.11–7.06 (m, 3H), 1.79 (s, 6H). **$^{13}$C NMR** (100.6 MHz, $CDCl_3$) $\delta$ 170.9, 161.8, 154.6, 134.8, 129.4, 128.9, 124.0, 123.2, 120.2, 78.6, 25.7. **HRMS–ESI** (*m/z*): $[M+NH_4]^+$ calcd for $C_{18}H_{19}N_2O_5$, 343.1288; found, 343.1287.

## 3. General Procedure for Synthesis of Phosphites

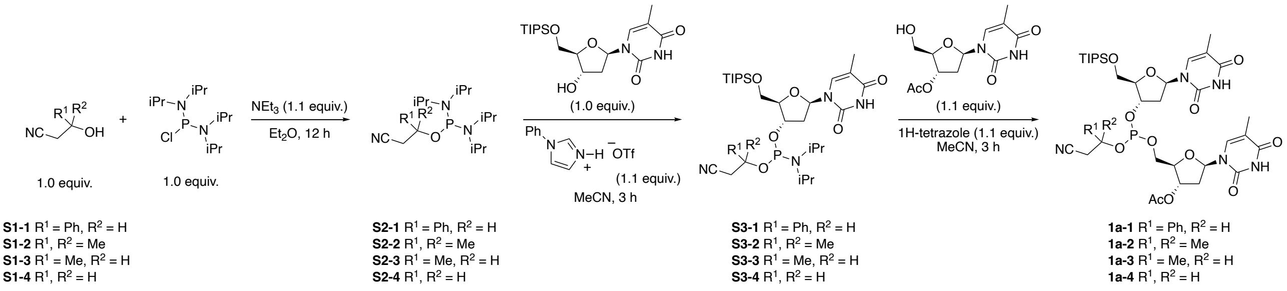


**Supplementary Fig. 1.** Synthesis of phosphites

**Synthesis of 1a-1 as a representative (Supplementary Fig. 1).** Phosphordiamidite **S2-1** was prepared according to the literature.[9] To a solution of 5'-*O*-TIPS thymidine (1.0 g, 2.5 mmol) and phosphordiamidite **S2-1** (0.94 g, 2.5 mmol) in MeCN (20 mL), 1-phenyl-1*H*-imidazol-3-ium trifluoromethanesulfonate (0.81 g, 2.75 mmol) was added. The reaction mixture was stirred for 3 h at room temperature under a nitrogen atmosphere. After the solvent was removed under reduced pressure, purification by flash column chromatography on silica gel (Biotage Selekt, 95:5–70:30, hexane/EtOAc) gave the phosphoramidite **S3-1** as a white amorphous solid (1.23 g, 1.82 mmol, 73%).

To a solution of phosphoramidite **S3-1** (1.22 g, 1.82 mmol) and 3'-*O*-Ac thymidine (0.57 g, 2.0 mmol) in MeCN (7 mL), 1*H*-tetrazole (0.14 g, 2.0 mmol) was added. The reaction mixture was stirred for 3 h at room temperature under a nitrogen atmosphere. After the filtrate was concentrated under reduced pressure, purification by flash column chromatography on silica gel (Biotage Selekt, 50:50:0–50:40:10, hexane/EtOAc/MeOH) gave the phosphite **1a-1** (1.37 g, 1.60 mmol, 88%) as a white amorphous solid.

## 4. Characterization Data for Phosphites and the Synthetic Intermediates

**3-{[Bis(diisopropylamino)phosphaneyl]oxy}-3-phenylpropanenitrile (S2-1)**

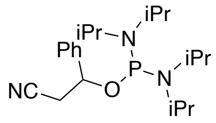

**S2-1** was purified by flash chromatography on silica gel (70:29:1, hexane/EtOAc/$NEt_3$) (Supplementary Fig. 1; 10 mmol scale; 3.24 g, 8.58 mmol, 86% isolated yield). White solid. The spectrum data of **S2-1** was consistent with the literature.[9]

**Phosphoramidite (S3-1)**

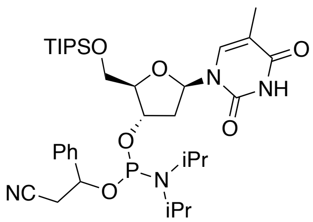

**S3-1** was purified by flash chromatography on silica gel (Biotage Selekt, 95:5–70:30, hexane/EtOAc) (Supplementary Fig 1; 2.5 mmol scale; 1.23 g, 1.82 mmol, 73% isolated yield). The ratio (2:2:1:1) of diastereomers was determined by $^{31}$P-NMR analysis. White amorphous solid. **$^1$H NMR** (400 MHz, $CDCl_3$) $\delta$ 8.21 (brs, 1H), 7.48–7.31 (m, 6H), 6.37 (m, 0.33H), 6.15 (m, 0.67H), 5.00 (m, 1H), 4.65 (m, 0.33H), 4.40 (m, 0.67H), 4.11 (m, 0.33H), 4.01–3.87 (m, 1.33H), 3.81–3.51 (m, 3.34H), 2.82–2.75 (m, 2H), 2.47 (m, 0.33H), 2.27 (m, 0.33H), 2.15 (m, 0.67H), 1.96–1.81 (m, 3.67H), 1.28–0.96 (m, 33H). **$^{13}$C NMR** (100.6 MHz, $CDCl_3$) $\delta$ 163.6, 150.3, 150.2, 150.1, 140.1, 140.1, 139.9, 135.4, 135.4, 135.3, 128.7, 128.7, 128.6, 128.6, 126.1, 126.1, 126.1, 126.0, 116.9, 116.8, 116.8, 111.0, 110.8, 110.8, 110.7, 86.8, 86.6, 86.6, 86.4, 86.4, 84.7, 84.6, 84.5, 73.7, 73.5, 73.3, 72.2, 72.0, 71.2, 71.0, 63.4, 63.3, 63.2, 43.5, 43.4, 43.3, 43.2, 40.1, 40.0, 39.9, 39.8, 39.8, 28.4, 28.4, 28.3, 28.3, 24.7, 24.6, 24.6, 24.6, 24.5, 24.5, 24.5, 24.4, 24.2, 24.1, 24.0, 18.0, 18.0, 12.3, 12.3, 11.8, 11.8 (only observed peaks). **$^{31}$P NMR** (162 MHz, $CDCl_3$) $\delta$ 149.8, 149.6, 148.3, 147.7. **HRMS–ESI** (*m/z*): $[M+Na]^+$ calcd for $C_{34}H_{55}N_4NaO_6PSi$, 697.3521; found, 697.3526.

**Phosphite (1a-1)**

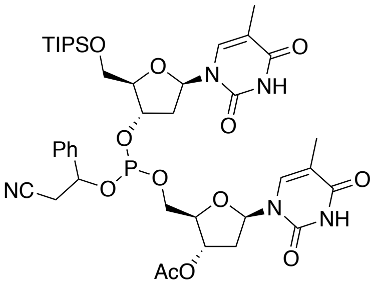

**1a-1** was purified by flash chromatography on silica gel (Biotage Selekt, 50:50:0–50:40:10, hexane/EtOAc/MeOH) (Supplementary Fig. 1; 1.82 mmol scale; 1.37 g, 1.60 mmol, 88% isolated yield) The ratio (1.3:1.2:1.1:1) of diastereomers was determined by $^{31}$P-NMR analysis. White amorphous solid. **$^1$H NMR** (400 MHz, $CDCl_3$) $\delta$ 9.09–8.90 (m, 2H), 7.52–7.28 (m, 7H), 6.34–6.21 (m, 2H), 5.40 (m, 1H), 5.19 (m, 1H), 4.83 (m, 1H), 4.17–3.53 (m, 6H), 2.94–2.75 (m, 2H), 2.43–2.04

(m, 7H), 1.99–1.88 (m, 6H), 1.14–1.05 (m, 21H). **$^{13}$C NMR** (100.6 MHz, $CDCl_3$) $\delta$ 170.7, 170.5, 170.4, 170.4, 163.7, 163.6, 150.3, 150.2, 139.3, 139.2, 138.9, 138.7, 136.2, 135.3, 135.2, 135.1, 135.0, 134.9, 129.4, 129.3, 129.3, 129.2, 129.1, 125.9, 125.8, 116.7, 116.6, 116.6, 116.5, 111.6, 111.4, 111.4, 111.3, 111.1, 111.0, 111.0, 86.3, 85.9, 85.0, 84.7, 84.6, 84.5, 84.4, 83.4, 83.3, 83.2, 74.7, 74.4, 74.2, 74.1, 73.9, 73.8, 73.5, 73.4, 71.6, 71.6, 71.5, 71.4, 71.0, 70.9, 70.7, 70.7, 63.1, 63.0, 63.0, 62.6, 62.4, 62.3, 62.1, 40.0, 39.9, 39.7, 37.3, 37.2, 37.2, 37.1, 37.0, 28.1, 27.9, 21.0, 20.9, 18.0, 12.6, 12.6, 12.3, 11.8, 11.8 (only observed peaks). **$^{31}$P NMR** (162 MHz, $CDCl_3$) $\delta$ 140.0, 139.9, 139.9, 139.2. **HRMS–ESI** (*m/z*): $[M+NH_4]^+$ calcd for $C_{40}H_{60}N_6O_{12}PSi$, 875.3771; found, 875.3772.

**3-{[Bis(diisopropylamino)phosphaneyl]oxy}-3-methylbutanenitrile (S2-2)**

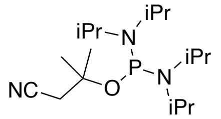


**S2-2** was purified by flash chromatography on silica gel (70:29:1, hexane/EtOAc/$NEt_3$) (Supplementary Fig. 1; 3.3 mmol scale; 789 mg, 2.4 mmol, 73% isolated yield). White solid. **M.p.** 57–59 °C. **$^1$H NMR** (400 MHz, $CDCl_3$) $\delta$ 3.62–3.53 (m, 4H), 2.59 (s, 2H), 1.51 (s, 6H), 1.20–1.16 (m, 24H). **$^{13}$C NMR** (100.6 MHz, $CDCl_3$) $\delta$ 118.1, 74.2 (d, $J_{C–P}$ = 17.4 Hz), 45.0 (d, $J_{C–P}$ = 13.4 Hz), 32.9 (d, $J_{C–P}$ = 4.9 Hz), 28.1 (d, $J_{C–P}$ = 10.2 Hz), 24.4 (d, $J_{C–P}$ = 5.7 Hz), 24.0 (d, $J_{C–P}$ = 8.4 Hz). **$^{31}$P NMR** (162 MHz, $CDCl_3$) $\delta$ 99.6. **HRMS–ESI** (*m/z*): $[M+H]^+$ calcd for $C_{17}H_{37}N_3OP$, 330.2669; found, 330.2675.

**Phosphoramidite (S3-2)**

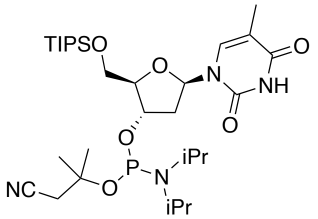


**S3-2** was purified by flash chromatography on silica gel (Biotage Selekt, 93:7–65:35, hexane/EtOAc) (Supplementary Fig. 1; 1.5 mmol scale; 621 mg, 0.99 mmol, 66% isolated yield). The ratio (1.2:1) of diastereomers was determined by $^{31}$P-NMR analysis. White amorphous solid. **$^1$H NMR** (400 MHz, $CDCl_3$) $\delta$ 8.89 (brs, 1H), 7.45 (s, 1H), 6.34 (m, 1H), 4.54 (m, 1H), 4.13 (m, 1H), 4.00–3.85 (m, 2H), 3.67–3.58 (m, 2H), 2.66–2.40 (m, 3H), 2.07 (m, 1H), 1.90 (s, 3H), 1.52–1.50 (m, 6H), 1.22–1.09 (m, 33H). **$^{13}$C NMR** (100.6 MHz, $CDCl_3$) $\delta$ 163.8, 163.8, 150.3, 150.2, 135.3, 135.3, 117.4, 117.4, 110.8, 110.8, 86.9, 86.8, 86.6, 86.5, 84.9, 84.7, 74.1, 74.0, 74.0, 73.9, 73.0, 72.9, 72.8, 72.7, 63.5, 63.5, 43.3, 43.2, 40.1, 40.0, 39.8, 39.8, 32.9, 32.9, 32.9, 32.8, 28.4, 28.4, 28.3, 28.3, 24.5, 24.4, 24.4, 24.3, 24.3, 24.2, 24.1, 18.0, 18.0, 12.3, 11.8, 11.8 (only observed peaks). **$^{31}$P NMR** (162 MHz, $CDCl_3$) $\delta$ 139.5, 139.4. **HRMS–ESI** (*m/z*): $[M+H]^+$ calcd for $C_{30}H_{56}N_4O_6PSi$, 627.3701; found, 627.3710.

**Phosphite (1a-2)**

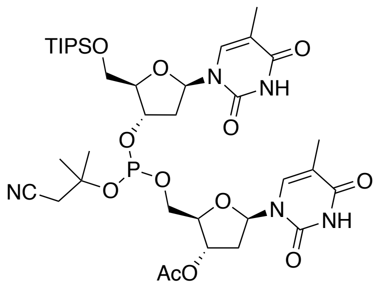


**1a-2** was purified by flash chromatography on silica gel (Biotage Selekt, 50:50:0–50:40:10, hexane/EtOAc/MeOH) (Supplementary Fig. 1; 0.6 mmol scale; 389 mg, 0.48 mmol, 80% isolated yield). The ratio (1.2:1) of diastereomers was determined by $^{31}$P-NMR analysis. White amorphous solid. **$^{1}$H NMR** (400 MHz, $CDCl_3$) $\delta$ 8.15–8.08 (m, 2H), 7.44–7.38 (m, 2H), 6.37–6.30 (m, 2H), 5.28 (m, 1H), 4.95 (m, 1H), 4.27–3.94 (m, 6H), 2.65–2.64 (m, 2H), 2.48–2.37 (m, 2H), 2.25–2.11 (m, 5H), 1.94–1.90 (m, 6H), 1.60–1.56 (m, 6H), 1.17–1.09 (m, 21H). **$^{13}$C NMR** (100.6 MHz, $CDCl_3$) $\delta$ 170.5, 170.4, 163.7, 163.5, 150.4, 150.3, 135.2, 135.1, 134.9, 116.8, 116.7, 111.5, 111.4, 111.1, 111.0, 86.4, 86.4, 86.3, 84.6, 84.6, 84.5, 83.4, 83.3, 83.3, 75.7, 75.6, 75.5, 75.4, 74.2, 73.8, 73.5, 73.4, 73.1, 73.0, 63.2, 63.2, 61.4, 61.1, 40.0, 39.8, 37.3, 37.3, 32.9, 32.9, 30.9, 28.9, 28.8, 28.8, 28.7, 28.6, 20.9, 20.9, 18.0, 18.0, 12.6, 12.6, 12.3, 12.3, 11.8 (only observed peaks). **$^{31}$P NMR** (162 MHz, $CDCl_3$) $\delta$ 135.0, 134.6. **HRMS–ESI** (*m/z*): $[M+H]^+$ calcd for $C_{36}H_{57}N_5O_{12}PSi$, 810.3505; found, 810.3497.

**3-{[Bis(diisopropylamino)phosphaneyl]oxy}butanenitrile (S2-3)**

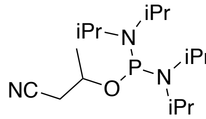


**S2-3** was purified by flash chromatography on silica gel (70:29:1, hexane/EtOAc/$NEt_3$) (Supplementary Fig. 1; 4 mmol scale; 505 mg, 1.6 mmol, 40% isolated yield). White solid. **M.p.** 41–45 °C. **$^{1}$H NMR** (400 MHz, $CDCl_3$) $\delta$ 4.00 (m, 1H), 3.58–3.48 (m, 4H), 2.64–2.54 (m, 2H), 1.38 (d, $J$ = 6.4 Hz, 3H), 1.19–1.16 (m, 24H). **$^{13}$C NMR** (100.6 MHz, $CDCl_3$) $\delta$ 117.6, 65.4, 65.2, 44.8, 44.6, 44.5, 26.6, 26.5, 24.4, 24.3, 24.2, 24.1, 24.1, 21.6, 21.6 (only observed peaks). **$^{31}$P NMR** (162 MHz, $CDCl_3$) $\delta$ 113.2. **HRMS–ESI** (*m/z*): $[M+H]^+$ calcd for $C_{16}H_{35}N_3OP$, 316.2512; found, 316.2513.

**Phosphoramidite (S3-3)**

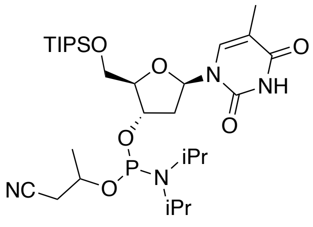


**S3-3** was purified by flash chromatography on silica gel (Biotage Selekt, 93:7–55:45, hexane/EtOAc) (Supplementary Fig. 1; 1 mmol scale; 251 mg, 0.41 mmol, 41% isolated yield). The ratio (1.3:1.2:1.1:1) of diastereomers was determined by $^{31}$P-NMR analysis. White amorphous solid. **$^{1}$H NMR** (400 MHz, $CDCl_3$) $\delta$ 8.15 (brs, 1H), 7.45 (m, 1H), 6.33 (m, 1H), 4.58 (m, 1H), 4.18–4.07 (m, 2H), 4.00–3.86 (m, 2H), 3.66–3.57 (m, 2H), 2.66–2.38 (m, 3H), 2.10 (m, 1H), 1.91 (s, 3H), 1.41–

1.36 (m, 3H), 1.22–1.09 (m, 33H). **$^{13}$C NMR** (100.6 MHz, $CDCl_3$) $\delta$ 163.6, 150.2, 150.2, 150.1, 135.4, 135.4, 135.3, 135.3, 117.1, 117.1, 117.1, 117.0, 110.9, 110.8, 110.8, 87.0, 87.0, 86.8, 86.8, 86.5, 86.5, 86.4, 84.8, 84.8, 84.6, 84.5, 73.5, 73.4, 73.3, 73.3, 73.2, 73.2, 66.2, 66.0, 65.9, 65.7, 65.6, 65.6, 65.4, 65.4, 63.5, 63.4, 63.3, 43.3, 43.3, 43.2, 43.2, 43.2, 43.1, 40.1, 40.1, 40.1, 40.0, 40.0, 39.9, 39.9, 26.9, 26.8, 26.8, 26.8, 26.7, 26.7, 24.6, 24.5, 24.5, 24.5, 24.4, 24.4, 24.3, 22.0, 21.9, 21.9, 21.9, 18.0, 18.0, 18.0, 12.3, 12.3, 11.8 (only observed peaks). **$^{31}$P NMR** (162 MHz, $CDCl_3$) $\delta$ 147.4, 147.2, 147.1, 146.8. **HRMS–ESI** (*m/z*): [M+Na]$^+$ calcd for $C_{29}H_{53}N_4NaO_6PSi$, 635.3364; found, 635.3361.

**Phosphite (1a-3)**

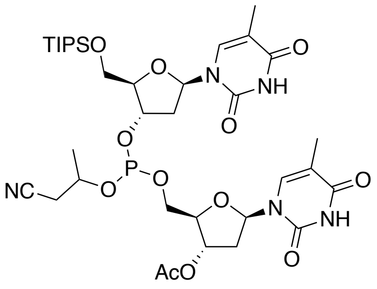


**1a-3** was purified by flash chromatography on silica gel (Biotage Selekt, 50:50:0–50:40:10, hexane/EtOAc/MeOH) (Supplementary Fig. 1; 0.4 mmol scale; 305 mg, 0.38 mmol, 96% isolated yield). The ratio (1.1:1:1:1) of diastereomers was determined by $^{31}$P-NMR analysis. White amorphous solid. **$^{1}$H NMR** (400 MHz, $CDCl_3$) $\delta$ 8.66–8.53 (m, 2H), 7.43–7.39 (m, 2H), 6.36–6.29 (m, 2H), 5.28 (m, 1H), 4.96 (m, 1H), 4.54 (m, 1H), 4.40–4.08 (m, 4H), 3.98–3.93 (m, 2H), 2.70–2.55 (m, 2H), 2.51–2.38 (m, 2H), 2.28–2.11 (m, 5H), 1.96–1.91 (m, 6H), 1.53–1.40 (m, 3H), 1.21–1.09 (m, 21H). **$^{13}$C NMR** (100.6 MHz, $CDCl_3$) $\delta$ 170.5, 170.5, 163.7, 163.6, 150.4, 150.3, 135.2, 135.2, 135.1, 134.9, 134.9, 134.9, 116.8, 116.7, 116.6, 116.6, 111.6, 111.6, 111.5, 111.3, 111.1, 86.4, 86.4, 86.4, 86.3, 86.3, 84.7, 84.6, 84.6, 84.5, 83.4, 83.3, 83.3, 83.2, 74.2, 74.1, 74.1, 74.0, 73.9, 73.8, 73.7, 73.5, 73.4, 73.3, 66.5, 66.4, 66.3, 66.2, 66.1, 66.1, 65.9, 63.4, 63.2, 62.1, 62.1, 62.0, 61.9, 61.8, 61.8, 61.7, 40.0, 40.0, 39.9, 39.9, 39.8, 39.8, 37.3, 37.2, 37.2, 27.0, 26.9, 26.9, 26.9, 26.8, 22.4, 22.4, 22.3, 22.3, 22.2, 20.9, 20.9, 20.8, 18.0, 18.0, 12.7, 12.6, 12.3, 11.8 (only observed peaks). **$^{31}$P NMR** (162 MHz, $CDCl_3$) $\delta$ 139.7, 139.2, 139.1, 139.0. **HRMS–ESI** (*m/z*): [M+Na]$^+$ calcd for $C_{35}H_{54}N_5NaO_{12}PSi$, 818.3168; found, 818.3167.

**Phosphite (1a-4)**

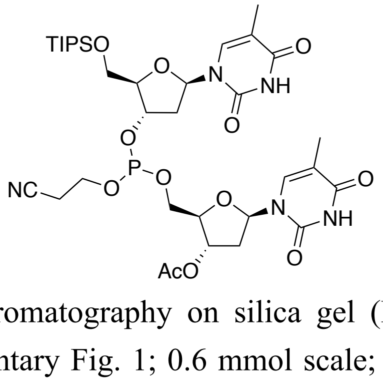


**1a-4** was purified by flash chromatography on silica gel (Biotage Selekt, 50:50:0–50:40:10, hexane/EtOAc/MeOH) (Supplementary Fig. 1; 0.6 mmol scale; 403 mg, 0.52 mmol, 86% isolated yield). The ratio (1.1:1) of diastereomers was determined by $^{31}$P-NMR analysis. White amorphous solid. **$^{1}$H NMR** (400 MHz, $CDCl_3$) $\delta$ 8.64 (brs, 2H), 7.42–7.38 (m, 2H), 6.36–6.29 (m, 2H), 5.29 (m,

1H), 4.95 (m, 1H), 4.20–4.04 (m, 6H), 3.98–3.91 (m, 2H), 2.71–2.68 (m, 2H), 2.49–2.39 (m, 2H), 2.27–2.11 (m, 5H), 1.94–1.91 (m, 6H), 1.19–1.08 (m, 21H). **$^{13}$C NMR** (100.6 MHz, $CDCl_3$) $\delta$ 170.7, 170.6, 170.5, 163.8, 163.7, 150.4, 150.3, 136.3, 135.3, 135.2, 135.0, 135.0, 117.2, 117.1, 111.6, 111.5, 111.4, 111.2, 111.2, 86.4, 86.4, 86.3, 86.0, 85.0, 84.8, 84.7, 84.6, 84.5, 83.4, 83.4, 83.3, 83.3, 74.7, 74.2, 74.1, 74.0, 73.8, 73.7, 63.1, 62.6, 62.3, 62.2, 62.2, 58.0, 57.8, 57.5, 57.4, 40.0, 39.9, 37.3, 37.2, 37.2, 21.0, 20.9, 20.4, 20.3, 20.3, 18.0, 18.0, 12.6, 12.6, 12.6, 12.3, 11.8 (only observed peaks). **$^{31}$P NMR** (162 MHz, $CDCl_3$) $\delta$ 139.7, 139.3. **HRMS–ESI** (*m/z*): $[M+NH_4]^+$ calcd for $C_{34}H_{56}N_6O_{12}PSi$, 799.3458; found, 799.3459.

### *N*-Benzoyl-5'-*O*-TIPS-5-methyl-2'-deoxycytidine

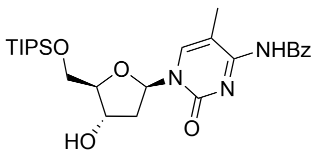


***N*-Benzoyl-5'-*O*-TIPS-5-methyl-2'-deoxycytidine** was prepared according to the reported procedure[9] and purified by flash chromatography on silica gel (Biotage Selekt, 50:50:0–50:45:5, hexane/EtOAc/MeOH) (3 mmol scale; 948 mg, 1.89 mmol, 63% isolated yield). White solid. **M.p.** 113–117 °C. **$^{1}$H NMR** (400 MHz, $CDCl_3$) $\delta$ 13.3 (brs, 1H), 8.31 (d, $J$ = 7.6 Hz, 2H), 7.65 (s, 1H), 7.52 (m, 1H), 7.46–7.42 (m, 2H), 6.39 (t, $J$ = 6.8 Hz, 1H), 4.58 (m, 1H), 4.06–3.95 (m, 3H), 2.45 (m, 1H), 2.20–2.10 (m, 5H), 1.20–1.11 (m, 21H). **$^{13}$C NMR** (100.6 MHz, $CDCl_3$) $\delta$ 179.6, 159.7, 147.9, 137.2, 136.5, 132.4, 129.9, 128.1, 111.7, 87.1, 85.4, 72.1, 63.6, 41.4, 18.0, 13.5, 11.8. **HRMS–ESI** (*m/z*): $[M+H]^+$ calcd for $C_{26}H_{40}N_3O_5Si$, 502.2732; found, 502.2734.

### *N*-Benzoyl-5'-*O*-TIPS-5-methyl-2'-deoxycytidine Phosphoramidite

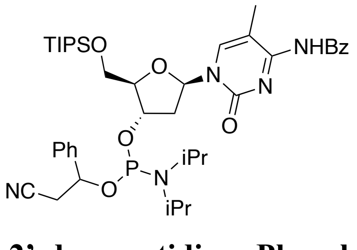


***N*-Benzoyl-5'-*O*-TIPS-5-methyl-2'-deoxycytidine Phosphoramidite** was purified by flash chromatography on silica gel (Biotage Selekt, 100:0–80:20, hexane/EtOAc) (1.5 mmol scale; 840 mg, 1.08 mmol, 72% isolated yield). The ratio (2.2:1.8:1:1) of diastereomers was determined by $^{31}$P-NMR analysis. White amorphous solid. **$^{1}$H NMR** (400 MHz, $CDCl_3$) $\delta$ 13.29 (brs, 1H), 8.33–8.30 (m, 2H), 7.69–7.31 (m, 9H), 6.39 (m, 0.34H), 6.15 (m, 0.66H), 5.02 (m, 1H), 4.66 (m, 0.34H), 4.40 (m, 0.66H), 4.20–3.51 (m, 5H), 2.84–2.72 (m, 2H), 2.54 (m, 0.34H), 2.38–2.07 (m, 4H), 1.90 (m, 0.66H), 1.28–0.96 (m, 33H). **$^{13}$C NMR** (100.6 MHz, $CDCl_3$) $\delta$ 179.6, 159.8, 159.7, 147.7, 147.7, 140.1, 140.1, 140.1, 140.0, 139.8, 137.2, 136.7, 136.6, 136.5, 132.3, 129.9, 128.7, 128.7, 128.7, 128.1, 126.1, 126.1, 126.1, 126.0, 116.9, 116.8, 116.8, 116.8, 111.6, 111.5, 87.1, 86.9, 86.9, 86.7, 86,6, 85.5, 85.4, 85.2, 73.8, 73.7, 73.6, 73.5, 73.3, 72.3, 72.2, 72.0, 71.9, 71.2, 71.2, 71.0, 71.0, 63.4, 63.3, 63.3, 63.1, 43.6, 43.6, 43.5, 43.4, 43.4, 43.2, 40.3, 40.3, 40.2, 40.1, 28.5, 28.4, 28.3, 28.3, 24.7, 24.6, 24.6, 24.5, 24.5, 24.1, 24.1, 24.0, 18.0, 18.0, 13.4, 11.9, 11.8 (only observed peaks). **$^{31}$P NMR** (162 MHz, $CDCl_3$)

$\delta$ 149.8, 149.8, 148.3, 147.9. **HRMS–ESI** (*m/z*): $[M+H]^+$ calcd for $C_{41}H_{61}N_5O_6PSi$, 778.4123; found, 778.4122.

**Phosphite (1b)**

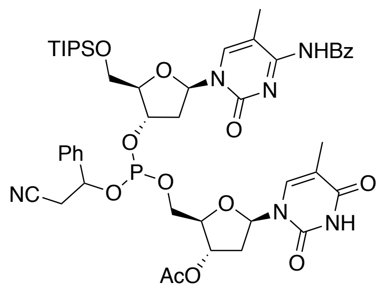

**1b** was purified by flash chromatography on silica gel (Biotage Selekt, 50:50:0–50:40:10, hexane/EtOAc/MeOH) (0.48 mmol scale; 300 mg, 0.31 mmol, 65% isolated yield). The ratio (1.1:1.1:1:1) of diastereomers was determined by $^{31}$P-NMR analysis. White amorphous solid. **$^1$H NMR** (400 MHz, $CDCl_3$) $\delta$ 13.30 (brs, 1H), 8.33–8.30 (m, 3H), 7.61–7.28 (m, 10H), 6.35–6.22 (m, 2H), 5.43 (m, 1H), 5.21 (m, 1H), 4.86 (m, 1H), 4.18–3.56 (m, 6H), 2.94–2.75 (m, 2H), 2.48–1.78 (m, 13H), 1.25–1.06 (m, 21H). **$^{13}$C NMR** (100.6 MHz, $CDCl_3$) $\delta$ 170.4, 170.4, 170.4, 163.2, 163.2, 159.6, 150.1, 150.1, 147.8, 147.8, 139.3, 139.2, 138.8, 138.7, 137.1, 136.2, 136.1, 136.1, 135.3, 135.1, 135.0, 135.0, 132.4, 129.9, 129.4, 129.4, 129.3, 129.2, 129.1, 128.1, 125.9, 125.8, 125.8, 116.7, 116.6, 116.6, 116.5, 111.9, 111.8, 111.8, 111.5, 111.4, 111.4, 111.3, 86.6, 86.6, 86.2, 85.2, 85.1, 85.0, 84.7, 84.6, 84.5, 84.5, 83.3, 83.3, 83.2, 83.2, 74.5, 74.2, 74.2, 74.1, 74.0, 73.3, 71.8, 71.6, 71.4, 71.0, 70.9, 70.7, 63.1, 63.0, 62.5, 62.5, 62.1, 40.3, 40.1, 37.3, 37.2, 37.1, 37.0, 28.1, 27.9, 20.9, 20.9, 18.0, 13.4, 12.6, 12.6, 11.8, 11.8 (only observed peaks). **$^{31}$P NMR** (162 MHz, $CDCl_3$) $\delta$ 139.9, 139.8, 139.7, 139.2. **HRMS–ESI** (*m/z*): $[M+H]^+$ calcd for $C_{47}H_{62}N_6O_{12}PSi$, 961.3927; found, 961.3930.

**Phosphite (1c)**

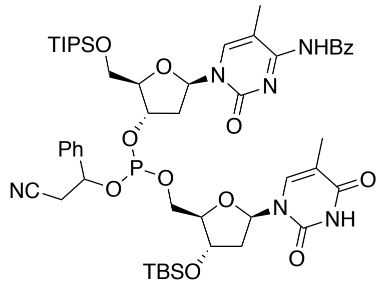

**1c** was purified by flash chromatography on silica gel (Biotage Selekt, 98:2–80:20, DCM/EtOAc) (0.63 mmol scale; 267 mg, 0.26 mmol, 41% isolated yield). The ratio (1.3:1.2:1.1:1) of diasteromers was determined by $^{31}$P-NMR analysis. White amorphous solid. **$^1$H NMR** (400 MHz, $CDCl_3$) $\delta$ 13.29 (brs, 1H), 8.32–8.30 (m, 2H), 8.07 (m, 1H), 7.59–7.51 (m, 2H), 7.46–7.20 (m, 8H), 6.31–6.16 (m, 2H), 5.39 (m, 1H), 4.85 (m, 1H), 4.38–3.54 (m, 7H), 2.92–2.74 (m, 2H), 2.46–1.74 (m, 10H), 1.18–1.07 (m, 21H), 0.90–0.88 (m, 9H), 0.09–0.08 (m, 6H). **$^{13}$C NMR** (100.6 MHz, $CDCl_3$) $\delta$ 179.5, 163.6, 159.6, 150.2, 150.1, 147.8, 139.4, 139.2, 138.9, 138.7, 137.1, 136.1, 135.8, 135.6, 132.4, 129.8, 129.3, 129.3, 129.2, 129.0, 128.1, 125.8, 125.7, 116.6, 116.6, 116.5, 116.4, 111.8, 111.8, 111.7, 111.0, 110.9, 110.9, 110.9, 86.7, 86.6, 86.6, 86.6, 86.3, 86.3, 85.7, 85.7, 85.6, 85.6, 85.5, 85.4, 85.4, 85.2, 85.1, 85.0, 85.0, 85.0, 84.9, 74.3, 74.1, 74.1, 73.9, 73.6, 73.5, 73.1, 73.0, 71.9, 71.6, 71.5, 71.4, 71.4, 71.4,

71.3, 70.7, 70.6, 70.3, 70.3, 63.0, 63.0, 62.9, 62.4, 62.3, 62.1, 62.1, 61.8, 61.7, 61.6, 61.5, 40.4, 40.3, 40.2, 40.0, 28.2, 27.9, 27.8, 25.6, 18.0, 17.8, 13.4, 13.3, 12.6, 12.5, 11.8, 11.7, −4.7, −4.7, −4.7, −4.8 (only observed peaks). **$^{31}$P NMR** (162 MHz, $CDCl_3$) $\delta$ 139.7, 139.6, 139.4, 139.1. **HRMS–ESI** (*m/z*): $[M+H]^+$ calcd for $C_{51}H_{74}N_6O_{11}PSi_2$, 1033.4686; found, 1033.4683.

**Phosphite (1d)**

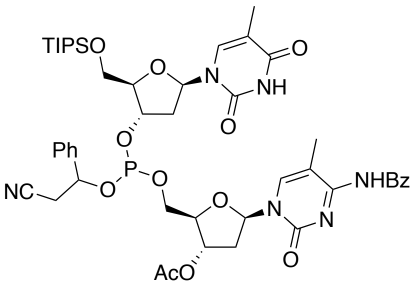


**1d** was purified by flash chromatography on silica gel (Biotage Selekt, 50:50:0–50:40:10, hexane/EtOAc/MeOH) (0.8 mmol scale; 431 mg, 0.45 mmol, 56% isolated yield). The ratio (1.1:1.1:1:1) of diastereomers was determined by $^{31}$P-NMR analysis. White amorphous solid. **$^1$H NMR** (400 MHz, $CDCl_3$) $\delta$ 13.25 (brs, 1H), 8.62 (m, 1H), 8.33–8.31 (m, 2H), 7.59–7.34 (m, 10H), 6.36–6.23 (m, 2H), 5.43 (m, 1H), 5.18 (m, 1H), 4.86 (m, 1H), 4.19–3.57 (m, 6H), 2.95–2.75 (m, 2H), 2.45–1.74 (m, 13H), 1.16–1.04 (m, 21H). **$^{13}$C NMR** (100.6 MHz, $CDCl_3$) $\delta$ 179.7, 179.7, 170.4, 170.4, 170.3, 163.4, 159.4, 159.4, 150.2, 150.1, 147.9, 139.2, 138.8, 138.8, 137.0, 136.3, 136.3, 136.2, 136.2, 136.1, 136.0, 135.0, 135.0, 134.9, 132.5, 129.9, 129.4, 129.4, 129.3, 129.2, 129.1, 129.1, 128.1, 126.0, 125.8, 125.8, 116.7, 116.6, 116.5, 112.4, 112.3, 112.2, 112.1, 111.1, 111.0, 111.0, 111.0, 86.3, 86.3, 86.0, 85.9, 85.2, 85.1, 85.0, 84.9, 84.5, 84.4, 84.4, 83.6, 83.5, 83.5, 83.4, 83.4, 74.4, 74.3, 74.2, 74.1, 74.1, 74.0, 73.8, 73.5, 73.4, 71.6, 71.6, 71.5, 71.0, 70.9, 70.9, 70.8, 63.1, 63.1, 63.0, 62.6, 62.5, 62.5, 62.4, 62.3, 62.2, 62.1, 62.0, 40.0, 39.8, 39.7, 39.7, 37.6, 37.5, 37.4, 37.3, 28.1, 27.9, 27.9, 20.9, 20.9, 18.0, 13.7, 12.3, 11.8, 11.8 (only observed peaks). **$^{31}$P NMR** (162 MHz, $CDCl_3$) $\delta$ 140.3, 140.0, 139.7, 139.2. **HRMS–ESI** (*m/z*): $[M+H]^+$ calcd for $C_{47}H_{62}N_6O_{12}PSi$, 961.3927; found, 961.3925.

***N*$^6$-Benzoyl-5'-*O*-TIPS-2'-deoxyadenosine Phosphoramidite**

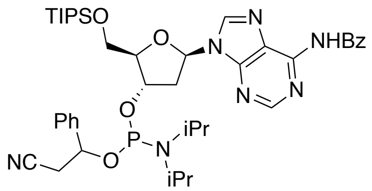


***N*$^6$-Benzoyl-5'-*O*-TIPS-2'-deoxyadenosine Phosphoramidite** was purified by flash chromatography on silica gel (Biotage Selekt, 88:12–45:55, hexane/EtOAc) (3.33 mmol scale; 1.32 g, 1.67 mmol, 50% isolated yield). The ratio (2.3:2.3:1:1) of diastereomers was determined by $^{31}$P-NMR analysis. White amorphous solid. **$^1$H NMR** (400 MHz, $CDCl_3$) $\delta$ 9.00 (m, 1H), 8.82 (m, 1H), 8.34 (s, 0.15H), 8.32 (s, 0.15H), 8.26 (s, 0.35H), 8.17 (s, 0.35H), 8.04–8.01 (m, 2H), 7.63–7.51 (m, 3H), 7.42–7.24 (m, 5H), 6.58 (m, 0.3H), 6.31 (m, 0.35H), 6.18 (m, 0.35H), 5.04 (m, 1H), 4.84 (m, 0.3H), 4.59 (m, 0.7H), 4.27 (m, 0.3H), 4.07–3.85 (m, 2H), 3.80–3.55 (m, 2.7H), 2.86–2.70 (m, 2.65H), 2.54 (m, 1H), 2.28 (m, 0.35H), 1.30–0.97 (m, 33H). **$^{13}$C NMR** (100.6 MHz, $CDCl_3$) $\delta$ 164.6, 152.6, 152.5, 152.5, 151.5, 151.5, 151.4, 151.4, 149.4, 149.4, 149.3, 141.5, 141.4, 141.3,140.2, 140.2, 140.2,

139.9, 139.9, 139.9, 133.8, 133.7, 132.7, 128.8, 128.7, 128.6, 127.8, 126.1, 126.1, 126.0, 123.3, 123.2, 123.2, 123.2, 116.9, 116.8, 116.8, 116.7, 87.4, 87.4, 87.1, 87.1, 87.0, 86.9, 84.6, 84.5, 84.5, 73.9, 73.7, 73.6, 73.5, 73.4, 73.3, 73.2, 73.1, 72.3, 72.1, 72.1, 71.9, 71.4, 71.3, 71.2, 71.1, 63.3, 63.3, 63.1, 53.4, 43.6, 43.5, 43.4, 43.3, 43.2, 43.2, 40.1, 40.0, 40.0, 39.9, 39.5, 39.5, 28.4, 28.4, 28.3, 24.7, 24.6, 24.6, 24.5, 24.5, 24.5, 24.2, 24.1, 24.1, 24.0, 17.9, 17.9, 11.8, 11.8 (only observed peaks). **$^{31}$P NMR** (162 MHz, $CDCl_3$) $\delta$ 150.3, 149.2, 148.4, 147.6. **HRMS–ESI** (*m/z*): $[M+H]^+$ calcd for $C_{41}H_{59}N_7O_5PSi$, 788.4079; found, 788.4083.

**Phosphite (1e)**

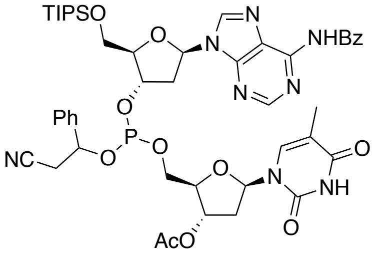


**1e** was purified by flash chromatography on silica gel (Biotage Selekt, 50:50:0–50:40:10, hexane/EtOAc/MeOH) (1.67 mmol scale; 1.06 g, 1.09 mmol, 65% isolated yield). The ratio (1.4:1.4:1.2:1) of diastereomers was determined by $^{31}$P-NMR analysis. White amorphous solid. **$^1$H NMR** (400 MHz, $CDCl_3$) $\delta$ 9.31–8.79 (m, 3H), 8.26 (m, 1H), 8.06–8.04 (m, 2H), 7.63–7.50 (m, 3H), 7.43–7.31 (m, 6H), 6.47–6.22 (m, 2H), 5.52–4.93 (m, 3H), 4.28–4.08 (m, 3H), 4.04–3.61 (m, 3H), 2.96–2.60 (m, 4H), 2.49–2.05 (m, 5H), 1.95–1.87 (m, 3H), 1.14–1.02 (m, 21H). **$^{13}$C NMR** (100.6 MHz, $CDCl_3$) $\delta$ 170.6, 170.5, 170.4, 170.4, 164.8, 163.4, 152.5, 151.6, 150.2, 149.6, 141.6, 141.5, 141.2, 141.0, 139.2, 139.2, 138.9, 138.8, 136.2, 135.2, 135.1, 135.0, 133.6, 132.7, 129.4, 129.4, 129.3, 129.1, 129.1, 128.9, 128.8, 128.0, 125.8, 125.7, 125.5, 123.4, 123.4, 116.7, 116.6, 116.6, 116.5, 111.5, 111.4, 111.4, 86.9, 86.9, 86.8, 86.8, 86.8, 85.9, 85.0, 84.8, 84.7, 84.6, 84.6, 84.6, 84.3, 83.5, 83.5, 83.4, 83.4, 83.3, 83.3, 74.7, 74.4, 74.3, 74.3, 74.2, 74.2, 74.1, 73.9, 73.7, 71.8, 71.6, 71.5, 71.3, 71.2, 70.2, 63.0, 62.9, 62.9, 62.6, 62.5, 62.4, 62.3, 62.3, 62.2, 62.2, 40.0, 40.0, 39.4, 39.1, 39.1, 37.3, 37.2, 37.2, 37.0, 28.2, 28.1, 28.0, 28.0, 28.0, 21.0, 20.9, 20.9, 18.0, 18.0, 12.6, 12.6, 12.5, 11.8,11.8 (only observed peaks). **$^{31}$P NMR** (162 MHz, $CDCl_3$) $\delta$ 140.8, 140.0, 139.8, 139.0. **HRMS–ESI** (*m/z*): $[M+H]^+$ calcd for $C_{47}H_{60}N_8O_{11}PSi$, 971.3883; found, 971.3892.

***N*²-Isobutyryl-*O*⁶-(diphenylcarbamoyl)-5'-*O*-TIPS-2'-deoxyguanosine**

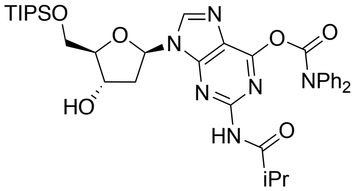


***N*²-Isobutyryl-*O*⁶-(diphenylcarbamoyl)-5'-*O*-TIPS-2'-deoxyguanosine** was prepared according to the reported procedure[10] and purified by flash chromatography on silica gel (Biotage Selekt, 90:10–67:33, DCM/EtOAc) (2.84 mmol scale; 1.39 g, 2.02 mmol, 71% isolated yield). White solid. **M.p.** 123–127 °C. **$^1$H NMR** (400 MHz, $CDCl_3$) $\delta$ 8.27 (m, 1H), 8.03 (m, 1H), 7.44–7.34 (m, 8H), 7.26–7.23 (m, 2H), 6.63 (m, 1H), 4.80 (m, 1H), 4.14 (m, 1H), 3.99–3.91 (m, 2H), 2.85 (brs, 1H),

2.67–2.59 (m, 2H), 1.64 (m, 1H), 1.26 (d, $J$ = 6.8 Hz, 6H), 1.18–1.05 (m, 21H). **$^{13}$C NMR** (100.6 MHz, $CDCl_3$) $\delta$ 175.3, 155.8, 154.7, 151.5, 150.4, 142.8, 141.7, 129.1, 127.1, 121.3, 88.2, 84.6, 72.4, 64.2, 41.7, 36.3, 19.3, 18.0, 11.9. **HRMS–ESI** (*m/z*): $[M+H]^+$ calcd for $C_{36}H_{49}N_6O_6Si$, 689.3477; found, 689.3475.

**$N^2$-Isobutyryl-$O^6$-(diphenylcarbamoyl)-5'-*O*-TIPS-2'-deoxyguanosine Phosphoramidite**

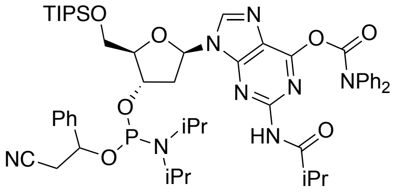

**$N^2$-Isobutyryl-$O^6$-(diphenylcarbamoyl)-5'-*O*-TIPS-2'-deoxyguanosine Phosphoramidite** was purified by flash chromatography on silica gel (Biotage Selekt, 93:7–66:34, hexane/EtOAc) (1 mmol scale; 676 mg, 0.7 mmol, 70% isolated yield). The ratio (2.1:1.9:1:1) of diastereomers was determined by $^{31}$P-NMR analysis. White amorphous solid. **$^1$H NMR** (400 MHz, $CDCl_3$) $\delta$ 8.18 (m, 1H), 7.94 (m, 1H), 7.43–7.22 (m, 15H), 6.47 (m, 0.34H), 6.16 (m, 0.66H), 5.03 (m, 1H), 4.80 (m, 0.34H), 4.54 (m, 0.66H), 4.23 (m, 0.34H), 4.02–3.57 (m, 4.66H), 3.07 (m, 1H), 2.88–2.29 (m, 4H), 1.29–0.97 (m, 39H). **$^{13}$C NMR** (100.6 MHz, $CDCl_3$) $\delta$ 175.9, 175.5, 156.0, 155.9, 155.9, 154.5, 154.4, 154.4, 151.9, 151.9, 151.8, 150.4, 150.3, 142.3, 142.2, 142.2, 141.7, 140.1, 139.9, 129.0, 128.6, 128.6, 128.5, 126.8, 126.1, 126.1, 126.0, 126.0, 121.3, 121.2, 121.1, 116.9, 116.8, 116.7, 87.4, 87.4, 87.2, 87.1, 87.0, 87.0, 87.0, 86.9, 84.5, 84.3, 73.9, 73.7, 73.5, 73.4, 73.3, 73.2, 73.1, 72.9, 72.0, 72.0, 71.8, 71.8, 71.3, 71.2, 71.1, 71.0, 63.4, 63.0, 43.6, 43.5, 43.4, 43.3, 43.3, 43.2, 43.1, 40.2, 40.0, 39.6, 39.6, 35.6, 35.3, 28.4, 28.4, 28.3, 28.2, 24.6, 24.6, 24.5, 24.4, 24.1, 24.0, 24.0, 19.2, 19.1, 17.9, 11.8, 11.8 (only observed peaks). **$^{31}$P NMR** (162 MHz, $CDCl_3$) $\delta$ 150.1, 149.2, 148.6, 147.7. **HRMS–ESI** (*m/z*): $[M+H]^+$ calcd for $C_{51}H_{70}N_8O_7PSi$, 965.4869; found, 965.4866.

**Phosphite (1f)**

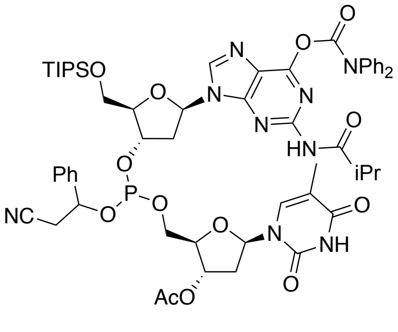

**1f** was purified by flash chromatography on silica gel (Biotage Selekt, 50:50:0–50:40:10, hexane/EtOAc/MeOH) (0.6 mmol scale; 641 mg, 0.56 mmol, 93% isolated yield). The ratio (1.2:1.1:1:1) of diastereomers was determined by $^{31}$P-NMR analysis. White amorphous solid. **$^1$H NMR** (400 MHz, $CDCl_3$) $\delta$ 8.83 (m, 1H), 8.29 (m, 1H), 8.16 (m, 1H), 7.50–7.24 (m, 16H), 6.35–6.24 (m, 2H), 5.50–4.93 (m, 3H), 4.25–3.59 (m, 6H), 2.94–2.57 (m, 4H), 2.50–2.29 (m, 2H), 2.24–2.04 (m, 4H), 1.97–1.88 (m, 3H), 1.26–1.24 (m, 6H), 1.09–1.00 (m, 21H). **$^{13}$C NMR** (100.6 MHz, $CDCl_3$) $\delta$ 175.4, 175.3, 170.6, 170.5, 170.4, 170.4, 163.5, 156.0, 154.5, 152.0, 151.9, 150.4, 150.2, 142.5, 142.3, 142.2, 141.7, 139.2, 139.0, 138.8, 136.2, 135.4, 135.3, 135.3, 129.3, 129.3, 129.2, 129.1, 129.0,

127.1, 125.9, 125.8, 121.4, 121.4, 121.3, 116.6, 116.6, 116.5, 111.4, 111.3, 111.3, 111.3, 86.9, 86.8, 86.6, 85.8, 85.0, 84.9, 84.9, 84.7, 84.6, 84.5, 84.4, 84.3, 84.2, 83.6, 83.5, 83.3, 83.3, 74.7, 74.4, 74.3, 74.3, 74.2, 74.1, 73.6, 71.7, 71.5, 71.3, 71.0, 70.8, 70.7, 63.2, 63.0, 62.5, 62.5, 62.3, 62.1, 40.0, 39.9, 39.5, 39.4, 37.3, 37.2, 37.1, 37.0, 36.0, 28.1, 27.9, 21.0, 20.9, 19.3, 17.9, 12.6, 12.5, 11.8, 11.8 (only observed peaks). **$^{31}$P NMR** (162 MHz, $CDCl_3$) $\delta$ 141.0, 140.5, 139.8, 139.1. **HRMS–ESI** (*m/z*): $[M+H]^+$ calcd for $C_{57}H_{71}N_9O_{13}PSi$, 1148.4673; found, 1148.4672.

## 5. General Procedure for Synthesis of *tert*-Alkylphosphonate Oligonucleotides

**The reaction in Table 2, entry 2 is representative.** In a glovebox, to an oven-dried vial with a stirring bar was added **PTH2** (13.2 mg, 0.05 mmol), phosphite **1a-1** (42.9 mg, 0.05 mmol), redox-active ester **2a** (46.4 mg, 0.15 mmol) and $LiBF_4$ (4.7 mg, 0.05 mmol). Then, MeCN (300 µL) and DCM (200 µL) were added to the reaction mixture. After sealing the vial with parafilm, the reaction mixture was stirred and irradiated with a 34W blue LED with UC reactor to keep the temperature at 10 °C (Supplementary Fig. 2). After 18 h, the solvents were removed under reduced pressure. The crude material was then purified by flash column chromatography on silica gel (80:20:0–50:45:5, hexane/EtOAc/MeOH) to give the alkylated product **3aa** (29.6 mg, 0.035 mmol, 70% isolated yield) as a white amorphous solid.

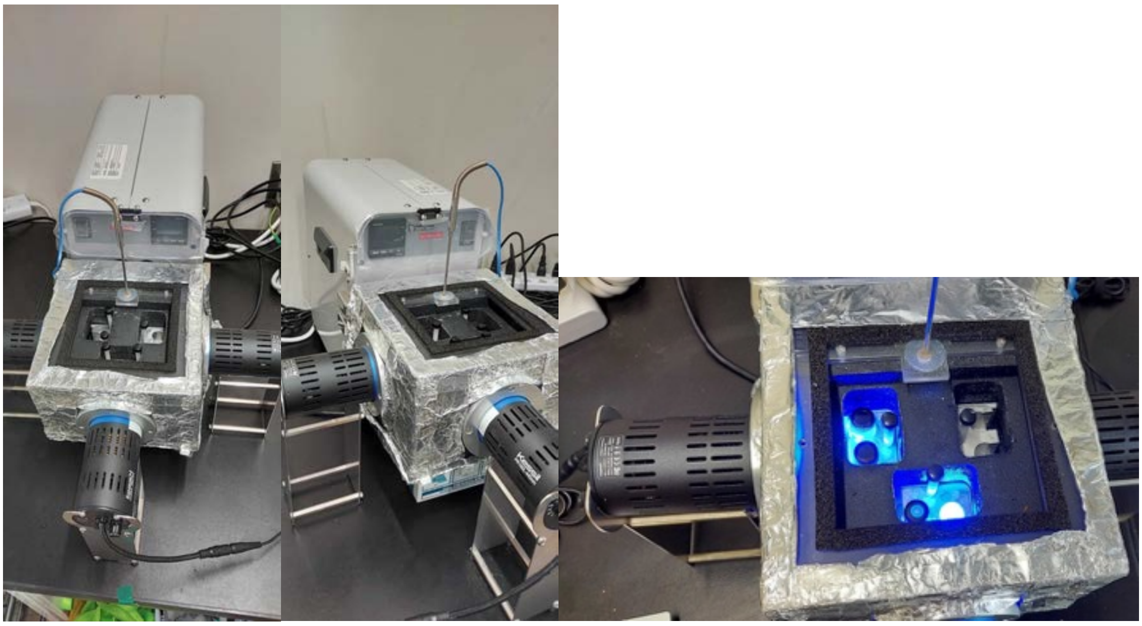

**Supplementary Fig. 2.** Light set up

## 6. Characterization Data for Alkylated Products

***tert*-Alkylphosphonate 3aa**

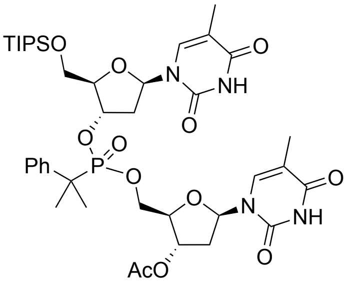

The product **3aa** was purified by flash chromatography on silica gel (80:20:0–50:45:5, hexane/EtOAc/MeOH) (Table 2, entry 2; 29.6 mg, 0.035 mmol, 70% isolated yield). The ratio (before isolation: 1.2:1→after isolation: 1:1) of diastereomers was determined by $^{31}$P-NMR analysis. White amorphous solid. **$^{1}$H NMR** (400 MHz, $CDCl_3$) $\delta$ 9.07–8.98 (m, 2H), 7.55–7.23 (m, 7H), 6.29–6.24 (m, 2H), 5.14 (m, 1H), 4.86 (t, $J$ = 5.8 Hz, 0.5H), 4.60 (t, $J$ = 6.0 Hz, 0.5H), 4.26–4.11 (m, 3.5H), 3.87 (m, 1H), 3.71 (m, 1H), 3.39 (m, 0.5H), 2.45–2.34 (m, 1.5H), 2.10–1.85 (m, 11.5H), 1.70–1.65 (m, 6H), 1.10–1.03 (m, 21H). **$^{13}$C NMR** (100.6 MHz, $CDCl_3$) $\delta$ 170.5, 170.3, 163.6, 163.5, 150.3, 140.2, 140.2, 140.0, 139.9, 135.1, 134.8, 134.6, 128.4, 128.4, 128.4, 127.7, 127.7, 127.6, 127.5, 127.4, 127.4, 127.4, 111.6, 111.5, 111.2, 86.8, 86.8, 85.6, 85.6, 84.7, 84.7, 84.3, 83.0, 82.9, 82.8, 82.7, 78.5, 78.5, 78.4, 78.3, 73.9, 73.9, 65.6, 65.5, 64.8, 64.8, 63.2, 40.1, 40.1, 39.9, 39.0, 38.9, 38.8, 38.7, 37.2, 37.2, 24.5, 24.5, 24.1, 24.1, 23.7, 23.7, 23.5, 23.5, 20.8, 18.0, 18.0, 18.0, 12.6, 12.3, 12.3, 12.2, 11.7, 11.7 (only observed peaks). **$^{31}$P NMR** (162 MHz, $CDCl_3$) $\delta$ 34.6, 33.5. **HRMS–ESI** (*m/z*): $[M+H]^+$ calcd for $C_{40}H_{60}N_4O_{12}PSi$, 847.3709; found, 847.3710.

**The separation of the diastereomers of 3aa.**

The crude residue of **3aa** (from 20 × 0.05 mmol scale) was purified with column chromatography (silica gel, hexane/EtOAc/MeOH = 50:50:0 to 42.5:42.5:15), and then the fractions containing the diastereomers mixture were collected and further purified by preparative HPLC (Reverse phase, C18 column, 10mM $NH_4HCO_3$ aq./MeCN = 30/70 to 15/85) to obtain 200 mg (0.24 mmol) of **(*R*$_P$)-3aa** and 250 mg (0.30 mmol) of **(*S*$_P$)-3aa**.

50 mg of the single diastereomer [identified as **(*R*$_P$)-3aa**] was weighed out and transferred to a clean glass tube. About 1 mL of acetonitrile was added to the tube and the mixture was heated until the solid dissolved completely. To the solution was added water (2~3 mL) quietly and the solution was allowed to cool slowly to room temperature. The solution was then placed in the fridge to further promote crystal formation. The crystals were collected using a filtration apparatus and washed with a small amount of water. The crystals were then dried in vacuo.

***tert*-Alkylphosphonate 3ba**

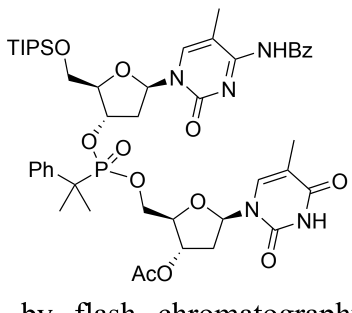


The product **3ba** was purified by flash chromatography on silica gel (80:20:0–50:45:5, hexane/EtOAc/MeOH) (Figure 2; 24.7 mg, 0.026 mmol, 52% isolated yield). The ratio (before isolation: 1.2:1) of diastereomers was determined by $^{31}$P-NMR analysis. The diastereomers **3ba-d1** and **3ba-d2** were separated and data were measured respectively.

**3ba-d1**

White amorphous solid. **$^1$H NMR** (400 MHz, $CDCl_3$) $\delta$ 13.29 (brs, 1H), 8.32–8.30 (m, 2H), 8.15 (brs, 1H), 7.56–7.51 (m, 4H), 7.46–7.42 (m, 2H), 7.36–7.32 (m, 2H), 7.27–7.24 (m, 2H), 6.30–6.24 (m, 2H), 5.14 (m, 1H), 4.61 (t, $J$ = 6.0 Hz, 1H), 4.25 (m, 1H), 4.15–4.13 (m, 2H), 4.07 (m, 1H), 3.92–3.86 (m, 2H), 2.37 (m, 1H), 2.17 (m, 1H), 2.10–2.07 (m, 6H), 1.99 (d, $J$ = 1.2 Hz, 3H), 1.93–1.84 (m, 2H), 1.70–1.65 (m, 6H), 1.14–1.03 (m, 21H). **$^{13}$C NMR** (100.6 MHz, $CDCl_3$) $\delta$ 170.3, 163.0, 159.6, 149.9, 147.8, 140.0, 139.9, 137.1, 136.0, 134.6, 132.5, 129.9, 128.5, 128.5, 128.1, 127.6, 127.6, 127.5, 112.0, 111.6, 87.2, 85.0, 84.8, 82.8, 82.8, 78.6, 78.5, 73.9, 64.9, 64.8, 63.3, 40.1, 39.4, 39.3, 38.8, 37.2, 24.2, 24.1, 23.7, 23.7, 20.9, 18.0, 18.0, 13.4, 12.6, 11.8 (only observed peaks). **$^{31}$P NMR** (162 MHz, $CDCl_3$) $\delta$ 34.7. **HRMS–ESI** (*m/z*): $[M+H]^+$ calcd for $C_{47}H_{65}N_5O_{12}PSi$, 950.4131; found, 950.4128.

**3ba-d2**

White amorphous solid. **$^1$H NMR** (400 MHz, $CDCl_3$) $\delta$ 13.29 (brs, 1H), 8.32–8.29 (m, 2H), 8.13 (m, 1H), 7.57–7.42 (m, 7H), 7.37–7.34 (m, 2H), 7.28 (m, 1H), 6.29–6.22 (m, 2H), 5.14 (m, 1H), 4.89 (t, $J$ = 6.4 Hz, 1H), 4.25–4.08 (m, 3H), 3.75–3.72 (m, 2H), 3.41 (m, 1H), 2.53 (dd, $J$ = 13.6, 4.8 Hz, 1H), 2.38 (m, 1H), 2.10–2.03 (m, 7H), 1.99 (d, $J$ = 1.2 Hz, 3H), 1.85 (m, 1H), 1.69–1.64 (m, 6H), 1.13–1.04 (m, 21H). **$^{13}$C NMR** (100.6 MHz, $CDCl_3$) $\delta$ 170.5, 163.2, 159.6, 149.9, 147.9, 140.2, 137.1, 136.0, 135.1, 132.5, 129.9, 128.4, 128.4, 128.1, 127.7, 127.7, 127.4, 127.4, 112.0, 111.5, 86.0, 85.9, 84.9, 84.8, 83.0, 83.0, 78.5, 78.4, 73.8, 65.5, 65.4, 63.3, 40.3, 40.3, 40.1, 38.8, 37.2, 24.5, 24.5, 23.6, 23.5, 20.9, 18.0, 13.4, 12.3, 11.7 (only observed peaks). **$^{31}$P NMR** (162 MHz, $CDCl_3$) $\delta$ 33.6. **HRMS–ESI** (*m/z*): $[M+H]^+$ calcd for $C_{47}H_{65}N_5O_{12}PSi$, 950.4131; found, 950.4131.

***tert*-Alkylphosphonate 3ca**

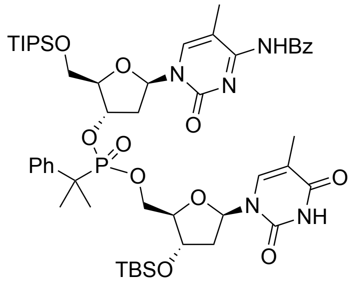


The product **3ca** was purified by flash chromatography on silica gel (80:20:0–50:45:5, hexane/EtOAc/MeOH) (Figure 2; 25.6 mg, 0.025 mmol, 50% isolated yield). The ratio (before isolation: 1.1:1→after isolation: 1.3:1) of diastereomers was determined by $^{31}$P-NMR analysis. White amorphous solid. **$^1$H NMR** (400 MHz, $CDCl_3$) $\delta$ 13.30 (brs, 1H), 8.37–8.30 (m, 3H), 7.58–7.22 (m, 10H), 6.30–6.17 (m, 2H), 4.88 (t, $J$ = 6.0 Hz, 0.43H), 4.63 (t, $J$ = 6.0 Hz, 0.57H), 4.24 (m, 1H), 4.12–3.88 (m, 4.57H), 3.76 (m, 1H), 3.49 (m, 0.43H), 2.50 (m, 0.43H), 2.22–1.77 (m, 9.57H), 1.70–1.64 (m, 6H), 1.11–1.05 (m, 21H), 0.89 (s, 9H), 0.08–0.07 (m, 6H). **$^{13}$C NMR** (100.6 MHz, $CDCl_3$) $\delta$ 179.6, 163.4, 163.3, 159.6, 149.9, 147.8, 140.4, 140.1, 140.0, 137.1, 136.0, 135.7, 135.1, 132.5, 129.9, 128.4, 128.4, 128.4, 128.1, 127.8, 127.7, 127.6, 127.5, 127.4, 127.3, 112.0, 112.0, 111.2, 111.1, 87.2, 86.1, 85.4, 85.3, 85.3, 85.1, 85.0, 78.3, 78.3, 78.0, 77.9, 71.8, 71.6, 65.5, 65.4, 64.4, 64.3, 63.3, 63.2, 40.6, 40.5, 40.3, 40.2, 40.1, 39.4, 39.3, 38.8, 38.7, 25.7, 24.2, 24.1, 24.0, 23.8, 23.6, 18.0, 18.0, 18.0, 17.9, 13.4, 13.4, 12.6, 12.3, 11.8, 11.7, −4.7, −4.8, −4.9 (only observed peaks). **$^{31}$P NMR** (162

MHz, $CDCl_3$) $\delta$ 34.7, 33.2. **HRMS–ESI** (*m/z*): $[M+H]^+$ calcd for $C_{51}H_{77}N_5O_{11}PSi_2$, 1022.4890; found, 1022.4885.

***tert*-Alkylphosphonate 3da**

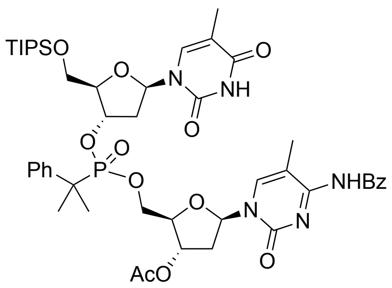

The product **3da** was purified by flash chromatography on silica gel (80:20:0–50:45:5, hexane/EtOAc/MeOH) (Figure 2; 21.9 mg, 0.023 mmol, 46% isolated yield). The ratio (before isolation: 1.1:1→after isolation: 1:1) of diastereomers was determined by $^{31}$P-NMR analysis. White amorphous solid. **$^1$H NMR** (400 MHz, $CDCl_3$) $\delta$ 13.29 (brs, 1H), 8.42–8.32 (m, 3H), 7.72–7.25 (m, 10H), 6.30–6.24 (m, 2H), 5.14 (m, 1H), 4.89 (t, *J* = 6.0 Hz, 0.5H), 4.66 (t, *J* = 6.0 Hz, 0.5H), 4.28–4.08 (m, 3.5H), 3.88 (m, 1H), 3.72 (m, 1H), 3.42 (m, 0.5H), 2.47–2.42 (m, 1.5H), 2.18–2.17 (m, 3H), 2.12–2.04 (m, 4H), 1.91–1.81 (m, 4.5H), 1.71–1.65 (m, 6H), 1.10–1.04 (m, 21H). **$^{13}$C NMR** (100.6 MHz, $CDCl_3$) $\delta$ 179.7, 170.5, 170.3, 163.3, 159.6, 159.4, 150.1, 147.8, 140.2, 140.2, 140.0, 139.9, 137.1, 137.0, 136.3, 135.6, 134.8, 134.8, 132.6, 132.5, 129.9, 128.5, 128.4, 128.4, 128.1, 128.1, 127.7, 127.7, 127.6, 127.5, 127.4, 112.5, 112.4, 111.2, 86.9, 85.7, 85.6, 85.3, 85.2, 84.4, 84.3, 83.3, 83.2, 83.1, 83.0, 78.5, 78.4, 78.3, 73.9, 65.4, 65.4, 64.8, 64.7, 63.3, 40.1, 40.1, 39.9, 39.1, 39.0, 38.8, 38.8, 37.5, 37.5, 24.5, 24.5, 24.1, 24.1, 23.7, 23.6, 20.8, 18.0, 18.0, 18.0, 13.7, 13.4, 12.3, 12.2, 11.8, 11.7 (only observed peaks). **$^{31}$P NMR** (162 MHz, $CDCl_3$) $\delta$ 34.7, 33.7. **HRMS–ESI** (*m/z*): $[M+H]^+$ calcd for $C_{47}H_{65}N_5O_{12}PSi$, 950.4131; found, 950.4133.

***tert*-Alkylphosphonate 3ea**

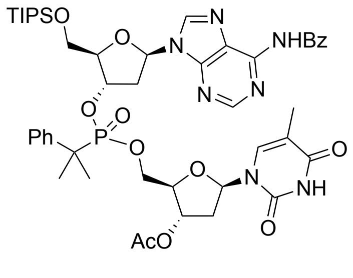

The product **3ea** was purified by flash chromatography on silica gel (50:50:0–50:42:8, hexane/EtOAc/MeOH) (Figure 2; 15.8 mg, 0.017 mmol, 33% isolated yield). The ratio (before isolation: 1.1:1) of diasteromers was determined by $^{31}$P-NMR analysis. The diastereomers **3ea-d1** and **3ea-d2** were separated and data were measured respectively.

**3ea-d1**

White amorphous solid. **$^1$H NMR** (400 MHz, $CDCl_3$) $\delta$ 9.08 (brs, 1H), 8.81 (s, 1H), 8.69 (brs, 1H), 8.28 (s, 1H), 8.03 (d, *J* = 7.2 Hz, 2H), 7.62–7.28 (m, 9H), 6.51 (m, 1H), 6.18 (m, 1H), 5.12–5.08 (m, 2H), 4.24–4.12 (m, 3H), 3.94 (s, 1H), 3.79 (m, 1H), 3.50 (m, 1H), 2.81–2.71 (m, 2H), 2.32 (m, 1H),

2.07 (s, 3H), 1.99 (s, 3H), 1.80–1.68 (m, 7H), 1.12–1.03 (m, 21H). **$^{13}$C NMR** (100.6 MHz, $CDCl_3$) $\delta$ 170.5, 163.3, 150.0, 140.4, 140.3, 135.3, 133.6, 132.8, 132.8, 128.8, 128.5, 128.5, 128.0, 127.7, 127.6, 127.4, 127.4, 111.5, 86.4, 86.4, 85.2, 84.3, 83.2, 83.2, 78.6, 78.6, 74.0, 63.3, 40.2, 37.0, 29.7, 29.6, 24.6, 24.5, 23.7, 23.6, 20.9, 18.0, 12.4, 11.8 (only observed peaks). **$^{31}$P NMR** (162 MHz, $CDCl_3$) $\delta$ 33.8. **HRMS–ESI** (*m/z*): [M+H]$^+$ calcd for $C_{47}H_{63}N_7O_{11}PSi$, 960.4087; found, 960.4096.

**3ea-d2**

White amorphous solid. **$^1$H NMR** (400 MHz, $CDCl_3$) $\delta$ 9.00 (brs, 1H), 8.82 (s, 1H), 8.25 (brs, 1H), 8.21 (s, 1H), 8.03 (d, $J$ = 7.2 Hz, 2H), 7.64–7.51 (m, 5H), 7.37–7.33 (m, 2H), 7.28–7.22 (m, 2H), 6.37 (m, 1H), 6.27 (m, 1H), 5.16 (m, 1H), 4.87 (t, $J$ = 6.0 Hz, 1H), 4.33–4.24 (m, 2H), 4.17–4.11 (m, 2H), 3.93–3.84 (m, 2H), 2.55 (m, 1H), 2.39 (m, 1H), 2.21 (m, 1H), 2.11 (s, 3H), 2.00–1.91 (m, 4H), 1.73–1.69 (m, 6H), 1.14–1.02 (m, 21H). **$^{13}$C NMR** (100.6 MHz, $CDCl_3$) $\delta$ 170.3, 163.1, 149.9, 141.1, 134.7, 132.8, 128.9, 128.5, 128.5, 127.9, 127.6, 127.6, 127.5, 111.6, 87.2, 84.9, 84.2, 82.9, 82.8, 78.6, 78.5, 73.9, 64.9, 63.2, 40.1, 39.2, 39.2, 38.8, 37.2, 24.3, 24.3, 23.7, 23.6, 20.9, 18.0, 12.7, 11.8 (only observed peaks). **$^{31}$P NMR** (162 MHz, $CDCl_3$) $\delta$ 34.3. **HRMS–ESI** (*m/z*): [M+H]$^+$ calcd for $C_{47}H_{63}N_7O_{11}PSi$, 960.4087; found, 960.4088.

***tert*-Alkylphosphonate 3fa**

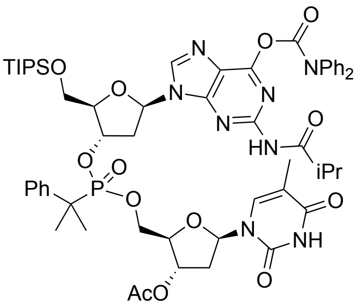


The product **3fa** (from total 0.45 mmol scale synthesis) was purified with column chromatography (silica gel, hexane/EtOAc/MeOH = 50:50:0 to 50:40:10), and then the fractions containing the product were collected and further purified by preparative HPLC (30:70–0:100, 10mM $NH_4HCO_3$ in $H_2O$/MeCN) (Figure 2; 25.6 mg, 0.023 mmol, 5% isolated yield). The ratio (before isolation: 1.1:1) of diasteromers was determined by $^{31}$P-NMR analysis. The diastereomers **3fa-d1** and **3fa-d2** were separated and data were measured respectively.

**3fa-d1**

Off-white amorphous solid. **$^1$H NMR** (400 MHz, $CDCl_3$) $\delta$ 8.36–8.26 (m, 2H), 8.12 (s, 1H), 7.59–7.21 (m, 16H), 6.29–6.25 (m, 2H), 5.18 (m, 1H), 4.86 (t, $J$ = 6.0 Hz, 1H), 4.32–4.12 (m, 4H), 3.90–3.82 (m, 2H), 2.38 (m, 1H), 2.20–2.08 (m, 6H), 2.01–1.95 (m, 4H), 1.72–1.68 (m, 6H), 1.27 (d, $J$ = 6.8 Hz, 6H), 1.05–1.00 (m, 21H). **$^{13}$C NMR** (100.6 MHz, $CDCl_3$) $\delta$ 170.3, 163.3, 156.0, 154.6, 152.1, 150.4, 150.0, 142.0, 140.1, 140.0, 134.8, 129.1, 128.5, 128.4, 127.6, 127.6, 127.5, 126.8, 121.2, 111.5, 87.3, 84.7, 83.9, 82.7, 82.7, 78.5, 78.4, 73.8, 64.9, 64.8, 63.2, 40.1, 39.3, 39.3, 38.8, 37.2, 36.0, 24.3, 24.3, 23.6, 23.6, 20.9, 19.3, 18.0, 18.0, 12.7, 11.8 (only observed peaks). **$^{31}$P NMR** (162 MHz, $CDCl_3$) $\delta$ 34.6. **HRMS–ESI** (*m/z*): [M+H]$^+$ calcd for $C_{57}H_{74}N_8O_{13}PSi$, 1137.4877; found, 1137.4885.

**3fa-d2**

Off-white amorphous solid. **$^1$H NMR** (400 MHz, $CDCl_3$) $\delta$ 8.41–8.24 (m, 2H), 8.17 (s, 1H), 7.58–7.24 (m, 16H), 6.42 (m, 1H), 6.16 (dd $J$ = 8.8, 5.2 Hz, 1H), 5.13 (m, 1H), 5.03 (m, 1H), 4.26–4.12 (m, 3H), 3.89 (m, 1H), 3.74 (m, 1H), 3.49 (m, 1H), 2.77–2.67 (m, 2H), 2.31 (m, 1H), 2.12–2.06 (m, 4H), 2.00–1.99 (m, 3H), 1.81 (m, 1H), 1.71–1.67 (m, 6H), 1.25 (d, $J$ = 6.8 Hz, 6H), 1.05–1.02 (m, 21H). **$^{13}$C NMR** (100.6 MHz, $CDCl_3$) $\delta$ 170.5, 163.4, 156.0, 154.6, 152.1, 150.4, 149.9, 142.1, 141.8, 140.4, 140.4, 135.5, 129.2, 128.4, 128.4, 127.7, 127.6, 127.4, 121.3, 111.4, 86.3, 86.3, 85.2, 84.2, 83.1, 83.0, 78.6, 78.6, 73.9, 65.6, 65.5, 63.3, 40.2, 38.9, 37.0, 24.5, 24.5, 23.7, 23.6, 20.8, 19.3, 19.3, 18.0, 12.3, 11.8 (only observed peaks). **$^{31}$P NMR** (162 MHz, $CDCl_3$) $\delta$ 33.5. **HRMS–ESI** (*m/z*): $[M+H]^+$ calcd for $C_{57}H_{74}N_8O_{13}PSi$, 1137.4877; found, 1137.4891.

***tert*-Alkylphosphonate 3ab**

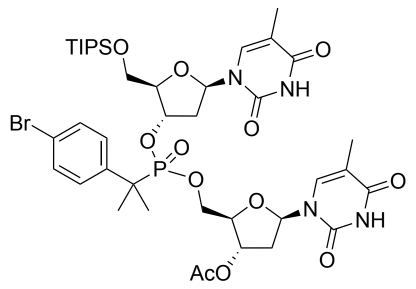


The product **3ab** was purified by flash chromatography on silica gel (80:20:0–50:45:5, hexane/EtOAc/MeOH) (Figure 2; 26.9 mg, 0.029 mmol, 58% isolated yield). The ratio (before isolation: 1.2:1→after isolation: 1:1) of diasteromers was determined by $^{31}$P-NMR analysis. White amorphous solid. **$^1$H NMR** (400 MHz, $CDCl_3$) $\delta$ 8.93–8.86 (m, 2H), 7.49–7.24 (m, 6H), 6.29–6.24 (m, 2H), 5.15 (m, 1H), 4.86 (t, $J$ = 6.0 Hz, 0.5H), 4.57 (t, $J$ = 5.8 Hz, 0.5H), 4.28–4.09 (m, 3.5H), 3.91–3.72 (m, 2H), 3.38 (m, 0.5H), 2.47–2.38 (m, 1.5H), 2.11–1.88 (m, 11.5H), 1.66–1.61 (m, 6H), 1.15–1.00 (m, 21H). **$^{13}$C NMR** (100.6 MHz, $CDCl_3$) $\delta$ 170.7, 170.5, 170.3, 163.6, 163.5, 150.5, 150.3, 150.2, 139.4, 139.4, 139.1, 139.1, 136.1, 135.1, 134.8, 134.7, 134.6, 131.5, 131.5, 131.4, 129.6, 129.5, 129.5, 129.4, 121.8, 121.7, 121.7, 121.6, 111.7, 111.6, 111.4, 111.3, 111.3, 86.9, 85.9, 85.6, 85.6, 85.0, 84.9, 84.3, 84.3, 82.9, 82.9, 82.7, 82.7, 78.8, 78.7, 78.5, 78.4, 74.7, 73.7, 65.8, 65.7, 64.9, 64.9, 63.2, 62.6, 40.0, 39.9, 39.0, 39.0, 38.7, 38.6, 37.2, 24.5, 24.4, 24.1, 24.1, 23.5, 23.5, 23.5, 23.4, 21.0, 20.8, 18.0, 12.6, 12.6, 12.4, 12.3, 11.8, 11.7 (only observed peaks). **$^{31}$P NMR** (162 MHz, $CDCl_3$) $\delta$ 33.7, 32.7. **HRMS–ESI** (*m/z*): $[M+H]^+$ calcd for $C_{40}H_{59}BrN_4O_{12}PSi$, 925.2814; found, 925.2816.

***tert*-Alkylphosphonate 3ac**

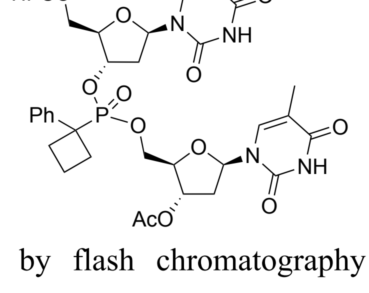


The product **3ac** was purified by flash chromatography on silica gel (80:20:0–50:45:5, hexane/EtOAc/MeOH) (Figure 2; 26.6 mg, 0.031 mmol, 62% isolated yield). The ratio (crude 1.1:1

→isolated 1.1:1) of diasteromers was determined by $^{31}$P-NMR analysis. White amorphous solid. **$^1$H NMR** (400 MHz, CDCl$_3$) $\delta$ 8.94–8.82 (m, 2H), 7.49–7.16 (m, 7H), 6.32–6.23 (m, 2H), 5.18 (m, 1H), 4.95 (t, $J$ = 5.8 Hz, 0.48H), 4.69 (t, $J$ = 6.0 Hz, 0.52H), 4.33–4.08 (m, 3.52H), 3.91–3.79 (m, 2H), 3.62 (m, 0.48H), 2.95–2.88 (m, 2H), 2.68–2.56 (m, 2H), 2.50–2.23 (m, 3H), 2.11–1.89 (m, 12H), 1.15–1.03 (m, 21H). **$^{13}$C NMR** (100.6 MHz, CDCl$_3$) $\delta$ 170.4, 170.4, 163.5, 163.4, 150.2, 141.6, 141.6, 141.5, 141.5, 135.1, 134.8, 134.8, 134.5, 128.3, 128.2, 128.2, 127.5, 127.5, 127.5, 127.5, 127.0, 126.9, 111.8, 111.6, 111.2, 111.2, 86.7, 85.8, 85.7, 84.7, 84.6, 84.4, 84.3, 83.0, 82.9, 82.7, 82.7, 78.3, 78.2, 78.2, 73.7, 73.7, 65.8, 65.8, 65.1, 65.1, 63.4, 63.2, 45.7, 45.6, 44.3, 44.2, 39.8, 39.3, 39.3, 37.2, 37.1, 30.9, 30.8, 30.5, 30.5, 30.5, 30.4, 29.7, 20.9, 18.0, 18.0, 18.0, 17.2, 17.2, 12.6, 12.3, 12.3, 11.7 (only observed peaks). **$^{31}$P NMR** (162 MHz, CDCl$_3$) $\delta$ 31.9, 30.8. **HRMS–ESI** (*m/z*): [M+H]$^+$ calcd for $C_{41}H_{60}N_4O_{12}PSi$, 859.3709; found, 859.3714.

### *tert*-Alkylphosphonate 3ad

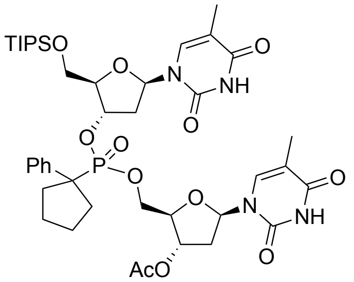


The product **3ad** was purified by flash chromatography on silica gel (80:20:0–50:45:5, hexane/EtOAc/MeOH) (Figure 2; 14.8 mg, 0.017 mmol, 34% isolated yield). The ratio (before isolation: 1.2:1→after isolation: 1.2:1) of diasteromers was determined by $^{31}$P-NMR analysis. White amorphous solid. **$^1$H NMR** (400 MHz, CDCl$_3$) $\delta$ 8.63–8.51 (m, 2H), 7.49–7.15 (m, 7H), 6.28–6.20 (m, 2H), 5.12 (m, 1H), 4.90 (t, $J$ = 5.6 Hz, 0.45H), 4.65 (t, $J$ = 5.8 Hz, 0.55H), 4.24–4.04 (m, 3.55H), 3.88–3.76 (m, 2H), 3.56 (m, 0.45H), 2.46–1.83 (m, 21H), 1.14–1.03 (m, 21H). **$^{13}$C NMR** (100.6 MHz, CDCl$_3$) $\delta$ 170.4, 170.3, 163.4, 150.1, 139.3, 135.2, 134.8, 134.6, 128.5, 128.5, 128.3, 127.3, 111.7, 111.5, 111.2, 86.7, 85.7, 84.8, 84.6, 84.3, 82.7, 78.2, 78.1, 73.8, 73.7, 65.6, 65.0, 64.9, 63.4, 63.3, 51.9, 51.9, 50.5, 39.8, 39.2, 39.2, 37.1, 35.1, 35.0, 34.2, 34.1, 24.2, 24.2, 20.9, 18.0, 18.0, 18.0, 12.6, 12.3, 12.3, 11.8 (only observed peaks). **$^{31}$P NMR** (162 MHz, CDCl$_3$) $\delta$ 34.2, 33.1. **HRMS–ESI** (*m/z*): [M+H]$^+$ calcd for $C_{42}H_{62}N_4O_{12}PSi$, 873.3866; found, 873.3863.

### *tert*-Alkylphosphonate 3ae

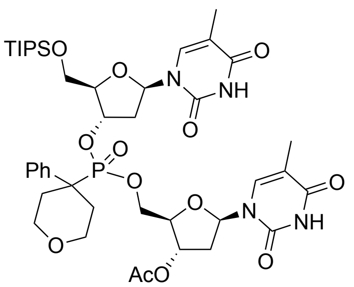


The product **3ae** was purified by flash chromatography on silica gel (80:20:0–50:45:5, hexane/EtOAc/MeOH) (Figure 2; 22.7 mg, 0.026 mmol, 51% isolated yield). The ratio (crude 1.2:1 →isolated 1.2:1) of diasteromers was determined by $^{31}$P-NMR analysis. White amorphous solid. **$^1$H**

**NMR** (400 MHz, $CDCl_3$) $\delta$ 8.69–8.51 (m, 2H), 7.48–7.22 (m, 7H), 6.28–6.20 (m, 2H), 5.16 (m, 1H), 4.84 (t, $J$ = 6.0 Hz, 0.43H), 4.45 (t, $J$ = 5.6 Hz, 0.57H), 4.26–4.05 (m, 3.57H), 3.86–3.74 (m, 4H), 3.54–3.50 (m, 1.43H), 3.40 (m, 1H), 2.46–2.34 (m, 5H), 2.11–1.80 (m, 12H), 1.13–1.02 (m, 21H). **$^{13}$C NMR** (100.6 MHz, $CDCl_3$) $\delta$ 170.4, 170.4, 163.4, 163.3, 163.2, 150.2, 150.1, 135.3, 134.8, 134.4, 129.3, 129.3, 129.2, 129.2, 129.0, 128.9, 127.7, 127.7, 127.6, 111.7, 111.6, 111.2, 86.9, 85.6, 85.5, 85.0, 84.3, 84.3, 82.9, 82.8, 82.7, 82.6, 78.8, 78.7, 78.4, 73.7, 73.6, 65.1, 65.0, 63.3, 63.2, 62.9, 62.8, 62.7, 43.1, 41.7, 39.9, 39.0, 37.1, 37.1, 30.5, 29.7, 29.6, 20.8, 18.0, 18.0, 18.0, 12.6, 12.3, 11.7 (only observed peaks). **$^{31}$P NMR** (162 MHz, $CDCl_3$) $\delta$ 30.2, 29.1. **HRMS–ESI** (*m/z*): $[M+H]^+$ calcd for $C_{42}H_{62}N_4O_{13}PSi$, 889.3815; found, 889.3814.

### *tert*-Alkylphosphonate 3af

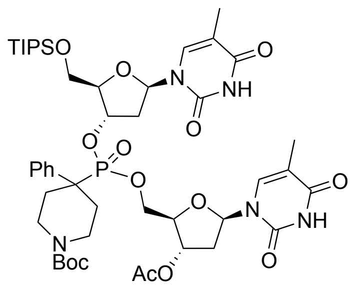


The product **3af** was purified by flash chromatography on silica gel (80:20:0–50:45:5, hexane/EtOAc/MeOH) (Figure 2; 14.8 mg, 0.015 mmol, 30% isolated yield). The ratio (crude 1.3:1 →isolated 2.2:1) of diasteromers was determined by $^{31}$P-NMR analysis. White amorphous solid. **$^{1}$H NMR** (400 MHz, $CDCl_3$) $\delta$ 8.39–8.20 (m, 2H), 7.47–7.17 (m, 7H), 6.27–6.16 (m, 2H), 5.13 (m, 1H), 4.84 (m, 0.31H), 4.44 (m, 0.69H), 4.22–3.53 (m, 10H), 2.84–2.74 (m, 2H), 2.54–2.32 (m, 4H), 2.11–2.07 (m, 5H), 1.96–1.88 (m, 6H), 1.43–1.42 (m, 9H), 1.10–1.02 (m, 21H). **$^{13}$C NMR** (100.6 MHz, $CDCl_3$) $\delta$ 170.4, 170.3, 163.2, 163.2, 163.1, 154.8, 150.0, 149.9, 135.5, 134.8, 129.3, 129.3, 129.2, 129.2, 129.0, 129.0, 128.9, 128.9, 127.8, 127.7, 111.7, 111.5, 111.2, 86.9, 85.3, 84.3, 82.7, 79.9, 77.7, 73.5, 70.5, 63.2, 37.0, 37.0, 29.7, 28.4, 28.3, 20.8, 18.0, 18.0, 18.0, 12.6, 12.3, 12.3, 11.8, 11.7 (only observed peaks). **$^{31}$P NMR** (162 MHz, $CDCl_3$) $\delta$ 30.6, 29.5. **HRMS–ESI** (*m/z*): $[M+NH_4]^+$ calcd for $C_{47}H_{74}N_6O_{14}PSi$, 1005.4764; found, 1005.4765.

### *tert*-Alkylphosphonate 3ag

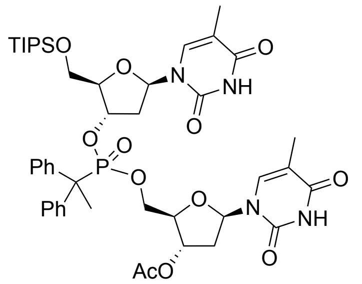


The product **3ag** was purified by flash chromatography on silica gel (80:20:0–50:45:5, hexane/EtOAc/MeOH) (Figure 2; 20.0 mg, 0.022 mmol, 44% isolated yield). The ratio (crude 1.2:1 →isolated 1.2:1) of diasteromers was determined by $^{31}$P-NMR analysis. White amorphous solid. **$^{1}$H NMR** (400 MHz, $CDCl_3$) $\delta$ 8.48–8.38 (m, 2H), 7.52–7.07 (m, 12H), 6.27–6.20 (m, 2H), 5.19 (m, 0.55H), 5.06 (m, 0.45H), 4.93 (t, $J$ = 6.0 Hz, 0.45H), 4.42 (m, 1H), 4.27 (m, 0.55H), 4.17–4.11 (m,

2.55H), 3.87 (m, 1H), 3.63 (m, 0.55H), 3.50 (s, 0.45H), 3.29 (m, 0.45H), 2.50 (m, 0.45H), 2.30 (m, 1H), 2.10–2.02 (m, 6H), 1.97–1.69 (m, 8.55H), 1.12–1.03 (m, 21H). **$^{13}$C NMR** (100.6 MHz, $CDCl_3$) $\delta$ 170.6, 170.3, 163.5, 163.5, 163.4, 163.3, 150.2, 150.2, 142.7, 142.7, 142.5, 142.5, 140.8, 140.8, 140.3, 140.3, 134.9, 134.8, 134.5, 129.2, 129.1, 129.0, 129.0, 128.5, 128.4, 128.3, 128.2, 127.5, 127.2, 111.7, 111.6, 111.2, 111.2, 87.1, 85.3, 85.3, 84.6, 84.5, 84.3, 83.0, 82.9, 82.7, 82.6, 79.2, 79.2, 79.2, 79.1, 74.0, 74.0, 65.8, 65.7, 65.1, 65.0, 63.3, 63.2, 51.0, 50.9, 49.6, 49.6, 40.0, 38.8, 38.7, 37.0, 29.7, 25.1, 25.0, 20.8, 18.0, 18.0, 18.0, 18.0, 12.5, 12.3, 12.2, 12.1, 11.8, 11.7 (only observed peaks). **$^{31}$P NMR** (162 MHz, $CDCl_3$) $\delta$ 31.1, 30.0. **HRMS–ESI** (*m/z*): $[M+NH_4]^+$ calcd for $C_{45}H_{65}N_5O_{12}PSi$, 926.4131; found, 926.4140.

***tert*-Alkylphosphonate 3ah**

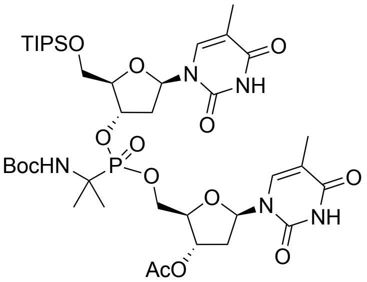


The product **3ah** was purified by flash chromatography on silica gel (80:20:0–50:45:5, hexane/EtOAc/MeOH) (Figure 2; 34.1 mg, 0.039 mmol, 77% isolated yield). The ratio (crude 1:1→ isolated 1:1) of diasteromers was determined by $^{31}$P-NMR analysis. Off-white amorphous solid. **$^{1}$H NMR** (400 MHz, $CDCl_3$) $\delta$ 8.46–8.32 (m, 2H), 7.62 (s, 0.5H), 7.45–7.36 (m, 1.5H), 6.38–6.27 (m, 2H), 5.27–5.17 (m, 2H), 4.89 (d, $J$ = 4.4 Hz, 0.5H), 4.76 (d, $J$ = 4.0 Hz, 0.5H), 4.41–4.19 (m, 4H), 4.03–3.94 (m, 2H), 2.58 (m, 1H), 2.44 (m, 1H), 2.30–2.10 (m, 5H), 1.95–1.91 (m, 6H), 1.63–1.55 (m, 6H), 1.42–1.41 (m, 9H), 1.17–1.08 (m, 21H). **$^{13}$C NMR** (100.6 MHz, $CDCl_3$) $\delta$ 170.5, 170.3, 163.8, 163.7, 163.6, 154.2, 154.2, 154.1, 150.5, 150.4, 150.4, 150.4, 135.4, 134.9, 134.9, 111.8, 111.7, 111.2, 111.2, 86.7, 85.8, 85.7, 85.0, 84.7, 84.4, 82.9, 82.8, 82.7, 82.7, 79.9, 79.8, 78.6, 78.5, 78.3, 78.3, 73.9, 73.8, 66.2, 66.1, 65.3, 65.3, 63.4, 63.3, 52.8, 52.8, 51.3, 51.2, 39.8, 39.3, 39.2, 37.0, 36.8, 28.3, 23.8, 23.8, 23.5, 23.3, 20.8, 18.0, 12.4, 12.3, 12.2, 11.8 (only observed peaks). **$^{31}$P NMR** (162 MHz, $CDCl_3$) $\delta$ 30.0, 29.2. **HRMS–ESI** (*m/z*): $[M+NH_4]^+$ calcd for $C_{39}H_{68}N_6O_{14}PSi$, 903.4295; found, 903.4296.

***tert*-Alkylphosphonate 3ai**

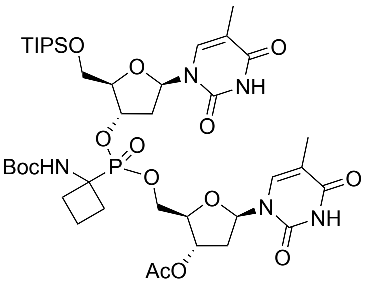


The product **3ai** was purified by flash chromatography on silica gel (80:20:0–50:45:5, hexane/EtOAc/MeOH) (Figure 2; 41.8 mg, 0.047 mmol, 93% isolated yield). The ratio (crude 1.2:1 →isolated 1.1:1) of diastereomers was determined by $^{31}$P-NMR analysis. Off-white amorphous solid. **$^{1}$H NMR** (400 MHz, $CDCl_3$) $\delta$ 8.47–8.37 (m, 2H), 7.62 (s, 0.48H), 7.46–7.35 (m, 1.52H), 6.38–6.25

(m, 2H), 5.26–5.21 (m, 2H), 5.10 (s, 0.52H), 4.92 (s, 0.48H), 4.39–4.18 (m, 4H), 4.05–3.93 (m, 2H), 2.63–2.10 (m, 13H), 1.96–1.91 (m, 6H), 1.42 (s, 9H), 1.19–1.08 (m, 21H). **$^{13}$C NMR** (100.6 MHz, $CDCl_3$) $\delta$ 170.4, 170.3, 163.7, 163.7, 163.6, 153.9, 153.8, 150.5, 150.4, 150.3, 135.4, 135.2, 134.9, 111.7, 111.6, 111.2, 111.2, 86.6, 85.9, 85.9, 85.2, 84.8, 84.4, 84.4, 82.9, 82.9, 82.8, 82.8, 80.0, 80.0, 78.5, 78.4, 78.3, 73.9, 66.0, 65.9, 65.4, 65.3, 63.5, 63.4, 54.2, 54.1, 52.7, 52.5, 39.8, 39.4, 39.4, 37.0, 36.7, 29.9, 29.7, 29.4, 28.3, 28.3, 20.9, 18.0, 16.4, 12.5, 12.3, 11.8 (only observed peaks). **$^{31}$P NMR** (162 MHz, $CDCl_3$) $\delta$ 27.5, 26.7. **HRMS–ESI** (*m/z*): $[M+NH_4]^+$ calcd for $C_{40}H_{68}N_6O_{14}PSi$, 915.4295; found, 915.4298.

***tert*-Alkylphosphonate 3aj**

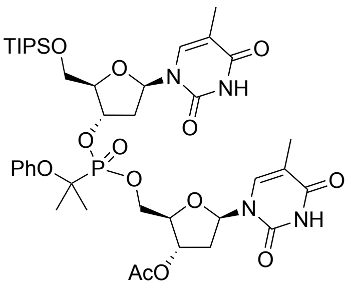


The product **3aj** was purified by flash chromatography on silica gel (80:20:0–50:45:5, hexane/EtOAc/MeOH) (Figure 2; 23.7 mg, 0.028 mmol, 55% isolated yield). The ratio (crude 1.2:1 →isolated 1.2:1) of diastereomers was determined by $^{31}$P-NMR analysis. White amorphous solid. **$^{1}$H NMR** (400 MHz, $CDCl_3$) $\delta$ 8.58–8.33 (m, 2H), 7.58 (m, 0.45H), 7.44 (m, 1H), 7.38 (m, 0.55H), 7.31–7.27 (m, 2H), 7.17–7.05 (m, 3H), 6.41–6.29 (m, 2H), 5.36–5.29 (m, 2H), 4.52–4.31 (m, 3H), 4.22 (s, 1H), 4.05–3.81 (m, 2H), 2.59 (m, 1H), 2.44 (m, 1H), 2.25–2.10 (m, 5H), 1.94–1.90 (m, 6H), 1.58–1.44 (m, 6H), 1.14–1.01 (m, 21H). **$^{13}$C NMR** (100.6 MHz, $CDCl_3$) $\delta$ 170.5, 170.4, 163.4, 163.3, 153.1, 153.0, 153.0, 152.9, 150.2, 150.2, 135.2, 134.8, 134.8, 129.2, 124.9, 124.2, 111.8, 111.7, 111.3, 111.2, 86.7, 86.7, 86.0, 86.0, 84.8, 84.7, 84.5, 84.3, 83.0, 82.9, 82.8, 82.7, 79.7, 79.6, 78.8, 78.8, 78.7, 78.0, 77.8, 74.0, 73.9, 66.2, 66.1, 65.7, 65.6, 63.5, 63.4, 40.0, 39.5, 39.4, 37.2, 37.0, 29.7, 29.6, 23.8, 23.8, 23.3, 23.3, 22.6, 22.6, 22.2, 22.2, 20.9, 20.8, 18.0, 17.9, 12.4, 12.3, 11.8, 11.7 (only observed peaks). **$^{31}$P NMR** (162 MHz, $CDCl_3$) $\delta$ 25.9, 25.3. **HRMS–ESI** (*m/z*): $[M+H]^+$ calcd for $C_{40}H_{60}N_4O_{13}PSi$, 863.3658; found, 863.3659.

## 7. X-ray Structure of ($R_P$)-3aa

Details of the crystal data and a summary of the intensity data collection parameters for ($R_P$)-**3aa** are listed in Table S1. A suitable crystal obtained by recrystallization from acetonitrile and water was mounted with mineral oil on a MiTeGen MicroMounts and transferred to the goniometer of the kappa goniometer of a RIGAKU XtaLAB Synergy-S system with 1.2 kW MicroMax-007HF microfocus rotating anode (Graphite-monochromated Mo Kα radiation ($\lambda$ = 0.71073 Å)) and HyPix-6000HE hybrid photon-counting detector. Cell parameters were determined and refined, and raw frame data were integrated using CrysAlis$^{Pro}$ (Agilent Technologies, 2010) and a multi-scan absorption correction using SCALE3 ABSPACK was applied. The structures were solved by direct methods with SHELXT[11] and refined by full-matrix least-squares techniques against $F^2$ (SHELXL-2018/3)[12] by using Olex2 software package.[13] The intensities were corrected for Lorentz and polarization effects. All non-hydrogen atoms were refined with anisotropic displacement parameters. The hydrogen atoms were refined isotropically on calculated positions using a riding model with their $U_{iso}$ values constrained to 1.5 times the $U_{eq}$ of their pivot atoms for terminal sp$^3$ carbon atoms and 1.2 times for all other carbon atoms. Crystallographic data for the structures reported in this paper have been deposited with the Cambridge Crystallographic Data Centre. CCDC 2263561 contains the supplementary crystallographic data for this paper. These data can be obtained free of charge from The Cambridge Crystallographic Data Centre via www.ccdc.cam.ac.uk/data_request/cif.

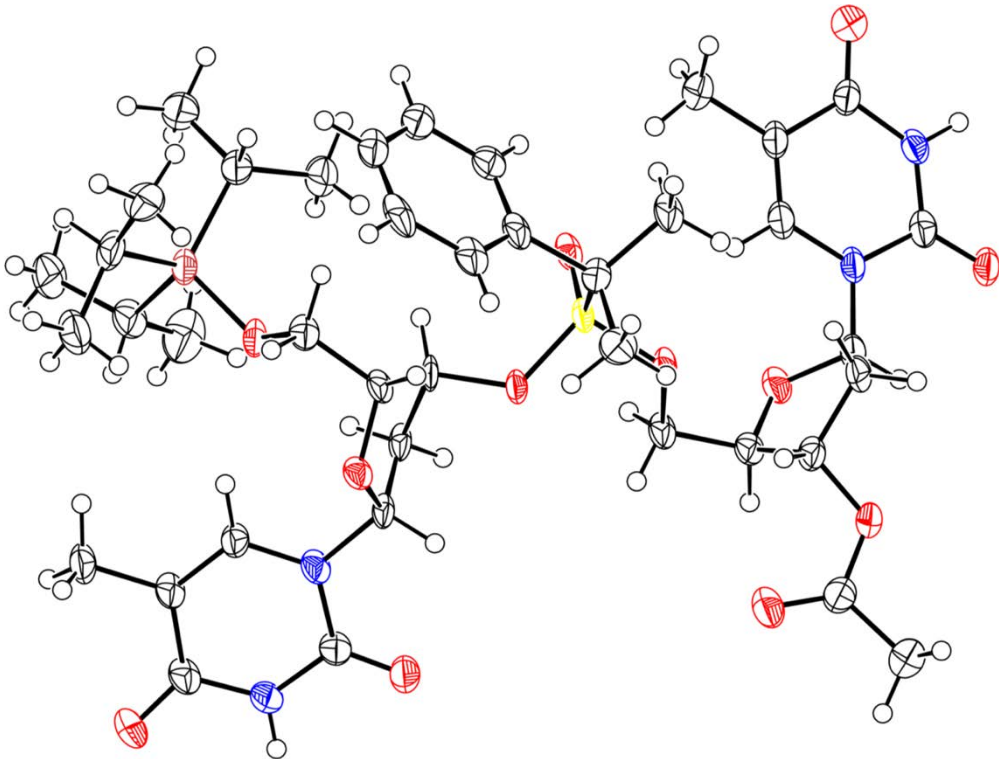

**Table S1.** Crystallographic data and structure refinement details of ($R_P$)-**3aa**

| | ($R_P$)-**3aa** |
|---|---|
| CCDC No. | 2263561 |
| formula | $C_{40}H_{59}N_4O_{12}PSi$ |
| fw | 846.97 |
| $T$ (K) | 123(2) |
| $\lambda$ (Å) | 0.71073 |
| cryst syst | orthorhombic |
| space group | $P2_12_12_1$ |
| $a$ (Å) | 9.8268(2) |
| $b$ (Å) | 13.5743(4) |
| $c$ (Å) | 32.2626(14) |
| $\alpha$ (deg) | 90 |
| $\beta$ (deg) | 90 |
| $\gamma$ (deg) | 90 |
| $V$ (Å$^3$) | 4303.6(2) |
| $Z$ | 4 |
| $D_{calc}$ (g·cm$^{-3}$) | 1.307 |
| $\mu$ (mm$^{-1}$) | 0.157 |
| F(000) | 1808 |
| cryst size (mm$^3$) | 0.2 × 0.04 × 0.01 |
| 2$\theta$ range (deg) | 5.12 to 54.97 |
| reflns collected | 22102 |
| indep reflns/$R_{int}$ | 9462 / 0.0632 |
| params | 534 |
| GOF on $F^2$ | 1.050 |
| $R_1$, $wR_2$ [$I$>2$\sigma$($I$)] | 0.0692, 0.1709 |
| $R_1$, $wR_2$ (all data) | 0.0909, 0.1798 |
| Flack parameter | -0.11(8) |

## 8. Derivatization of *tert*-Alkylphosphonates to the Phosphoramidites

### Procedure for synthesis of ($S_P$)-3aa-3 and ($R_P$)-3aa-3

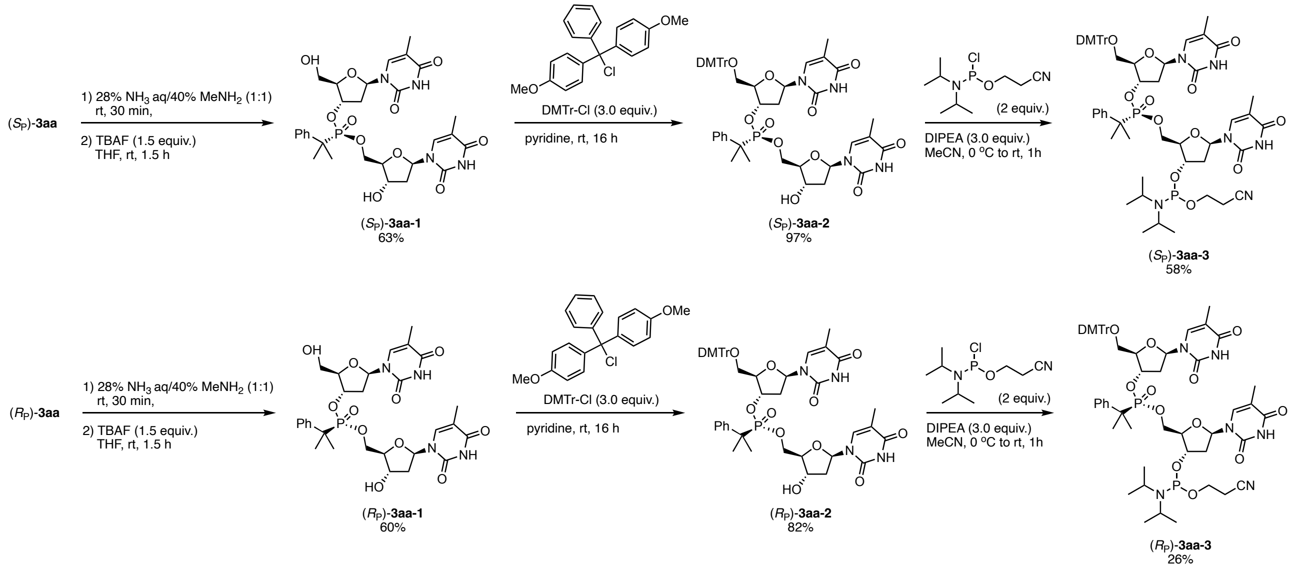


**Supplementary Fig. 3.** Derivatization of *tert*-alkylphosphonates to the phosphoramidites

**Deprotection**. ($S$P)-**3aa** (250 mg, 0.30 mmol) was disolved in ammonia solution (28% $NH_3$ aq.: 40% $MeNH_2$ aq. = 1:1). Stirred for 30 min, the reaction mixture was evaporated to remove solvents and reactants. The residue was used for the desilylation without further purification. The residue was disollved in THF (0.7 mL) and the THF solution of tetrabutylammonium fluoride (0.44 ml, 0.44 mmol) was added to the solution. The reaction mixture was stirred for 90 min and then was evaporated. The crude mixture was purified by silica-gel column chromatography (silica gel, hexane/EtOAc = 50:50 to EtOAc only, then EtOAc/MeOH = 100:0 to 90:10) to obtain ($S$P)-**3aa-1** (120 mg, 0.19 mmol, 63%). ($R$P)-**3aa-1** was synthesized using the same protocol (46 mg, 0.07 mmol, 60% from 0.12 mmol scale synthesis).

**DMTr protection.** To a solution of ($S$P)-**3aa-1** (120 mg, 0.19 mmol) in dry pyridine (2.0 mL) was added 4,4'-{chloro(phenyl)methylene}bis(methoxybenzene) (190 mg, 0.56 mmol) at room temperature. After stirring for 16 hours under a dry atmosphere, the reaction was quenched with MeOH (1 mL) and then the solvent was evaporated. The crude product was purified by column chromatography (silica gel, hexane/EtOAc = 20:80 to EtOAc only, then EtOAc/MeOH = 100:0 to 90:10) to give ($S$P)-**3aa-2** (170 mg, 0.18 mmol, 97 %) as amorphous solid. ($R$P)-**3aa-2** was synthesized using the same protocol (42 mg, 0.044 mmol, 82% from 0.05 mmol scale synthesis).

**Phosphoramidation.** To a solution of ($S$P)-**3aa-2** (160 mg, 0.17 mmol) in dry MeCN (4.0 ml) was added DIPEA (0.10 ml, 0.59 mmol) and 3-[{chloro(diisopropylamino)phosphaneyl}oxypropanenitrile] (0.094 ml, 0.34 mmol) at 0 °C. Cooling bath was removed and the mixture was allowed to be stirred at room temperature under dry nitrogen for 1 h. The reaction mixture was washed with hexane (5mL). The resulting oil was purified by column chromatography (silica gel, pre-wetted with hexane/EtOAc/triethylamine = 25:74:1, eluted with hexane/EtOAc = 25:75 to EtOAc only) to give ($S$P)-**3aa-3** (110 mg, 0.098 mmol, 58%) as a

colorless amorphous solid. ($R_P$)-**3aa-3** was synthesized using the same protocol, but the further purification with column chromatography (silica gel, pre-wetted with hexane/EtOAc/triethylamine = 25:74:1, eluted with hexane/EtOAc = 25:75 to EtOAc only) was required to result in lower yield. (12 mg, 0.04 mmol, 26% from 0.04 mmol scale synthesis).

## 9. Characterization Data for Phosphoramidites

**($S_P$)-3aa-1**

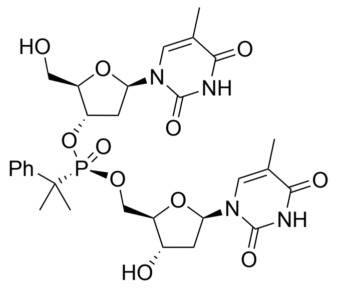


($S_P$)-**3aa-1**

Colorless amorphous solid. The sample contains inseparable impurities. **$^{1}$H NMR** (400 MHz, $CD_3OD$) $\delta$ 7.76 (m, 1H), 7.58–7.56 (m, 2H), 7.37–7.34 (m, 3H), 7.25 (m, 1H), 6.23–6.17 (m, 2H), 4.86 (m, 1H), 4.28 (m, 1H), 4.18–4.05 (m, 3H), 3.99 (m, 1H), 3.69–3.68 (m, 2H), 2.22 (m, 1H), 2.11–2.04 (m, 3H), 1.89–1.85 (m, 6H), 1.70–1.65 (m, 6H). **$^{13}$C NMR** (100.6 MHz, $CD_3OD$) $\delta$ 166.3, 166.3, 152.3, 152.2, 141.5 (d, $J_{C\text{–}P}$ = 5.1 Hz), 137.8, 137.5, 129.5 (×2C) (d, $J_{C\text{–}P}$ = 2.8 Hz), 128.9 (×2C) (d, $J_{C\text{–}P}$ = 5.4 Hz), 128.4 (d, $J_{C\text{–}P}$ = 2.8 Hz), 111.9, 111.8, 87.4 (d, $J_{C\text{–}P}$ = 3.5 Hz), 86.5, 86.2 (d, $J_{C\text{–}P}$ = 6.5 Hz), 85.9, 79.1 (d, $J_{C\text{–}P}$ = 7.6 Hz), 71.8, 67.3 (d, $J_{C\text{–}P}$ = 7.8 Hz), 62.4, 40.4 (d, $J_{C\text{–}P}$ = 135.8 Hz), 40.4, 39.3 (d, $J_{C\text{–}P}$ = 4.6 Hz), 24.4 (d, $J_{C\text{–}P}$ = 3.8 Hz), 24.1 (d, $J_{C\text{–}P}$ = 4.1 Hz), 12.7, 12.4. **$^{31}$P NMR** (162 MHz, $CD_3OD$) $\delta$ 33.7. **HRMS–ESI** (*m/z*): $[M+H]^+$ calcd for $C_{29}H_{38}N_4O_{11}P$, 649.2269; found, 649.2288.

**($S_P$)-3aa-2**

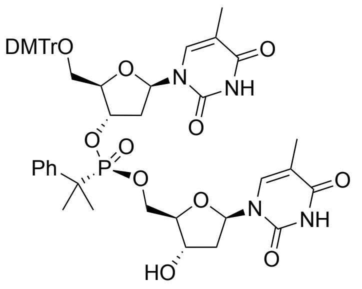


($S_P$)-**3aa-2**

Colorless amorphous solid. **$^{1}$H NMR** (400 MHz, $CDCl_3$) $\delta$ 9.09–8.98 (m, 2H), 7.58–7.56 (m, 2H), 7.39–7.19 (m, 13H), 7.08 (s, 1H), 6.84–6.82 (m, 4H), 6.30 (m, 1H), 6.13 (m, 1H), 4.81 (m, 1H), 4.19 (m, 1H), 4.12 (m, 1H), 3.99 (m, 1H), 3.85–3.78 (m, 8H), 3.42–3.34 (m, 3H), 2.28 (s, 1H), 2.10–2.08 (m, 2H), 1.95–1.89 (m, 4H), 1.69–1.65 (m, 6H), 1.38 (s, 3H). **$^{13}$C NMR** (100.6 MHz, $CDCl_3$) $\delta$ 163.6, 158.7, 158.7, 150.4, 150.1, 144.1, 139.9, 139.9, 135.3, 135.2, 135.1, 135.0, 130.1, 128.5, 128.5, 128.1, 128.0, 127.7, 127.7, 127.6, 127.5, 127.2, 113.3, 111.6, 111.1, 87.1, 85.0, 85.0, 84.8, 84.6, 84.2, 77.8, 77.7, 77.2, 70.5, 64.8, 63.1, 55.3, 40.0, 39.6, 38.7, 38.7, 38.7, 24.0, 24.0, 23.7, 23.7, 12.6, 11.6 (only

observed peaks). **$^{31}$P NMR** (162 MHz, $CDCl_3$) $\delta$ 34.7. **HRMS–ESI** (*m/z*): $[M+NH_4]^+$ calcd for $C_{50}H_{59}N_5O_{13}P$, 968.3842; found, 968.3840.

**(*S*P)-3aa-3**

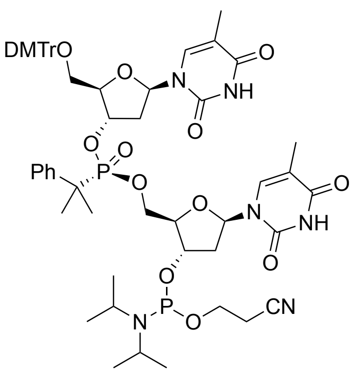


(*S*P)-**3aa-3**

Colorless amorphous solid. **$^{1}$H NMR** (400 MHz, $CDCl_3$) $\delta$ 8.45 (brs, 2H), 7.58–7.56 (m, 2H), 7.39–7.11 (m, 14H), 6.83–6.81 (m, 4H), 6.29 (m, 1H), 6.19 (m, 1H), 4.82 (m, 1H), 4.36 (m, 1H), 4.11–4.03 (m, 3H), 3.93–3.57 (m, 11H), 3.35–3.30 (m, 2H), 2.65–2.60 (m, 2H), 2.38 (m, 1H), 2.07–2.00 (m, 2H), 1.92–1.80 (m, 4H), 1.70–1.65 (m, 6H), 1.34–1.17 (m, 15H). **$^{13}$C NMR** (100.6 MHz, $CDCl_3$) $\delta$ 163.4, 163.3, 158.7, 158.7, 150.1, 150.0, 149.9, 144.1, 144.0, 140.2, 140.1, 140.1, 140.1, 135.2, 135.2, 135.1, 135.1, 134.9, 134.9, 130.1, 128.5, 128.1, 128.0, 127.7, 127.6, 127.5, 127.4, 127.2, 117.6, 113.3, 111.5, 111.3, 111.2, 87.1, 85.2, 85.0, 84.9, 84.3, 84.0, 83.9, 77.9, 77.9, 77.2, 73.2, 73.0, 72.9, 72.7, 64.6, 64.5, 64.5, 63.1, 63.1, 58.2, 58.1, 58.0, 57.9, 55.3, 43.4, 43.3, 40.0, 39.3, 39.2, 38.8, 38.7, 30.9, 24.6, 24.5, 24.5, 24.2, 24.2, 23.7, 23.6, 23.5, 23.5, 20.5, 20.4, 20.3, 20.3, 12.6, 12.5, 11.5 (only observed peaks). **$^{31}$P NMR** (162 MHz, $CDCl_3$) $\delta$ 149.3, 149.1, 34.4, 34.4. **HRMS–ESI** (*m/z*): $[M+NH_4]^+$ calcd for $C_{59}H_{76}N_7O_{14}P_2$, 1168.4920; found, 1168.4934.

**(*R*P)-3aa-1**

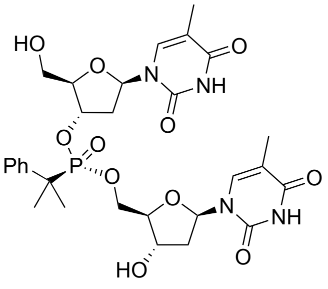


(*R*P)-**3aa-1**

Colorless amorphous solid. The sample contains inseprable impurities. **$^{1}$H NMR** (400 MHz, $CD_3OD$) $\delta$ 7.74 (m, 1H), 7.58–7.56 (m, 2H), 7.49 (m, 1H), 7.37–7.35 (m, 2H), 7.28 (m, 1H), 6.24–6.19 (m, 2H), 4.85 (m, 1H), 4.26 (m, 1H), 4.13 (m, 1H), 4.04 (m, 1H), 3.96 (m, 1H), 3.82 (m, 1H), 3.58 (m, 1H), 3.43 (m, 1H), 2.38 (m, 1H), 2.23–2.10 (m, 3H), 1.88–1.84 (m, 6H), 1.69–1.65 (m, 6H). **$^{13}$C NMR** (100.6 MHz, $CDCl_3$) $\delta$ 166.3, 166.3, 152.3, 152.2, 141.6 (d, $J_{C-P}$ = 5.1 Hz), 137.7 (×2C), 129.4 (×2C) (d, $J_{C-P}$ = 2.7 Hz), 128.9 (×2C) (d, $J_{C-P}$ = 5.4 Hz), 128.4 (d, $J_{C-P}$ = 3.0 Hz), 111.9, 111.9, 87.1 (d, $J_{C-P}$ = 6.0 Hz), 86.5, 86.2 (d, $J_{C-P}$ = 5.2 Hz), 86.0, 79.4 (d, $J_{C-P}$ = 7.6 Hz), 71.6, 68.0 (d, $J_{C-P}$ = 7.8 Hz), 62.5, 40.5 (d, $J_{C-P}$ = 136.0 Hz), 40.5, 40.2 (d, $J_{C-P}$ = 2.1 Hz), 24.4 (d, $J_{C-P}$ = 3.7 Hz), 24.1

(d, $J_{C–P}$ = 3.9 Hz), 12.5, 12.5. **$^{31}$P NMR** (162 MHz, $CD_3OD$) $\delta$ 33.3. **HRMS–ESI** (*m/z*): $[M+H]^+$ calcd for $C_{29}H_{38}N_4O_{11}P$, 649.2269; found, 649.2286.

**($R_P$)-3aa-2**

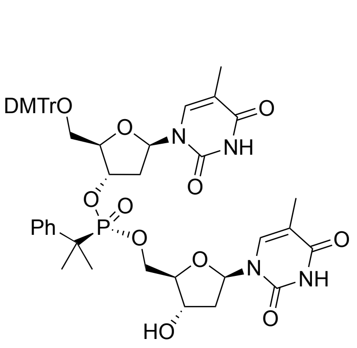

($R_P$)-**3aa-2**

Pale yellow amorphous solid. **$^1$H NMR** (400 MHz, $CDCl_3$) $\delta$ 9.62 (brs, 1H), 9.21 (brs, 1H), 7.52–7.45 (m, 3H), 7.30–7.16 (m, 12H), 7.06 (m, 1H), 6.85–6.83 (m, 4H), 6.34 (m, 1H), 6.21 (m, 1H), 4.66 (m, 1H), 4.44 (m, 1H), 4.25 (m, 1H), 4.12–3.99 (m, 3H), 3.81–3.79 (m, 7H), 3.26 (m, 1H), 2.78 (m, 1H), 2.54 (m, 1H), 2.44–2.29 (m, 2H), 2.08 (m, 1H), 1.93 (s, 3H), 1.64–1.57 (m, 6H), 1.29 (s, 3H). **$^{13}$C NMR** (100.6 MHz, $CDCl_3$) $\delta$ 163.7, 163.5, 163.5, 158.8, 158.8, 151.1, 150.3, 143.8, 140.0, 139.9, 135.6, 135.1, 135.0, 134.9, 130.1, 128.3, 128.3, 128.2, 128.0, 127.5, 127.4, 127.4, 127.3, 127.3, 113.3, 112.2, 111.1, 87.3, 85.3, 84.8, 84.7, 84.4, 84.3, 84.3, 79.7, 79.7, 77.2, 70.8, 65.4, 65.3, 63.3, 55.3, 40.3, 40.2, 39.0, 24.4, 24.4, 23.4, 23.4, 12.5, 11.5 (only observed peaks). **$^{31}$P NMR** (162 MHz, $CDCl_3$) $\delta$ 34.0. **HRMS–ESI** (*m/z*): $[M+NH_4]^+$ calcd for $C_{50}H_{59}N_5O_{13}P$, 968.3842; found, 968.3841.

**($R_P$)-3aa-3**

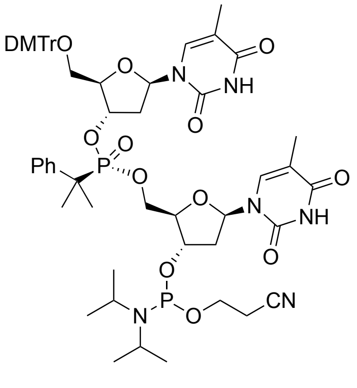

($R_P$)-**3aa-3**

Colorless amorphous solid. **$^1$H NMR** (400 MHz, $CDCl_3$) $\delta$ 8.44–8.40 (m, 2H), 7.48–7.10 (m, 16H), 6.85–6.82 (m, 4H), 6.36 (m, 1H), 6.19 (m, 1H), 4.90 (m, 1H), 4.45 (m, 1H), 4.21–4.06 (m, 3H), 3.86–3.59 (m, 11H), 3.26 (m, 1H), 2.87 (m, 1H), 2.65–2.62 (m, 2H), 2.51–2.34 (m, 3H), 2.01–1.88 (m, 4H), 1.62–1.56 (m, 6H), 1.30–1.18 (m, 15H). **$^{13}$C NMR** (100.6 MHz, $CDCl_3$) $\delta$ 163.5, 163.3, 158.8, 150.2, 150.2, 150.0, 143.9, 140.3, 140.2, 136.0, 135.8, 135.3, 135.3, 135.1, 134.9, 130.1, 128.3, 128.3, 128.2, 128.0, 127.6, 127.5, 127.5, 127.5, 127.3, 117.8, 113.1, 111.6, 111.2, 111.1, 87.2, 85.7, 84.5, 84.4, 84.2, 78.7, 78.6, 78.4, 77.2, 73.2, 65.5, 63.3, 58.1, 57.9, 55.3, 43.4, 43.4, 43.3, 43.2, 40.2, 40.2, 39.8, 39.4, 39.4, 39.1, 38.8, 38.8, 24.6, 24.5, 24.5, 24.4, 23.6, 20.5, 20.4, 20.4, 20.4, 12.,3 11.5, 11.4 (only observed peaks). **$^{31}$P NMR** (162 MHz, $CDCl_3$) $\delta$ 149.3, 148.9, 33.4, 33.3. **HRMS–ESI** (*m/z*): $[M+NH_4]^+$ calcd for $C_{59}H_{76}N_7O_{14}P_2$, 1168.4920; found, 1168.4943.

## 10. Procedure for Automated Solid-Support Synthesis of Oligonucleotides

The automated solid-support synthesis of oligonucleotides were performed on nS8-II (GeneDesign). The detailed protocol for nS8-II was given in the table below. All the oligonucleotides were synthesized using low-loaded Unylinker$^{TM}$ universal solid support (0.5 umol, 42.6 umol/g, ChemGenes, N-4000-10). After the synthetic cycle, the solid support was dried with an argon gas flush and placed into a 2 mL microtube. 500 uL of ammonia methyl amine (AMA) solution (28% $NH_3$ aq./ 40% $MeNH_2$ aq. = 1/1) was added to the tube and the support was treated for 30 minutes at 58 °C. The remaining ammonia and $MeNH_2$ was evaporated and purified with the cartridge purification system (Glen-Pak$^{TM}$ DNA Purification Cartridge, #60-5200, according to the manual provided) to afford aqueous solution containing the purified oligonucleotide. UV absorbance of the solution was measured and then the sample was lyophilyzed to afford a white powder. Yield was calculated based on absorbance of 260 nm, in which the molar absorption coefficient of the oligonucleotides (**Oligo-1-8**) was calculated from the known 260 nm absorption coefficient of thymine in aqueous media.

**Synthesis cycle for oligonucleotides (0.5 umol scale)**

| Step | Reaction | Reagent | Volume (1CV = 500 uL) | Reaction time |
|---|---|---|---|---|
| 1 | Detritylation | Dichloroacetic Acid-Toluene (3:97) | 2 CV (incubation twice) | 90 sec (60 + 30 seq) |
| 2 | Coupling | Activator solution: 0.1M Amidite solution = 40:60 | 12 eq. | 4 min for **(*R*$_P$/*S*$_P$)-3aa-3**<br>1 min for dT amidite |
| 3 | Oxidation | Iodine Solution (abt. 0.05mol/L)][Pyridine:Water(9:1) | 1 CV (incubation) | 30 sec |
| 4 | Capping | 1-Methylimidazole-Acetonitrile (2:8) + Acetic Anhydride-2,6-Lutidine-Acetonitrile (2:3:5) | 1 CV (incubation) | 90 sec |

Note: Before starting the coupling cycle, conduct deblocking of DMTr group on solid-support sufficiently in order to prevent generation of -1nt oligomer byproduct. The dimer amidite **(*R*$_P$/*S*$_P$)-3aa-3** was dehydrated by azeotropic distillation and dried in vacuo with $P_2O_5$ before use.

## UPLC-MS's LC charts and ESI-QTOF MS spectra of oligonucleotides

Analysis conditions with Waters UPLC MS systems

| | |
|---|---|
| System | Waters, UPLC Xevo G2-XS QTof |
| column | ACQUITY UPLC OST C18 Column, 1.7 µm, 2.1 x 50 mm |
| Buffer | A･･･240 mM HFIP, 7 mM TEA (5% MeOH)<br>B･･･240 mM HFIP, 7 mM TEA (100% MeOH) |
| Gradient | 10 to 80 for 6min |
| Flow rate | 0.2 ml/min |
| Column oven temperature | 60 °C |
| Detector | UV 260 nm |

# Oligo-1 ($R_P$)-TTTTTTTTT$_{Alk}$T

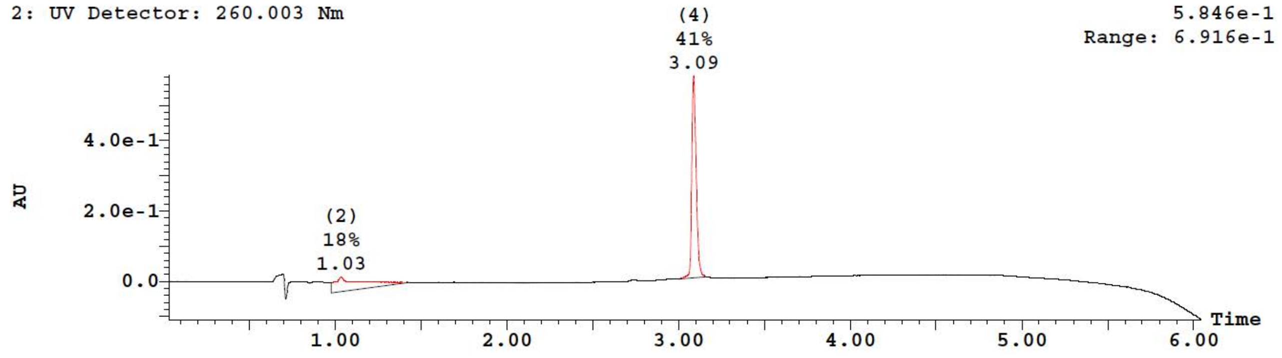


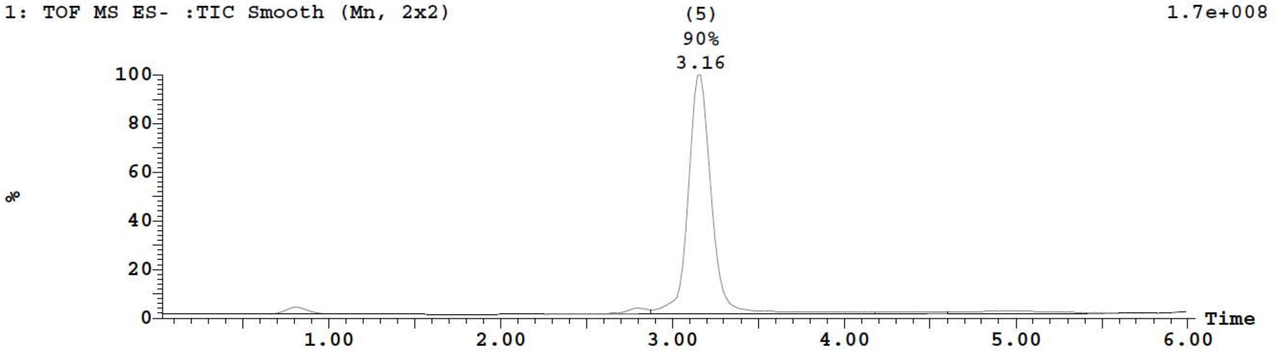


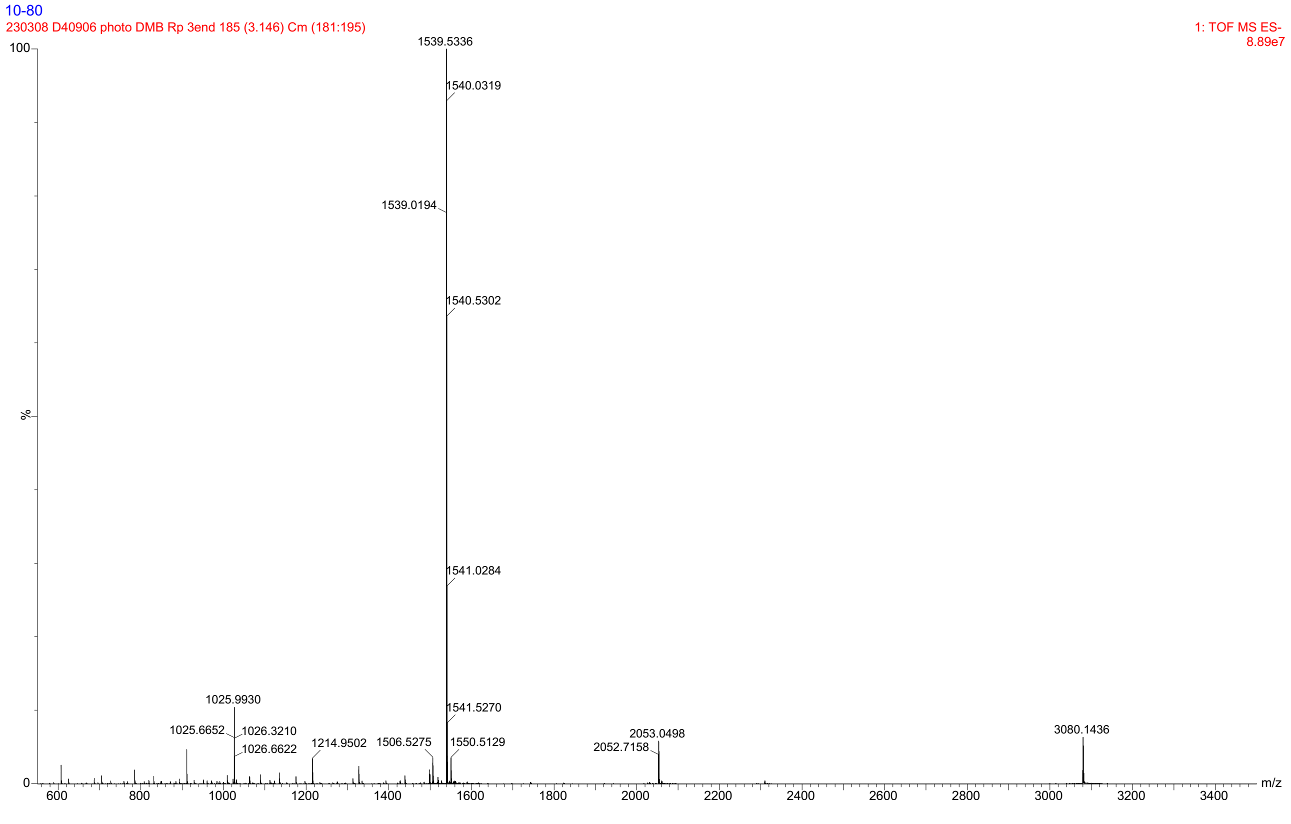

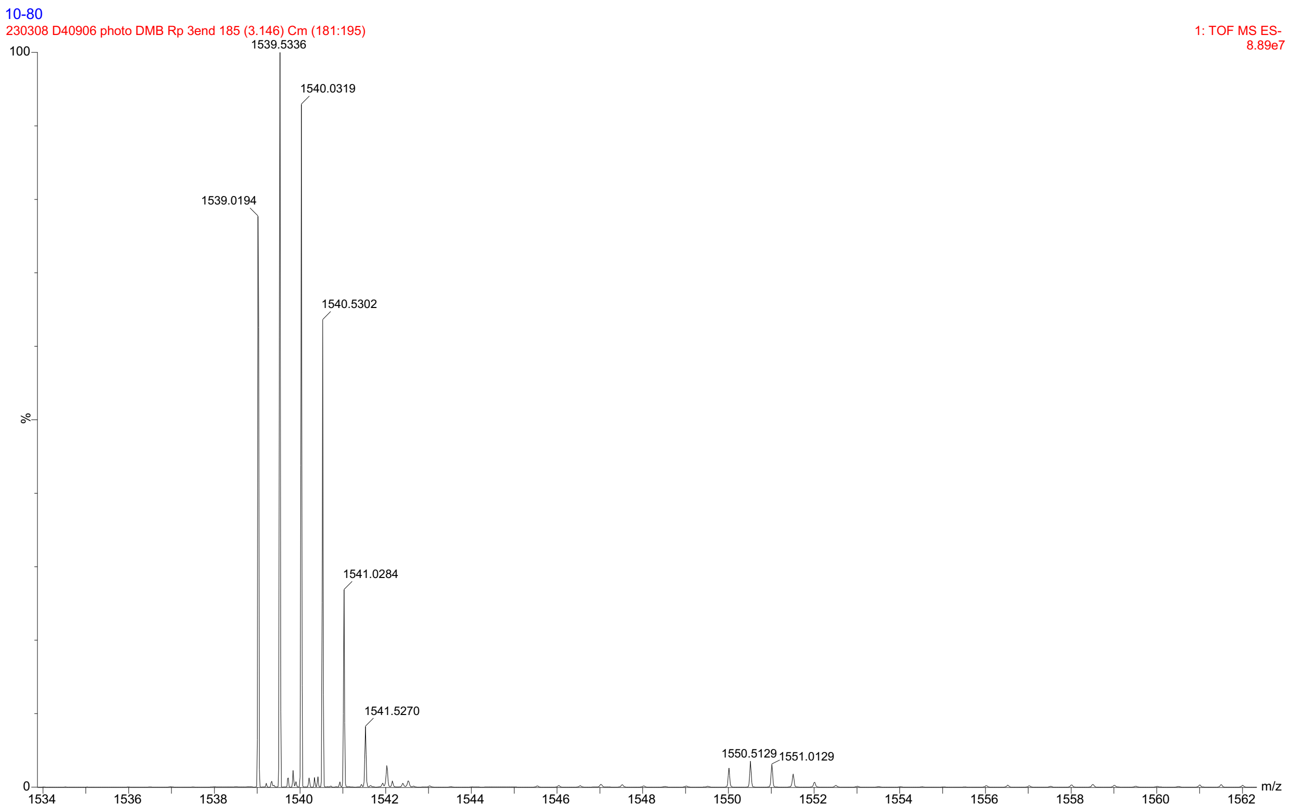
10-80
230308 D40906 photo DMB Rp 3end 185 (3.146) Cm (181:195)
1: TOF MS ES-
8.89e7
1539.5336
1540.0319
1539.0194
1540.5302
1541.0284
1541.5270
1550.5129
1551.0129
100
%
0
m/z
1534
1536
1538
1540
1542
1544
1546
1548
1550
1552
1554
1556
1558
1560
1562

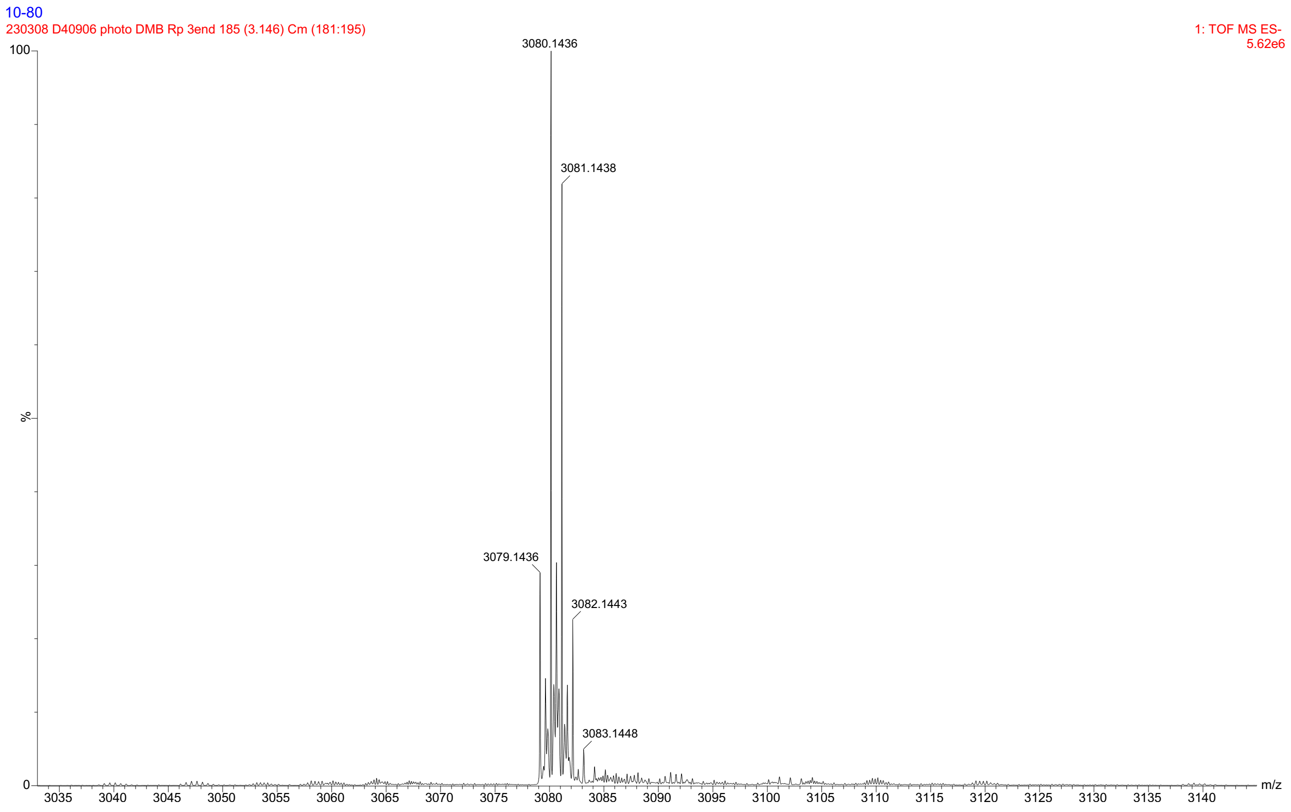
10-80
230308 D40906 photo DMB Rp 3end 185 (3.146) Cm (181:195)
1: TOF MS ES-
5.62e6
3080.1436
3081.1438
3079.1436
3082.1443
3083.1448
100
%
0
m/z
3035
3040
3045
3050
3055
3060
3065
3070
3075
3080
3085
3090
3095
3100
3105
3110
3115
3120
3125
3130
3135
3140

**Oligo-2 ($S_P$)-TTTTTTTTT$_{Alk}$T**

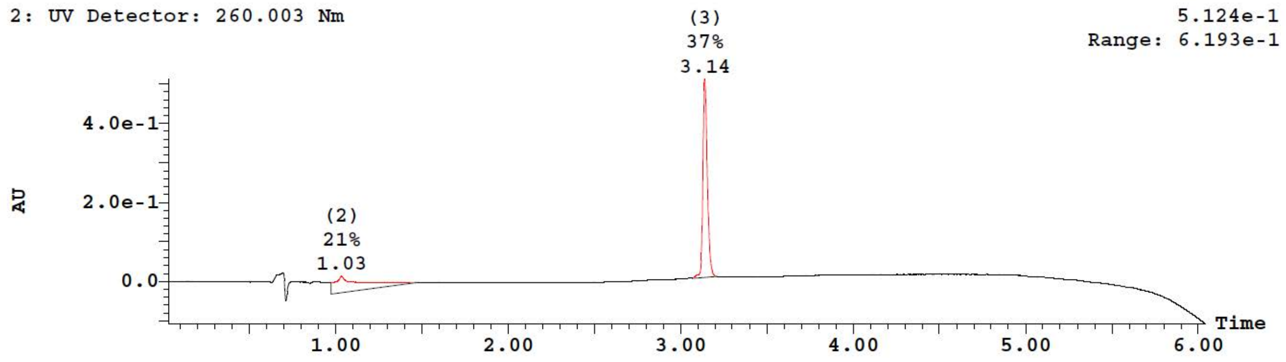


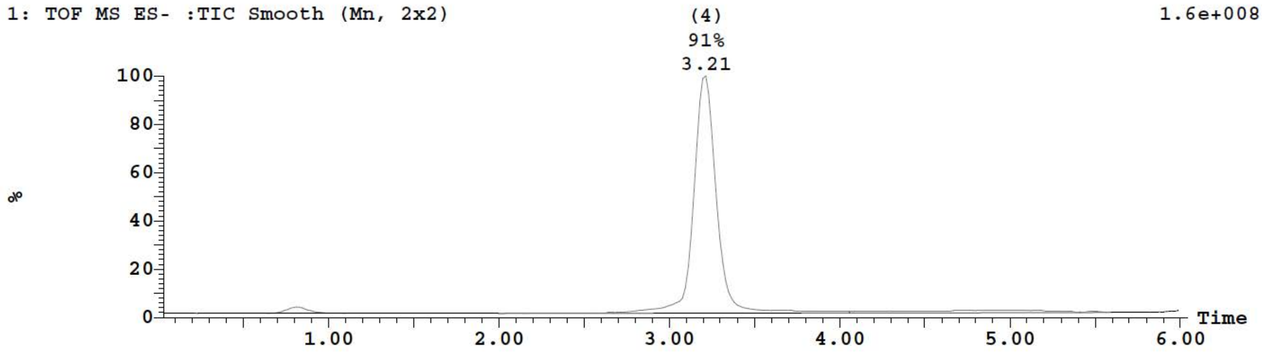


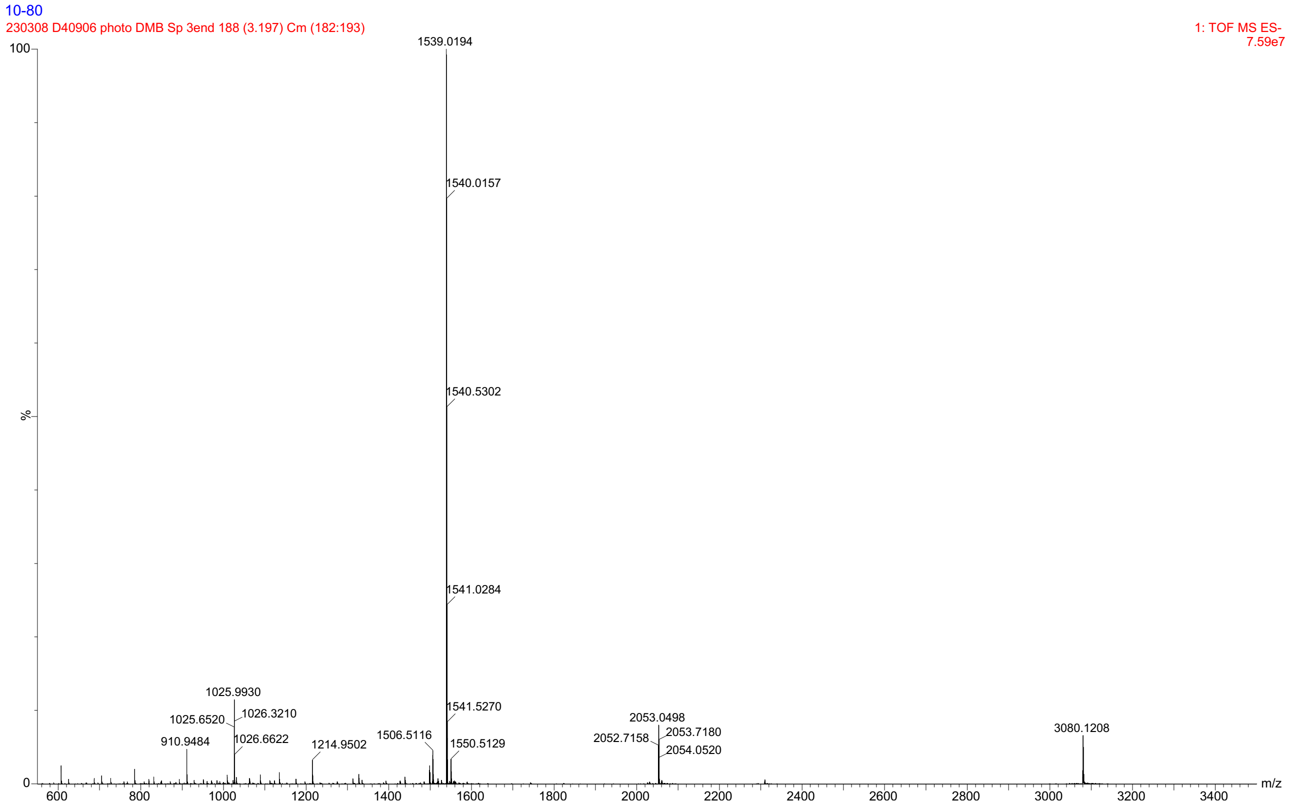

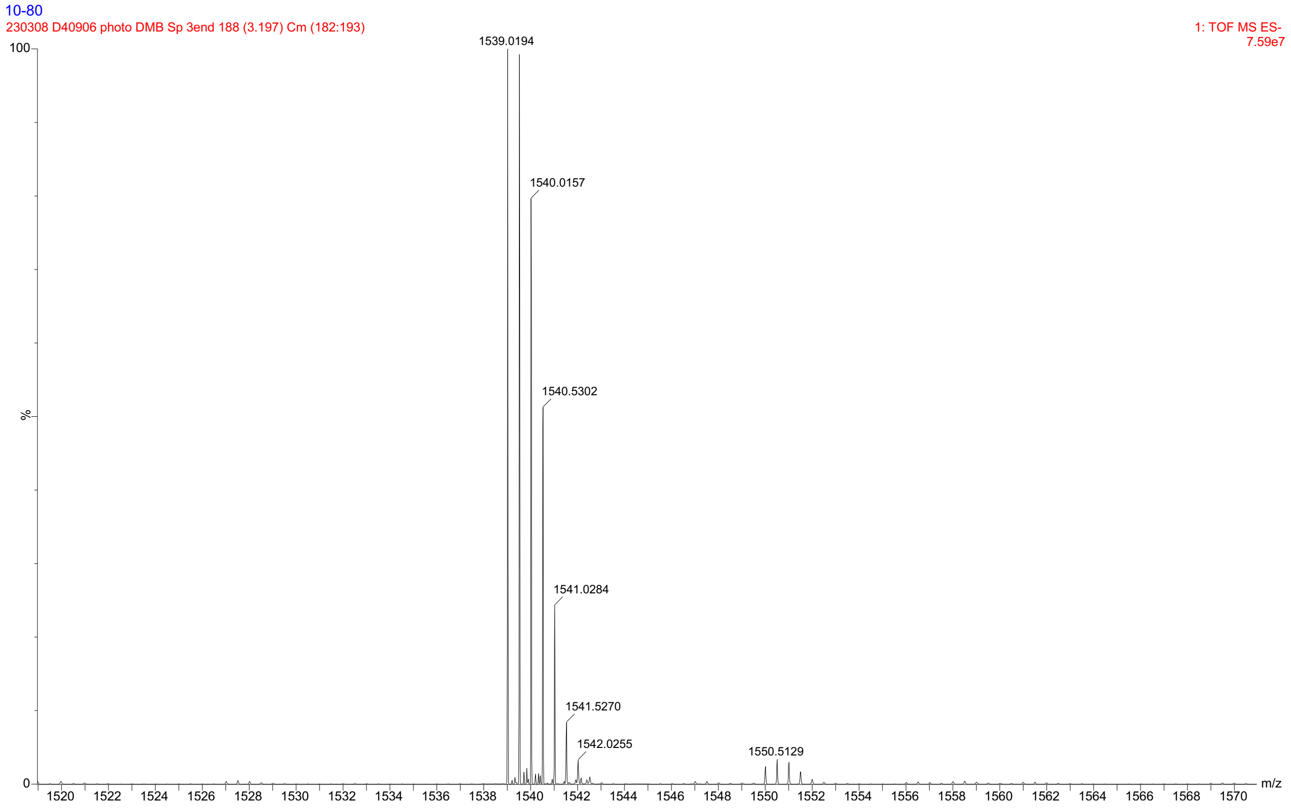
10-80
230308 D40906 photo DMB Sp 3end 188 (3.197) Cm (182:193)
1: TOF MS ES-
7.59e7
1539.0194
1540.0157
1540.5302
1541.0284
1541.5270
1542.0255
1550.5129
100
%
0
m/z
1520
1522
1524
1526
1528
1530
1532
1534
1536
1538
1540
1542
1544
1546
1548
1550
1552
1554
1556
1558
1560
1562
1564
1566
1568
1570

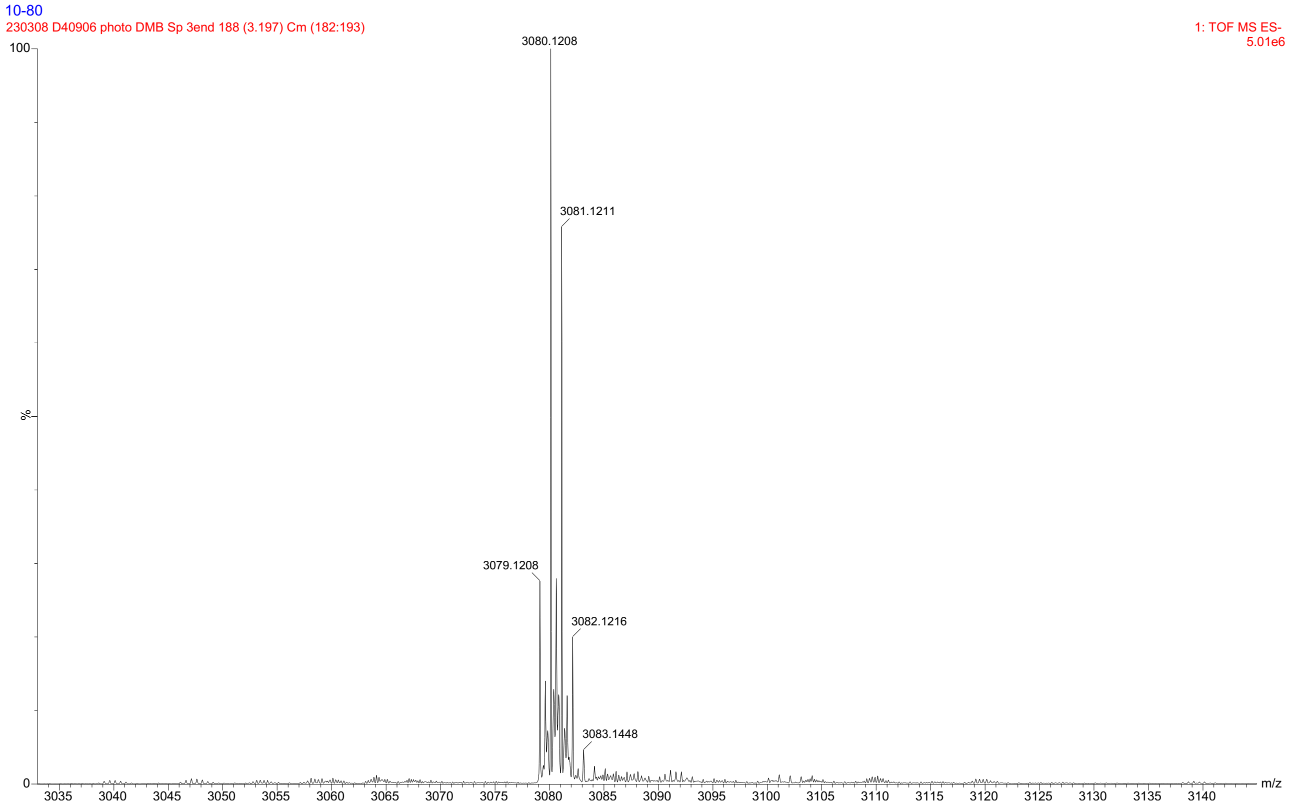
10-80
230308 D40906 photo DMB Sp 3end 188 (3.197) Cm (182:193)
1: TOF MS ES-
5.01e6
3080.1208
3081.1211
3079.1208
3082.1216
3083.1448
100
%
0
m/z
3035
3040
3045
3050
3055
3060
3065
3070
3075
3080
3085
3090
3095
3100
3105
3110
3115
3120
3125
3130
3135
3140

## Oligo-3 ($(R_P)$)-$TTTTT_{Alk}TTTTT$

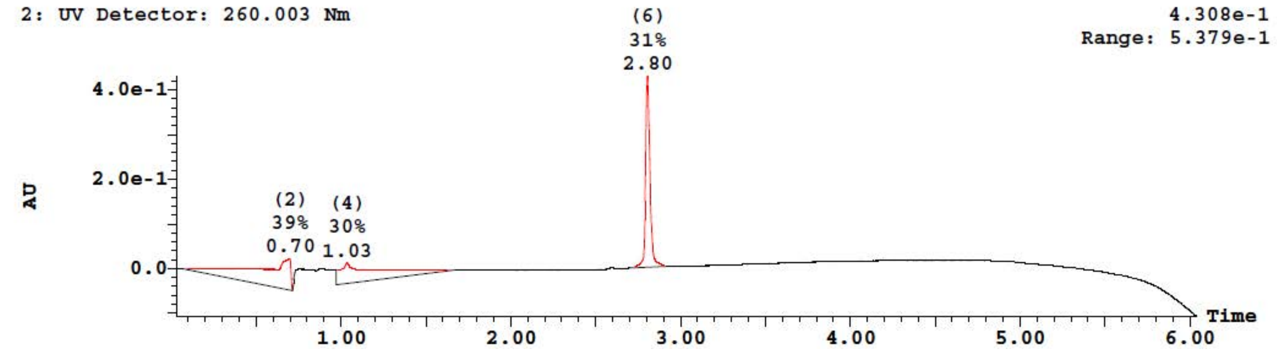


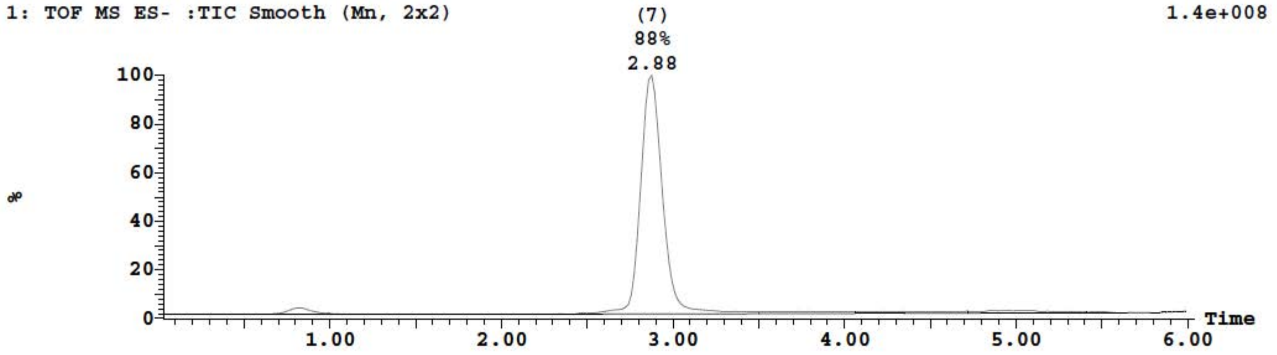


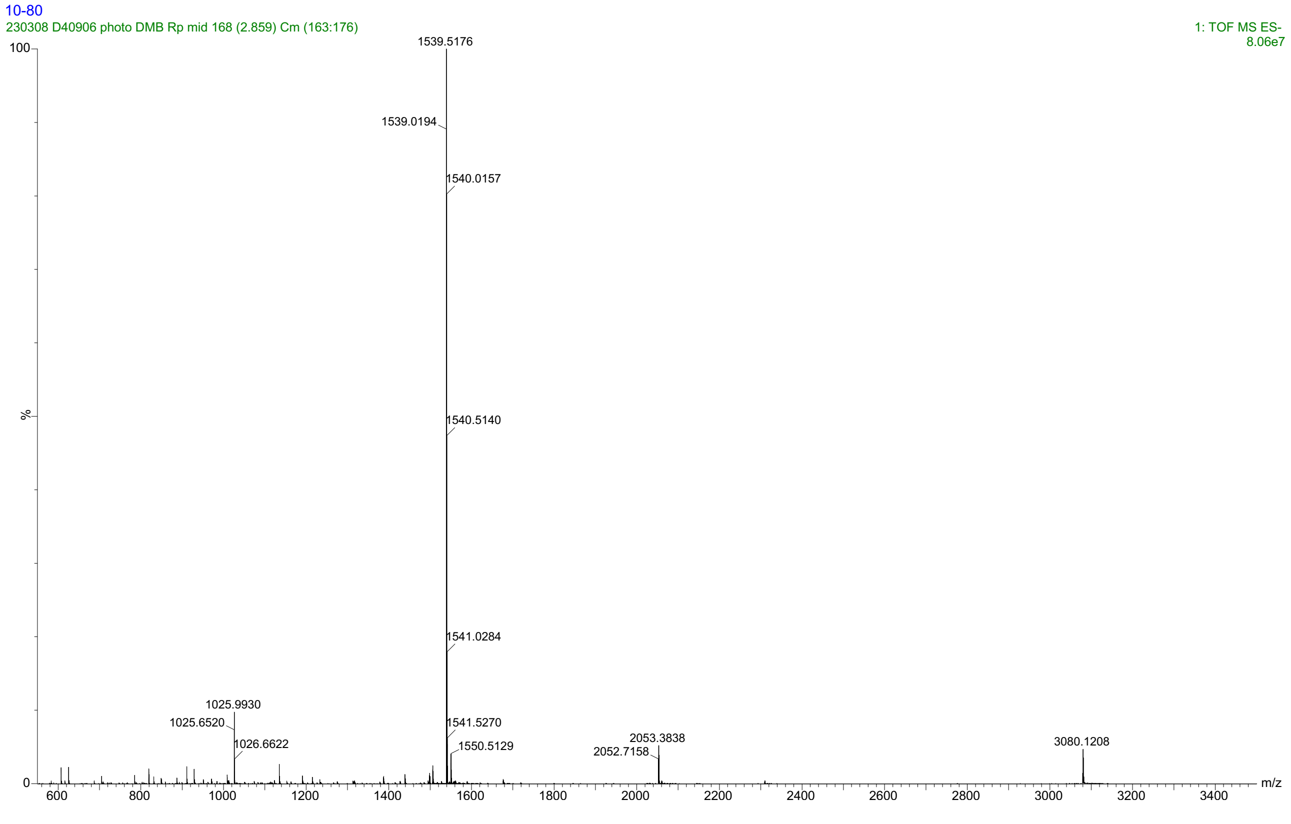

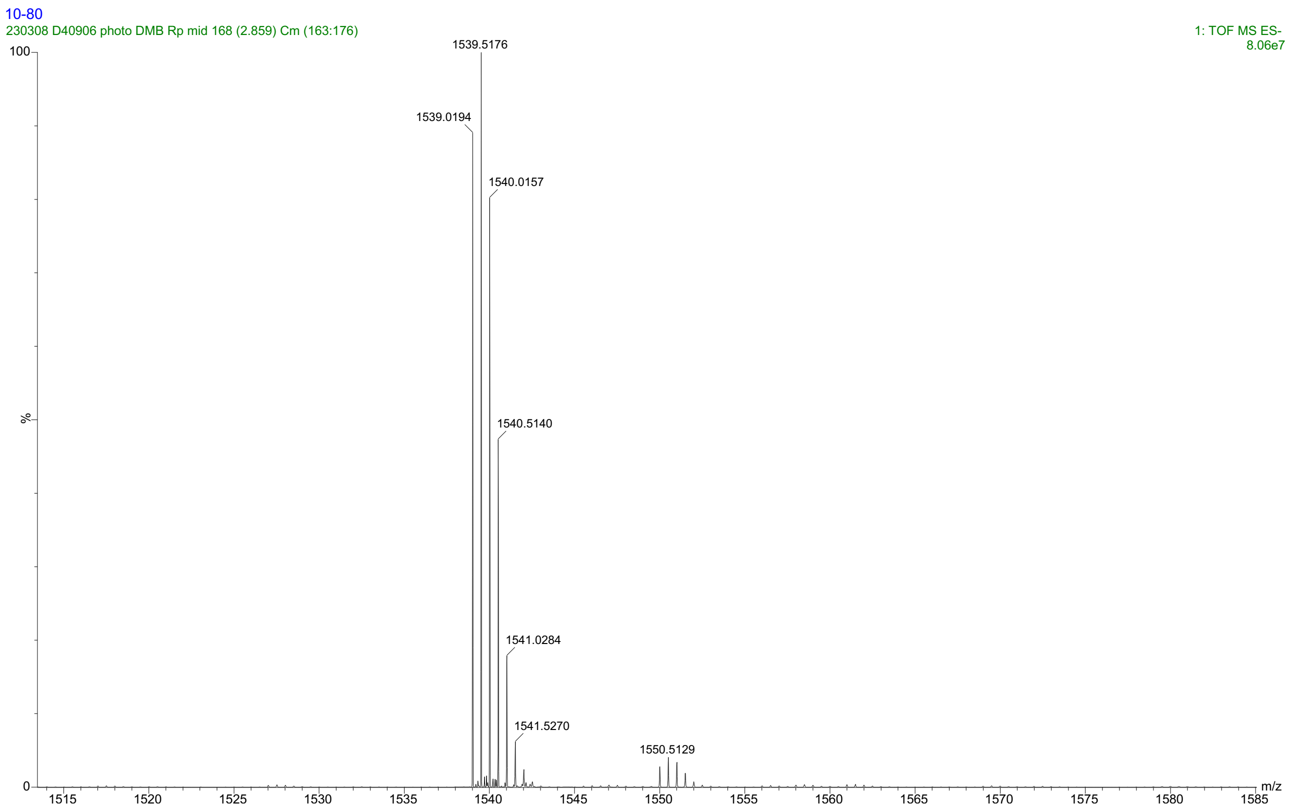
10-80
230308 D40906 photo DMB Rp mid 168 (2.859) Cm (163:176)
1: TOF MS ES-
8.06e7
1539.5176
1539.0194
1540.0157
1540.5140
1541.0284
1541.5270
1550.5129
%
m/z

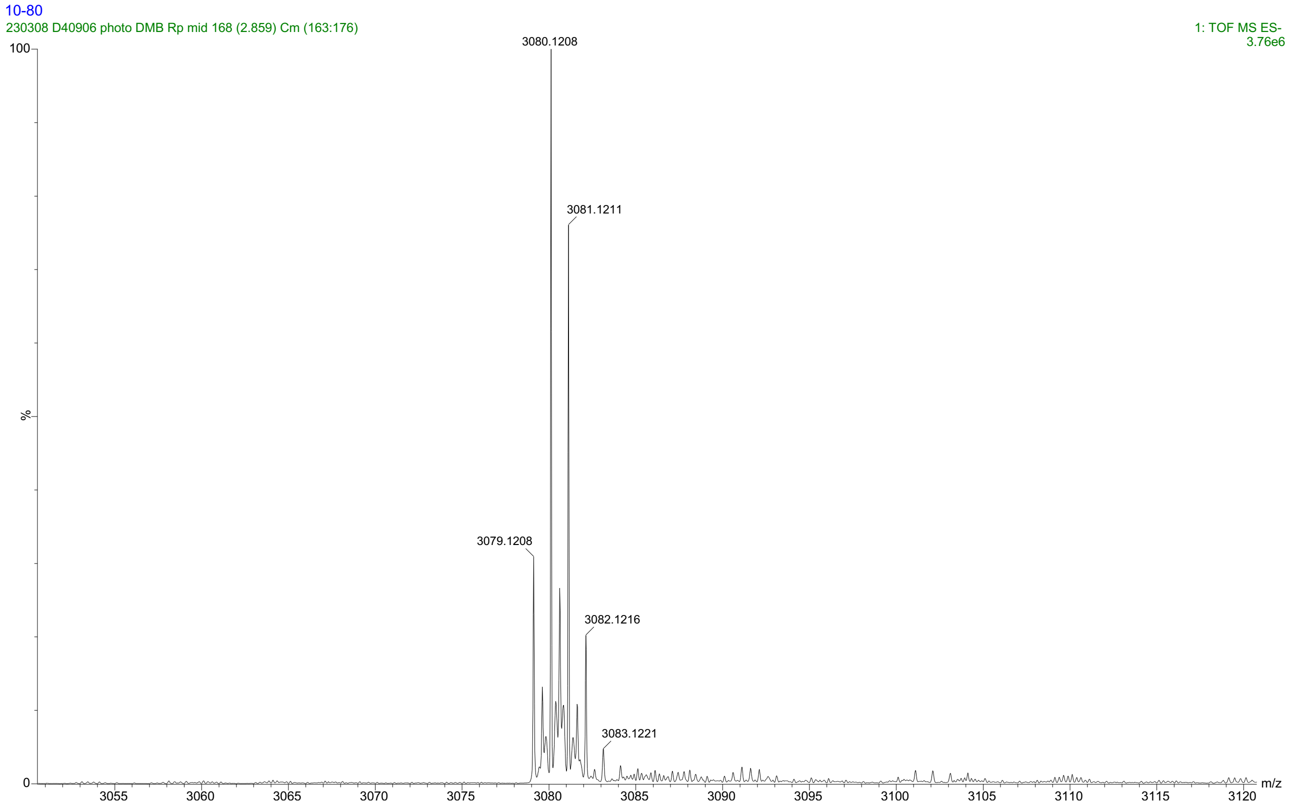
10-80
230308 D40906 photo DMB Rp mid 168 (2.859) Cm (163:176)
1: TOF MS ES-
3.76e6
3080.1208
3081.1211
3079.1208
3082.1216
3083.1221
%
m/z

**Oligo-4 ($S_P$)-TTTTT$_{Alk}$TTTTT**

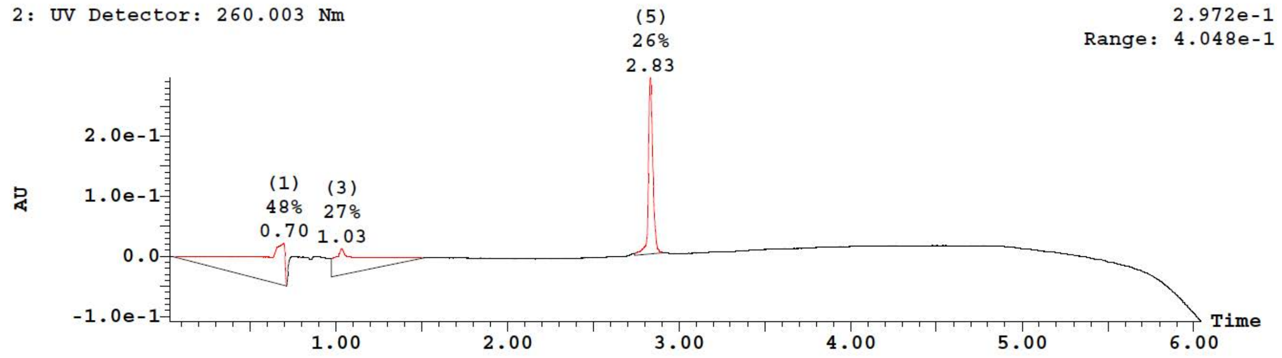


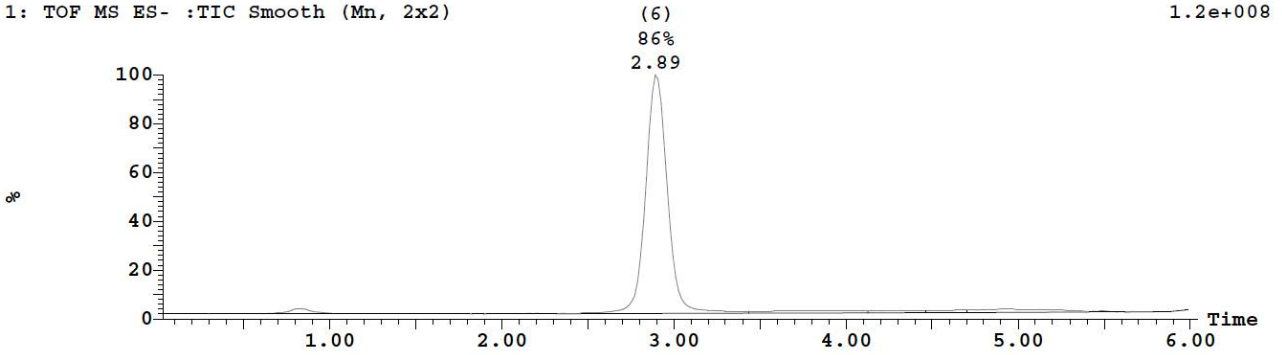


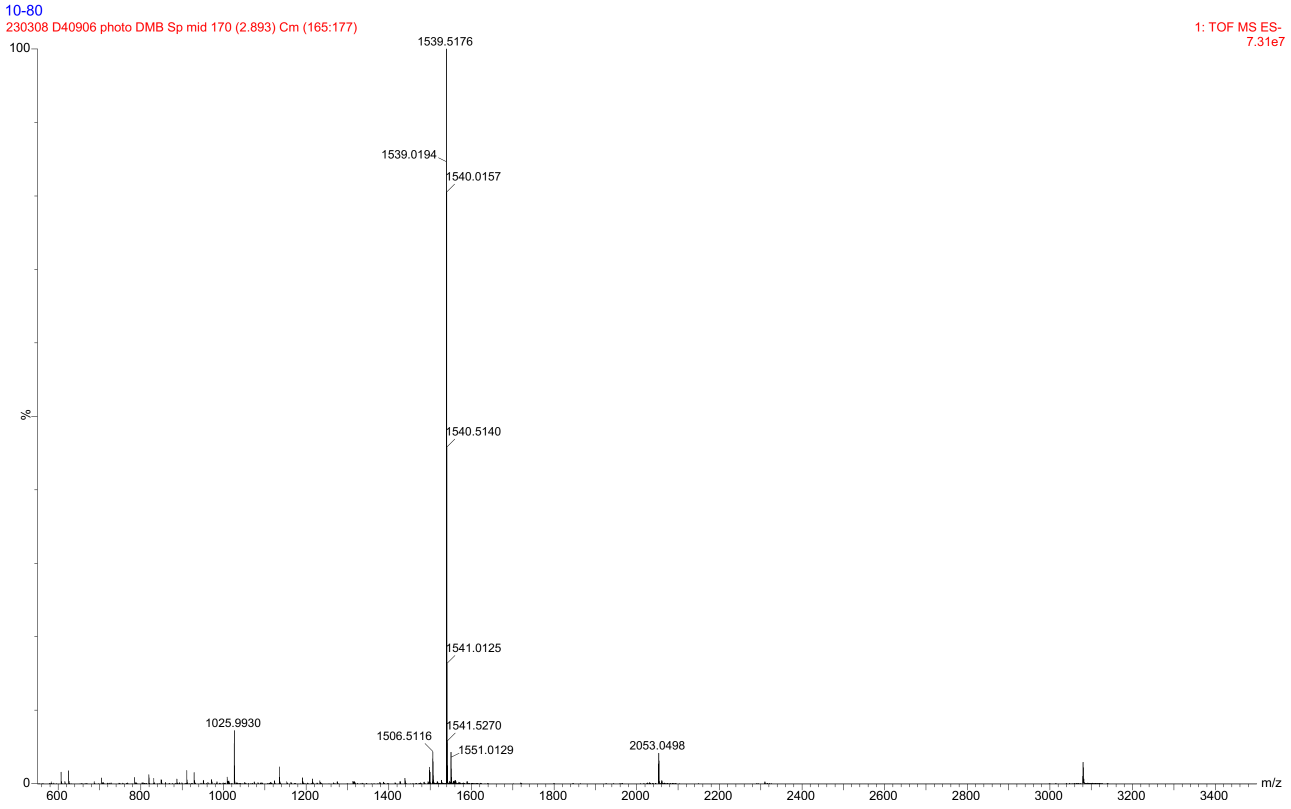

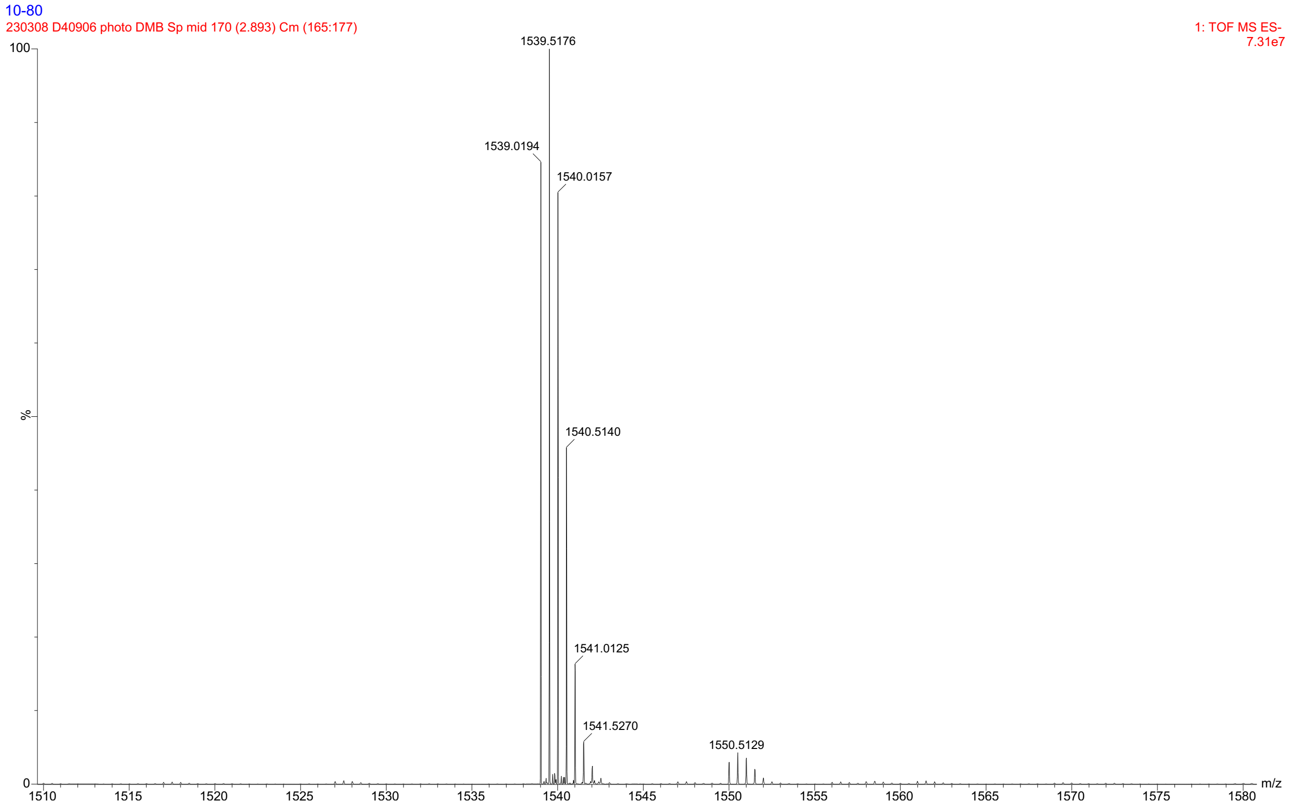
10-80
230308 D40906 photo DMB Sp mid 170 (2.893) Cm (165:177)
1: TOF MS ES-
7.31e7
1539.5176
1539.0194
1540.0157
1540.5140
1541.0125
1541.5270
1550.5129
100
%
0
m/z
1510
1515
1520
1525
1530
1535
1540
1545
1550
1555
1560
1565
1570
1575
1580

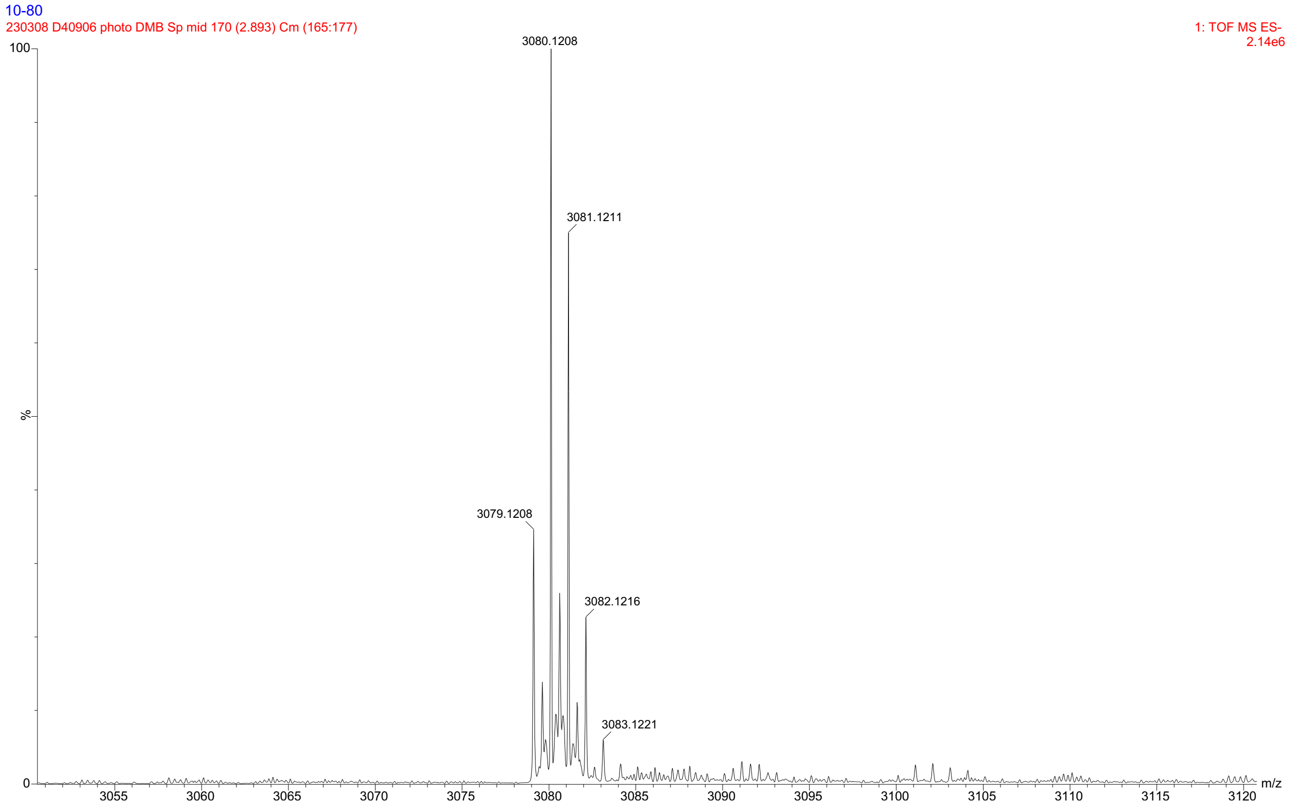
10-80
230308 D40906 photo DMB Sp mid 170 (2.893) Cm (165:177)
1: TOF MS ES-
2.14e6
3080.1208
3081.1211
3079.1208
3082.1216
3083.1221
100
%
0
m/z
3055
3060
3065
3070
3075
3080
3085
3090
3095
3100
3105
3110
3115
3120

**Oligo-5 ($S_P$,$S_P$)-T$_{Alk}$TTTTTTTTT$_{Alk}$T**

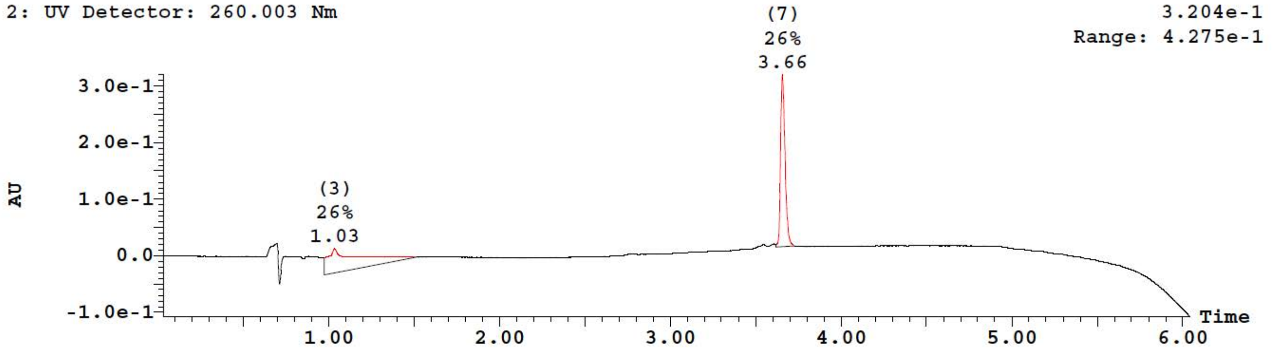


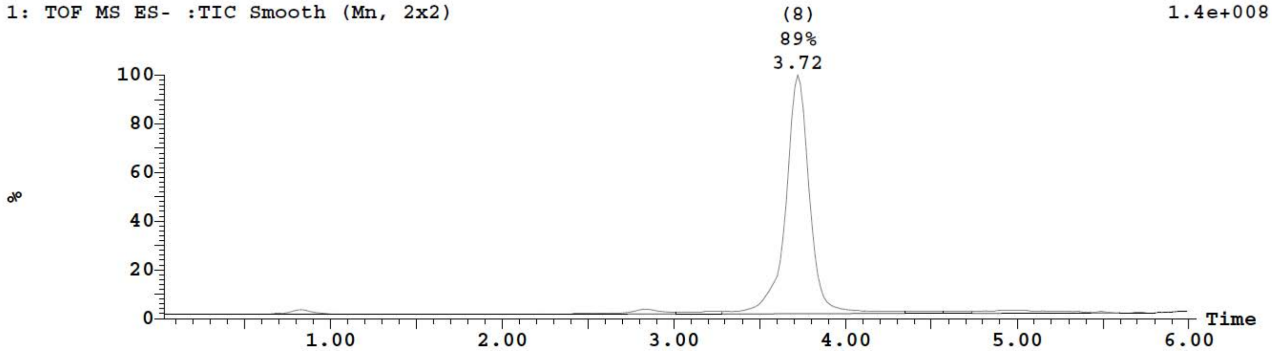


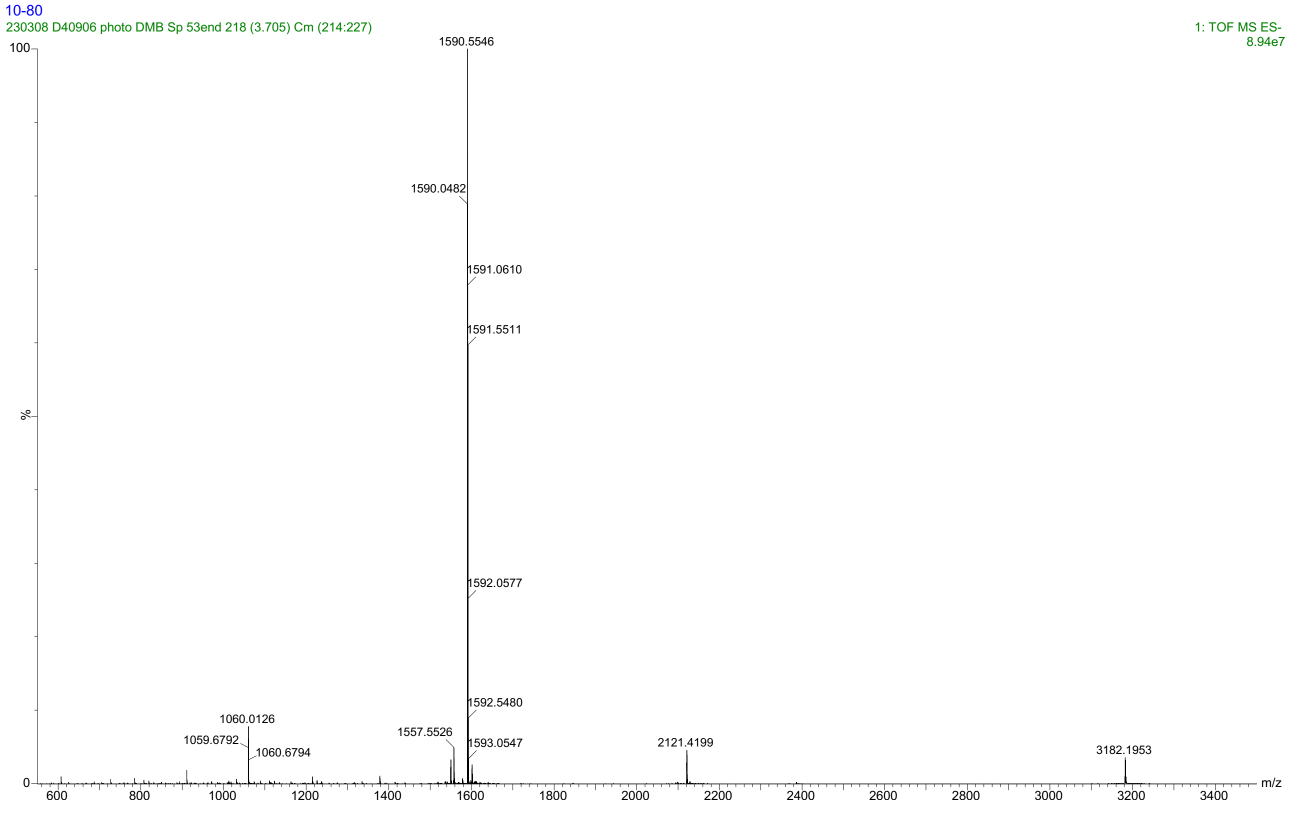

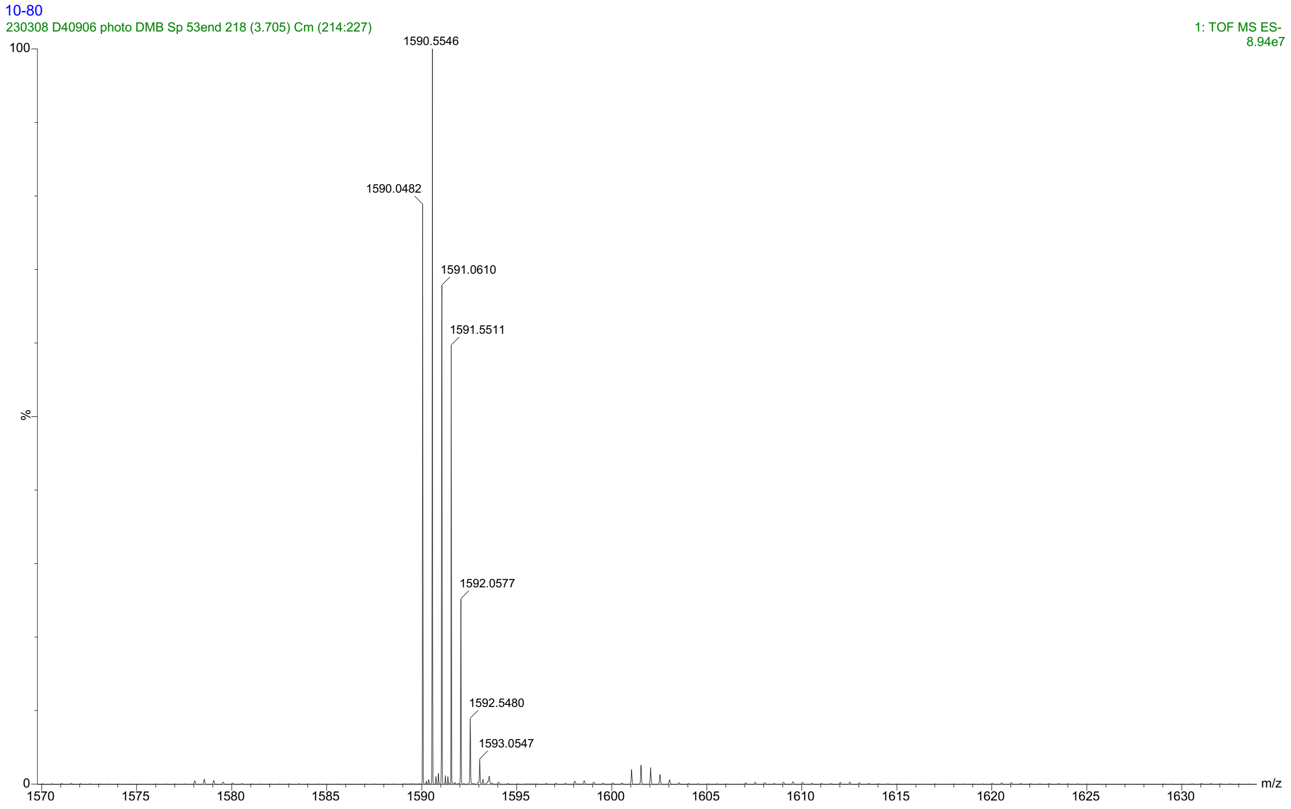
10-80
230308 D40906 photo DMB Sp 53end 218 (3.705) Cm (214:227)
1: TOF MS ES-
8.94e7
1590.5546
1590.0482
1591.0610
1591.5511
1592.0577
1592.5480
1593.0547
100
%
0
1570
1575
1580
1585
1590
1595
1600
1605
1610
1615
1620
1625
1630
m/z

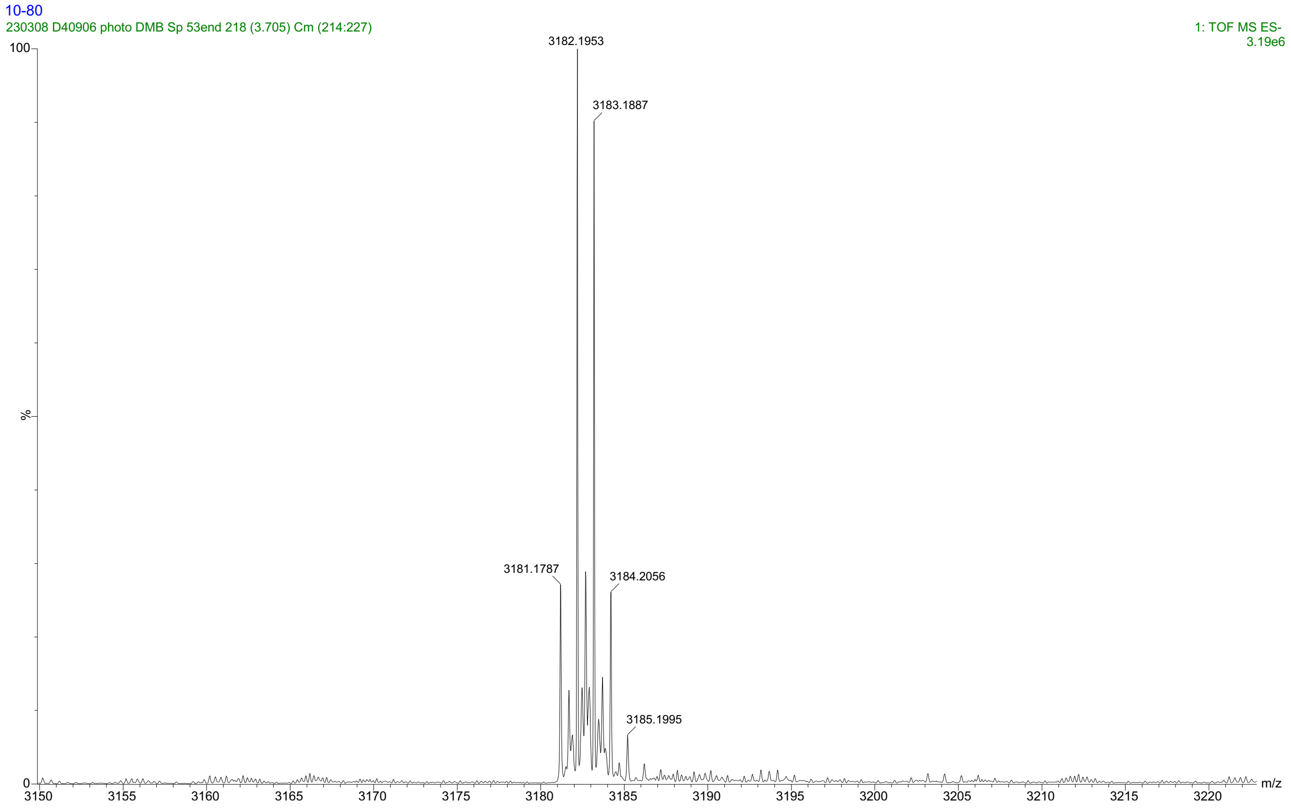
10-80
230308 D40906 photo DMB Sp 53end 218 (3.705) Cm (214:227)
1: TOF MS ES-
3.19e6
3182.1953
3183.1887
3181.1787
3184.2056
3185.1995
100
%
0
3150
3155
3160
3165
3170
3175
3180
3185
3190
3195
3200
3205
3210
3215
3220
m/z

## Oligo-6 TTTTTTTTTT

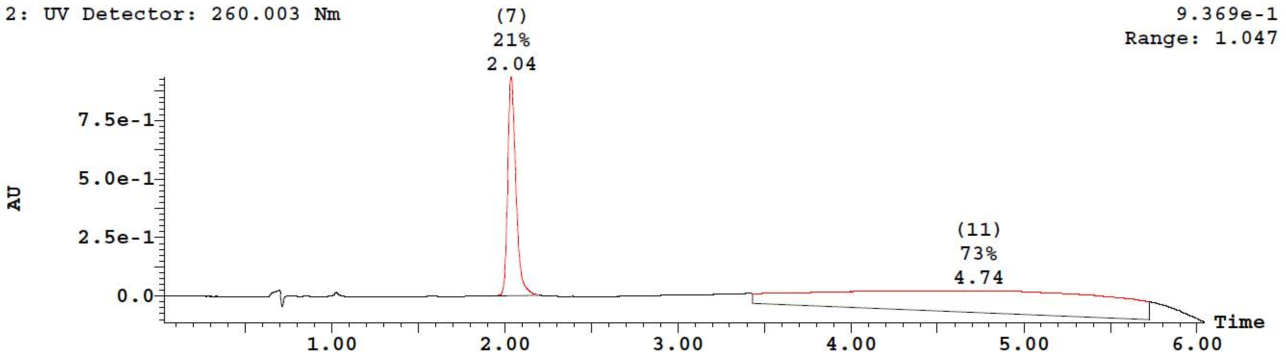


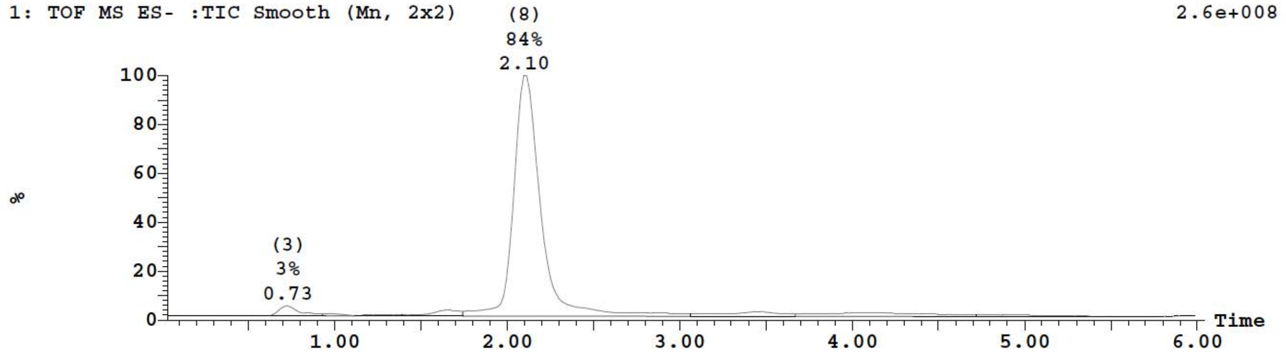


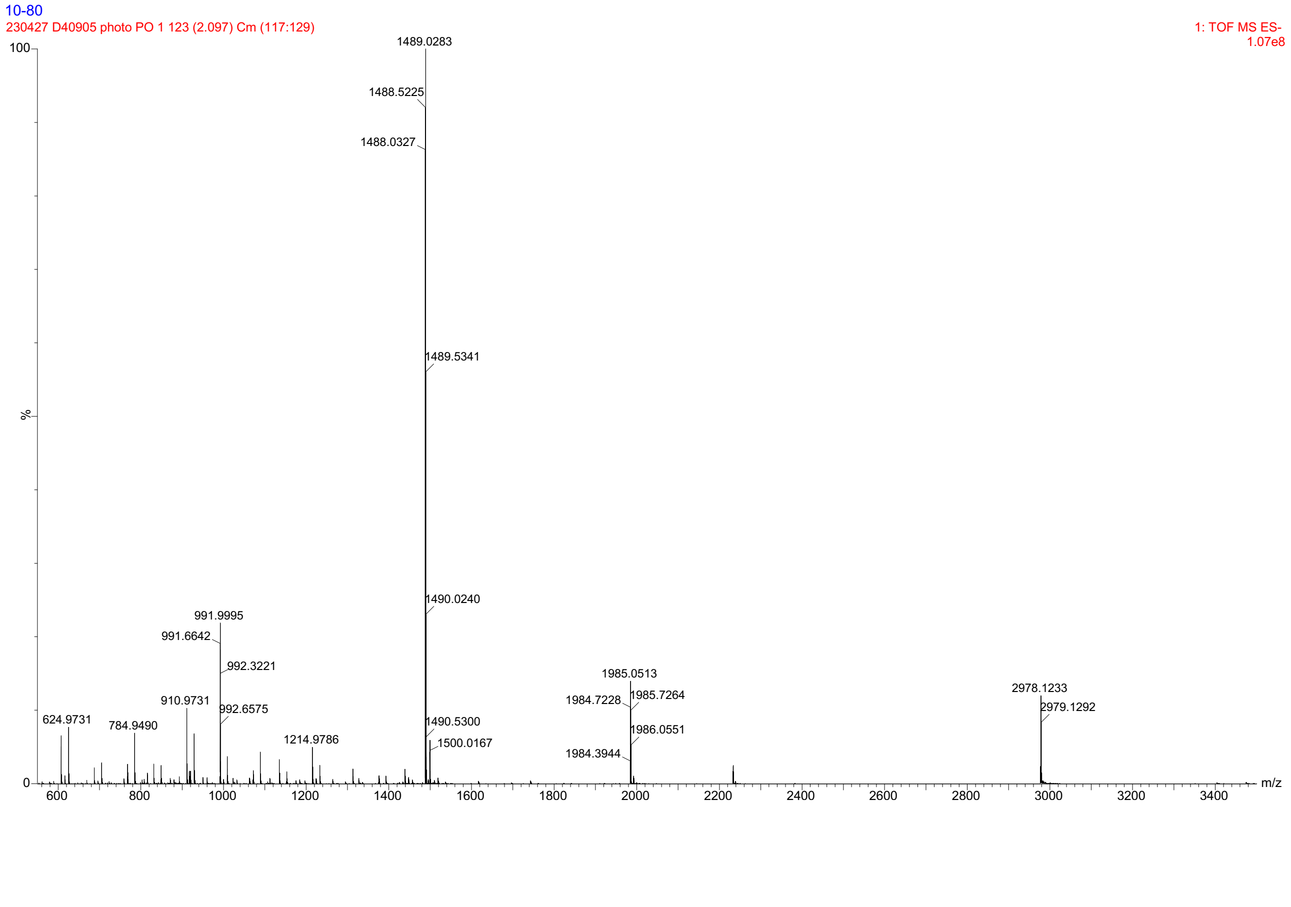

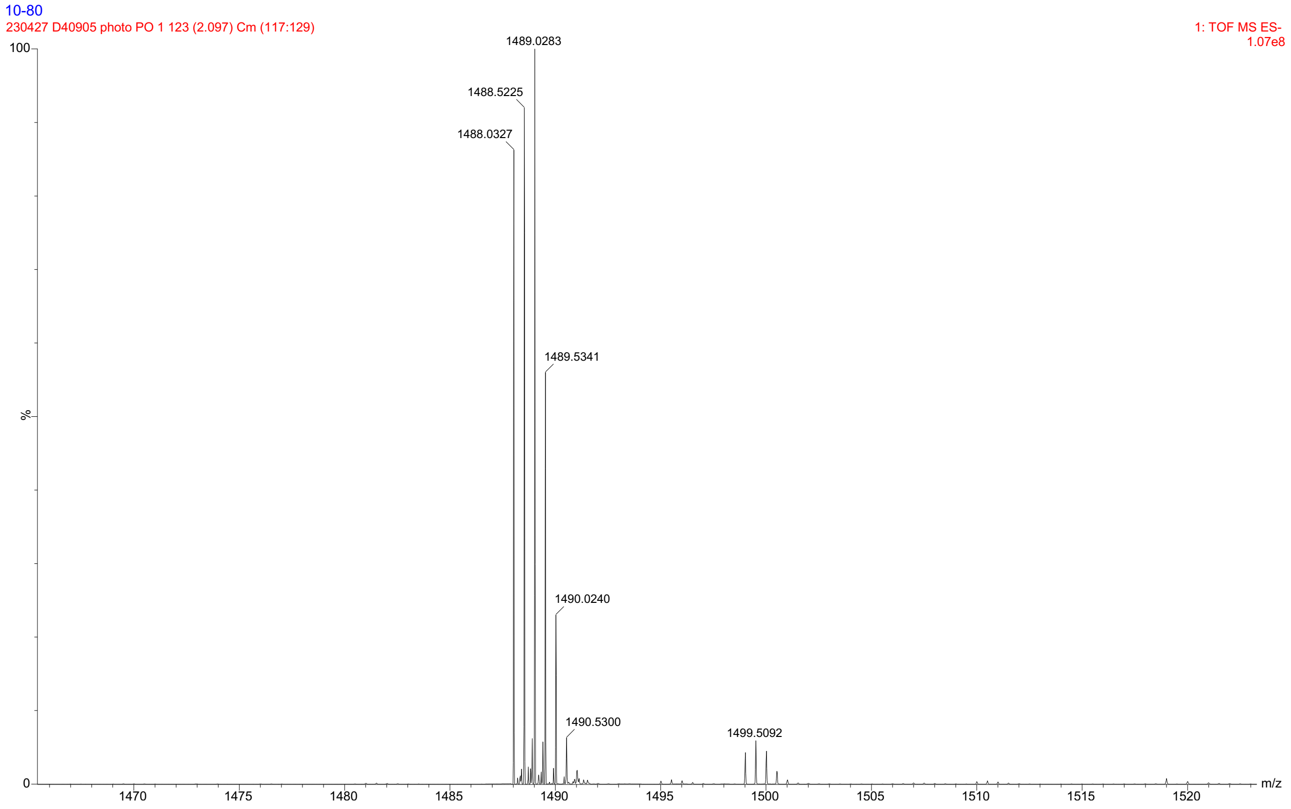

10-80
230427 D40905 photo PO 1 123 (2.097) Cm (117:129)
1: TOF MS ES-
1.07e8
1489.0283
1488.5225
1488.0327
1489.5341
1490.0240
1490.5300
1499.5092
100
%
0
1470
1475
1480
1485
1490
1495
1500
1505
1510
1515
1520
m/z


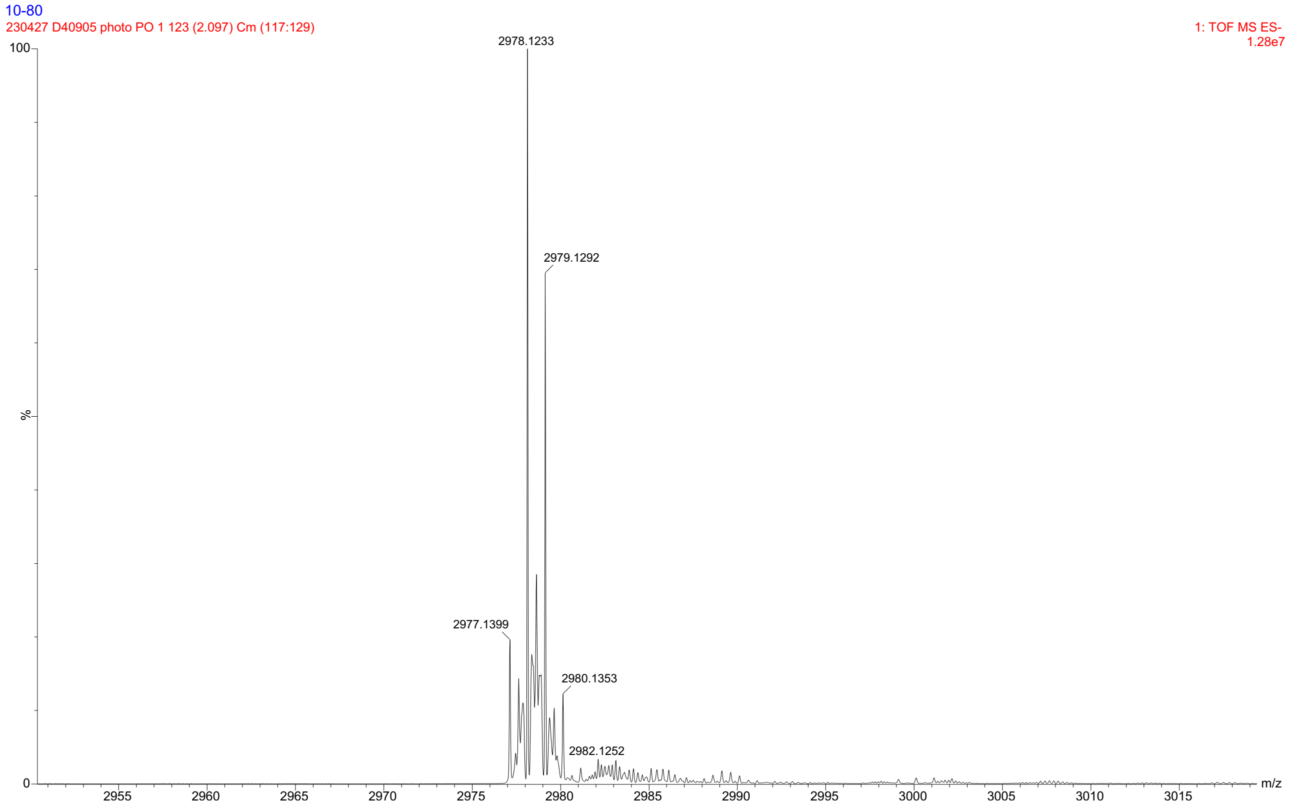

10-80
230427 D40905 photo PO 1 123 (2.097) Cm (117:129)
1: TOF MS ES-
1.28e7
2978.1233
2979.1292
2977.1399
2980.1353
2982.1252
100
%
0
2955
2960
2965
2970
2975
2980
2985
2990
2995
3000
3005
3010
3015
m/z

**Oligo-7 TTTTTTTTT$_{OET}$T**

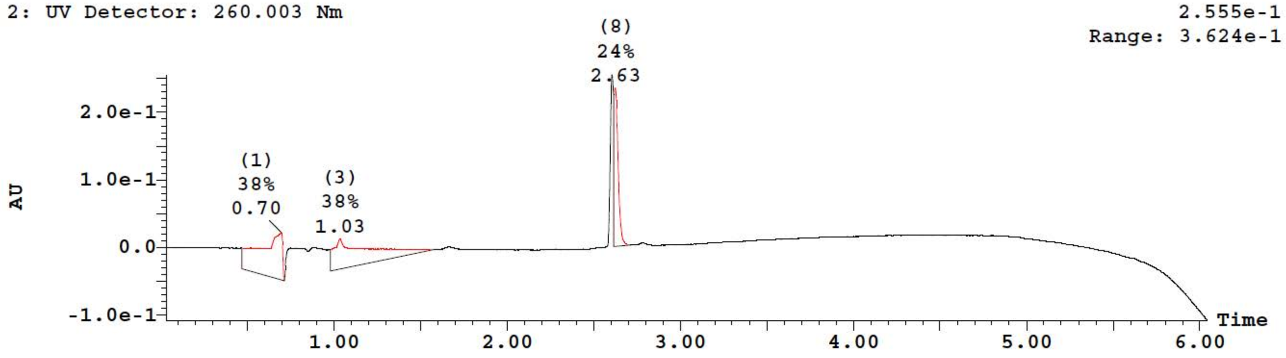


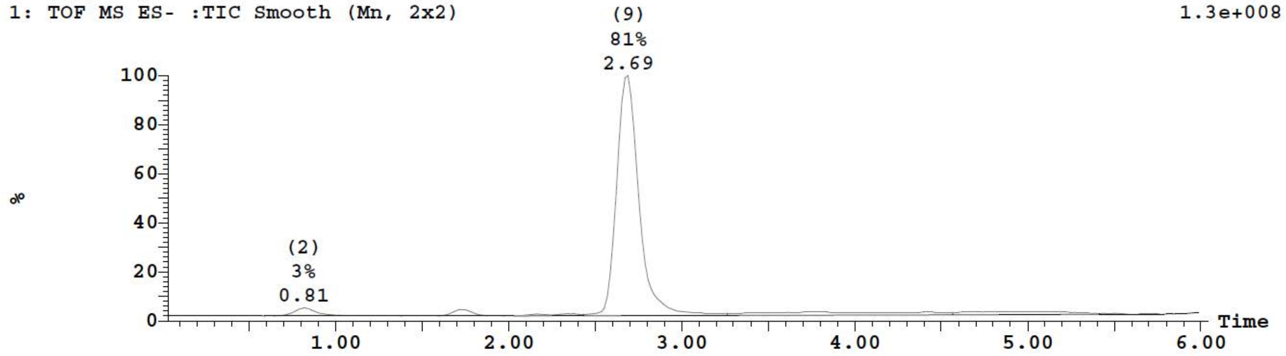


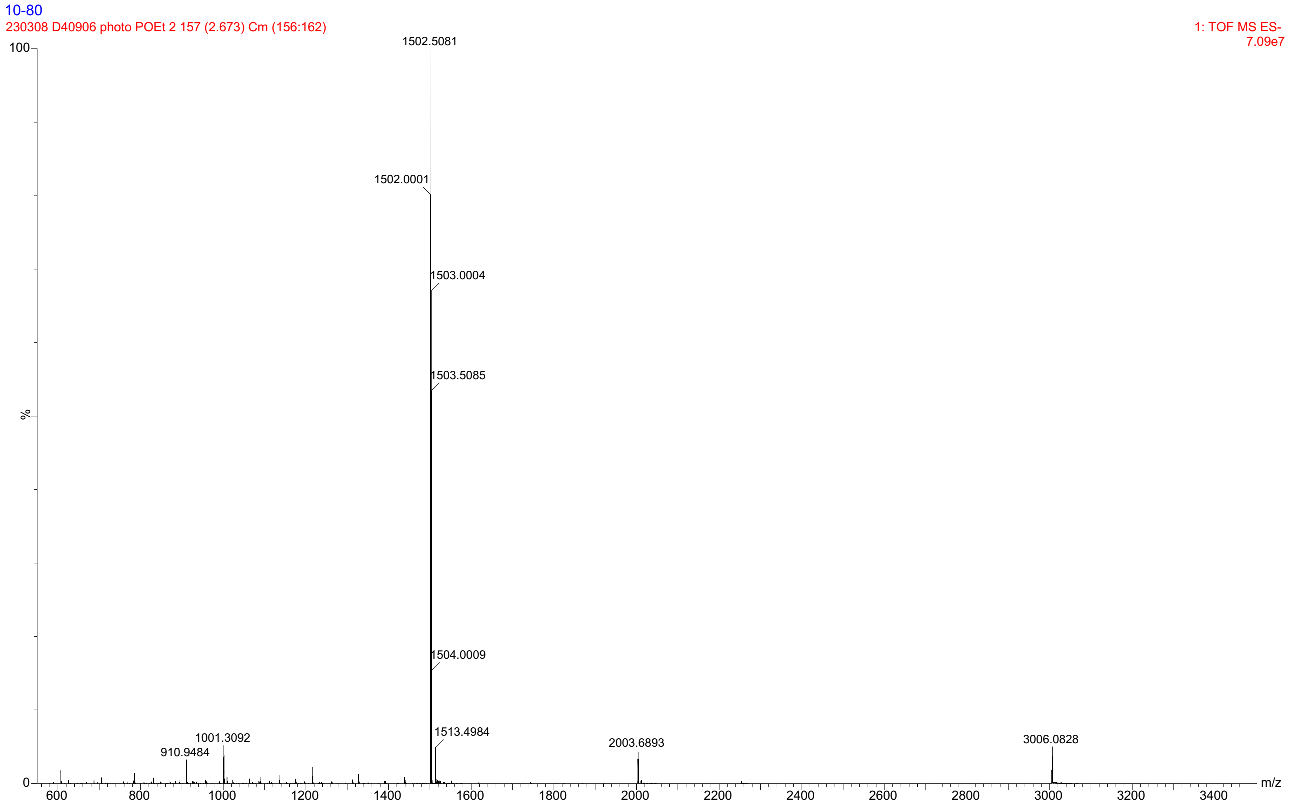

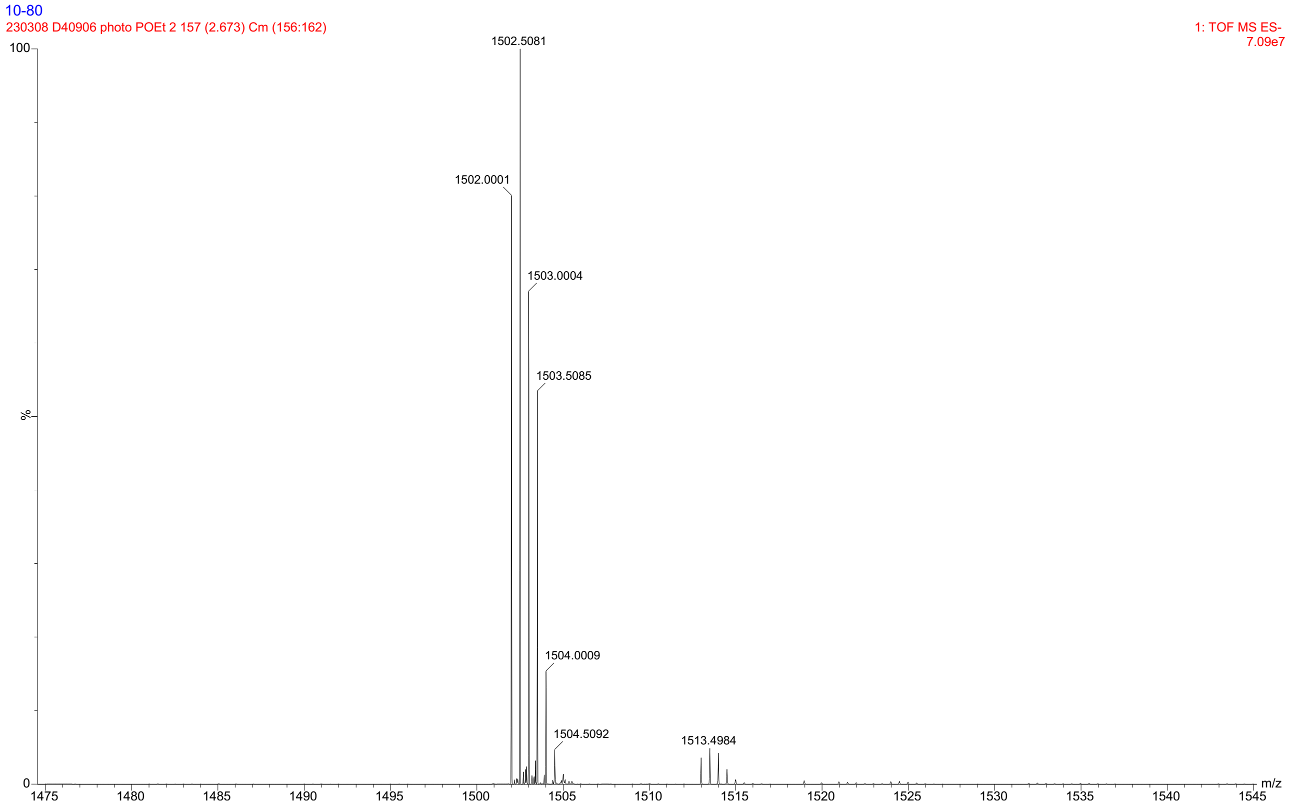
10-80
230308 D40906 photo POEt 2 157 (2.673) Cm (156:162)
1: TOF MS ES-
7.09e7
100
%
0
1502.5081
1502.0001
1503.0004
1503.5085
1504.0009
1504.5092
1513.4984
m/z
1475
1480
1485
1490
1495
1500
1505
1510
1515
1520
1525
1530
1535
1540
1545

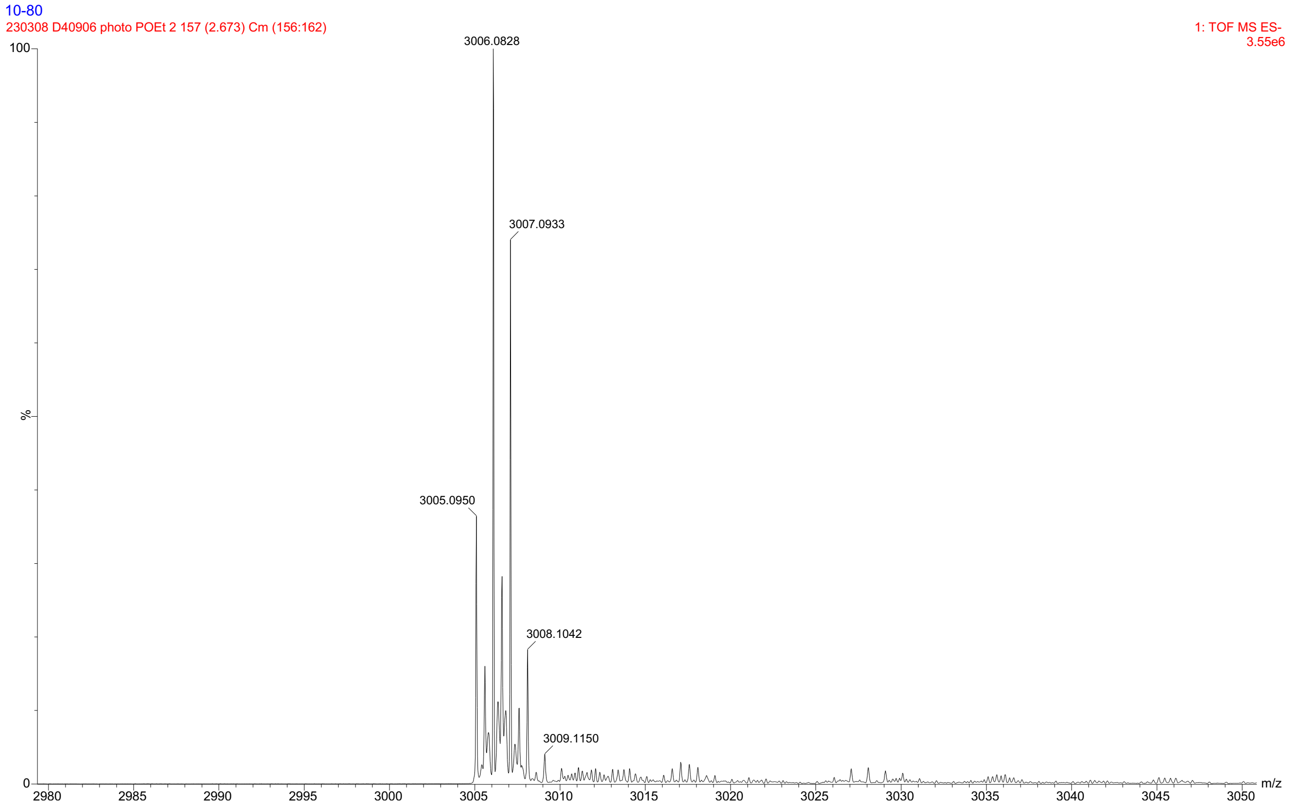
10-80
230308 D40906 photo POEt 2 157 (2.673) Cm (156:162)
1: TOF MS ES-
3.55e6
100
%
0
3006.0828
3007.0933
3005.0950
3008.1042
3009.1150
m/z
2980
2985
2990
2995
3000
3005
3010
3015
3020
3025
3030
3035
3040
3045
3050

## Oligo-8 TTTTTTTTT$_{MOP}$T

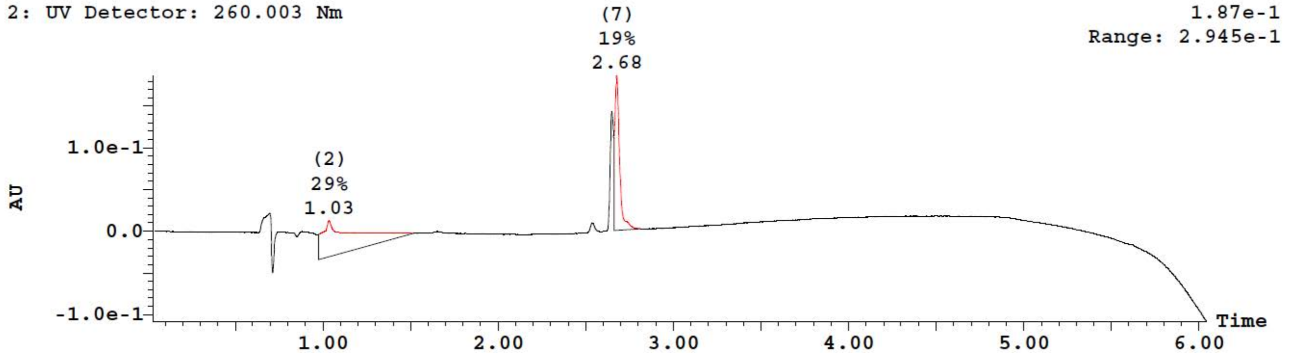


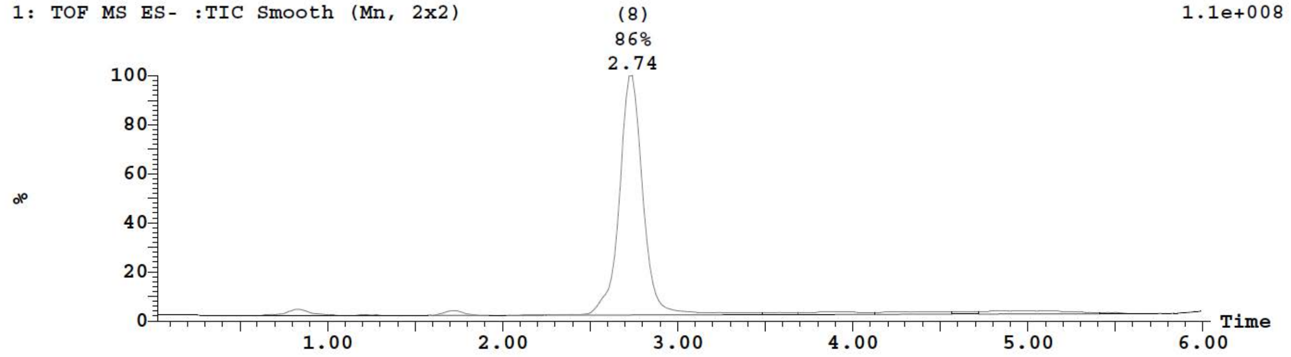


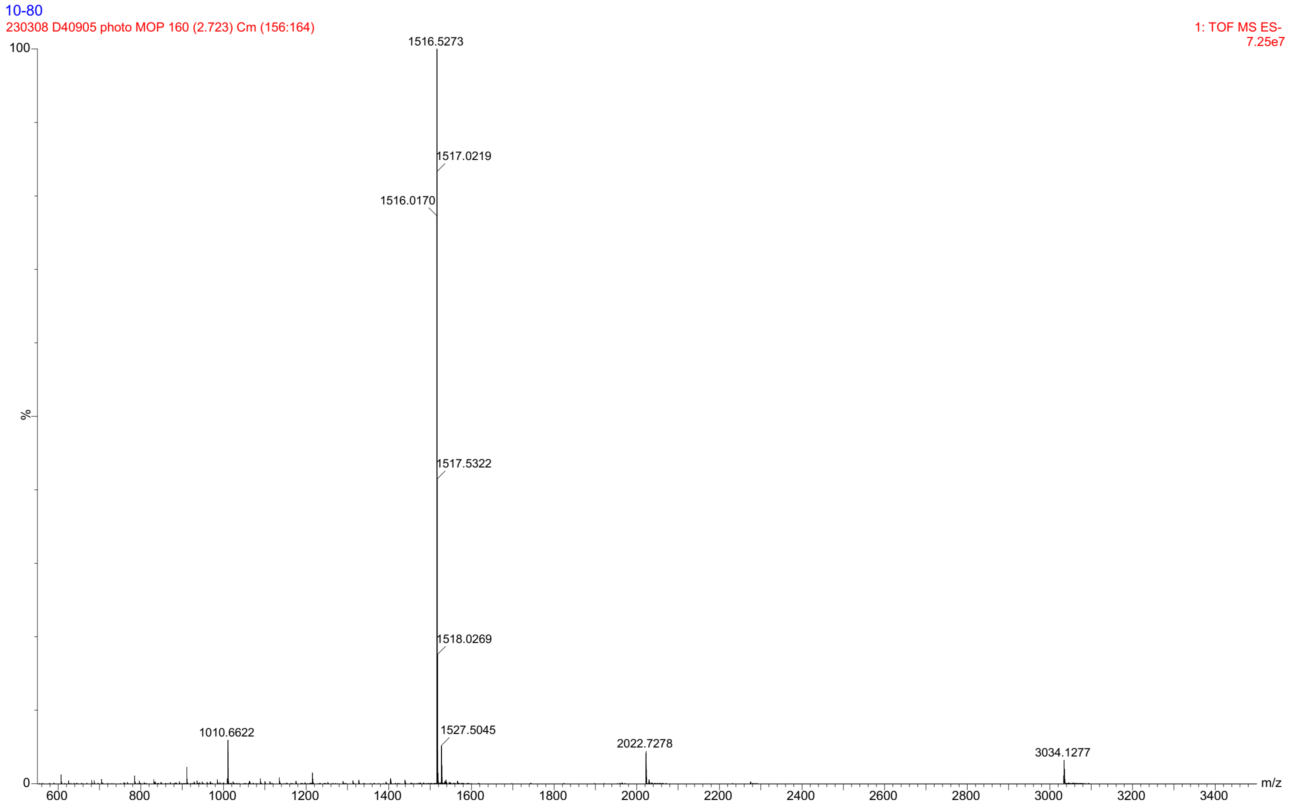

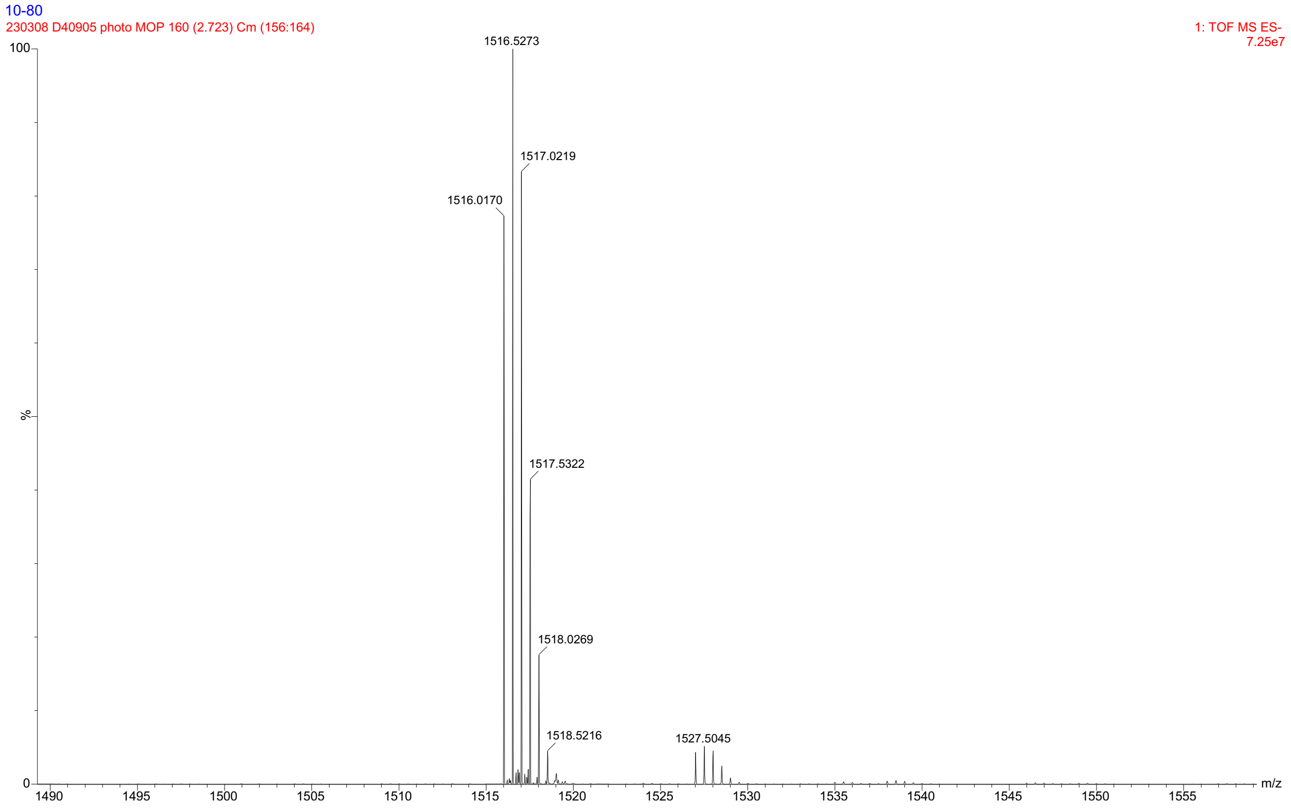
10-80
230308 D40905 photo MOP 160 (2.723) Cm (156:164)
1: TOF MS ES-
7.25e7
100
%
0
1516.5273
1517.0219
1516.0170
1517.5322
1518.0269
1518.5216
1527.5045
1490
1495
1500
1505
1510
1515
1520
1525
1530
1535
1540
1545
1550
1555
m/z

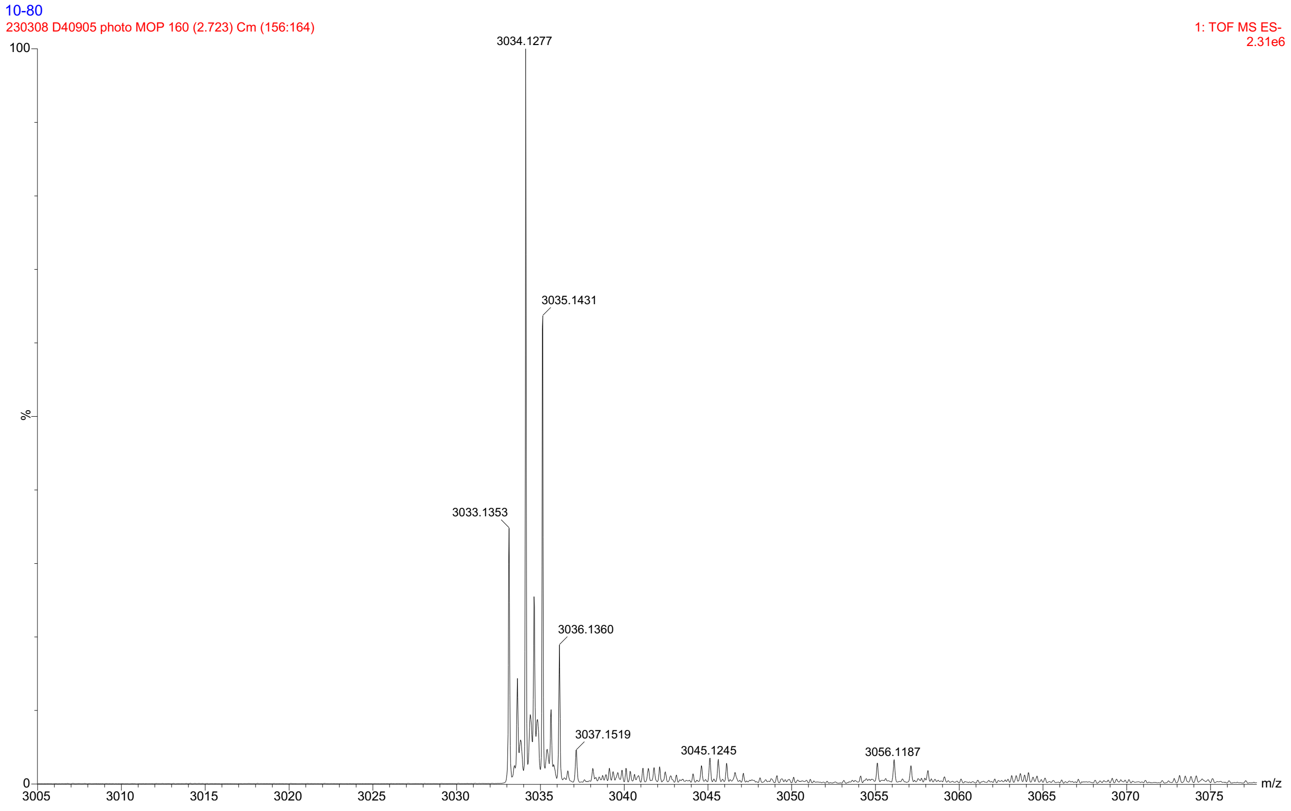
10-80
230308 D40905 photo MOP 160 (2.723) Cm (156:164)
1: TOF MS ES-
2.31e6
100
%
0
3034.1277
3035.1431
3033.1353
3036.1360
3037.1519
3045.1245
3056.1187
3005
3010
3015
3020
3025
3030
3035
3040
3045
3050
3055
3060
3065
3070
3075
m/z

| Synthesized oligonucleotides | MW: M-H (calcd) | MW: M-H (obsd) | crude yield (%) | retention time on LC (min) |
|---|---|---|---|---|
| **Oligo-1** ($R_P$)-TTTTTTTTT$_{Alk}$T | 3079.58 | 3079.14 | 35 | 3.09 |
| **Oligo-2** ($S_P$)-TTTTTTTTT$_{Alk}$T | 3079.58 | 3079.12 | 43 | 3.14 |
| **Oligo-3** ($R_P$)-TTTTT$_{Alk}$TTTTT | 3079.58 | 3079.12 | 47 | 2.80 |
| **Oligo-4** ($S_P$)-TTTTT$_{Alk}$TTTTT | 3079.58 | 3079.12 | 31 | 2.83 |
| **Oligo-5** ($S_P$,$S_P$)-T$_{Alk}$TTTTTTTT$_{Alk}$T | 3181.66 | 3181.18 | 32 | 3.66 |
| **Oligo-6** TTTTTTTTTT | 2977.50 | 2977.14 | 52 | 2.04 |
| **Oligo-7** TTTTTTTTT$_{OEt}$T | 3005.53 | 3005.10 | 25 | 2.63* |
| **Oligo-8** TTTTTTTTT$_{MOP}$T | 3033.56 | 3033.14 | 37 | 2.68* |

* LC diagram showed shouldered peaks in these compounds. MS spectra of each compound was the same, suggesting that they are diastereomers with a different stereo configuration around backbone phosphorous atom.

**Supplementary Fig. 4.** Summary of Yields and Retention Time of Oligo-1-8

## Comparizon between other charge-neutral backbonemodified Oligos (Oligo1,2 vs. Oligo-7,8)

Retention time (lipophilicity) of **Oligo-1** and **Oligo-2** was increased compared to the ethyl-triester- and MOP-modified oligos (**Oligo-7** and **8**), and the retention time gap due to the difference between the $S_P$ and $R_P$ configurations was also expanded. The influence of chirality on global hydrophilicity/hydrophobicity seemed to be increased due to its large size, which may also contribute to protein binding profiles and lead to eutomer/distomer differences as a pharmaceutical.

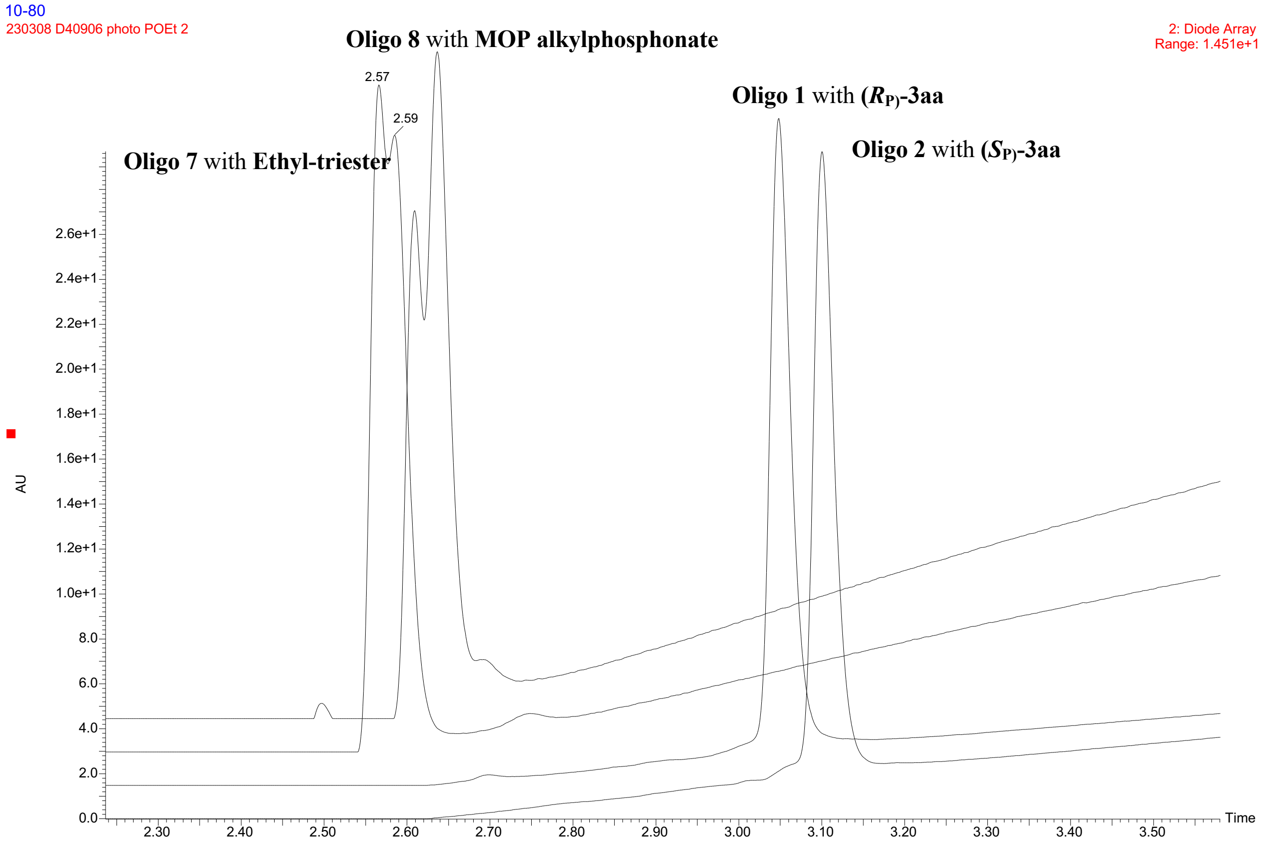

## 11. Detection of Alkylsulfonium

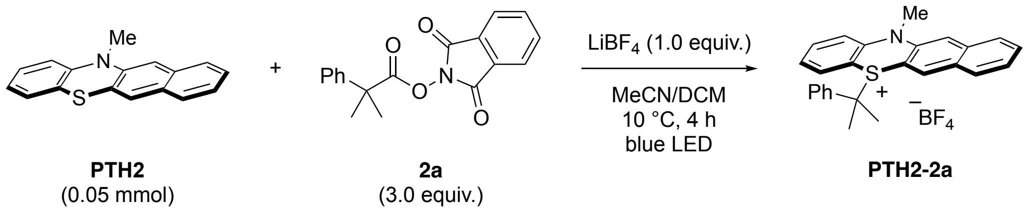


**Supplementary Fig. 5.** Detection of Alkylsulfonium

In a glovebox, to an oven-dried vial equipped with a stirring bar was added **PTH2** (13.2 mg, 0.05 mmol), redox-active ester **2a** (46.4 mg, 0.15 mmol) and $LiBF_4$ (4.7 mg, 0.15 mmol). Then, MeCN (300 μL) and DCM (200 μL) were added to the reaction mixture. After sealing the vial with a cap and removed from the glove box, the reaction was stirred and irradiated with a 34W blue LED with a cooling UC reactor to keep the temperature at 10 °C (Supplementary Fig. 2). After 4 h, the reaction solution was directly used for MS analysis (HRMS–ESI). **PTH2-2a**: **HRMS–ESI** (m/z): $[M–BF_4]^+$ calcd for $C_{26}H_{24}NS$, 382.1624; found, 382.1614.

## 12. Effects of Additives on Conversion of Redox-Active Esters

In a glovebox, to an oven-dried vial with a stirring bar was added **PTH2** (13.2 mg, 0.05 mmol), phosphite **1a** (42.9 mg, 0.05 mmol), redox-active ester **2a** (46.4 mg, 0.15 mmol), $LiBF_4$ (4.7 mg, 0.15 mmol) and thymine (6.3 mg, 0.05 mmol). Then, MeCN (300 μL) and DCM (200 μL) were added to the reaction mixture. After sealing the vial with parafilm, the reaction mixture was stirred and irradiated with a 34W blue LED with a cooling UC reactor to keep the temperature at 10 °C (Supplementary Fig. 2). After 4 h, the solvents were removed under reduced pressure and the residue was analyzed by $^1H$ NMR. The increase in conversion of **2a** to 28% was observed with the addition of thymine compared to without thymine (11%).

## 13. Comparison with Other Carbocation Methods

**Comparison with Aggarwal's Method**. Tertiary alkylation of phosphite **1a-1** was conducted with reference to the reported procedure.[14] In a glovebox, to an oven-dried vial with a stirring bar was added photoredox catalyst 4CzIPN (0.79 mg, 0.001 mmol), phosphite **1a-1** (42.9 mg, 0.05 mmol) and redox-active ester **2a** (46.4 mg, 0.15 mmol). Then, MeCN (500 μL) and TFA (5.7 μL, 0.075 mmol) were added to the reaction mixture. After sealing the vial with parafilm, the reaction mixture was stirred and irradiated with a 34W blue LED (3 cm away) with a cooling fan to keep the temperature around 40 °C. After 2 h, the solvents were removed under reduced pressure and the residue was analyzed by 1H NMR. However, the desired product **3aa** was not obtained, and the decomposition products of **1a-1** were observed.

**Comparison with methods using Lewis acid.** Tertiary alkylation of phosphite **1a-1** was conducted with reference to the reported procedure.[15,16] In a glovebox, to an oven-dried vial with a stirring bar was added phosphite **1a-1** (42.9 mg, 0.05 mmol) and 2-phenyl-2-propanol (**5a**) (20.4 mg, 0.15 mmol). Then, toluene (500 μL) and Lewis acid TMSOTf (1.81 μL, 0.01 mmol) or $ZnI_2$ (23.9 mg, 0.075

mmol) were added to the reaction mixture. After sealing the vial with parafilm, the reaction mixture was stirred at 110 °C. After 15−24 h, the solvents was removed under reduced pressure and the residue was analyzed by 1H NMR. However, the desired product **3aa** was not obtained, giving a complex mixture.

## 14. The Effects of Chiral Leaving Groups

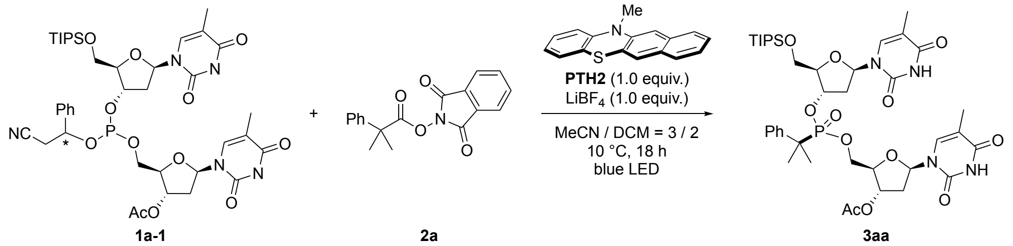


| Entry | Phosphite substrate | | Yield (%) of **3aa** |
|---|---|---|---|
| 1 | 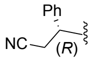 | d.r. = 1.1:1 | 71%, d.r. = 1.2:1 |
| 2 | 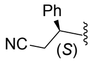 | d.r. = 1.1:1 | 61%, d.r. = 1.1:1 |
| 3 | 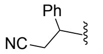 racemic | d.r. = 1.2:1.1:1.1:1 └(R)┘└(S)┘ | 74%, d.r. = 1.2:1 |

**Supplementary Fig. 6.** The Effects of Chiral Leaving Groups

Chiral alcohols were prepared from chiral styrene oxide.[17] Phosphite (*R*)/(*S*)-**1a-1** having chiral leaving groups were syshesized by the above mentioned procedure (Supplementary Fig. 1). In a glovebox, to an oven-dried vial with a stirring bar was added **PTH2** (13.2 mg, 0.05 mmol), phosphite (*R*)-**1a-1** or (*S*)-**1a-1** (42.9 mg, 0.05 mmol), redox-active ester **2a** (46.4 mg, 0.15 mmol) and LiBF4 (4.7 mg, 0.05 mmol). Then, MeCN (300 µL) and DCM (200 µL) were added to the reaction mixture. After sealing the vial with parafilm, the reaction mixture was stirred and irradiated with a 34W blue LED with a cooling UC reactor to keep the temperature at 10 °C (Supplementary Fig. 2). After 18 h, the solvents were removed under reduced pressure and the yield was determined by $^1$H-NMR analysis. The effect of the chiral leaving group on this reaction was investigated, but no significant difference in the yield and the ratio of diastereomers of the alkylated product **3aa** was observed.

## 15. Examination of Catalytic Reactions

When simple phosphite **1g** was used as a nucleophile, oxidative alkylation proceeded catalytically with high efficiency (Supplementary Fig. 7A). On the other hand, when H-phosphonate **1h** was used as a nucleophile instead of phosphite, no alkylated product was obtained (Supplementary Fig. 7B).

**Procedure.** In a glovebox, to an oven-dried vial with a stirring bar was added **PTH1** (3.3 mg, 0.01 mmol), trihexyl phosphite **1g** (74.3 µL, 0.2 mmol) or dibutyl phosphite **1h** (39.2 µL, 0.2 mmol), redox-active ester **2a** (92.8 mg, 0.3 mmol), $LiBF_4$ (0.9 mg, 0.01 mmol) and DCM (600 µL). After sealing the vial with parafilm, the reaction mixture was stirred and irradiated with a 34W blue LED with a cooling fan to keep the temperature around 40 °C. After 16 h, the solvents were removed under reduced pressure and the residue was analyzed by $^1$H NMR.

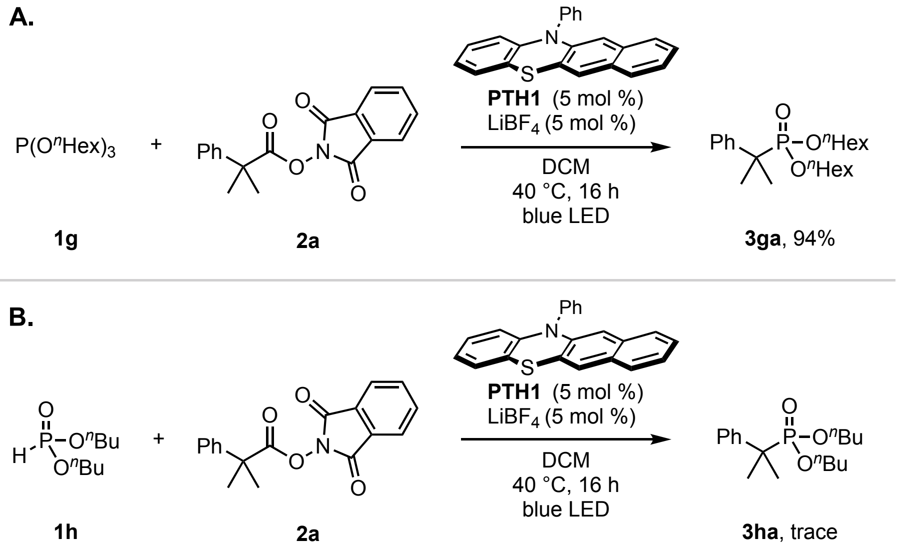


**Supplementary Fig. 7.** Examination of Catalytic Reactions

## 16. Examination of the Addition of Trapping Reagent for Carbocation

PTH-catalytic reactions were investigated by adding a stoichiometric amount of sulfilimine, used in the stabilized cation pool method,[18] as a trapping reagent for the carbocation, but the yield was not improved (Supplementary Fig. 8).

**Procedure.** In a glovebox, to an oven-dried vial with a stirring bar was added **PTH1** (1.63 mg, 0.005 mmol), phosphite **1a-1** (42.9 mg, 0.05 mmol), redox-active ester **2a** (46.4 mg, 0.15 mmol) or **2k** (37.1 mg, 0.15 mmol), $LiBF_4$ (0.47 mg, 0.005 mmol) and sulfilimine (17.8 mg, 0.05 mmol). Then, MeCN (300 µL) and DCM (200 µL) were added to the reaction mixture. After sealing the vial with parafilm, the reaction mixture was stirred and irradiated with a 34W blue LED with a cooling UC reactor to keep the temperature at 10 °C (Supplementary Fig. 2). After 18 h, the solvents were removed under reduced pressure and the residue was analyzed by $^1$H NMR.

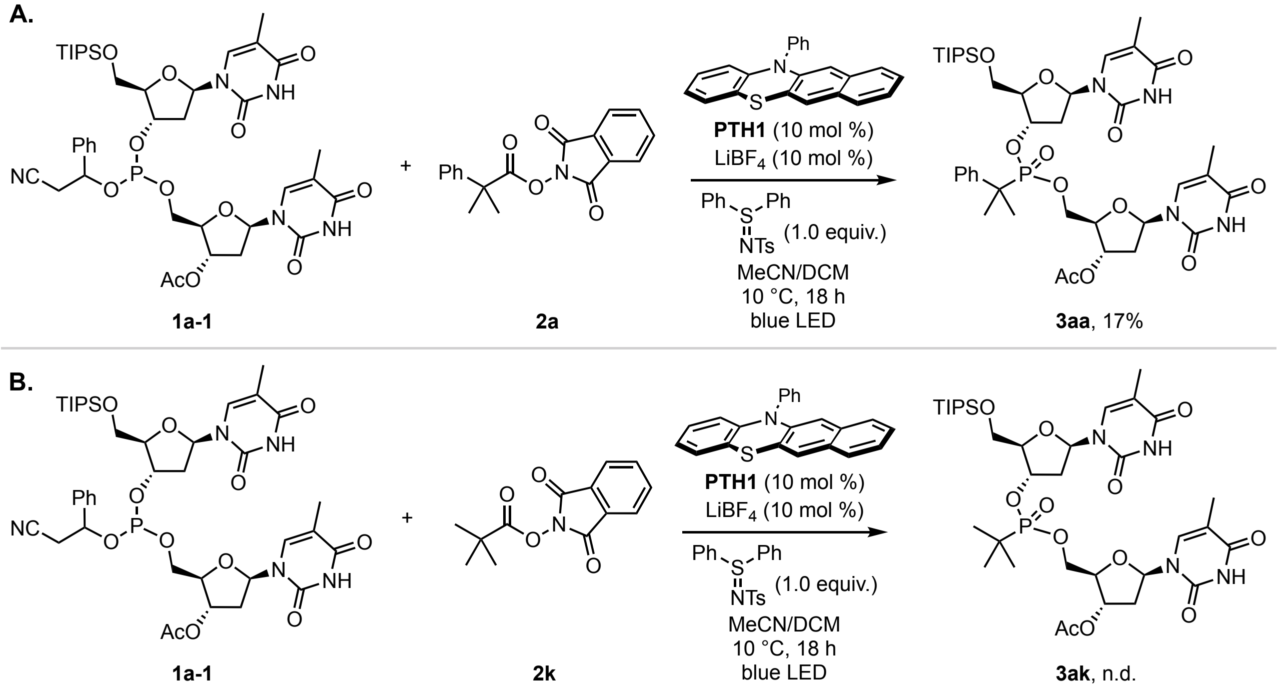


**Supplementary Fig. 8.** Examination of the Addition of Trapping Reagent for Carbocation

## 17. Unsuccessful Substrates

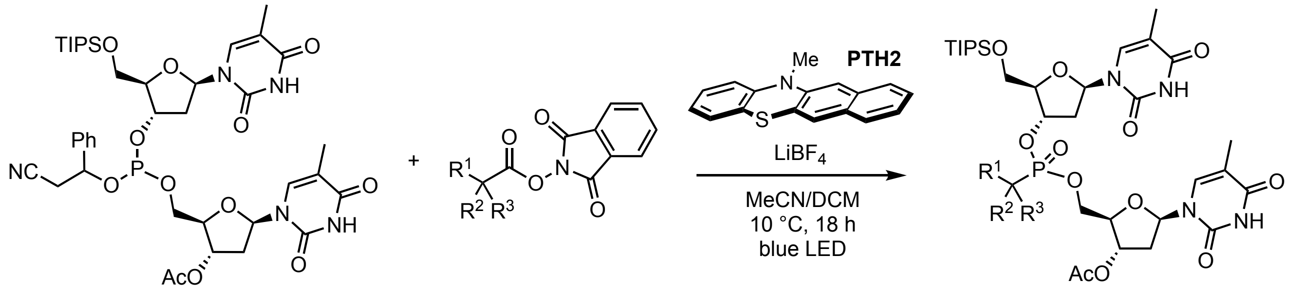


**1a-1** **2** **3**

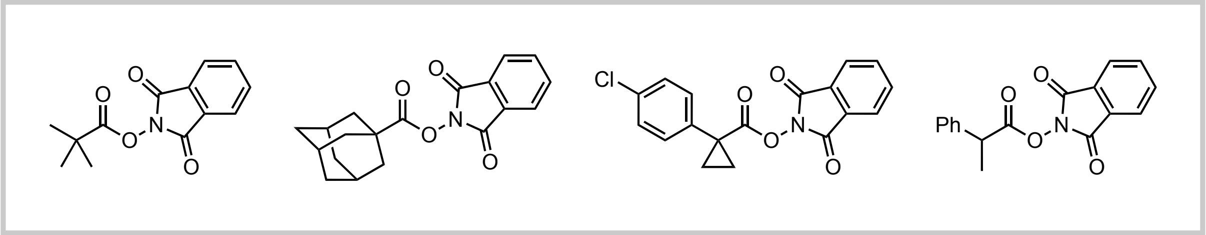

**Supplementary Fig. 9.** Unsuccessful Substrates

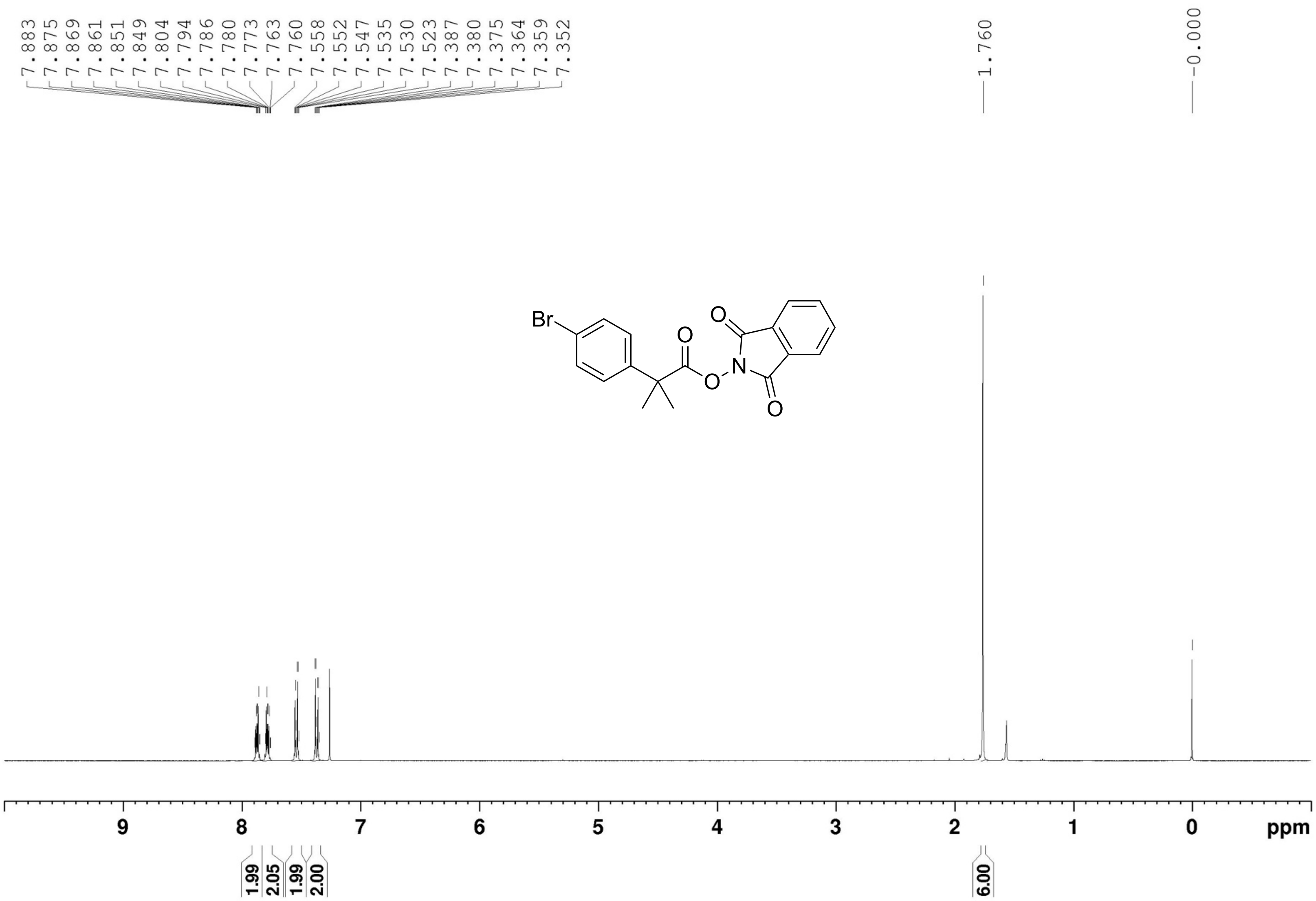


$^{1}$H NMR (400 MHz, $CDCl_3$) spectrum of **2b**

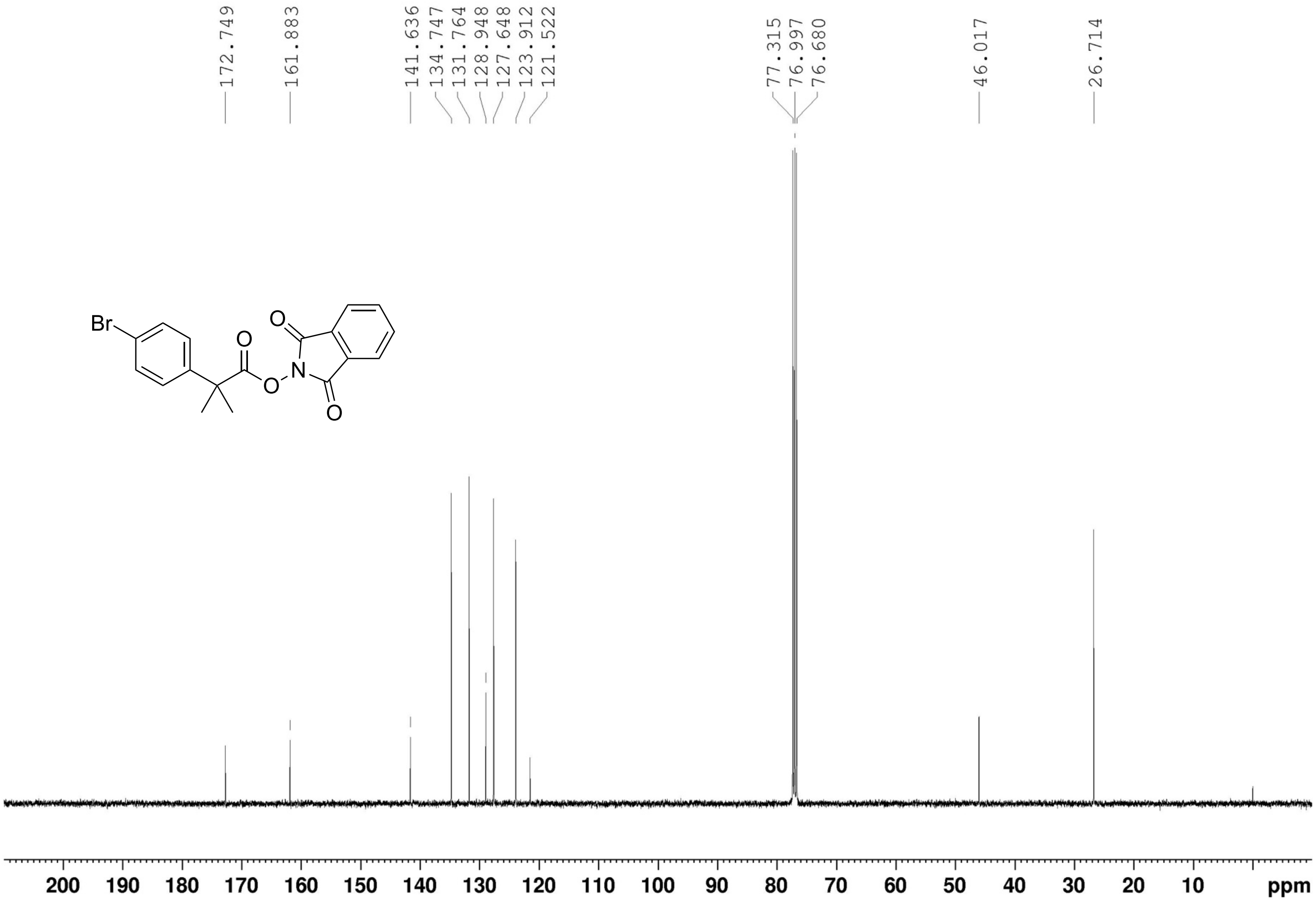


$^{13}$C NMR (100.6 MHz, $CDCl_3$) spectrum of **2b**

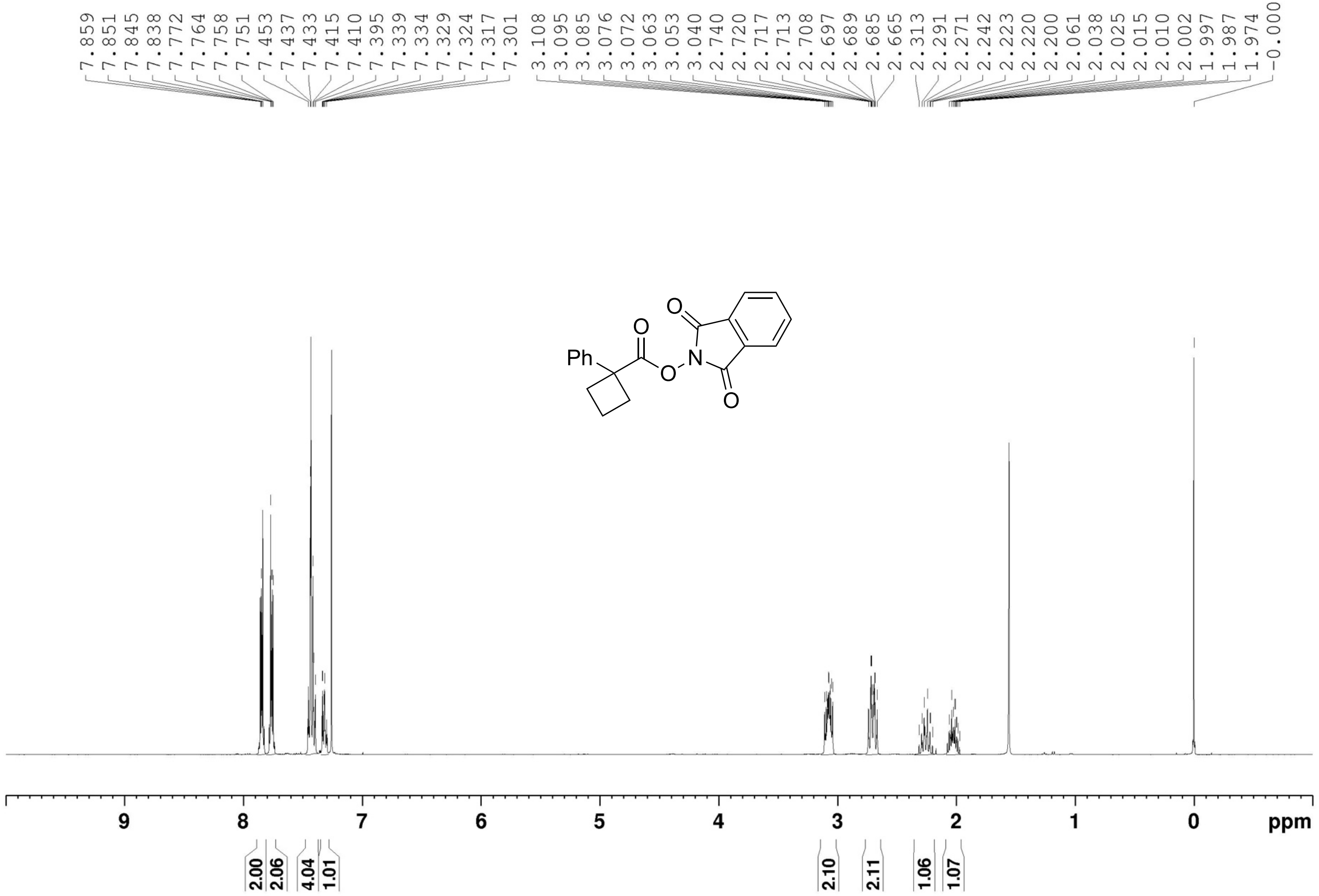


$^{1}$H NMR (400 MHz, $CDCl_3$) spectrum of **2c**

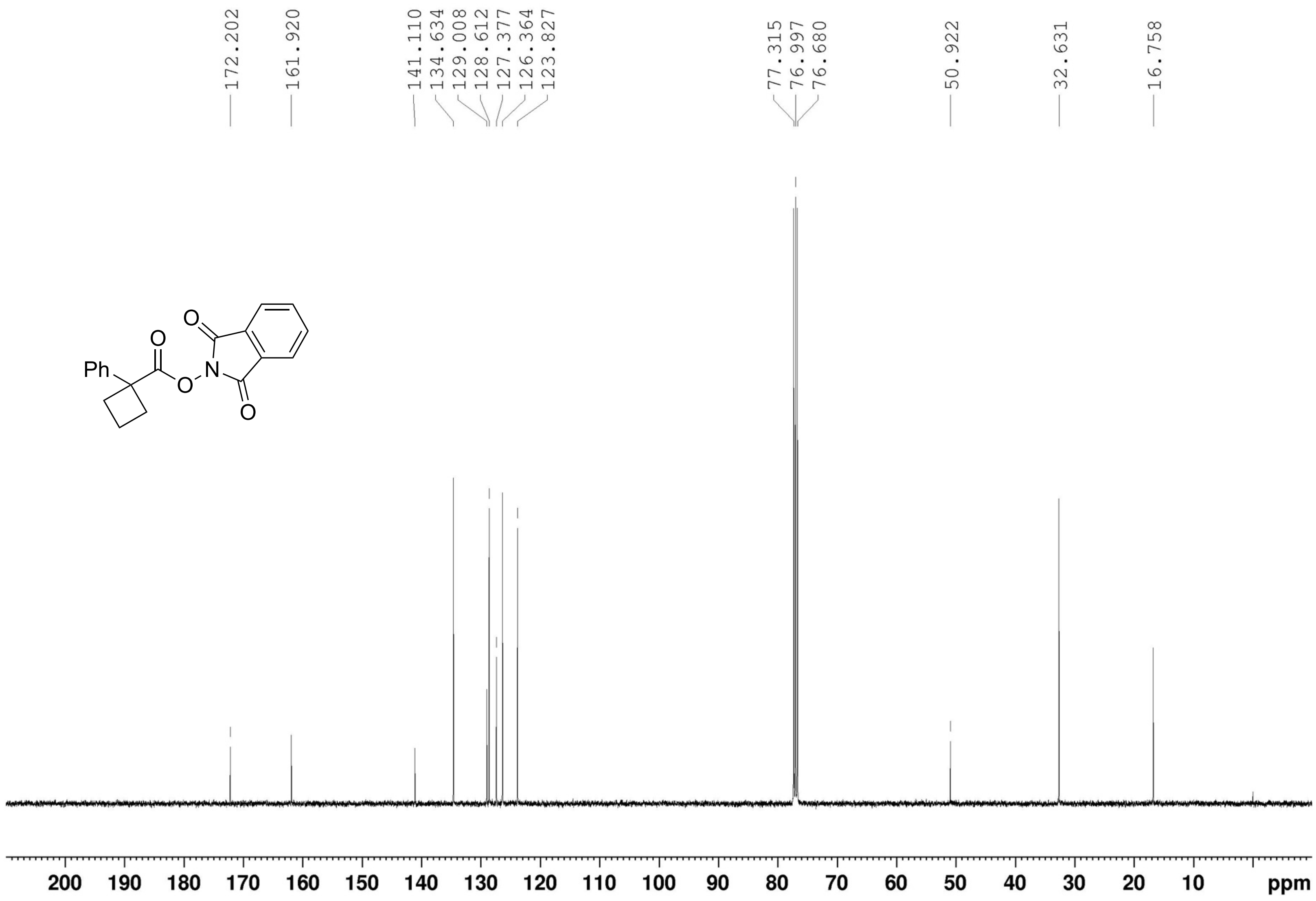


$^{13}$C NMR (100.6 MHz, $CDCl_3$) spectrum of **2c**

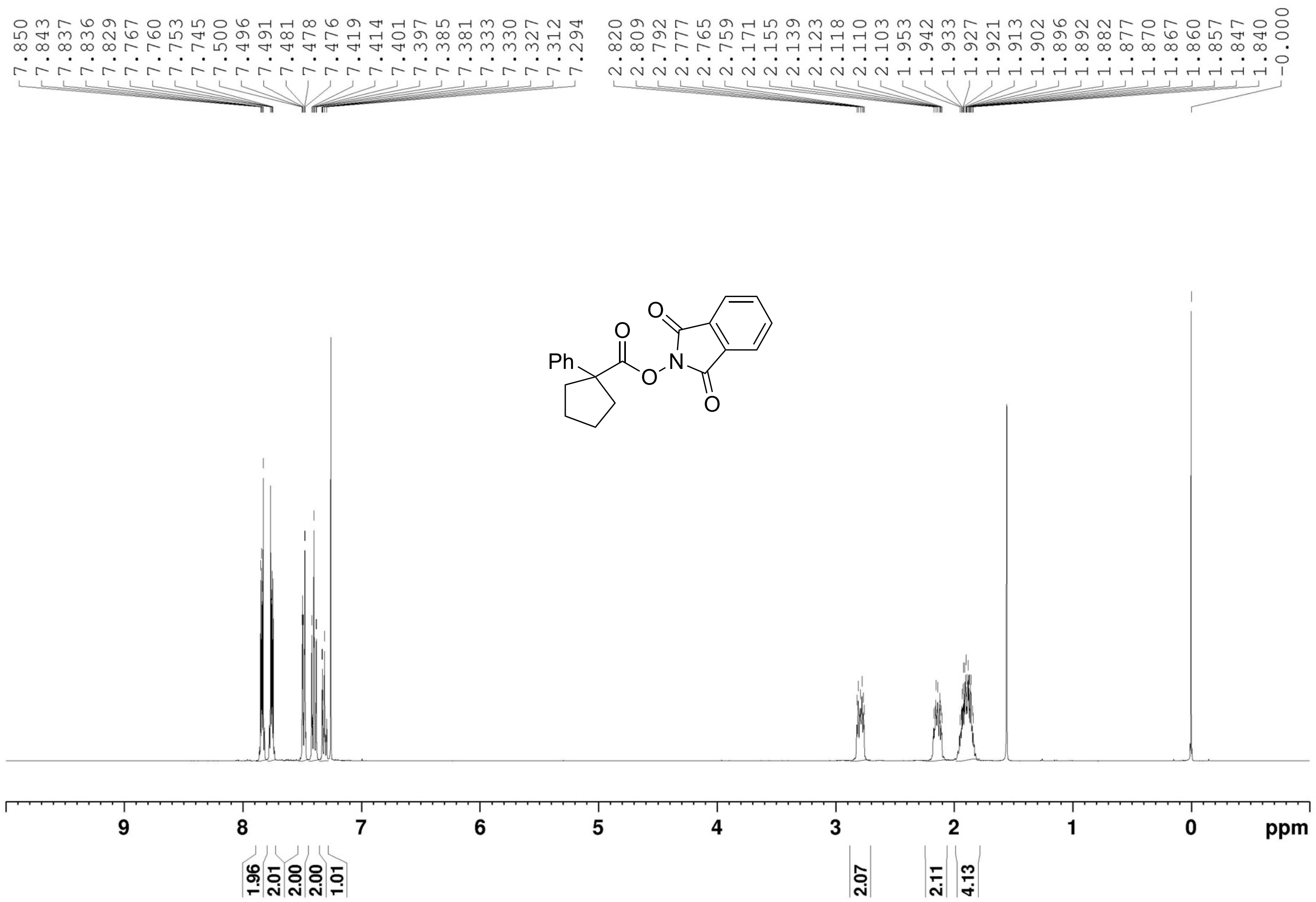


$^{1}$H NMR (400 MHz, $CDCl_3$) spectrum of **2d**

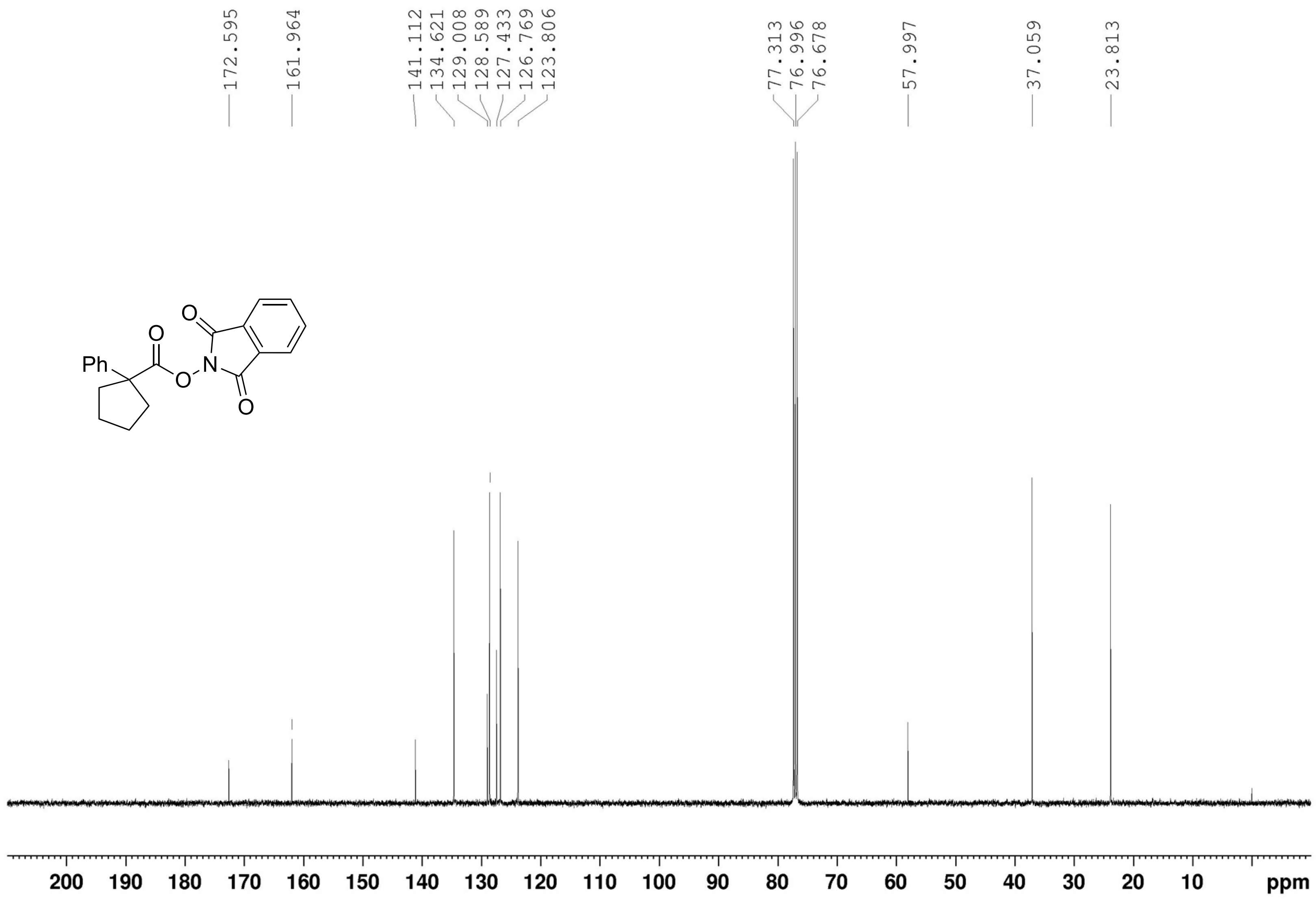


$^{13}C$ NMR (100.6 MHz, $CDCl_3$) spectrum of **2d**

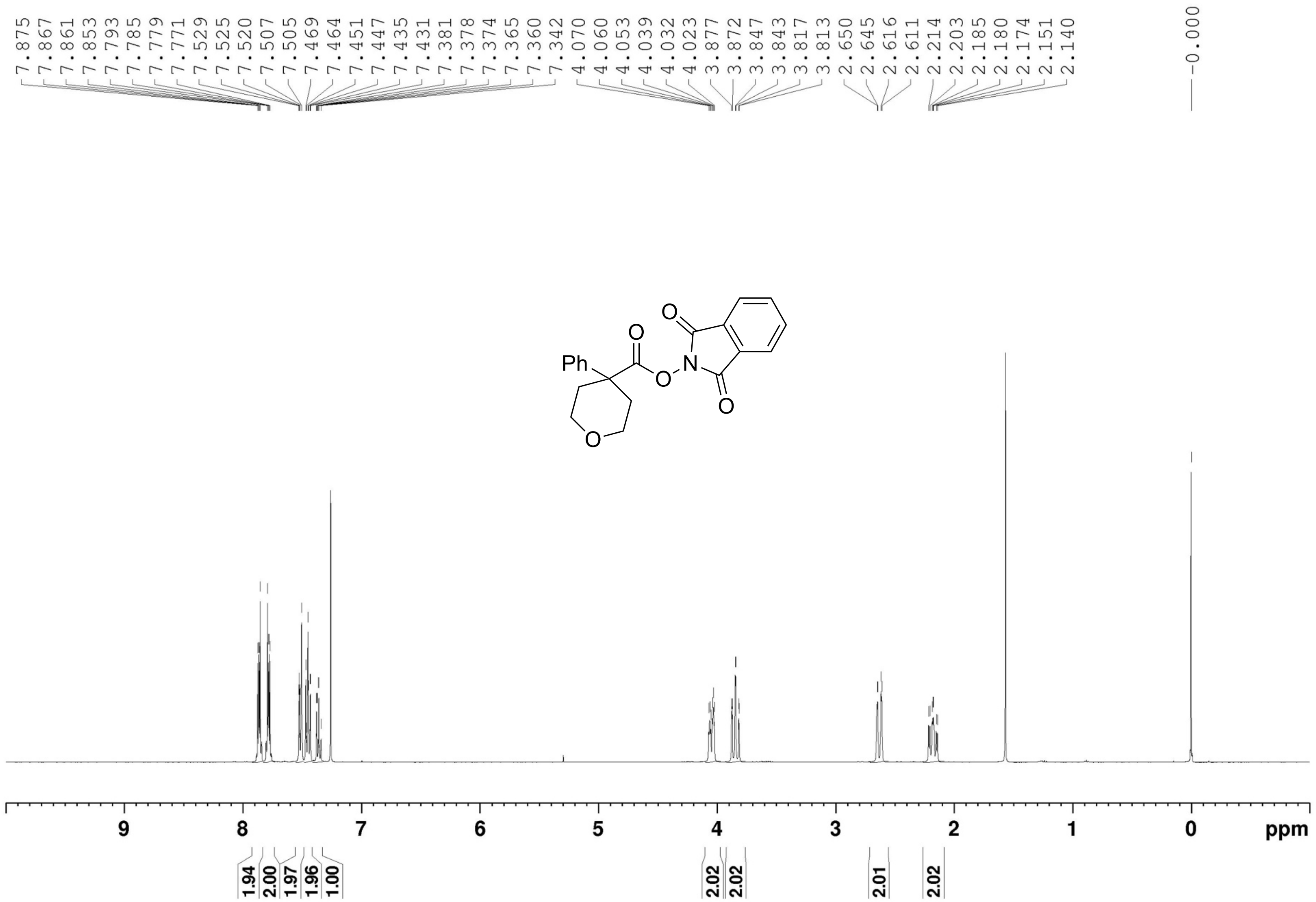


$^1$H NMR (400 MHz, $CDCl_3$) spectrum of **2e**

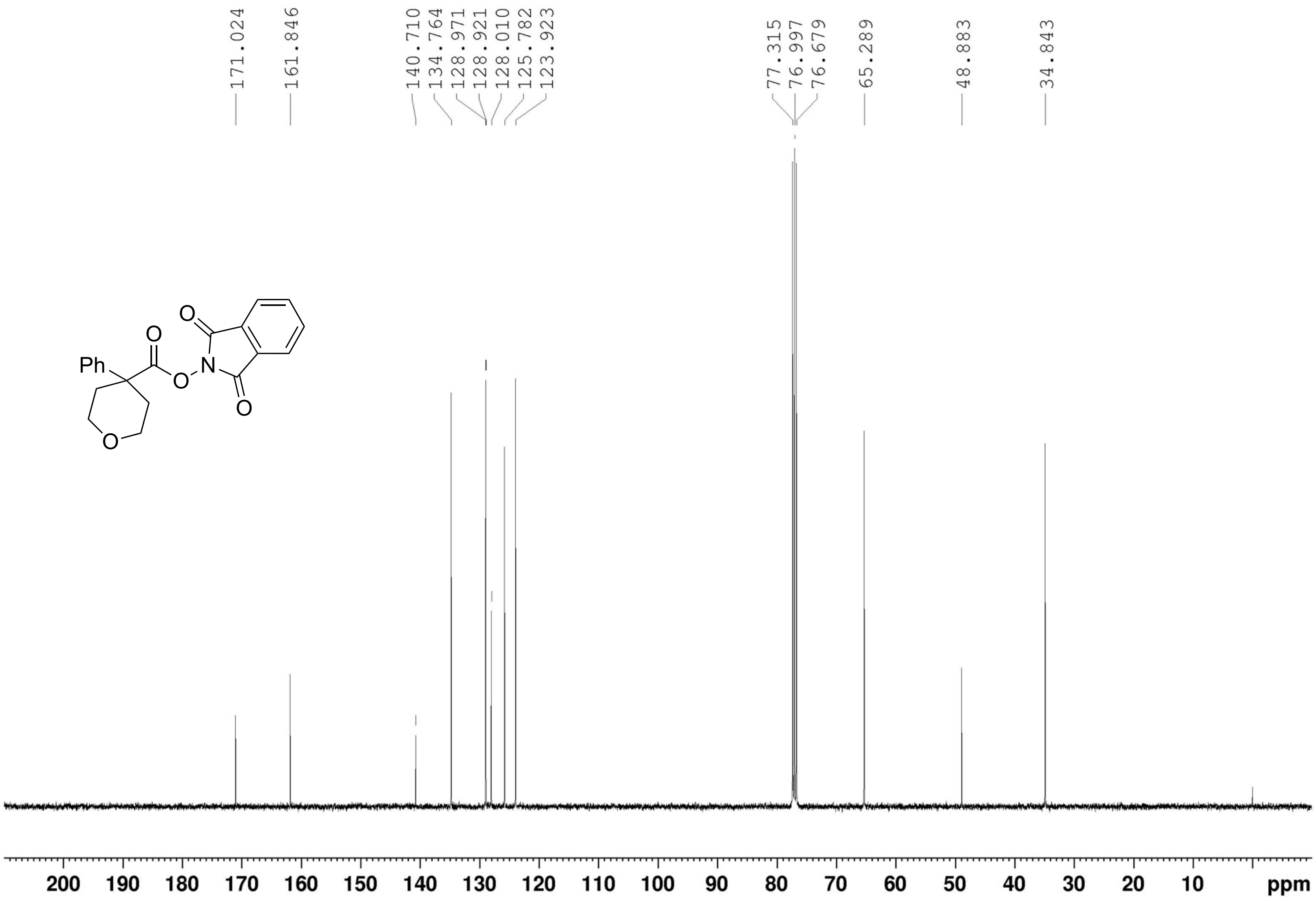


$^{13}$C NMR (100.6 MHz, $CDCl_3$) spectrum of **2e**

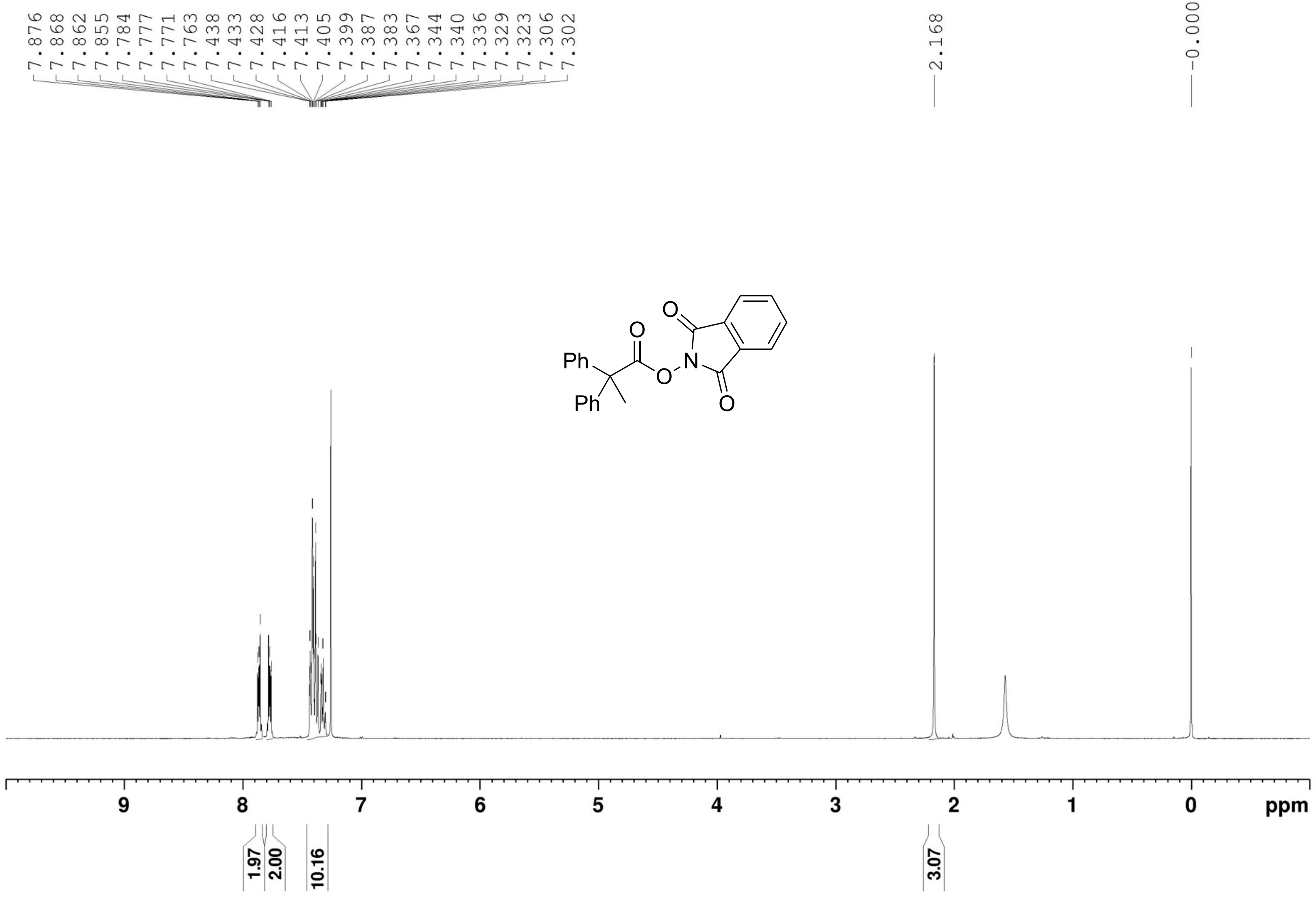


$^{1}$H NMR (400 MHz, $CDCl_3$) spectrum of **2g**

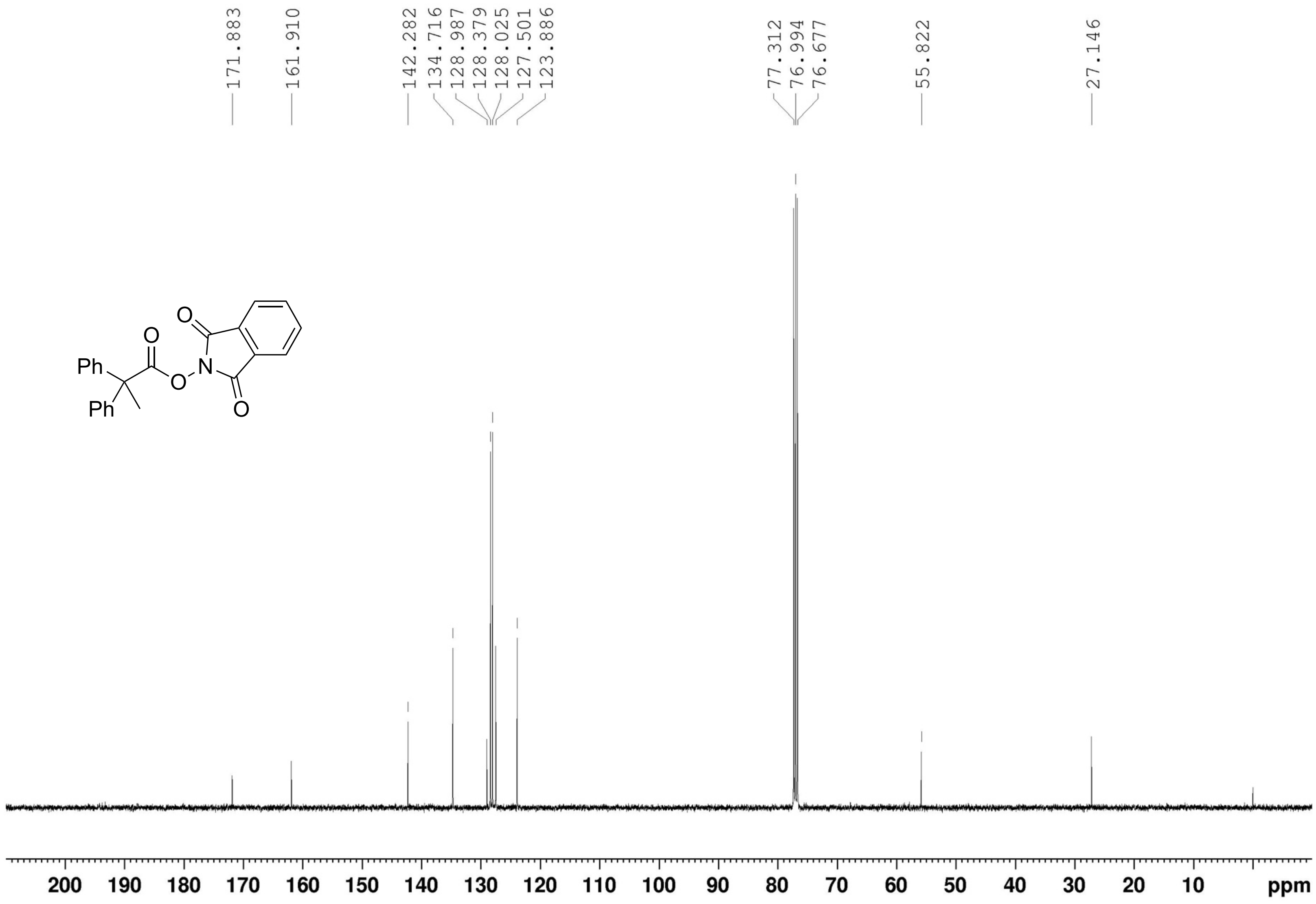


$^{13}$C NMR (100.6 MHz, $CDCl_3$) spectrum of **2g**

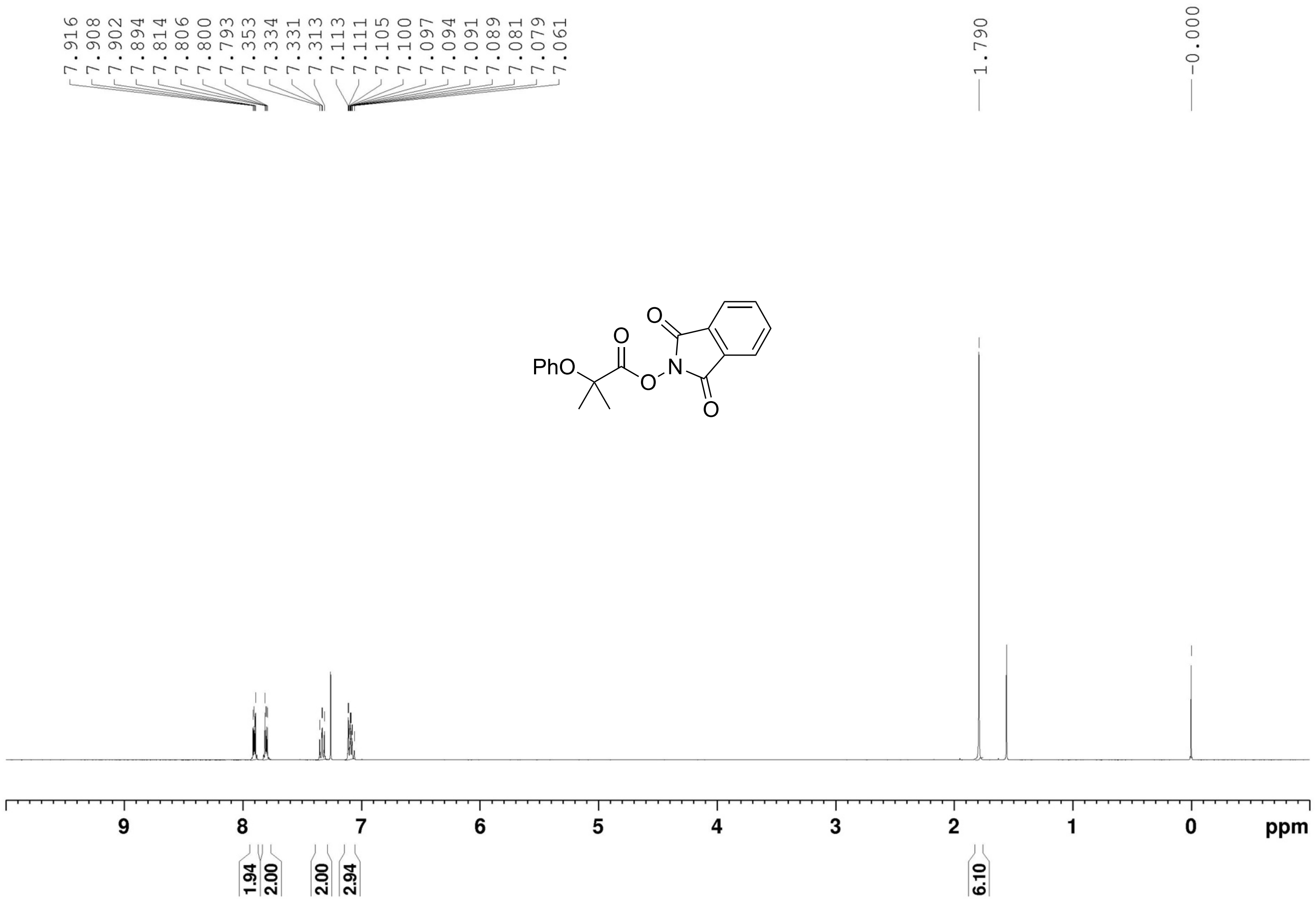


$^{1}$H NMR (400 MHz, $CDCl_3$) spectrum of **2j**

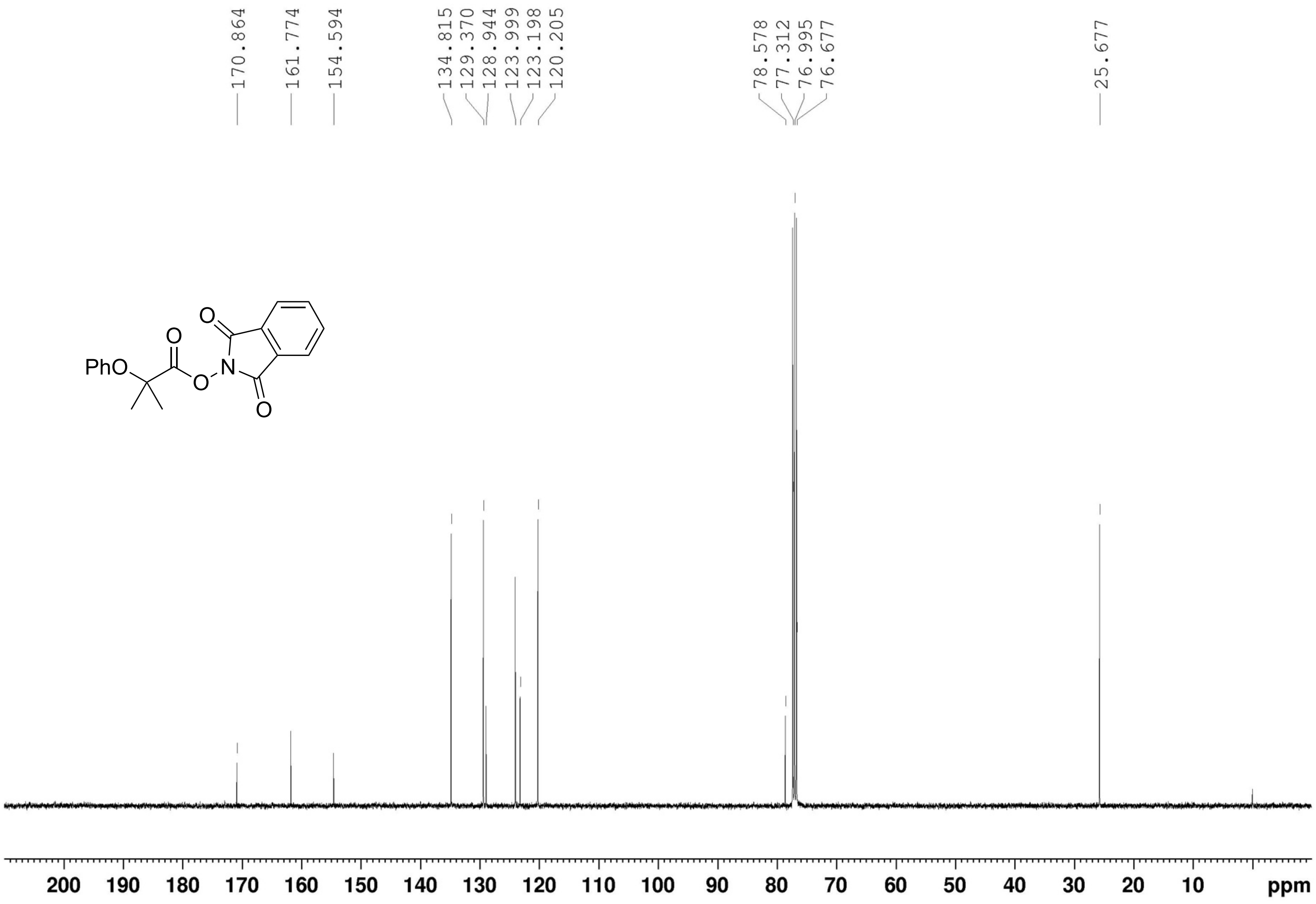


$^{13}C$ NMR (100.6 MHz, $CDCl_3$) spectrum of **2j**

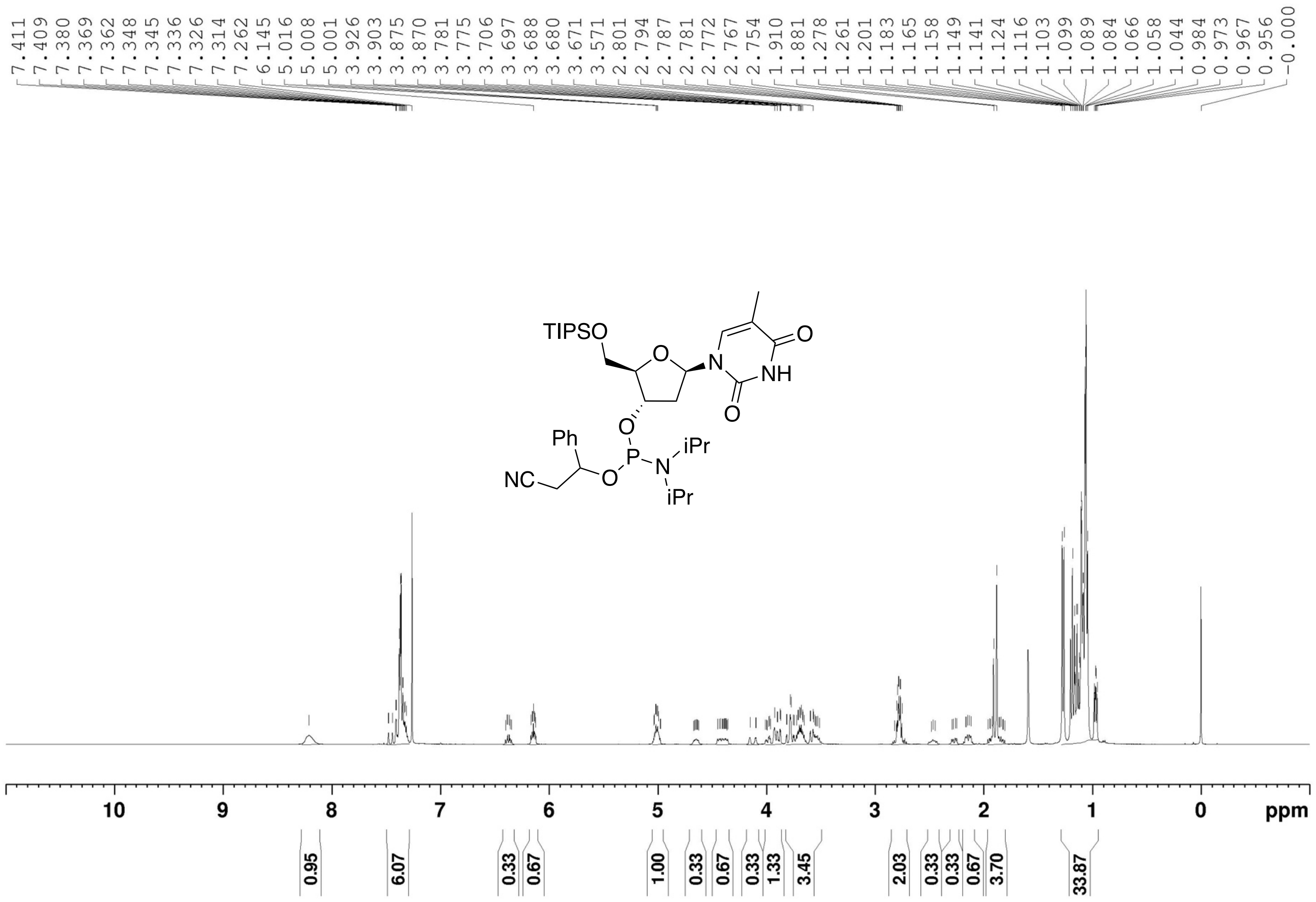

$^{1}H$ NMR (400 MHz, $CDCl_3$) spectrum of **S3-1**

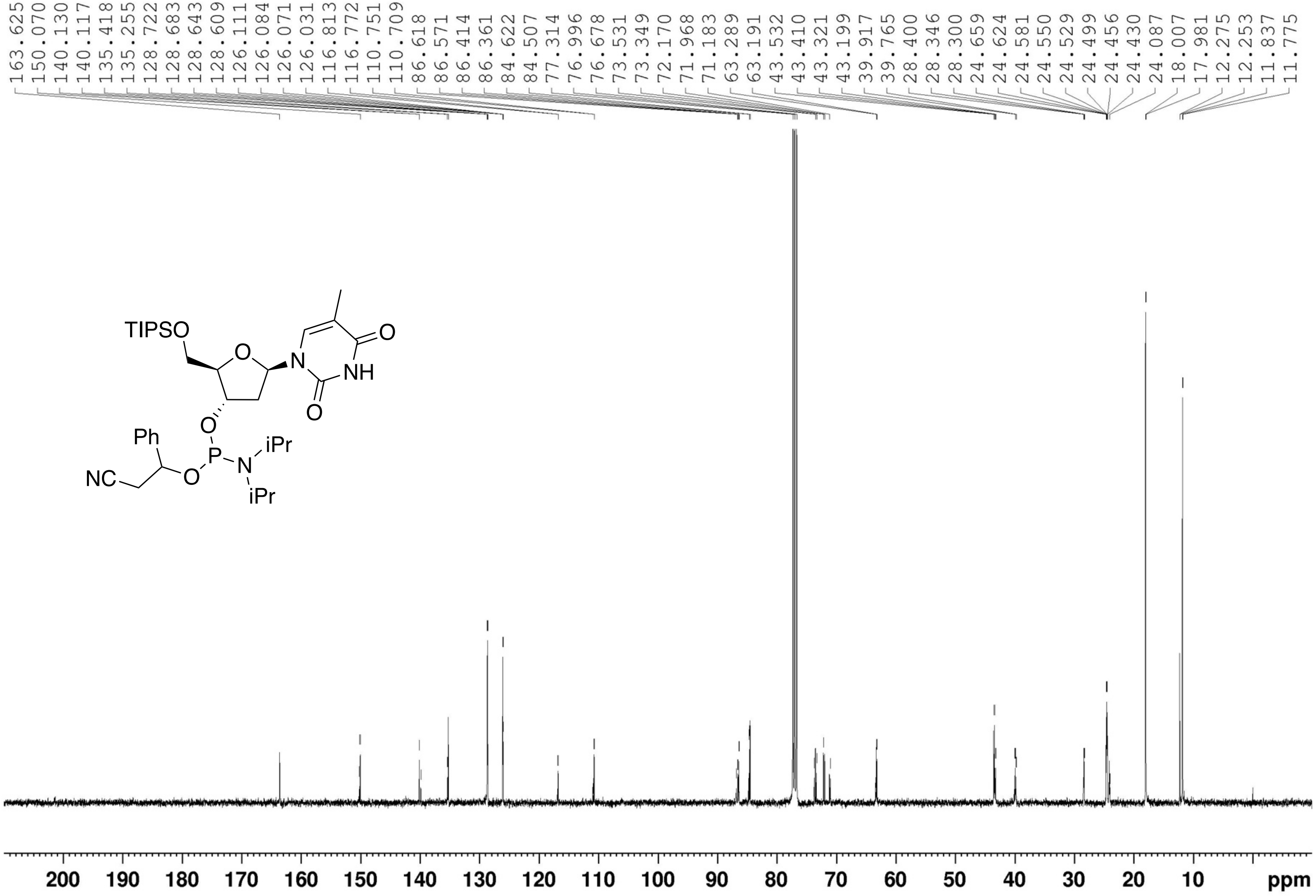


$^{13}$C NMR (100.6 MHz, CDCl$_3$) spectrum of **S3-1**

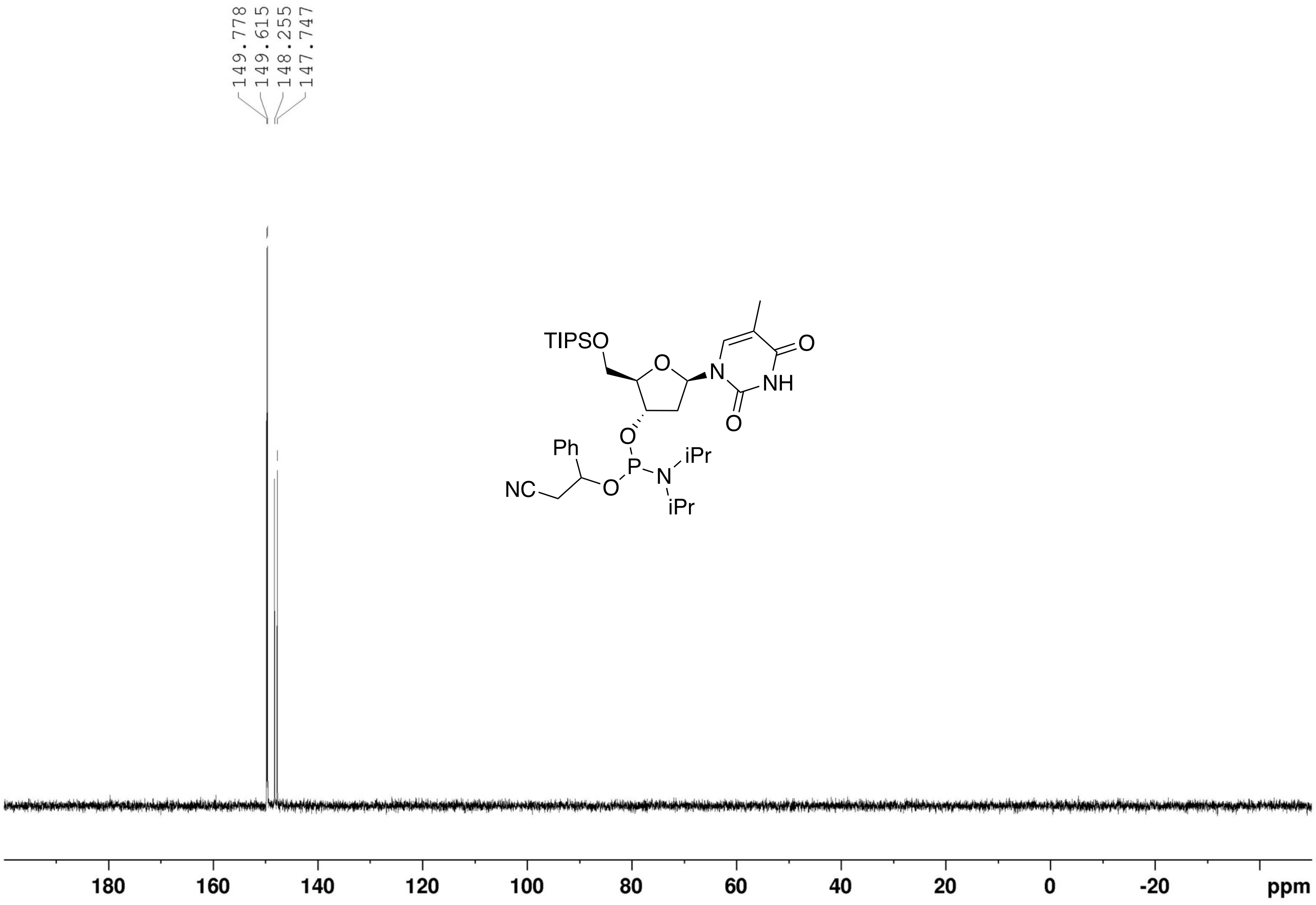


$^{31}$P NMR (162 MHz, $CDCl_3$) spectrum of **S3-1**

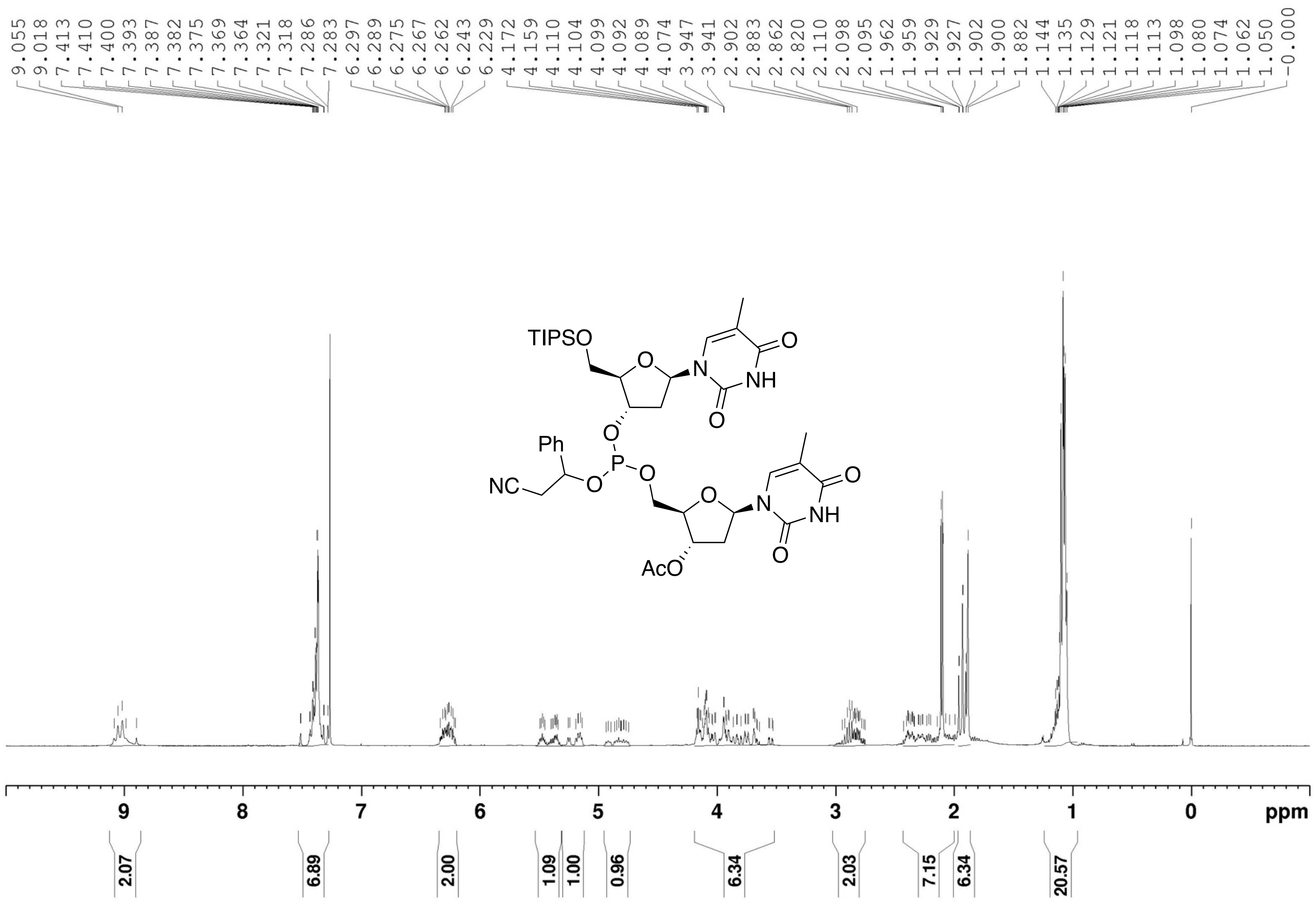


$^{1}$H NMR (400 MHz, $CDCl_3$) spectrum of **1a-1**

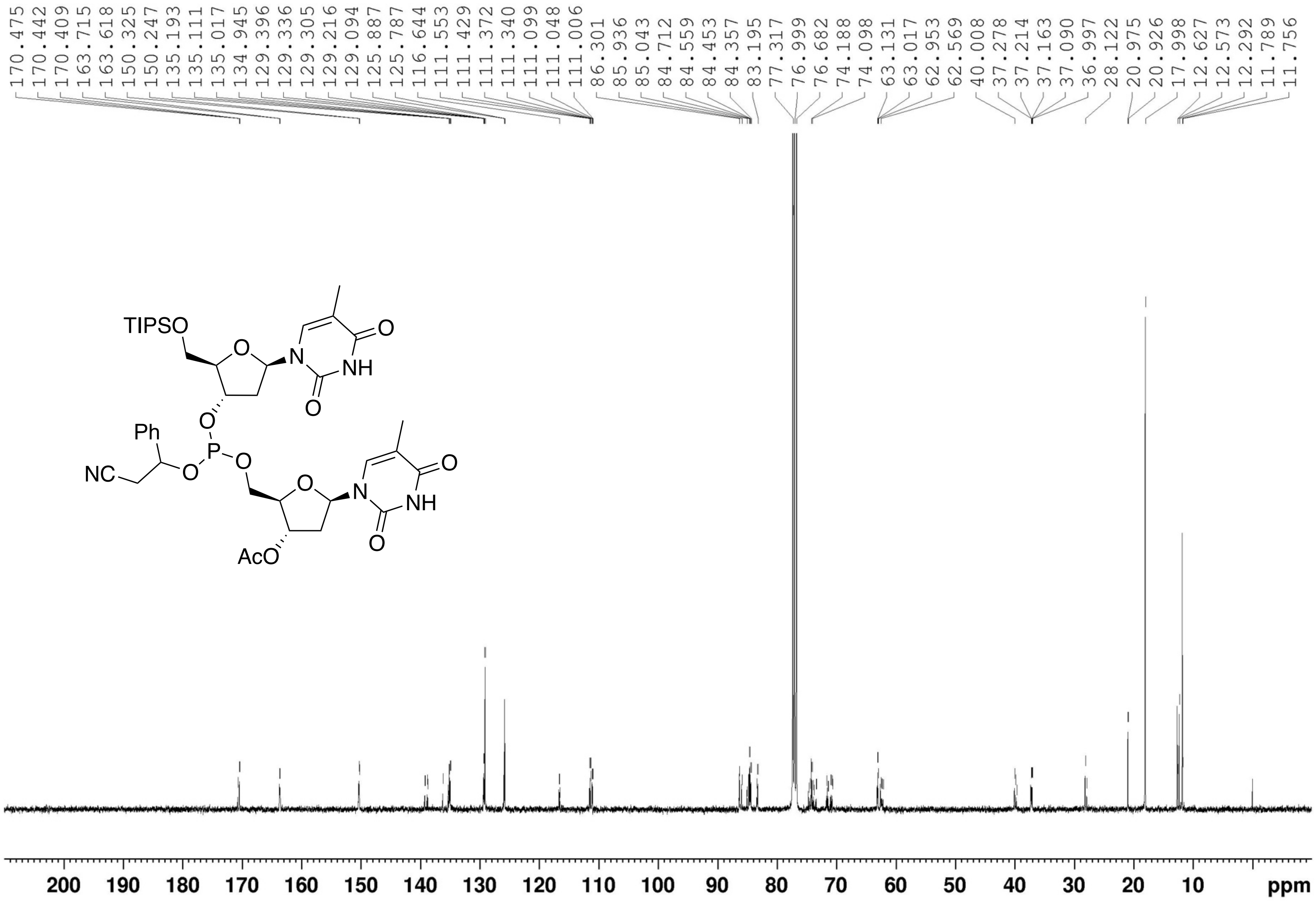


$^{13}C$ NMR (100.6 MHz, $CDCl_3$) spectrum of **1a-1**

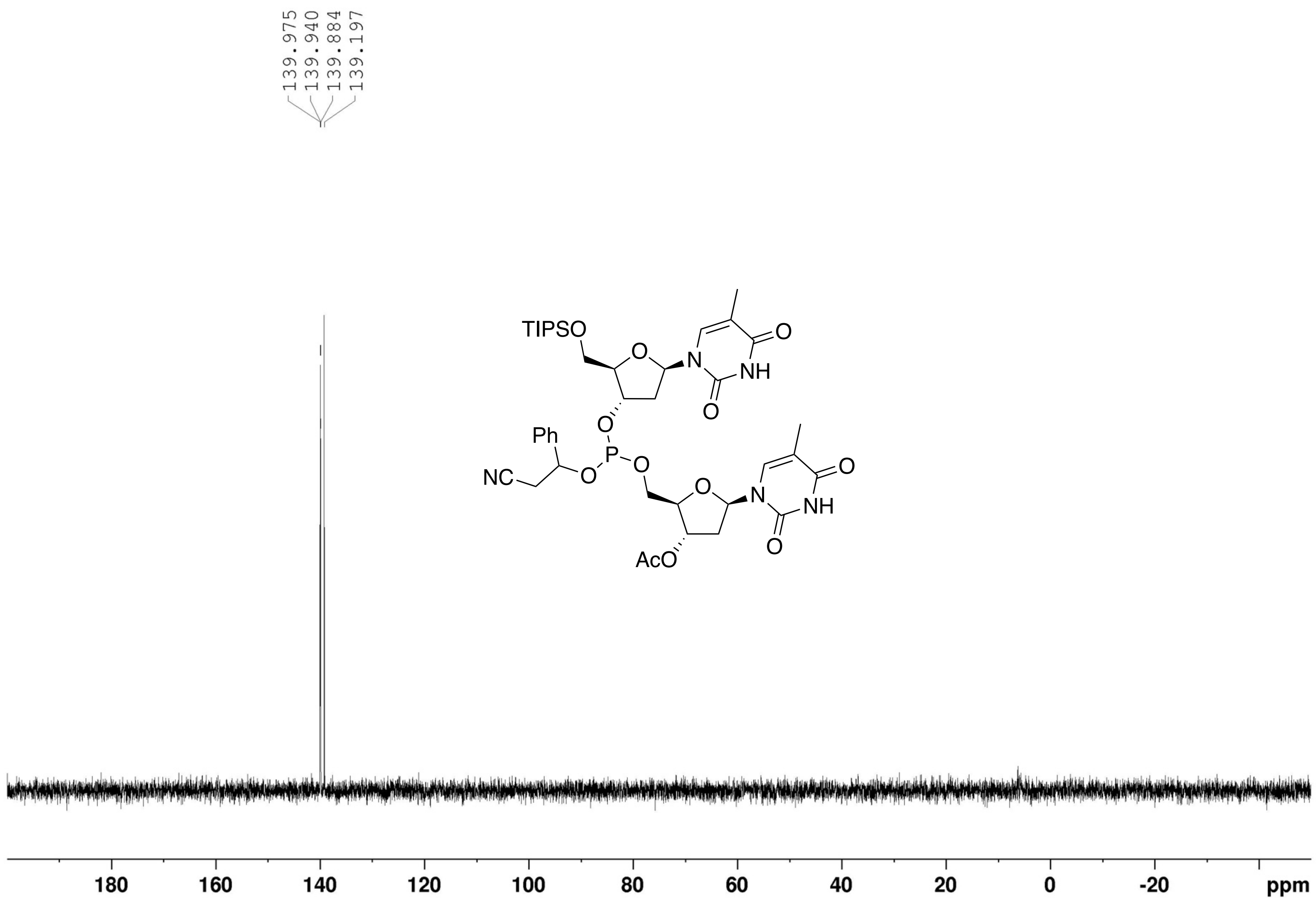


$^{31}P$ NMR (162 MHz, $CDCl_3$) spectrum of **1a-1**

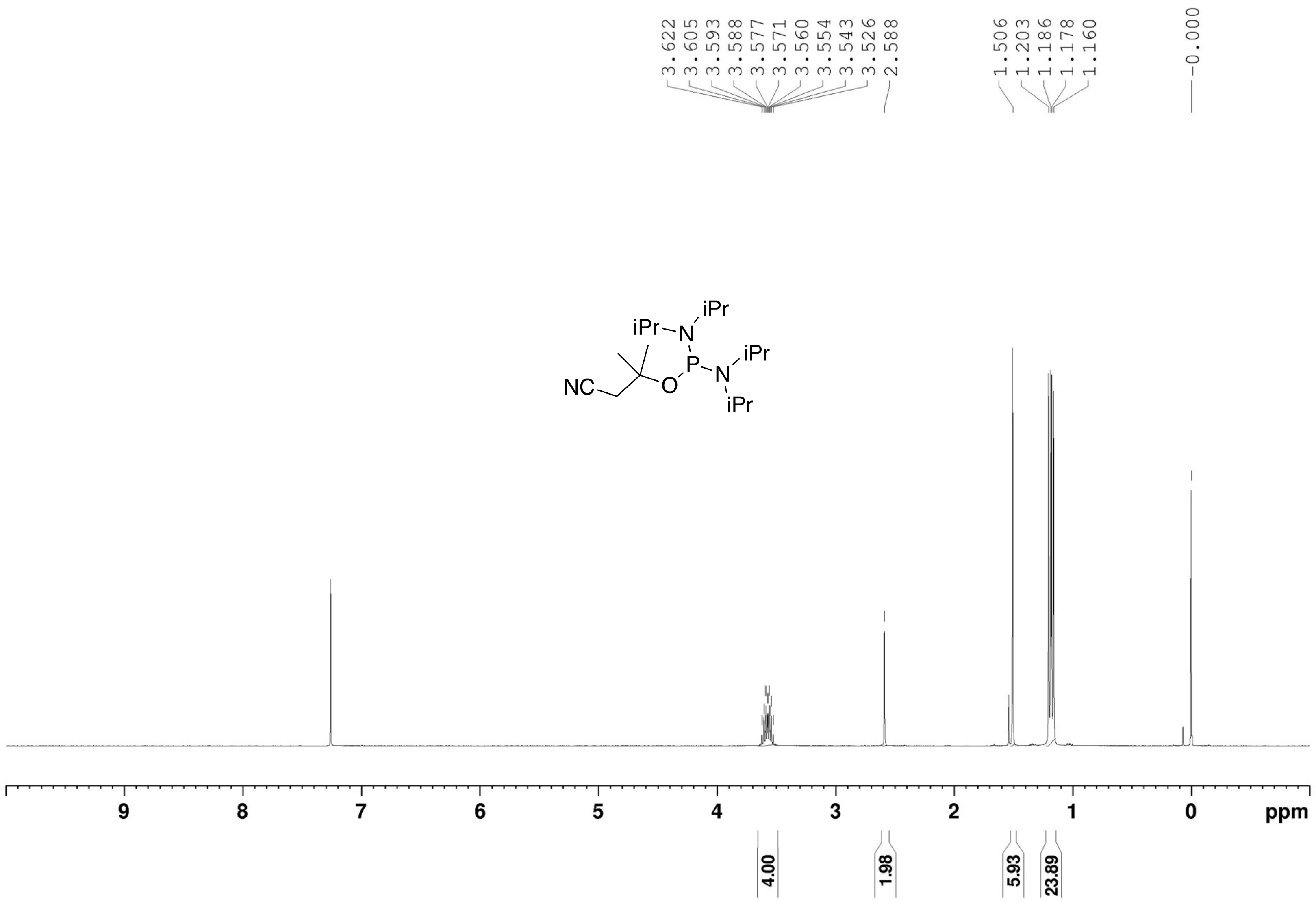


$^{1}$H NMR (400 MHz, $CDCl_3$) spectrum of **S2-2**

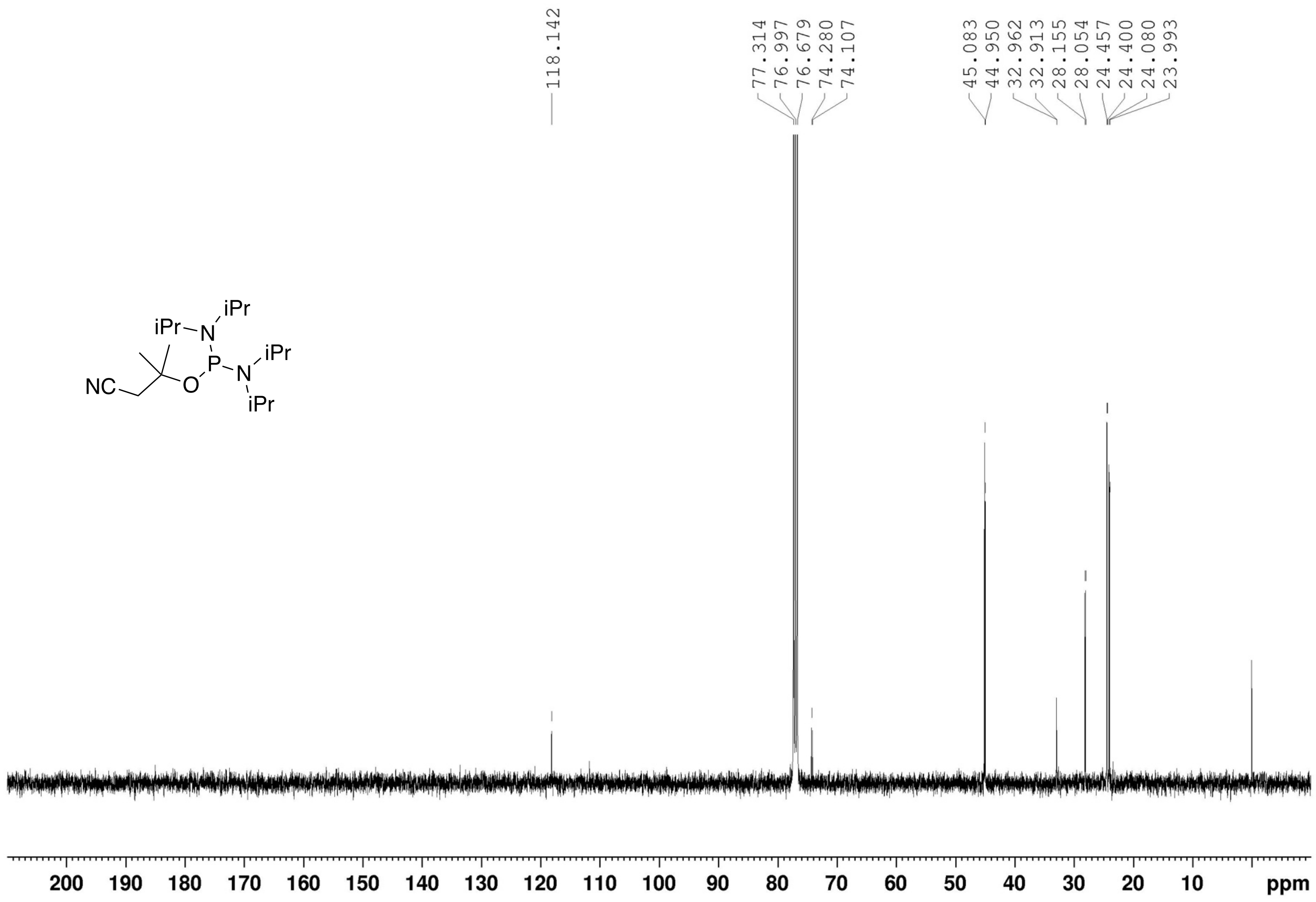


$^{13}$C NMR (100.6 MHz, $CDCl_3$) spectrum of **S2-2**

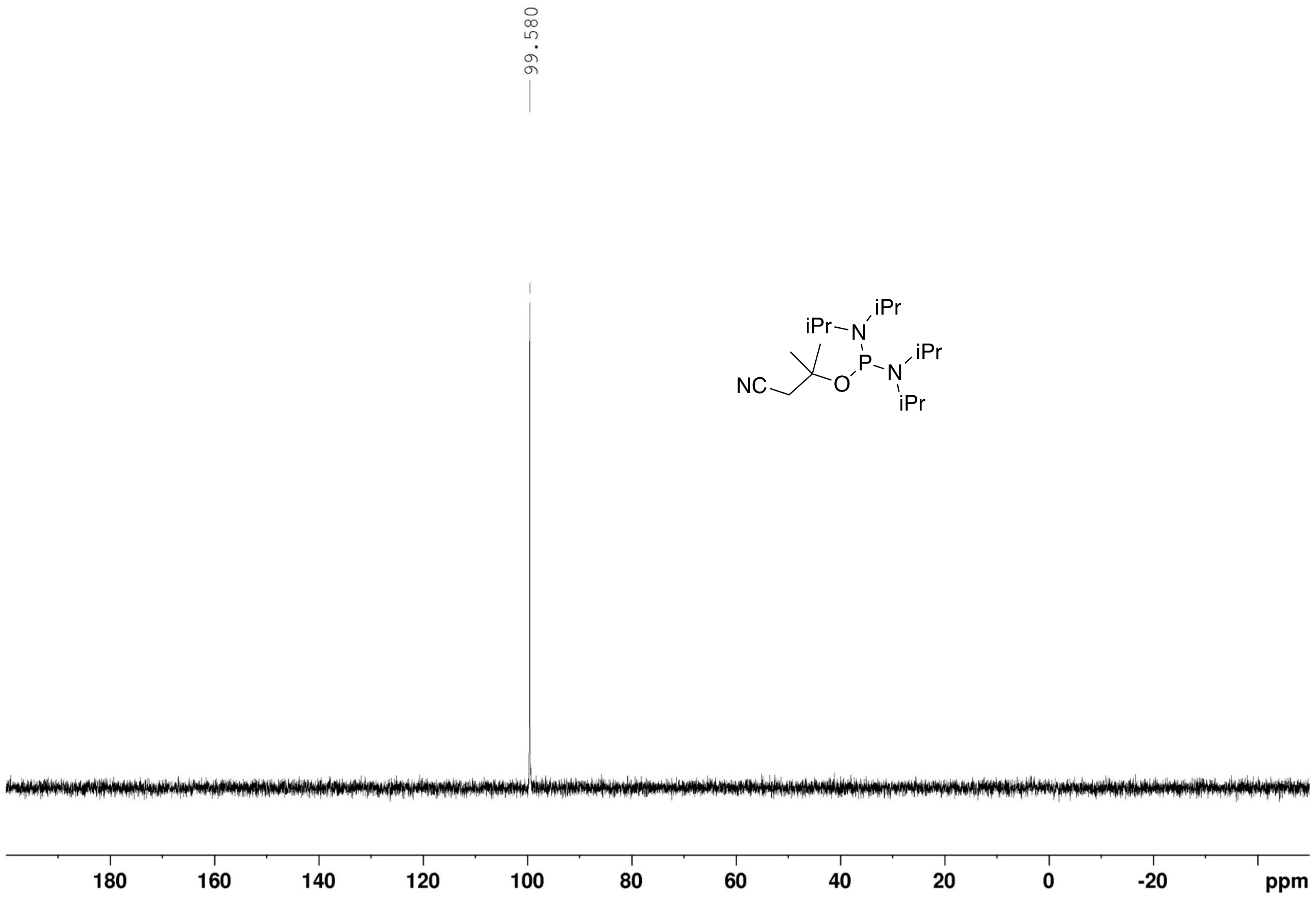


$^{31}P$ NMR (162 MHz, $CDCl_3$) spectrum of **S2-2**

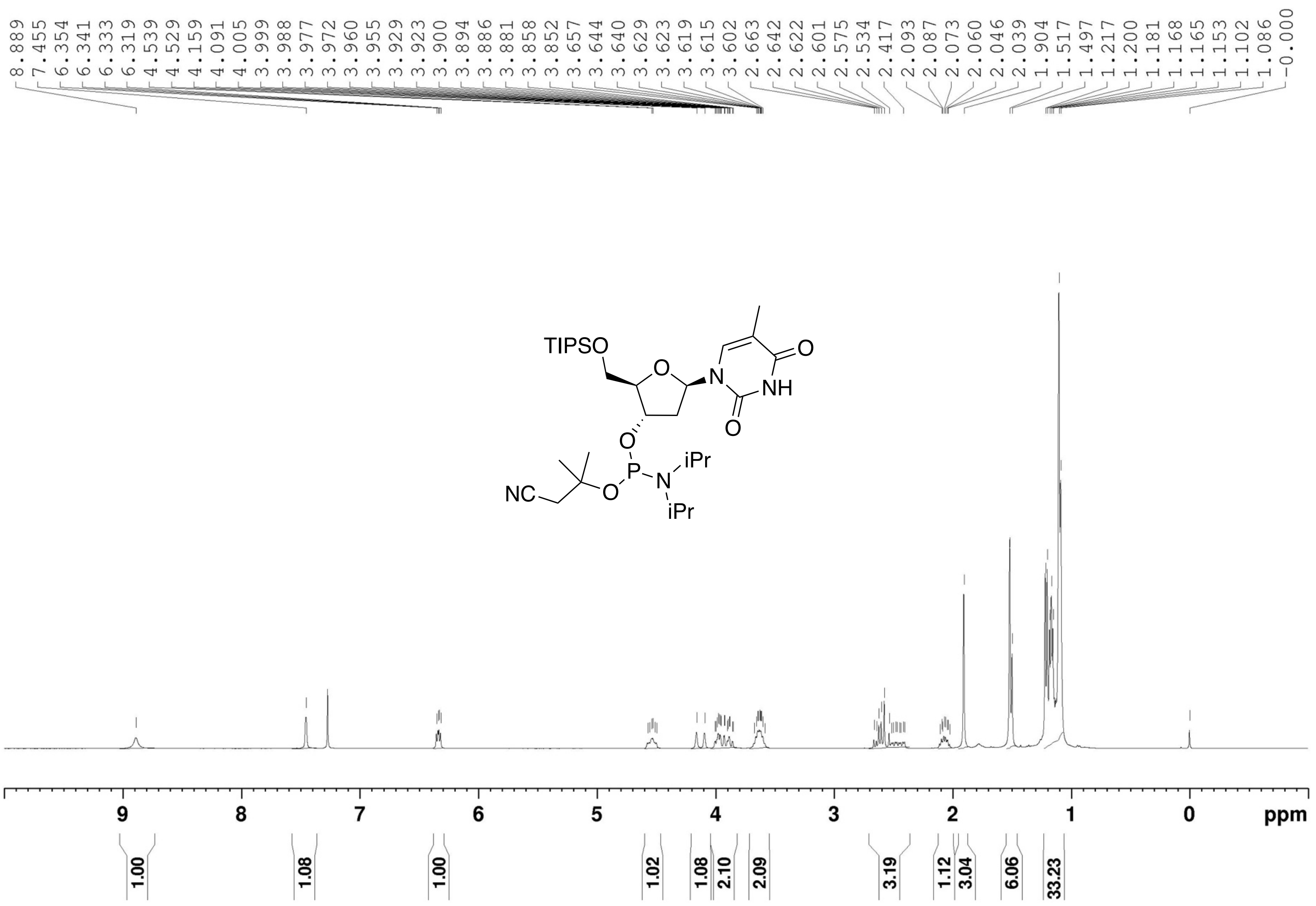


$^{1}$H NMR (400 MHz, $CDCl_3$) spectrum of **S3-2**

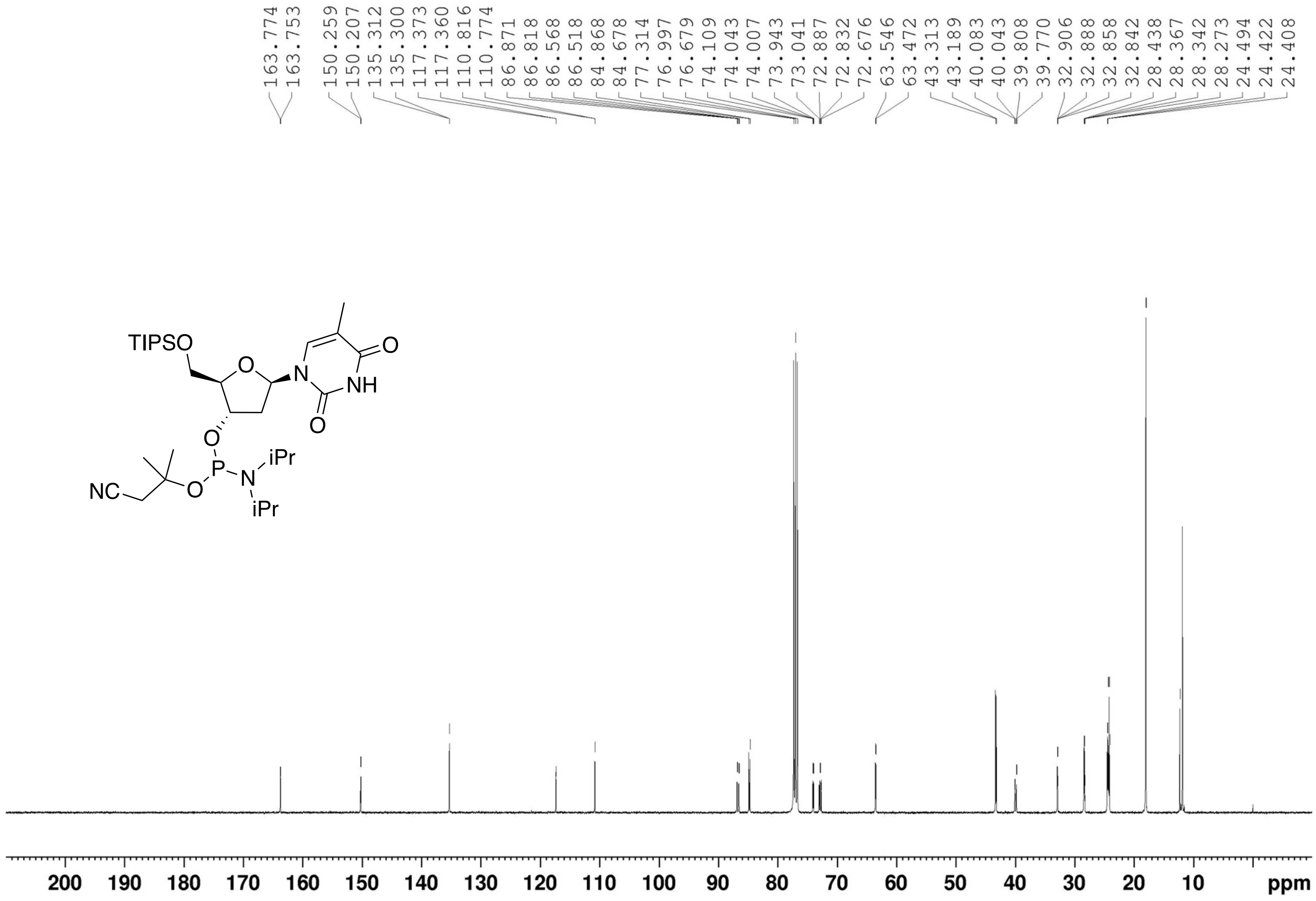


$^{13}C$ NMR (100.6 MHz, $CDCl_3$) spectrum of **S3-2**

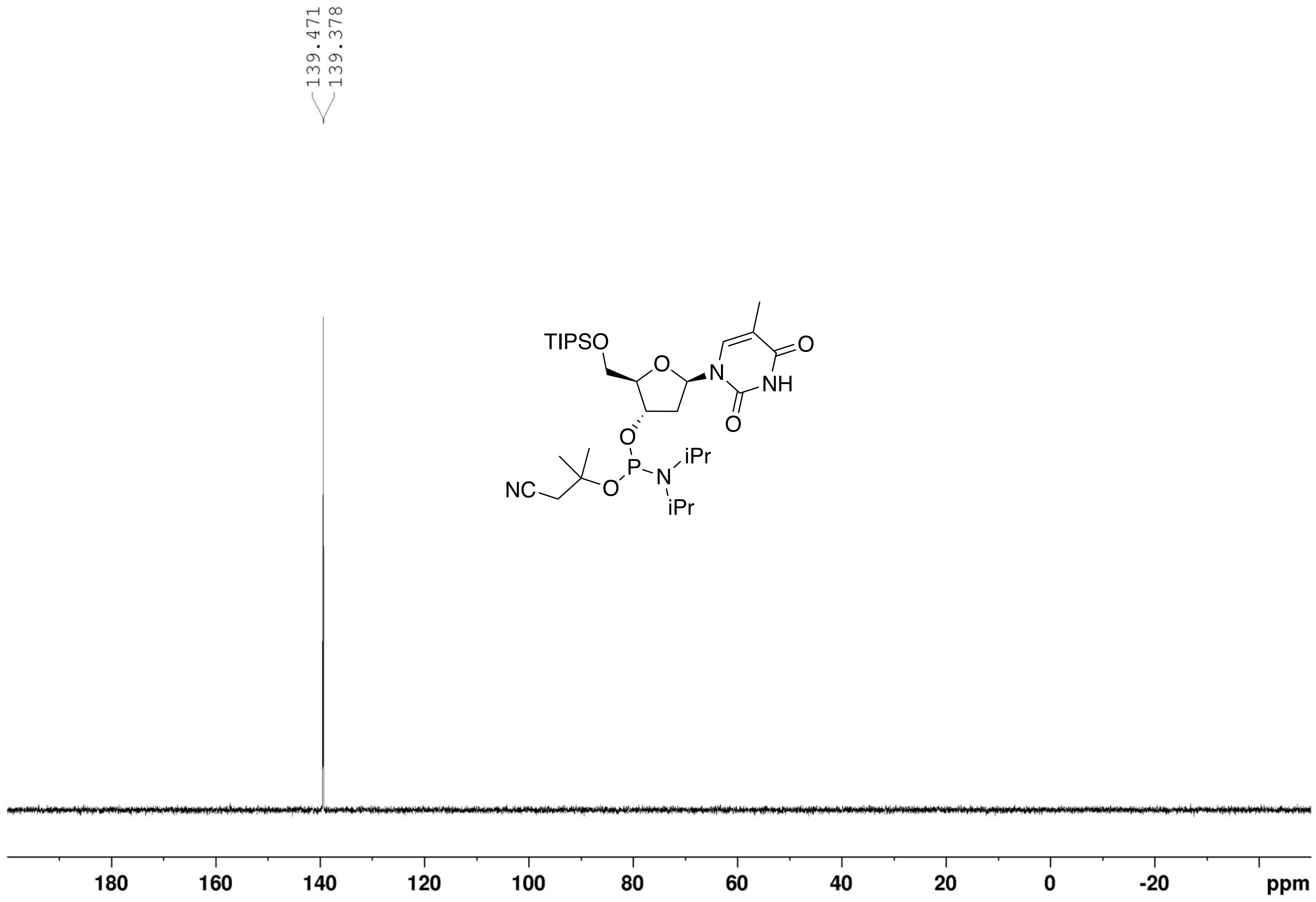


$^{31}P$ NMR (162 MHz, $CDCl_3$) spectrum of **S3-2**

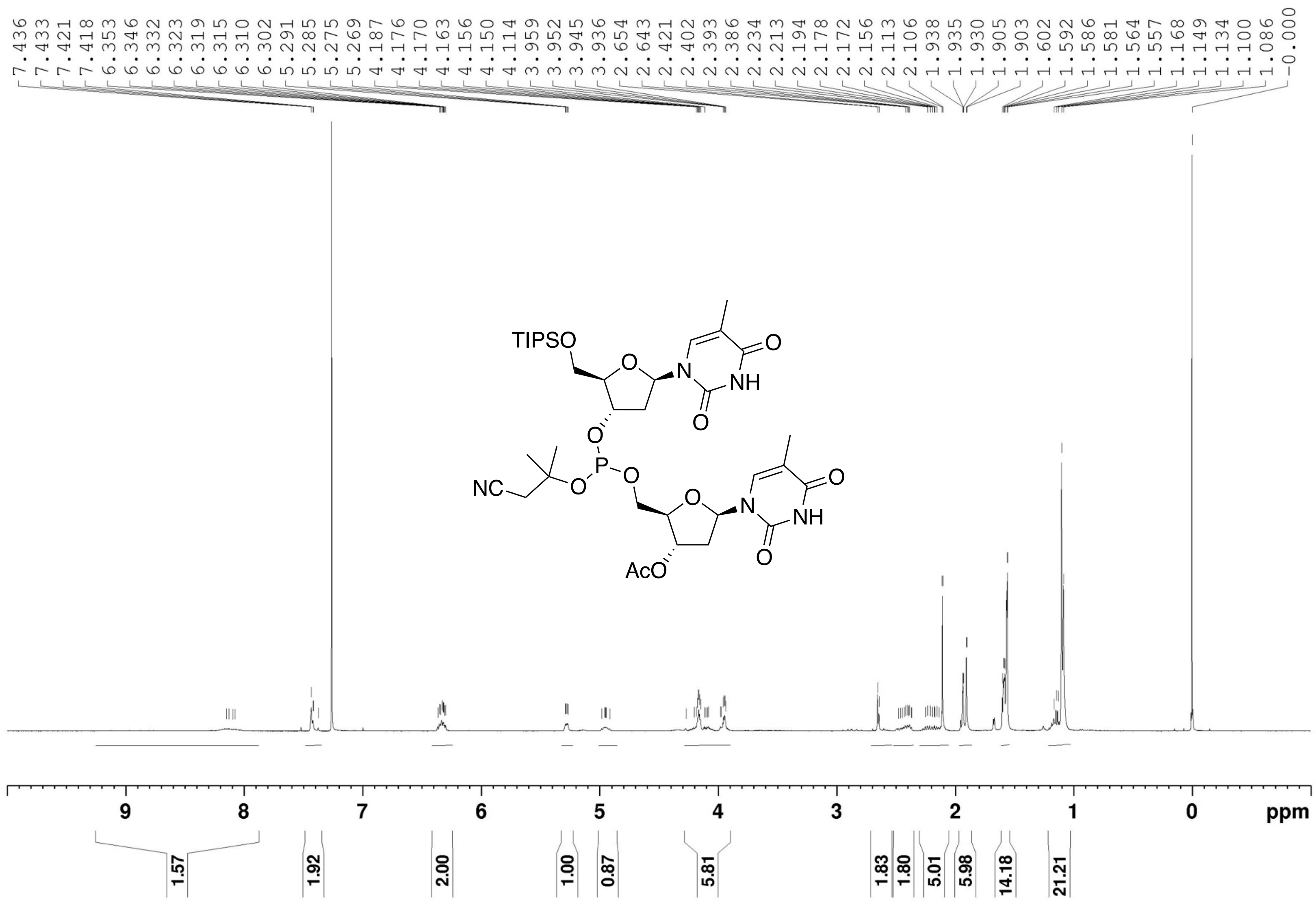

$^{1}H$ NMR (400 MHz, $CDCl_3$) spectrum of **1a-2**

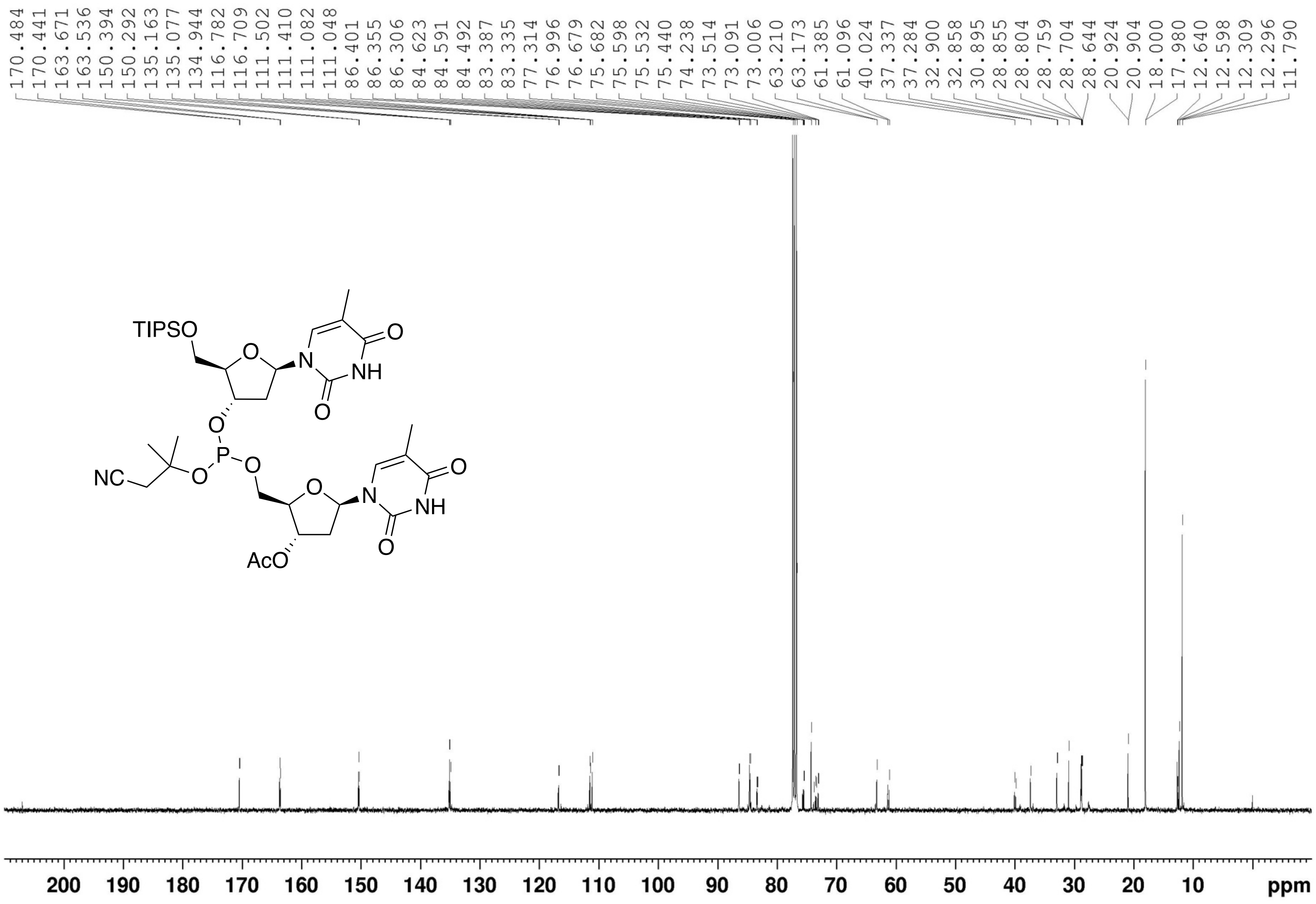


$^{13}C$ NMR (100.6 MHz, $CDCl_3$) spectrum of **1a-2**

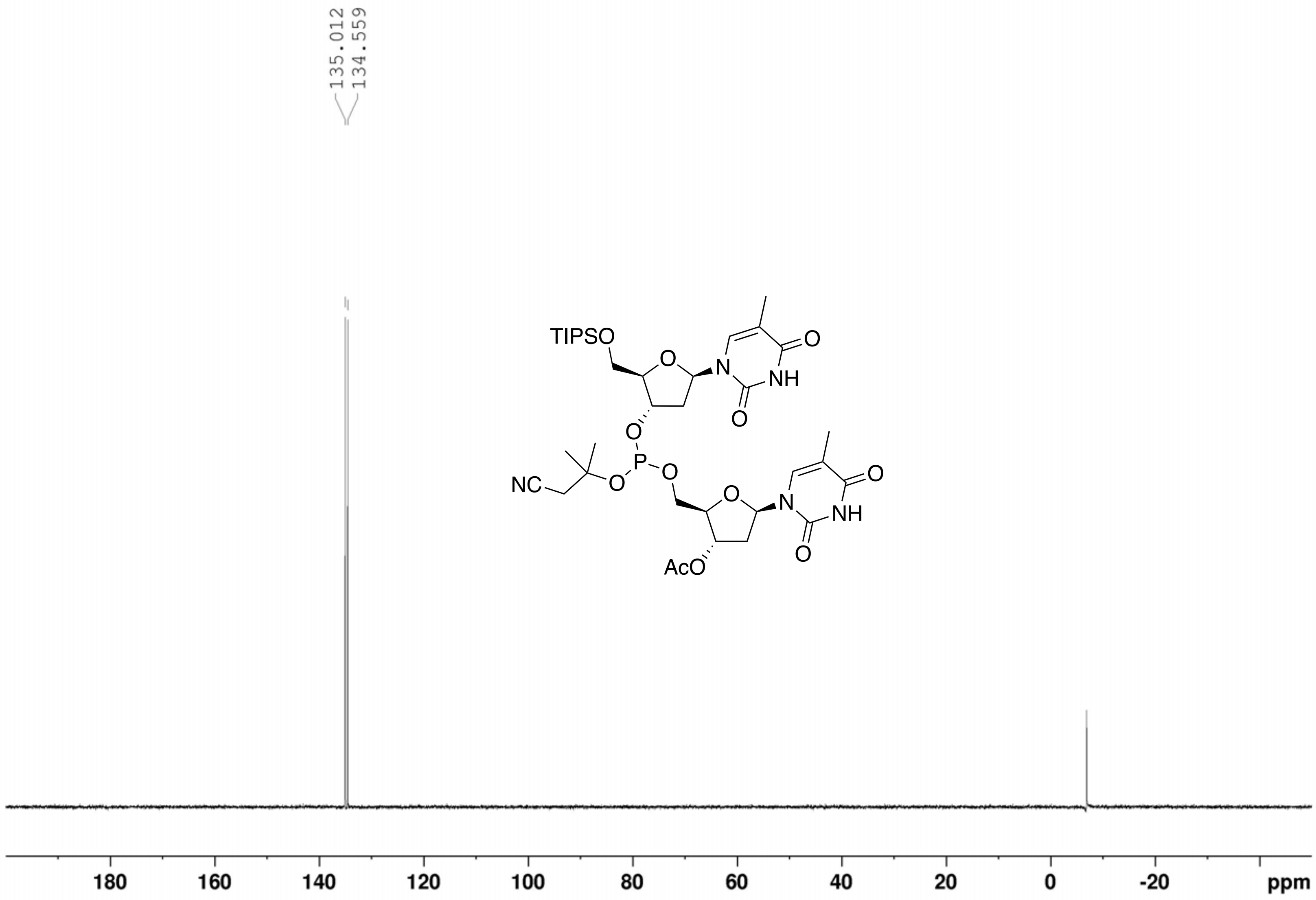


$^{31}$P NMR (162 MHz, $CDCl_3$) spectrum of **1a-2**

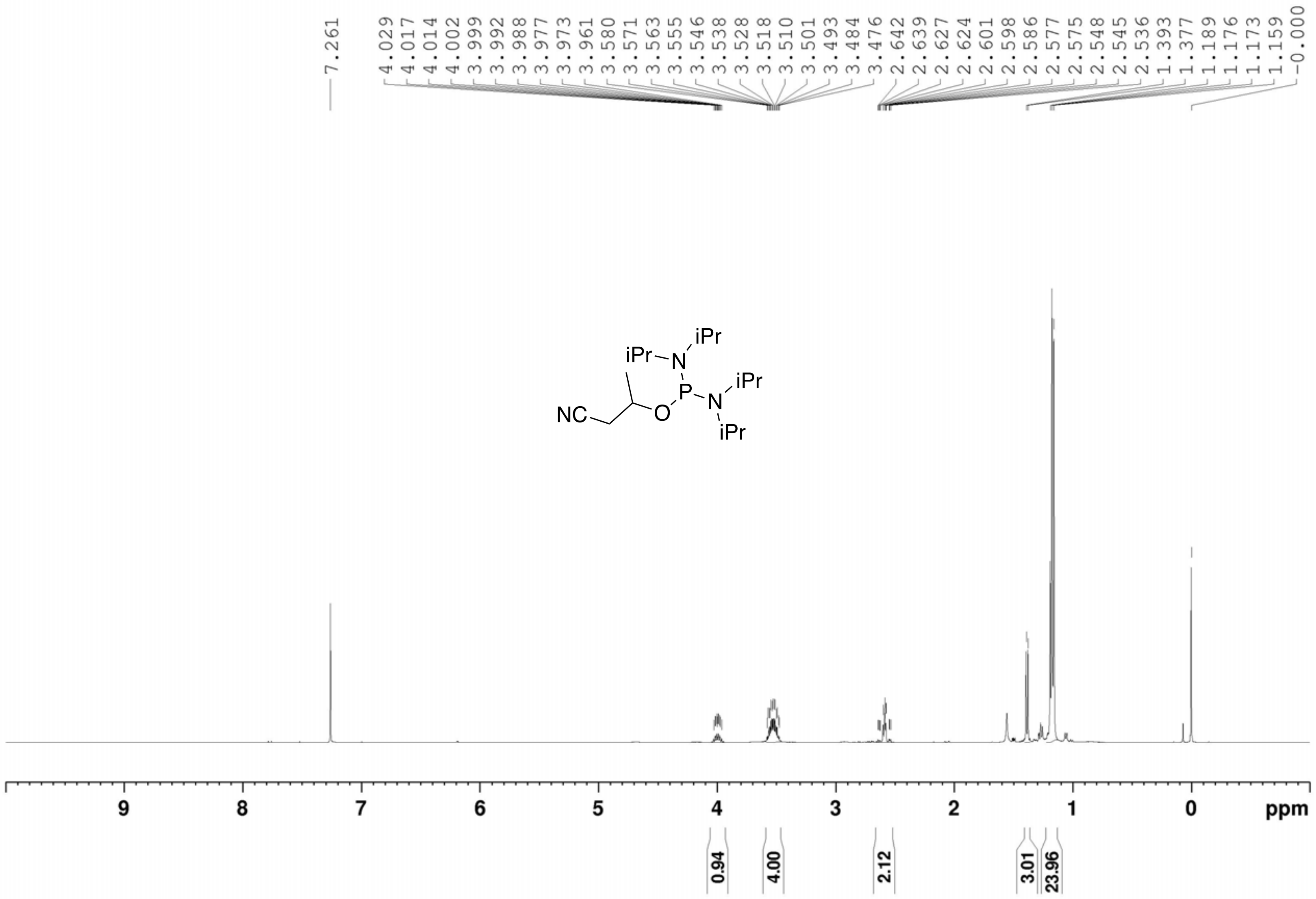


$^{1}$H NMR (400 MHz, $CDCl_3$) spectrum of **S2-3**

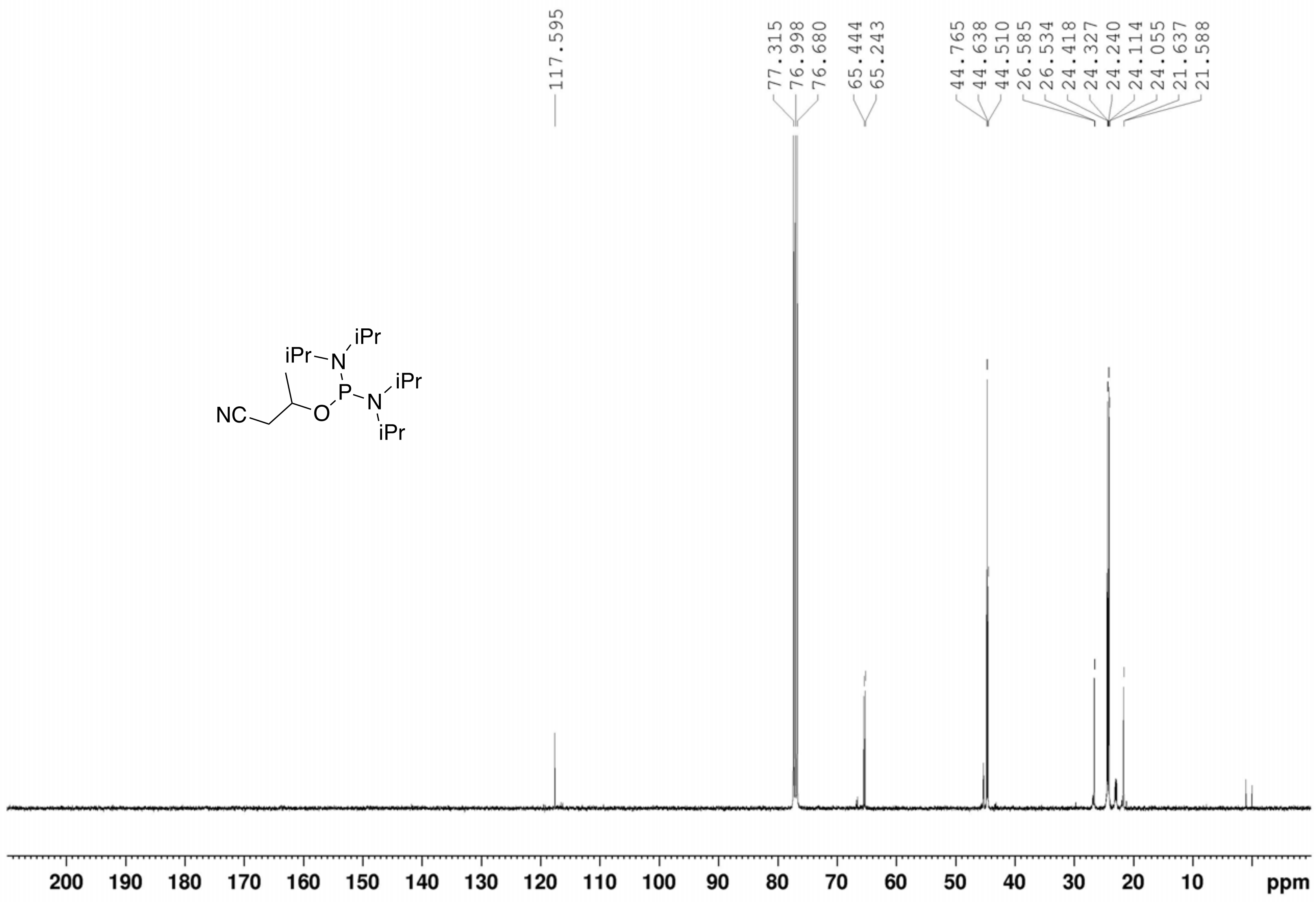


$^{13}C$ NMR (100.6 MHz, $CDCl_3$) spectrum of **S2-3**

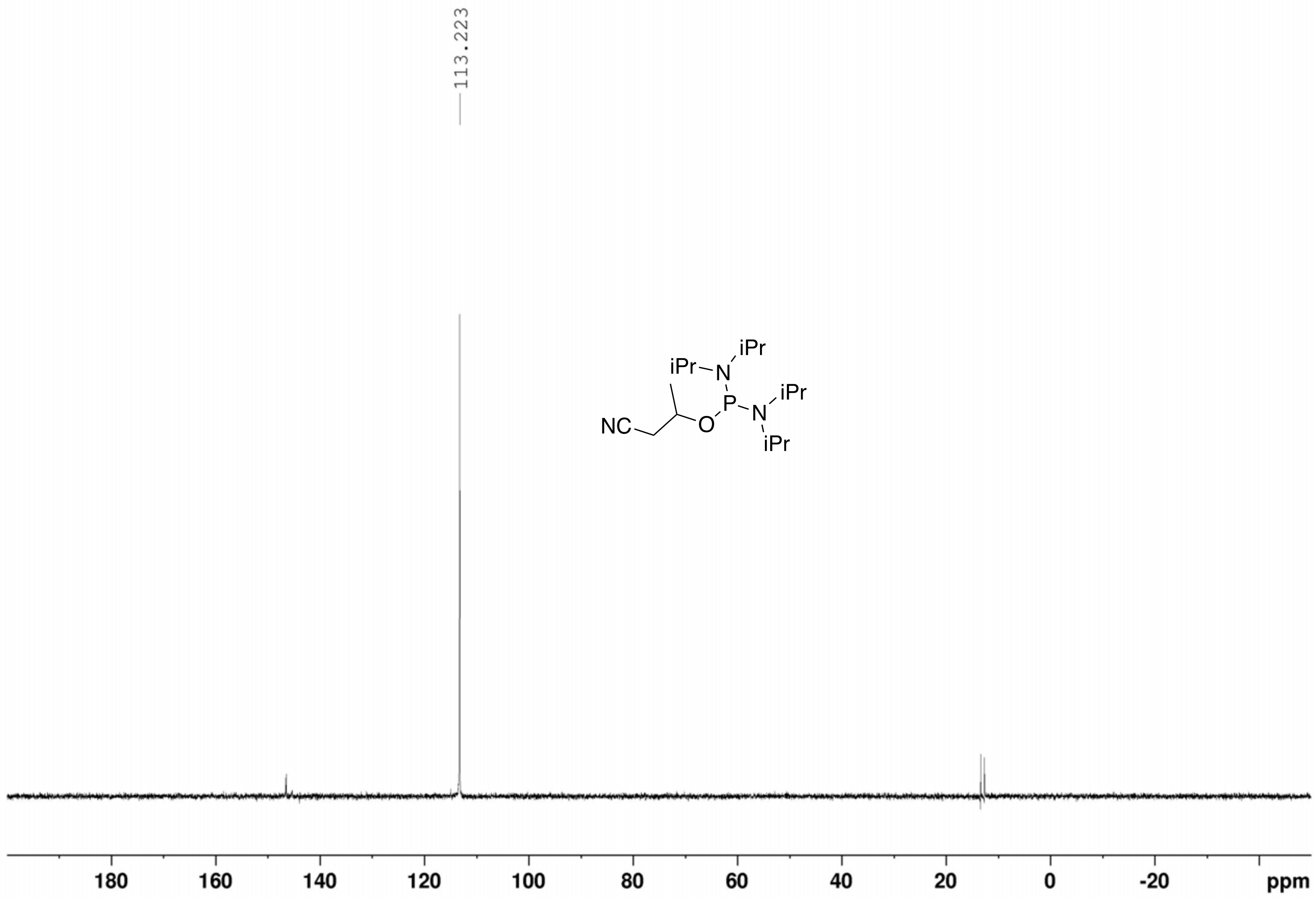


$^{31}P$ NMR (162 MHz, $CDCl_3$) spectrum of **S2-3**

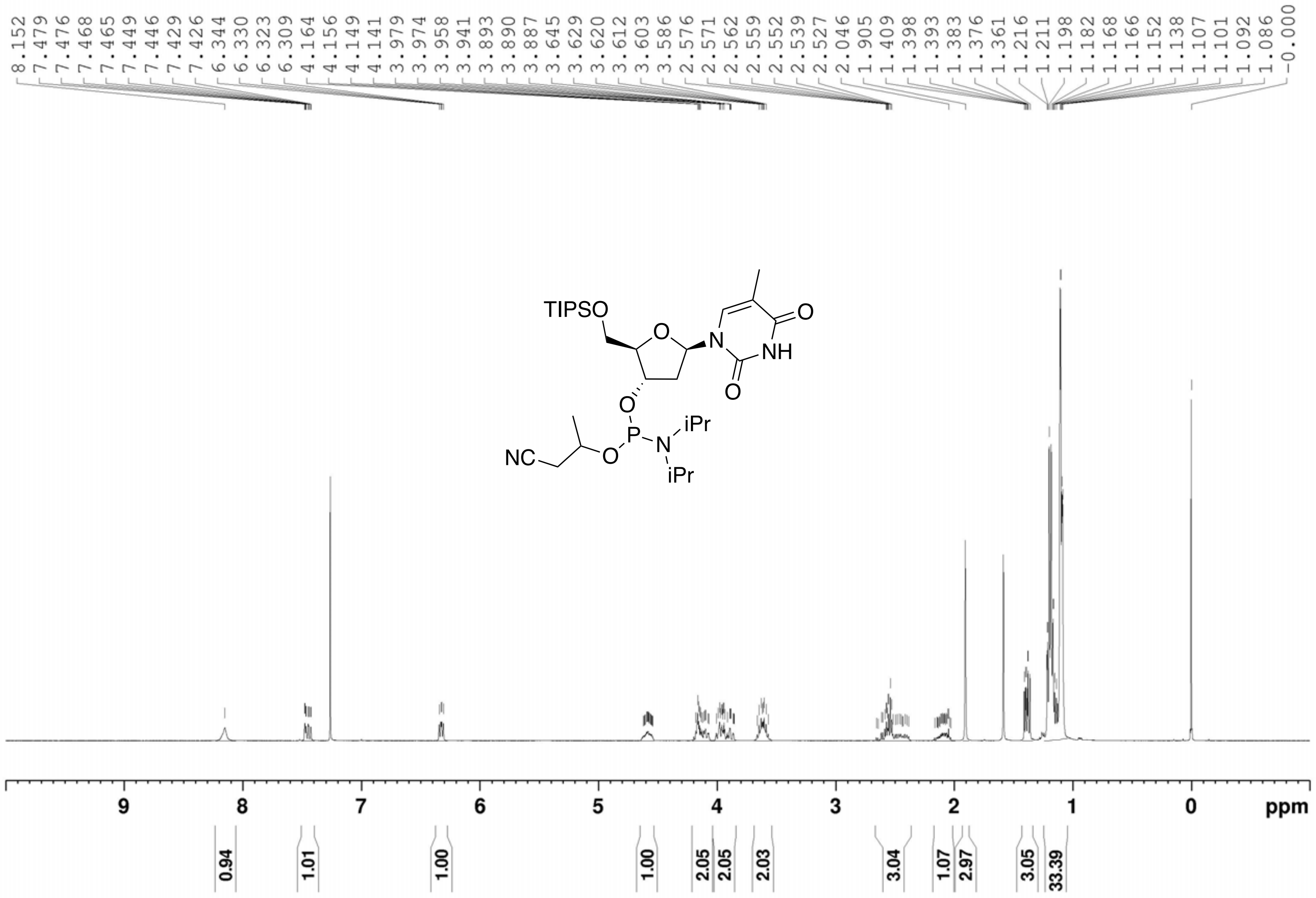

$^1H$ NMR (400 MHz, $CDCl_3$) spectrum of **S3-3**

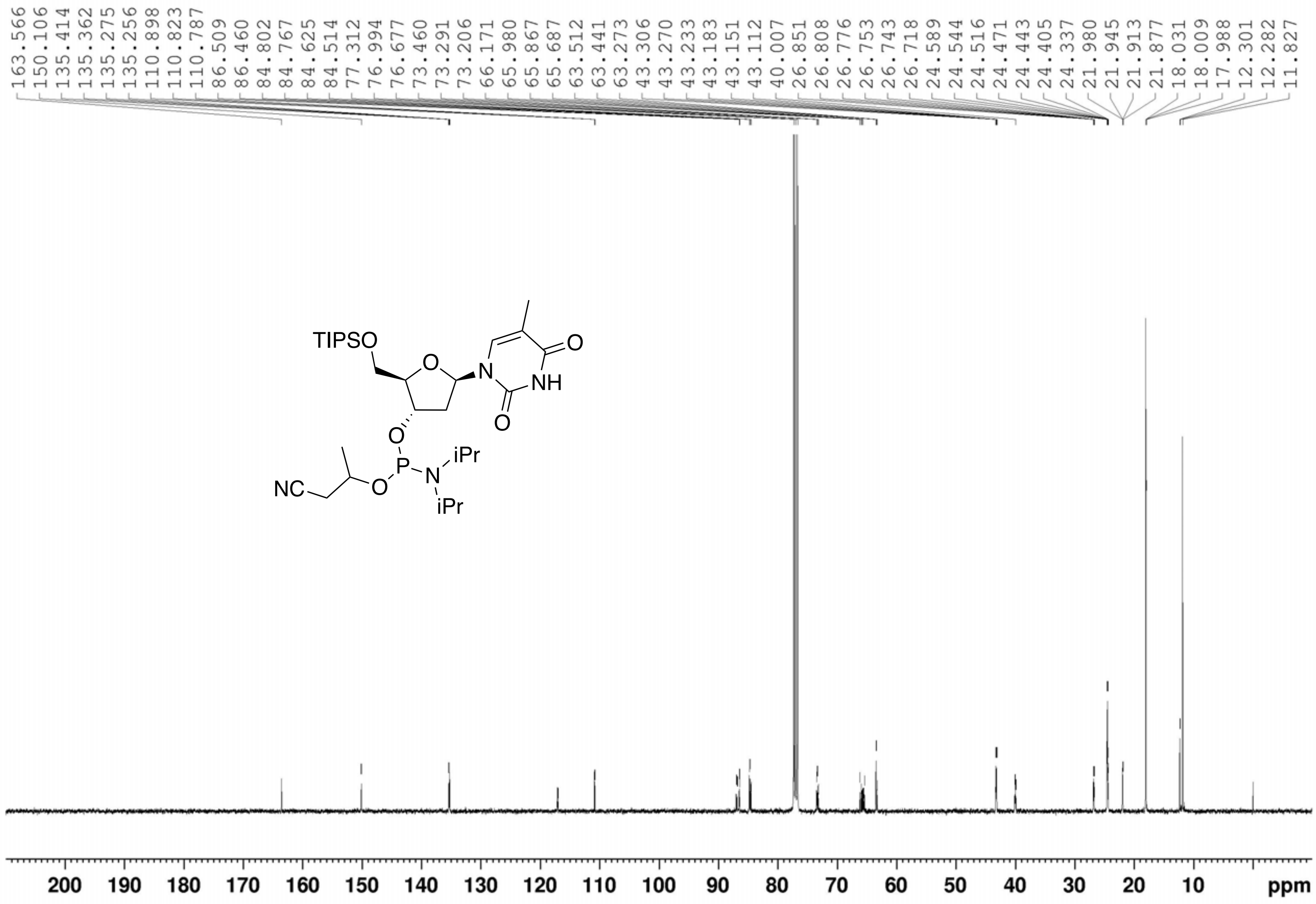


$^{13}$C NMR (100.6 MHz, $CDCl_3$) spectrum of **S3-3**

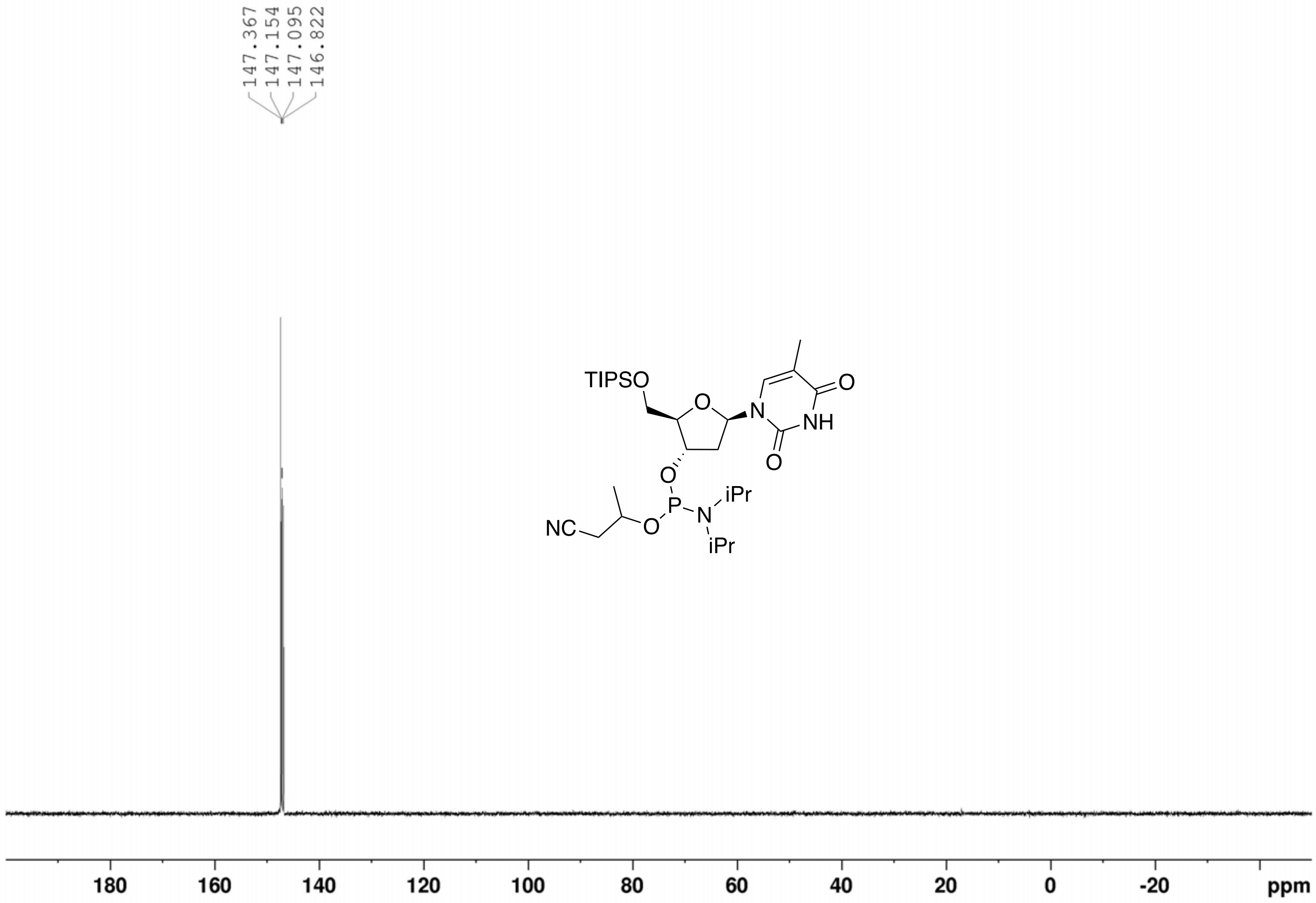


$^{31}P$ NMR (162 MHz, $CDCl_3$) spectrum of **S3-3**

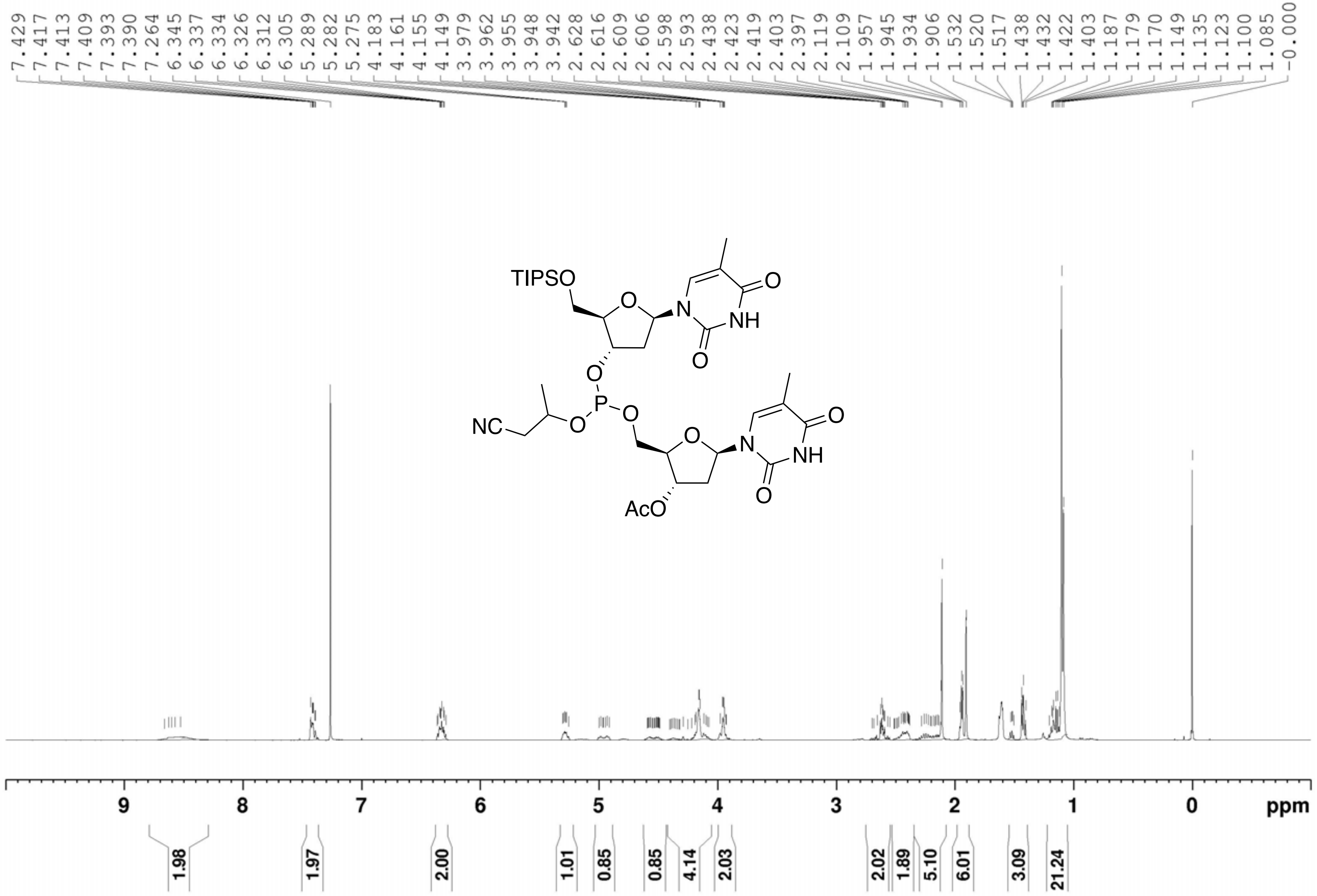


$^{1}$H NMR (400 MHz, $CDCl_3$) spectrum of **1a-3**

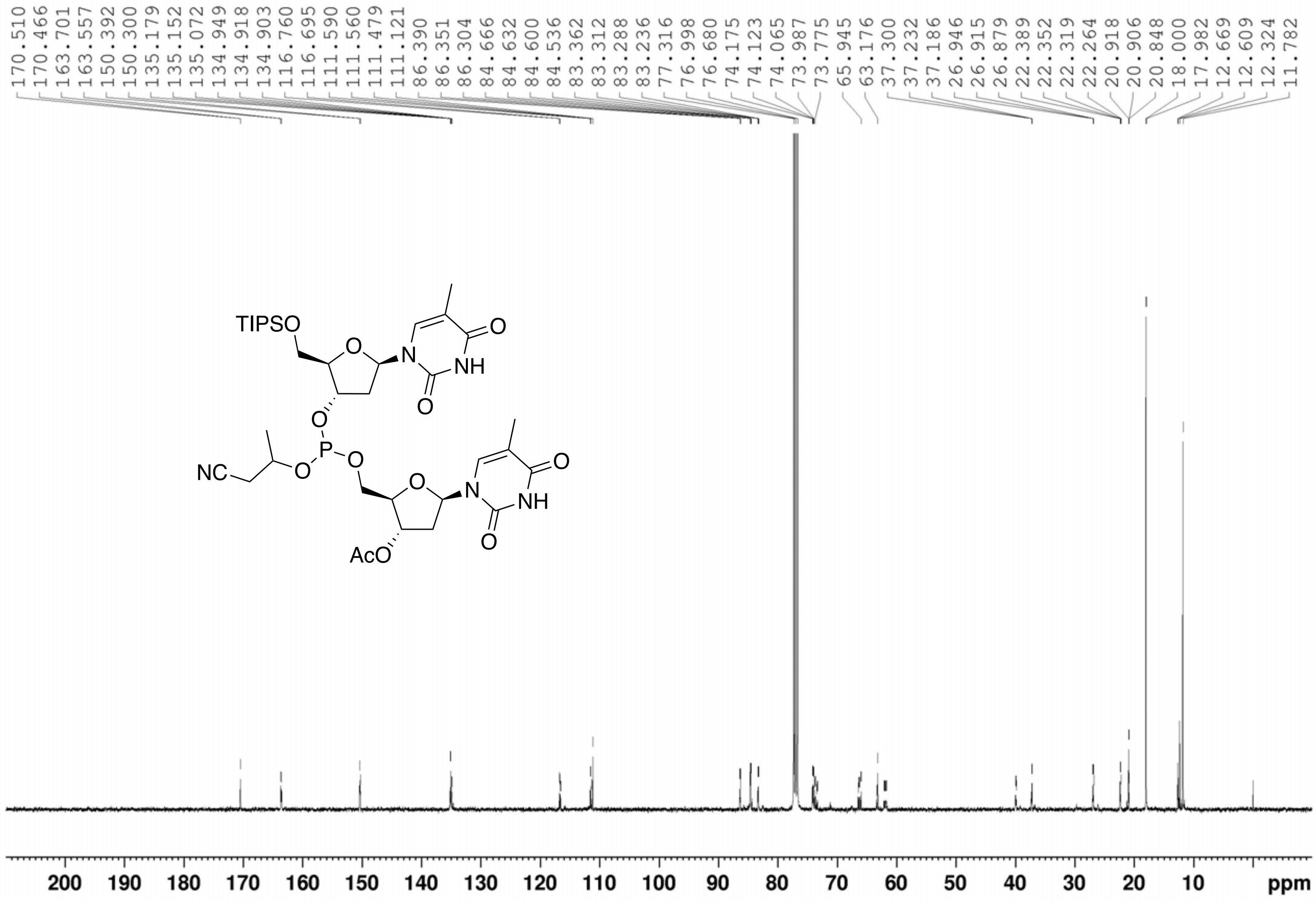


$^{13}$C NMR (100.6 MHz, $CDCl_3$) spectrum of **1a-3**

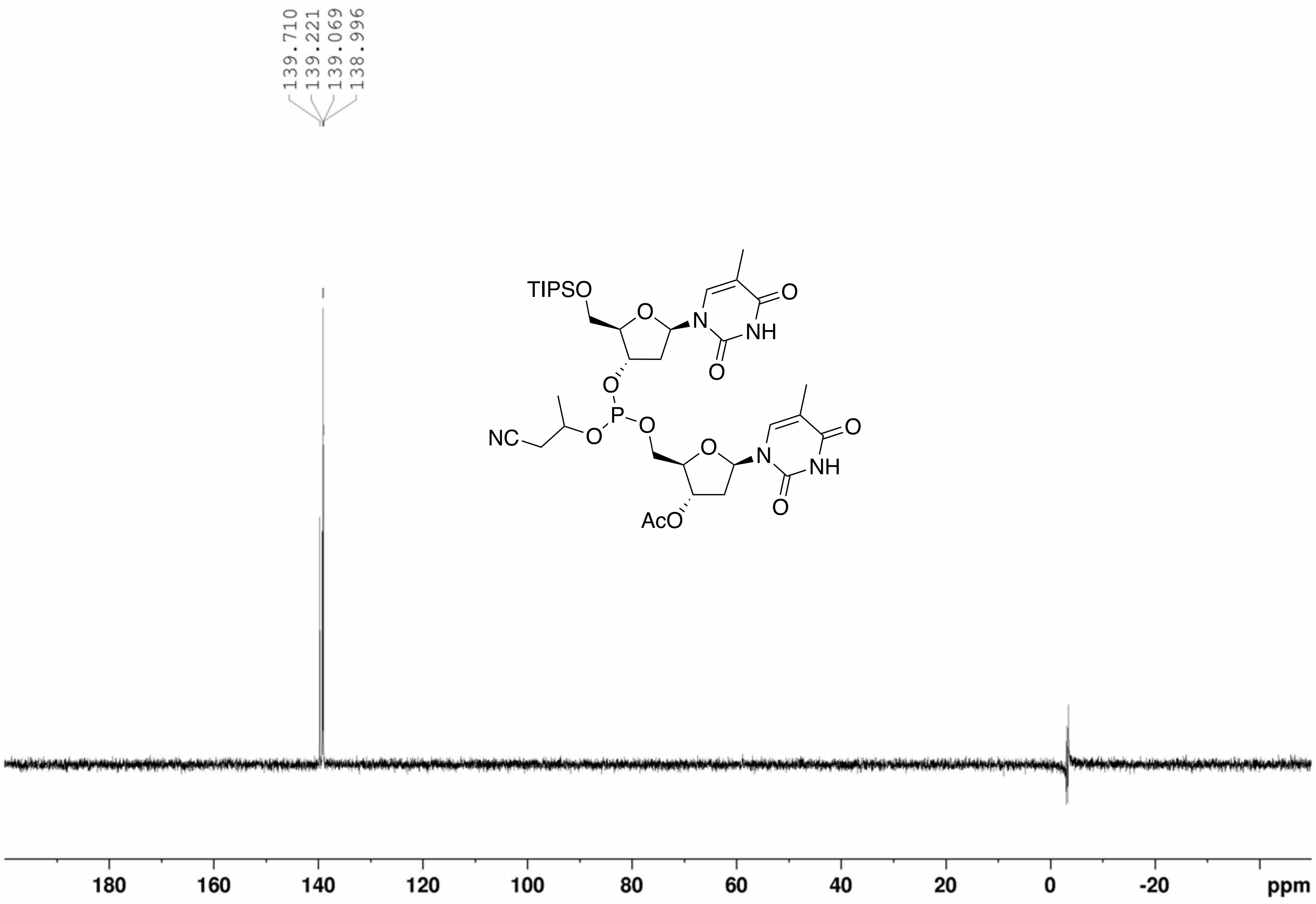


$^{31}$P NMR (162 MHz, $CDCl_3$) spectrum of **1a-3**

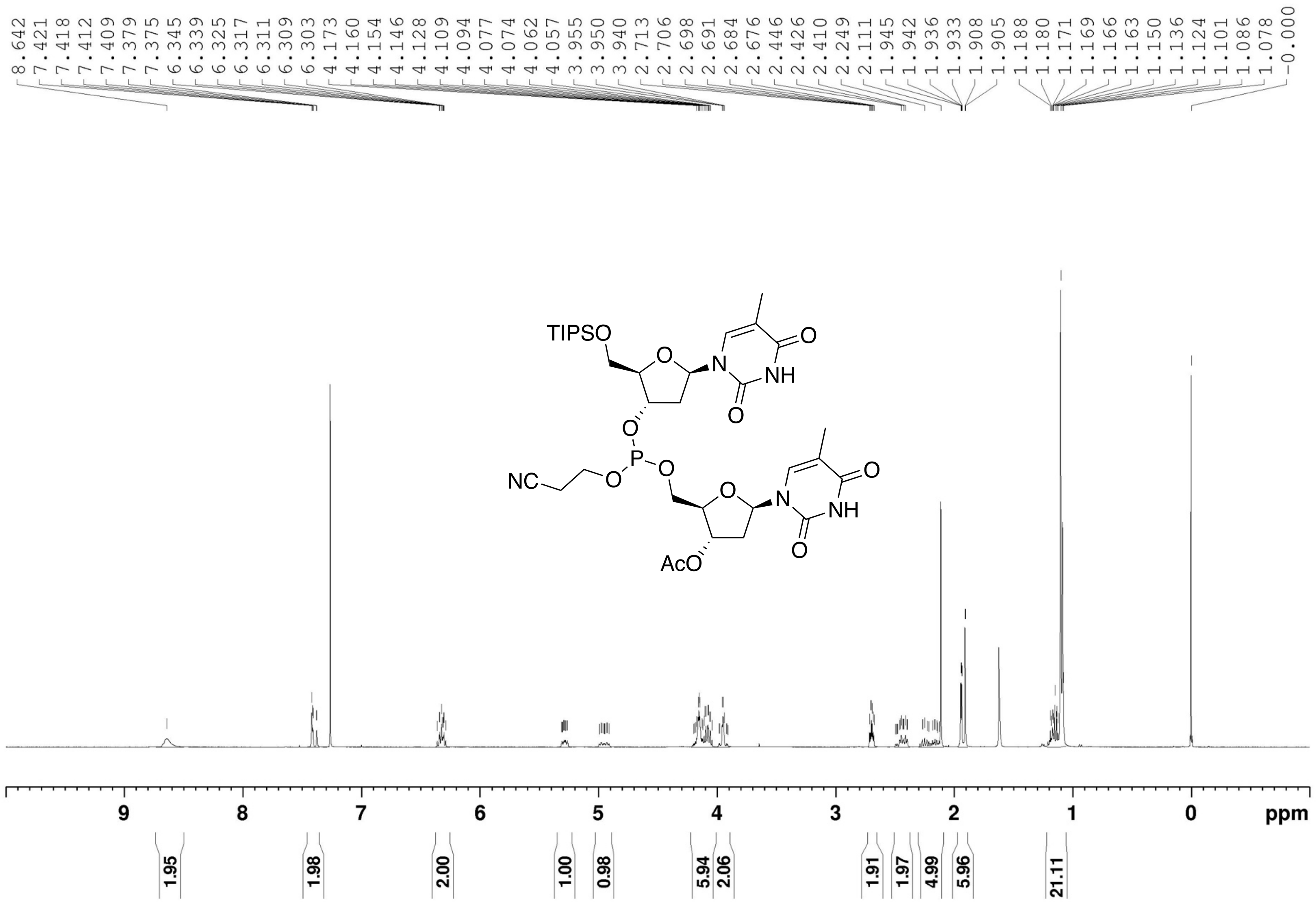


$^{1}$H NMR (400 MHz, $CDCl_3$) spectrum of **1a-4**

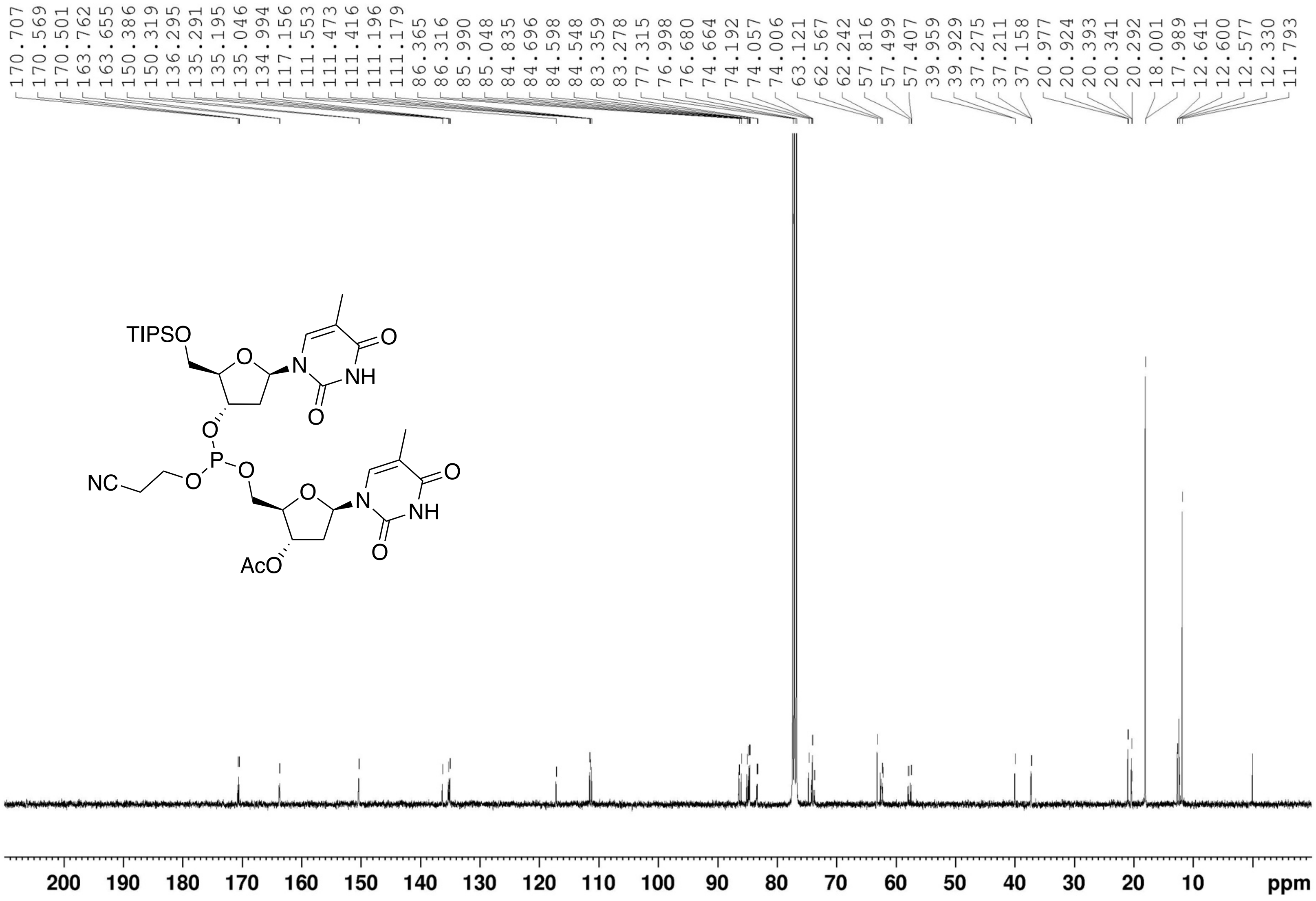


$^{13}$C NMR (100.6 MHz, $CDCl_3$) spectrum of **1a-4**

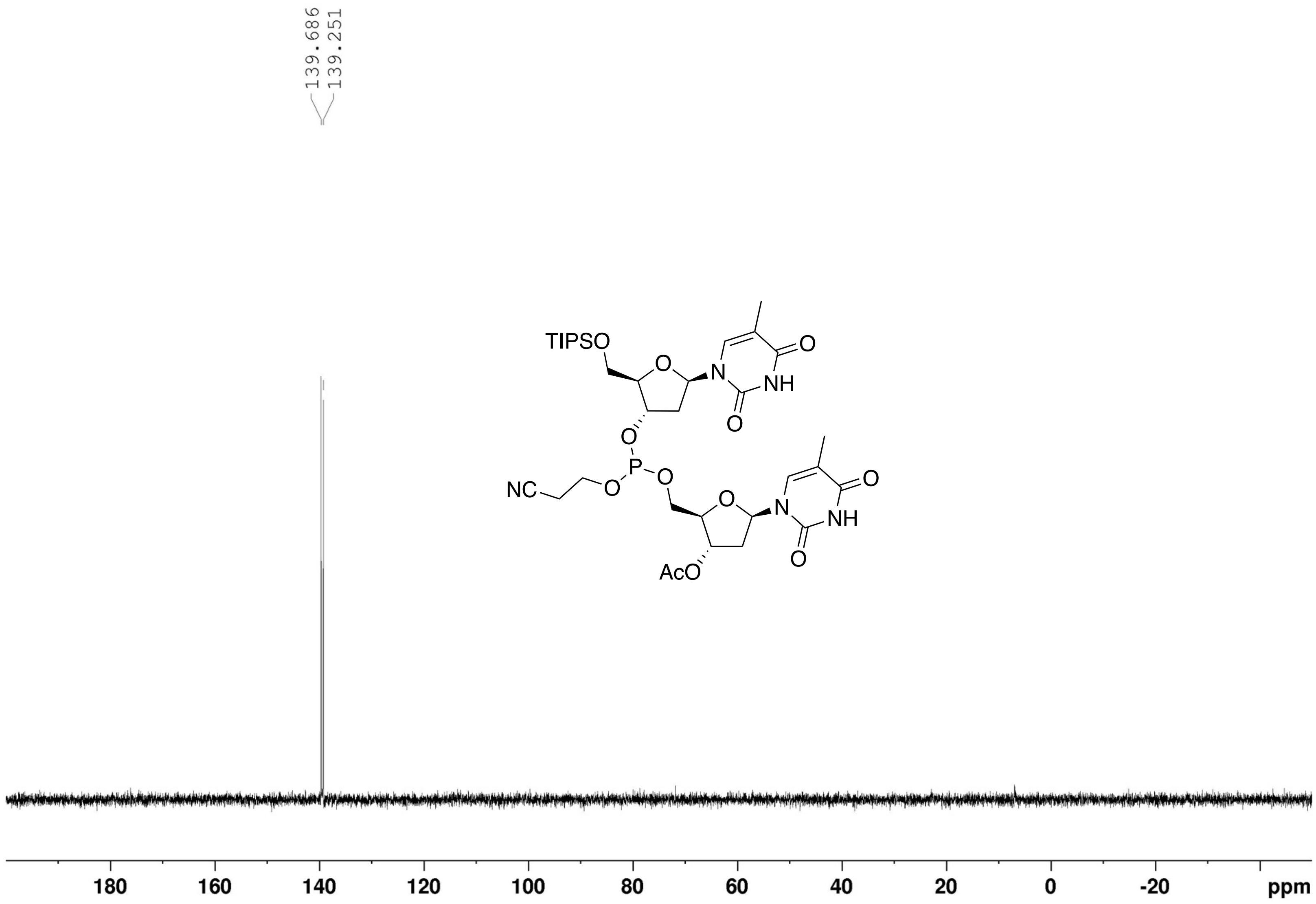


$^{31}P$ NMR (162 MHz, $CDCl_3$) spectrum of **1a-4**

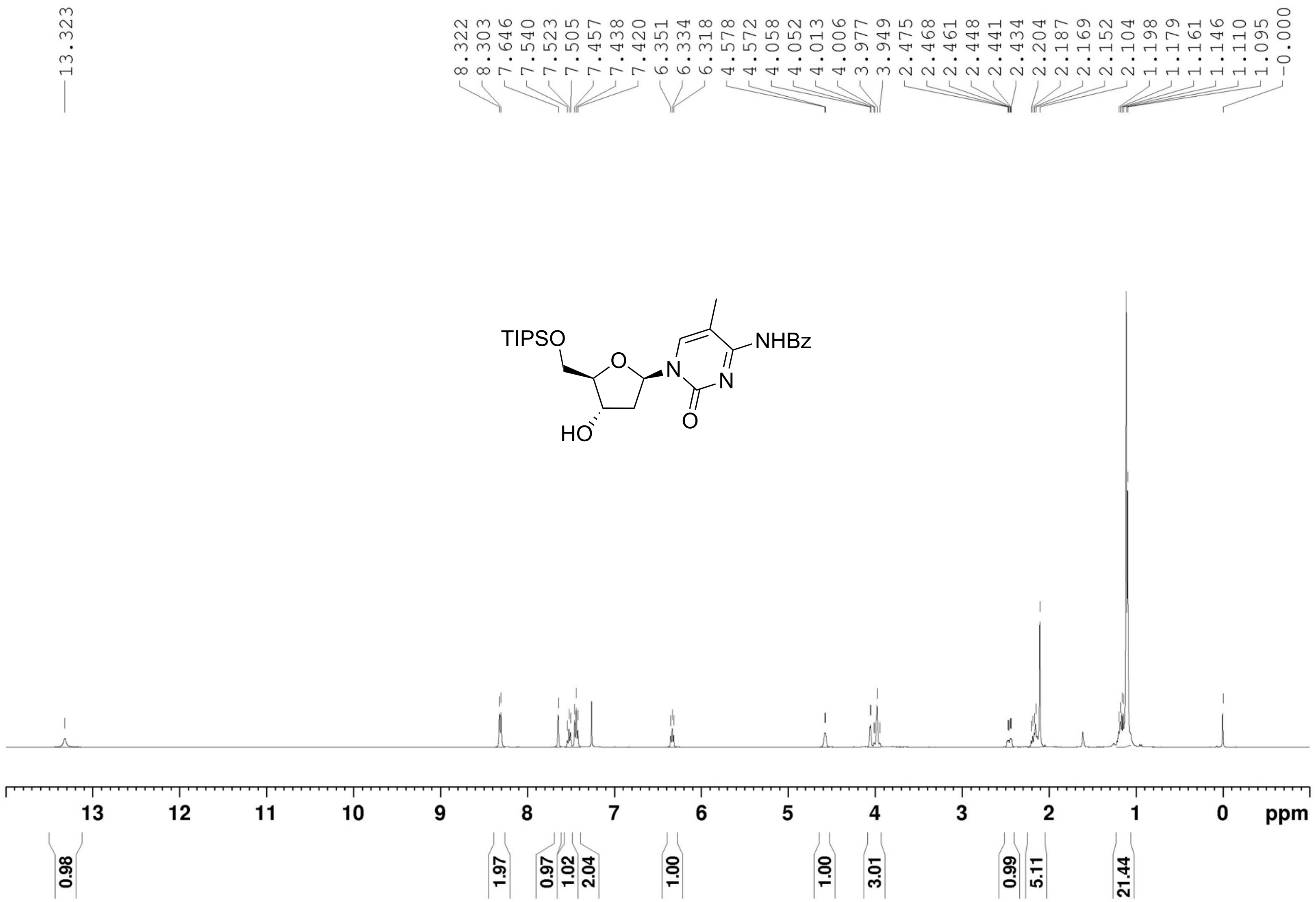


$^{1}$H NMR (400 MHz, $CDCl_3$) spectrum of ***N*-Benzoyl-5’-O-TIPS-5-methyl-2’-deoxycytidine**

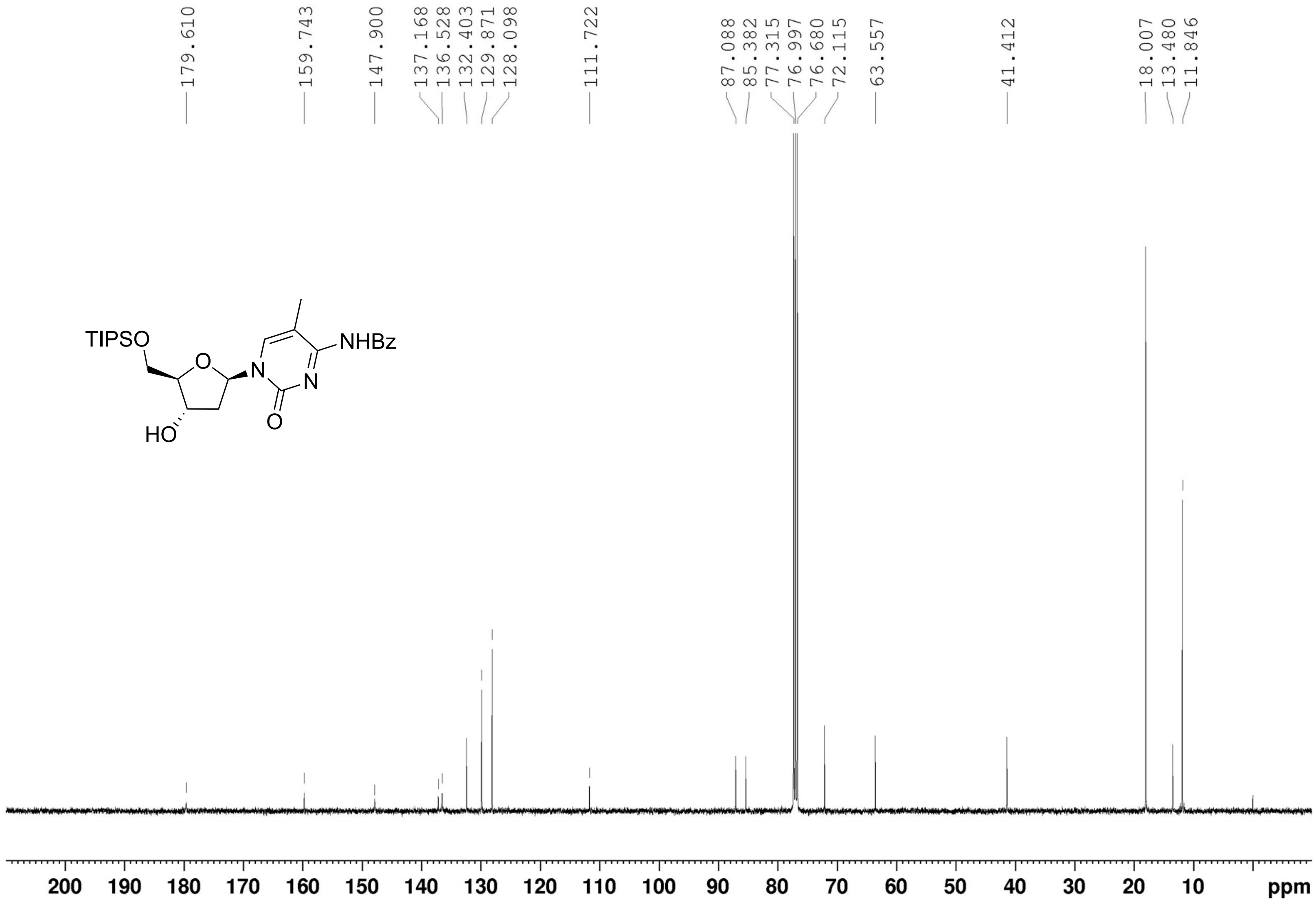


$^{13}$C NMR (100.6 MHz, $CDCl_3$) spectrum of ***N*-Benzoyl-5'-*O*-TIPS-5-methyl-2'-deoxycytidine**

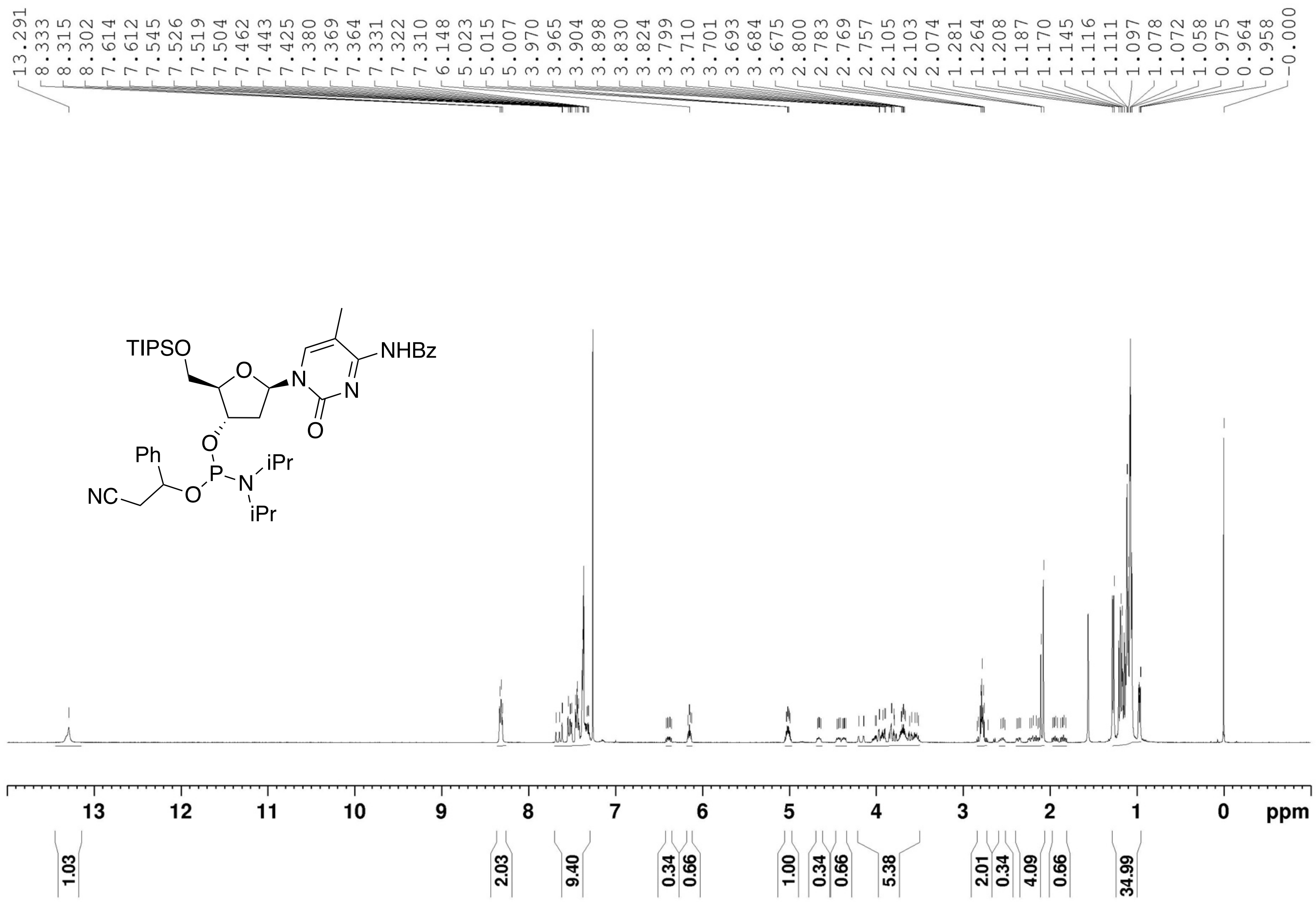


$^{1}$H NMR (400 MHz, $CDCl_3$) spectrum of ***N*-Benzoyl-5'-*O*-TIPS-5-methyl-2'-deoxycytidine Phosphoramidite**

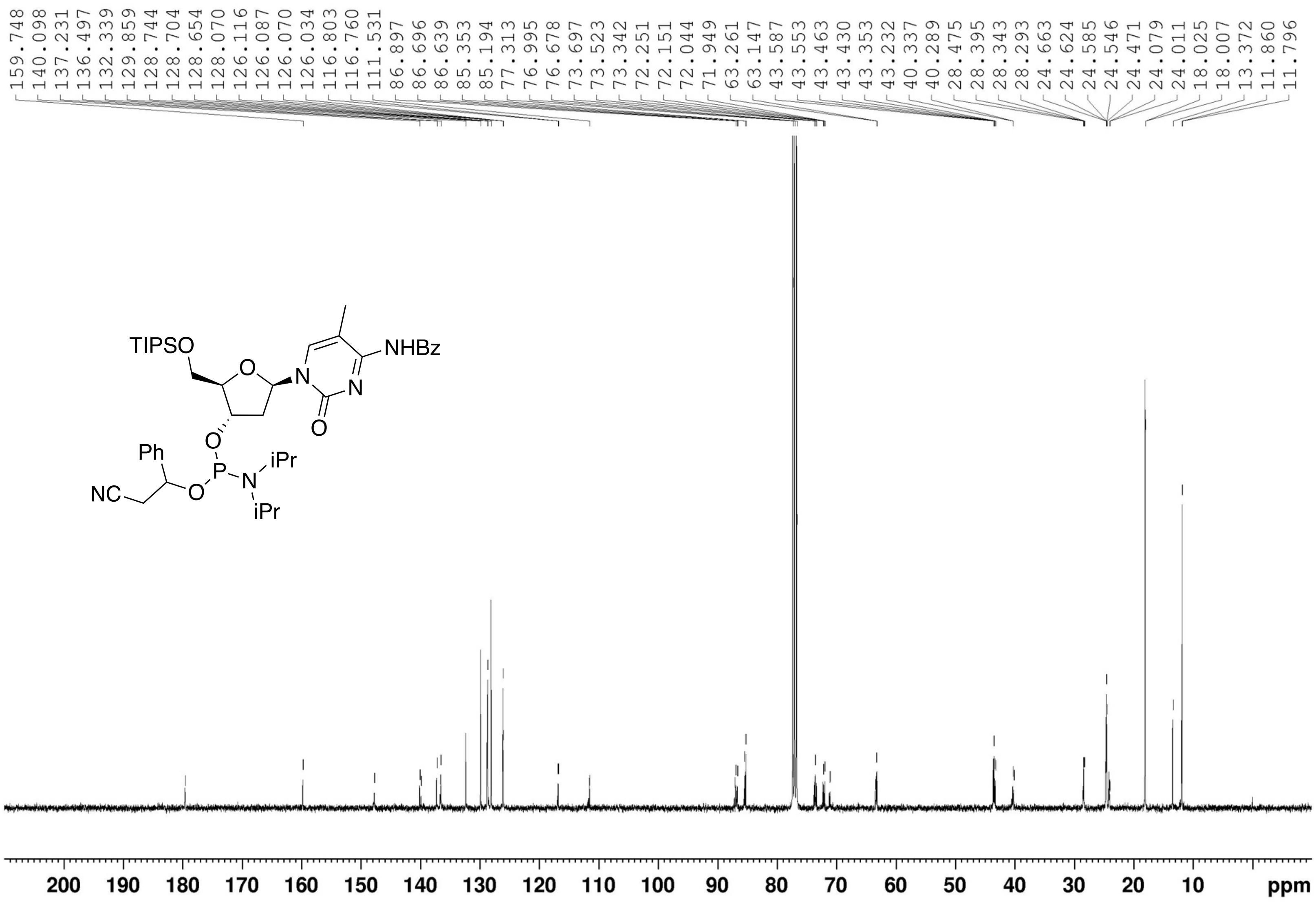


$^{13}$C NMR (100.6 MHz, $CDCl_3$) spectrum of ***N*-Benzoyl-5'-*O*-TIPS-5-methyl-2'-deoxycytidine Phosphoramidite**

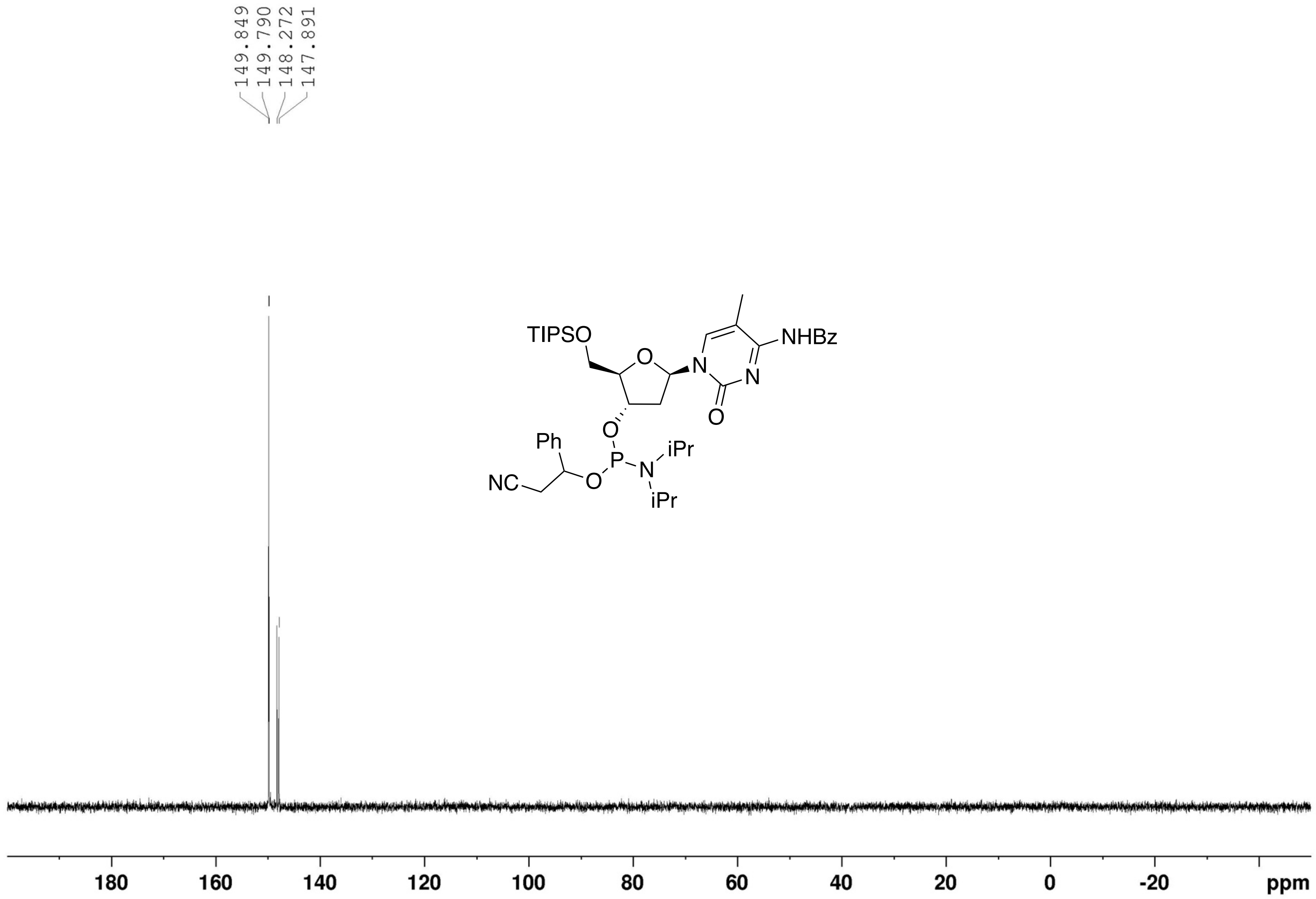


$^{31}$P NMR (162 MHz, $CDCl_3$) spectrum of ***N*-Benzoyl-5’-*O*-TIPS-5-methyl-2’-deoxycytidine Phosphoramidite**

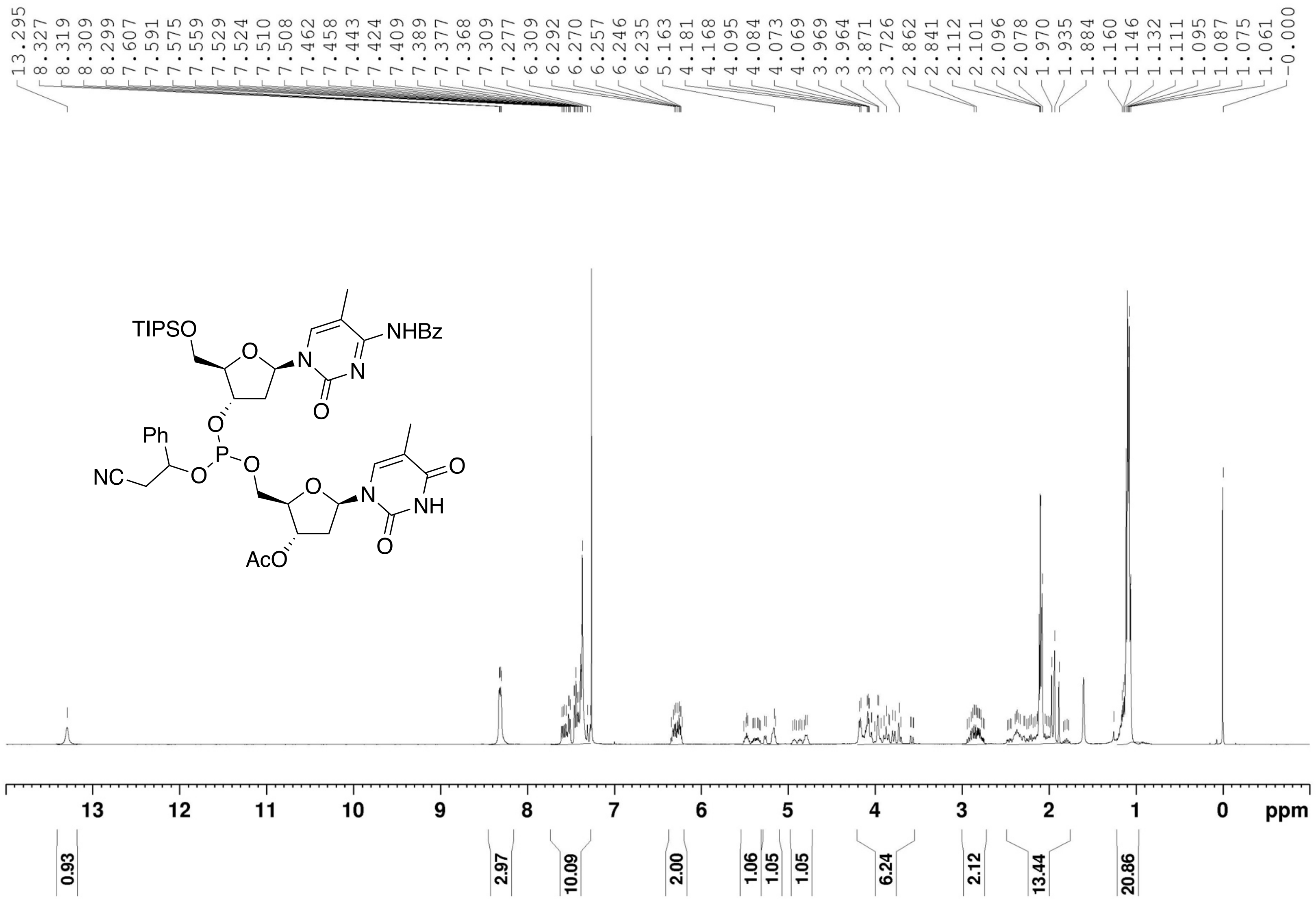


$^{1}H$ NMR (400 MHz, $CDCl_3$) spectrum of **1b**

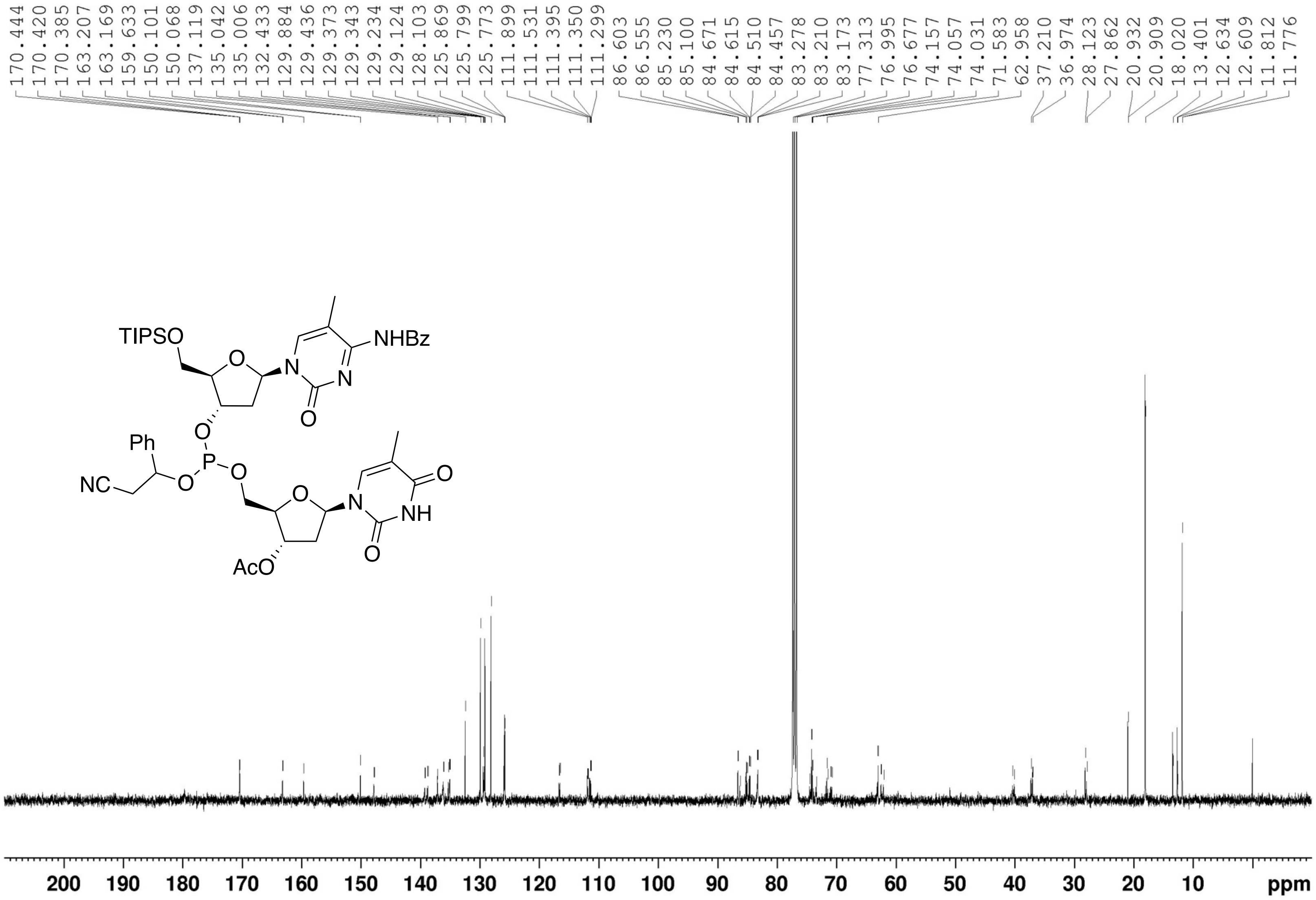


$^{13}C$ NMR (100.6 MHz, $CDCl_3$) spectrum of **1b**

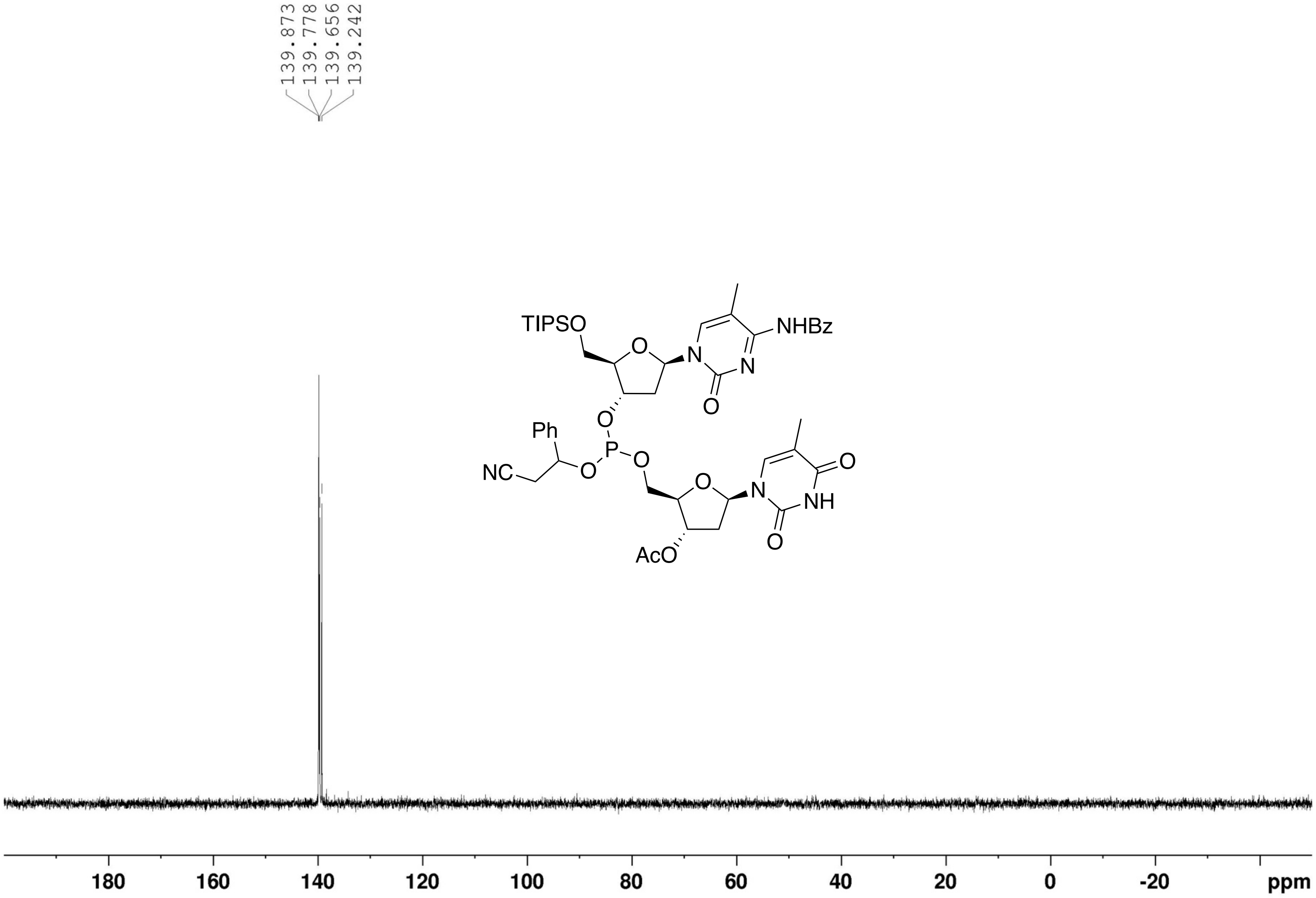


$^{31}P$ NMR (162 MHz, $CDCl_3$) spectrum of **1b**

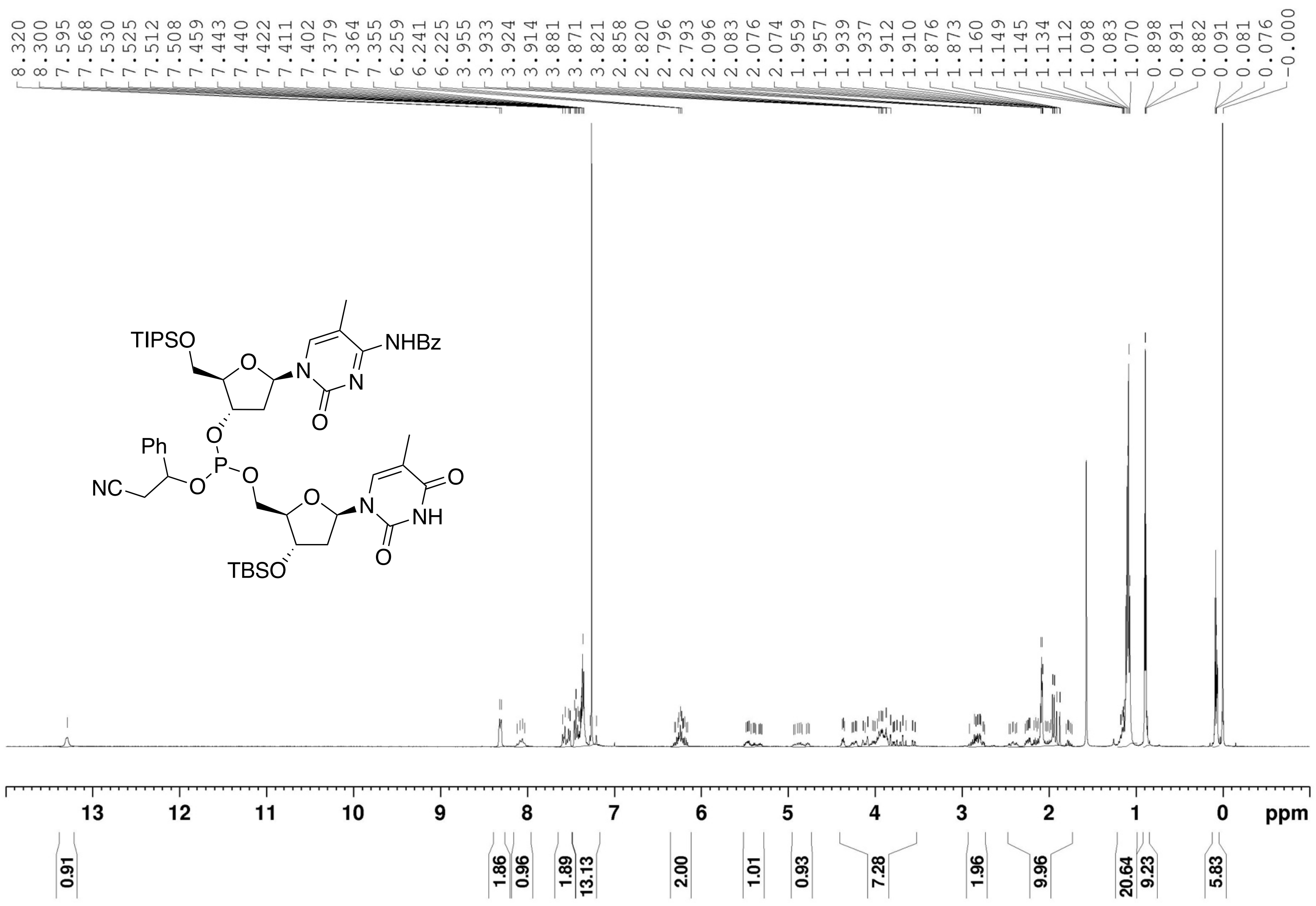

$^{1}$H NMR (400 MHz, $CDCl_3$) spectrum of **1c**

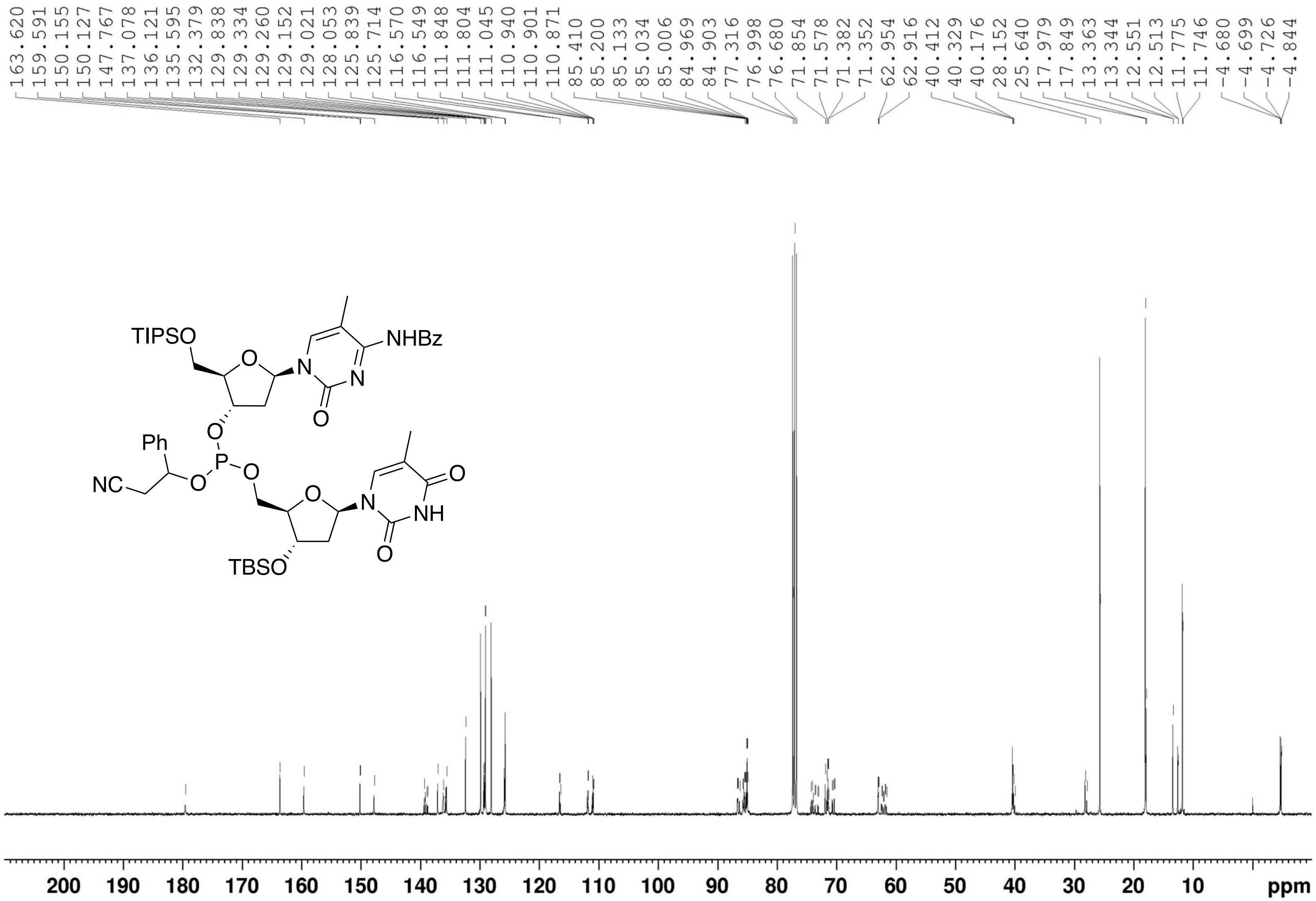


$^{13}$C NMR (100.6 MHz, $CDCl_3$) spectrum of **1c**

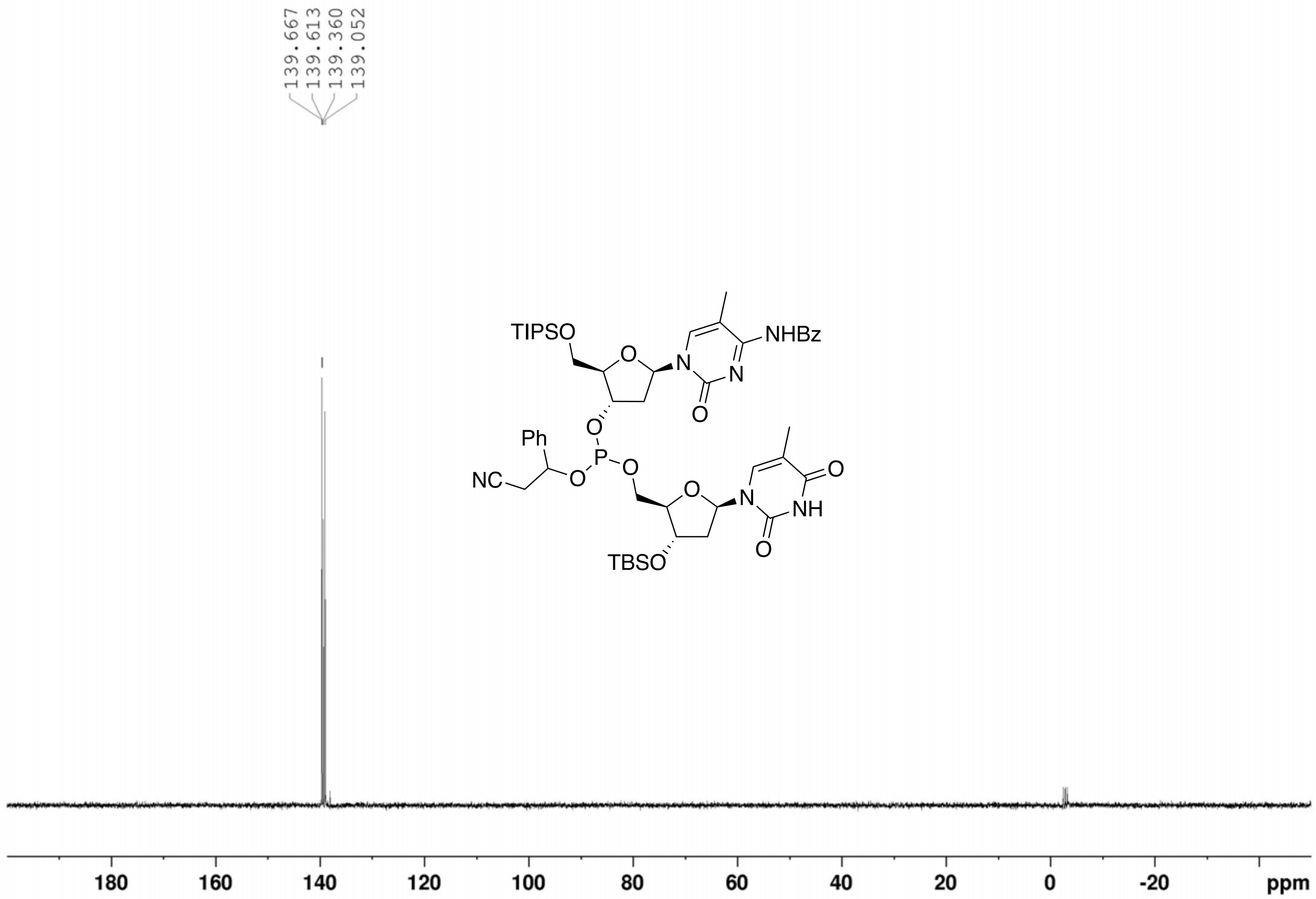


$^{31}$P NMR (162 MHz, $CDCl_3$) spectrum of **1c**

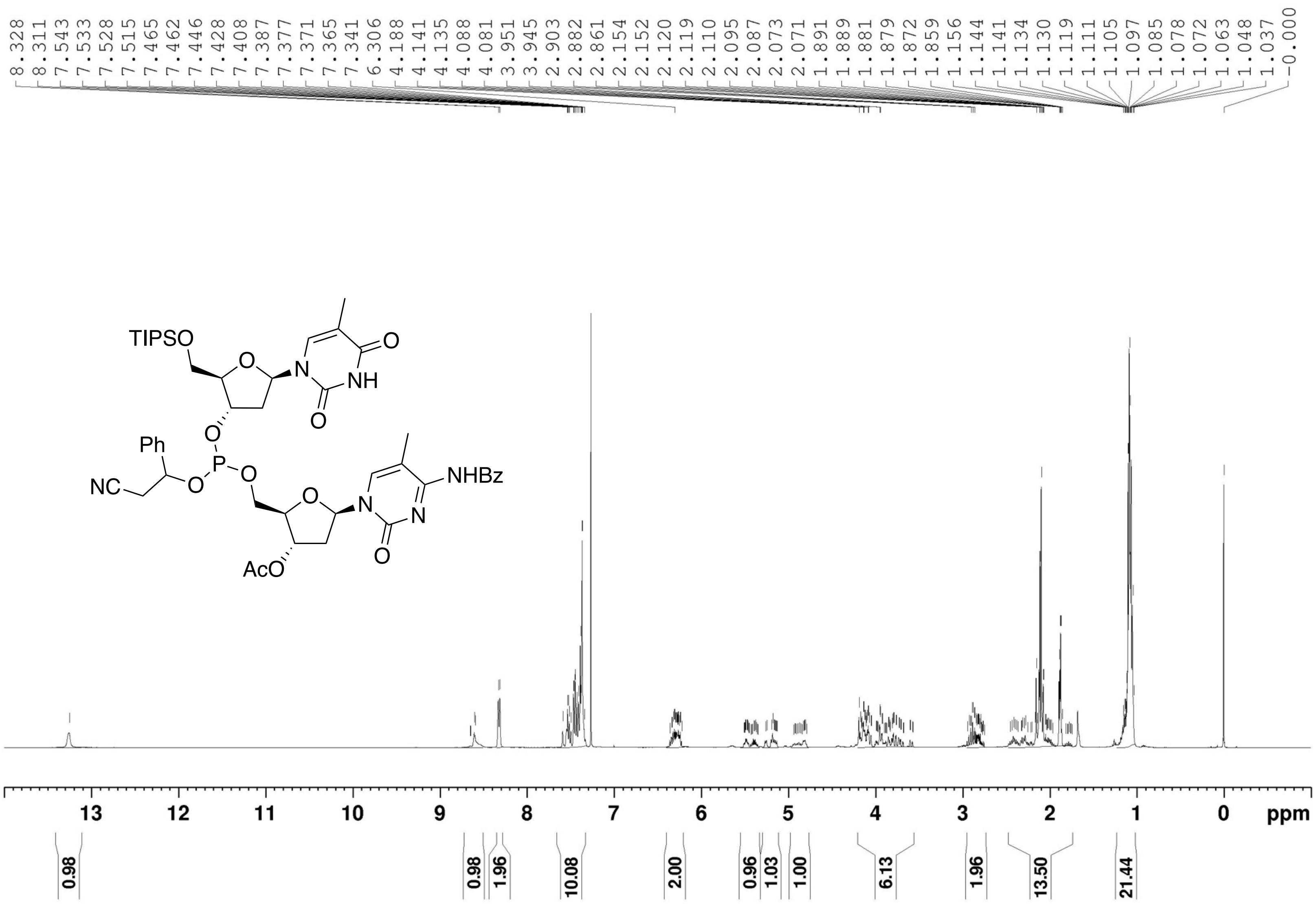

$^{1}$H NMR (400 MHz, $CDCl_3$) spectrum of **1d**

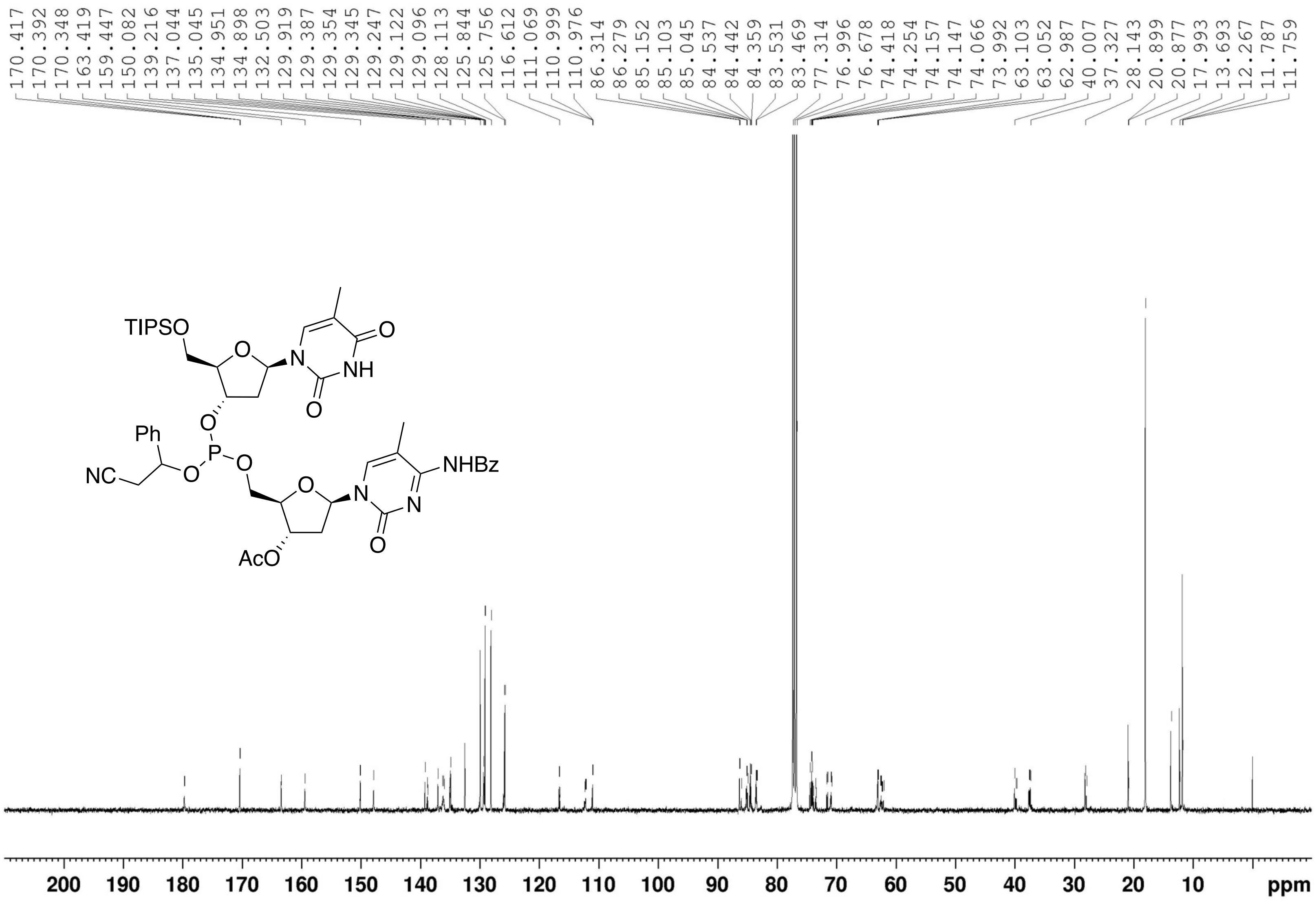


$^{13}C$ NMR (100.6 MHz, $CDCl_3$) spectrum of **1d**

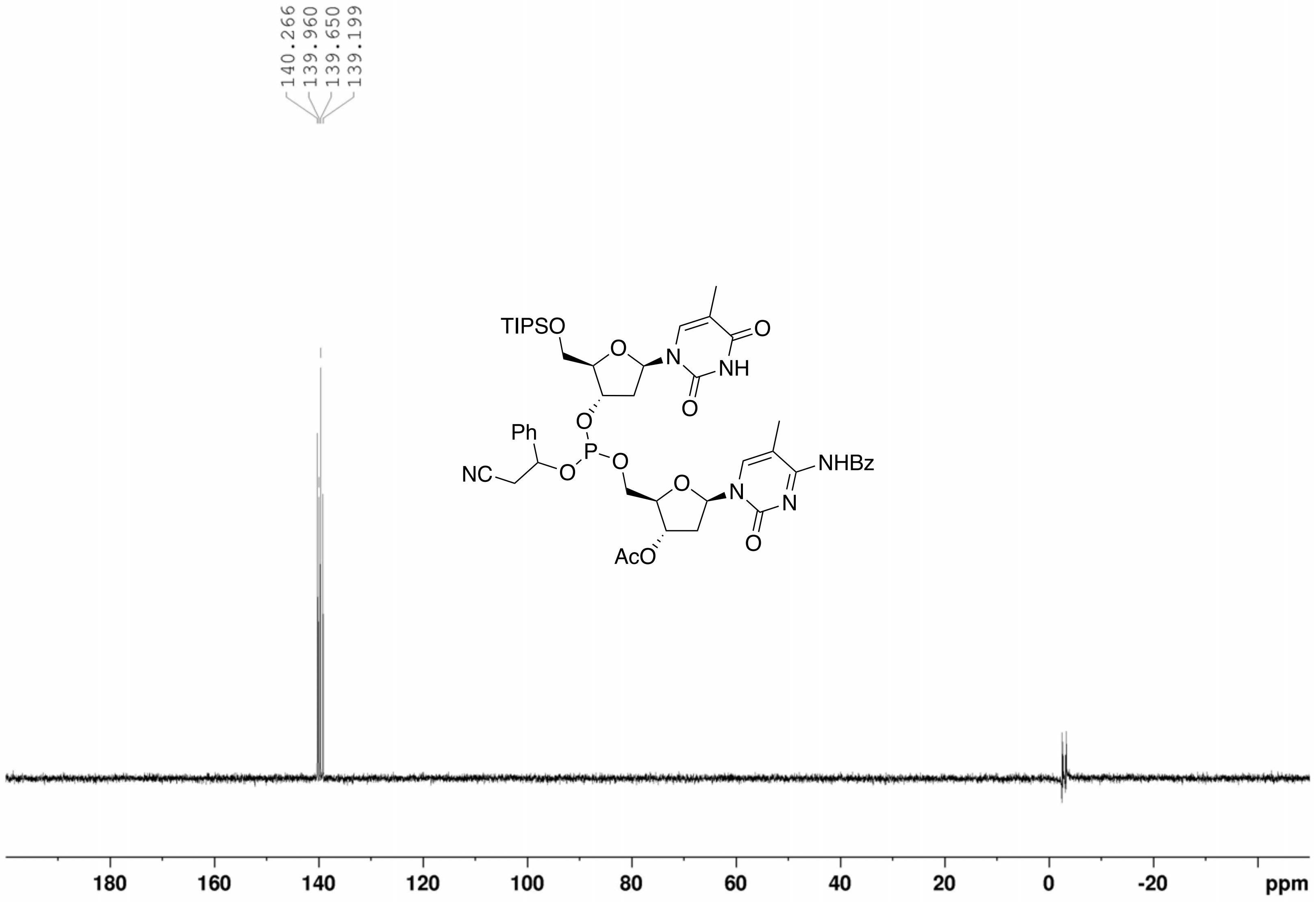


$^{31}$P NMR (162 MHz, $CDCl_3$) spectrum of **1d**

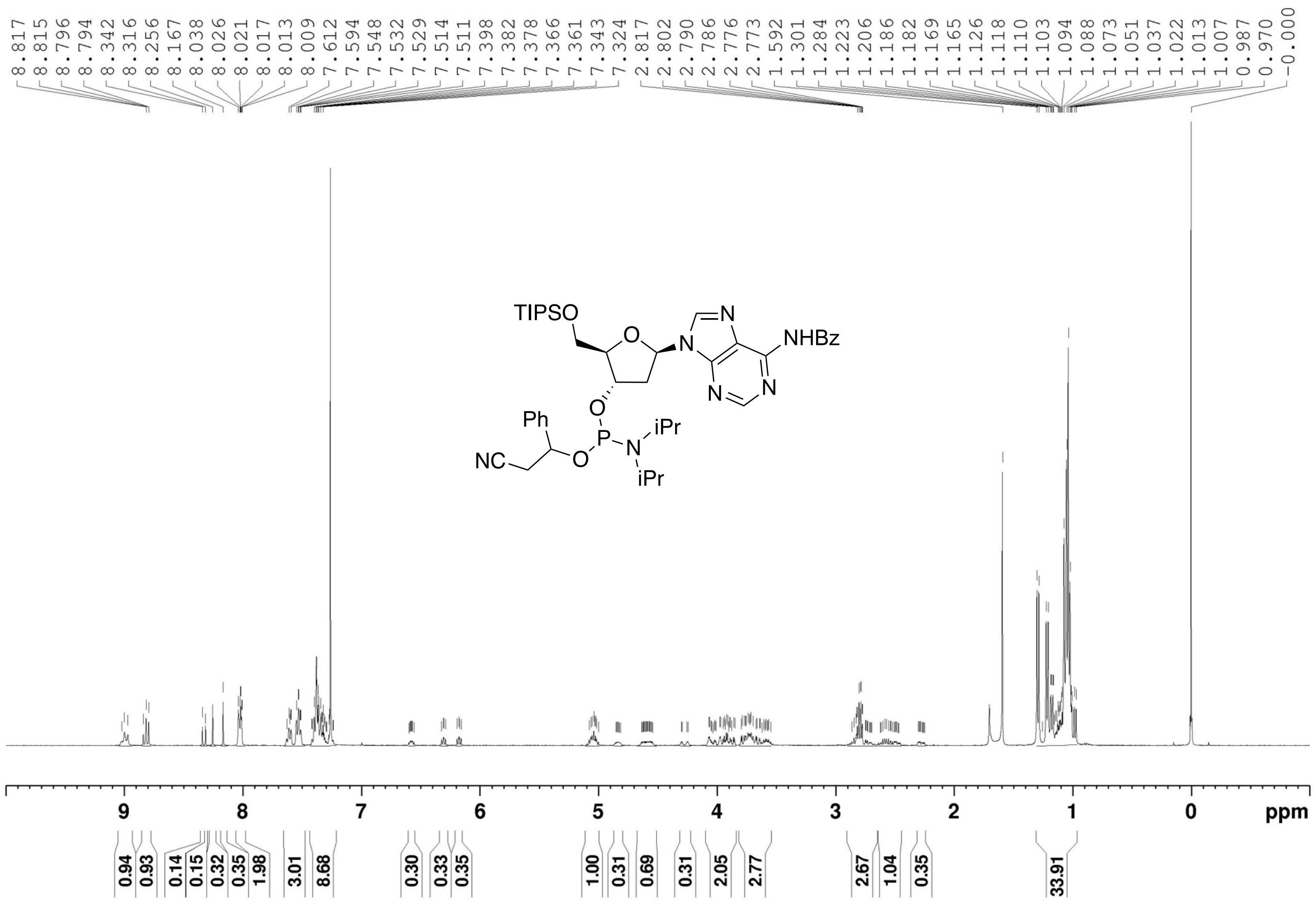


$^1$H NMR (400 MHz, $CDCl_3$) spectrum of ***N*$^6$-Benzoyl-5'-*O*-TIPS-2'-deoxyadenosine Phosphoramidite**

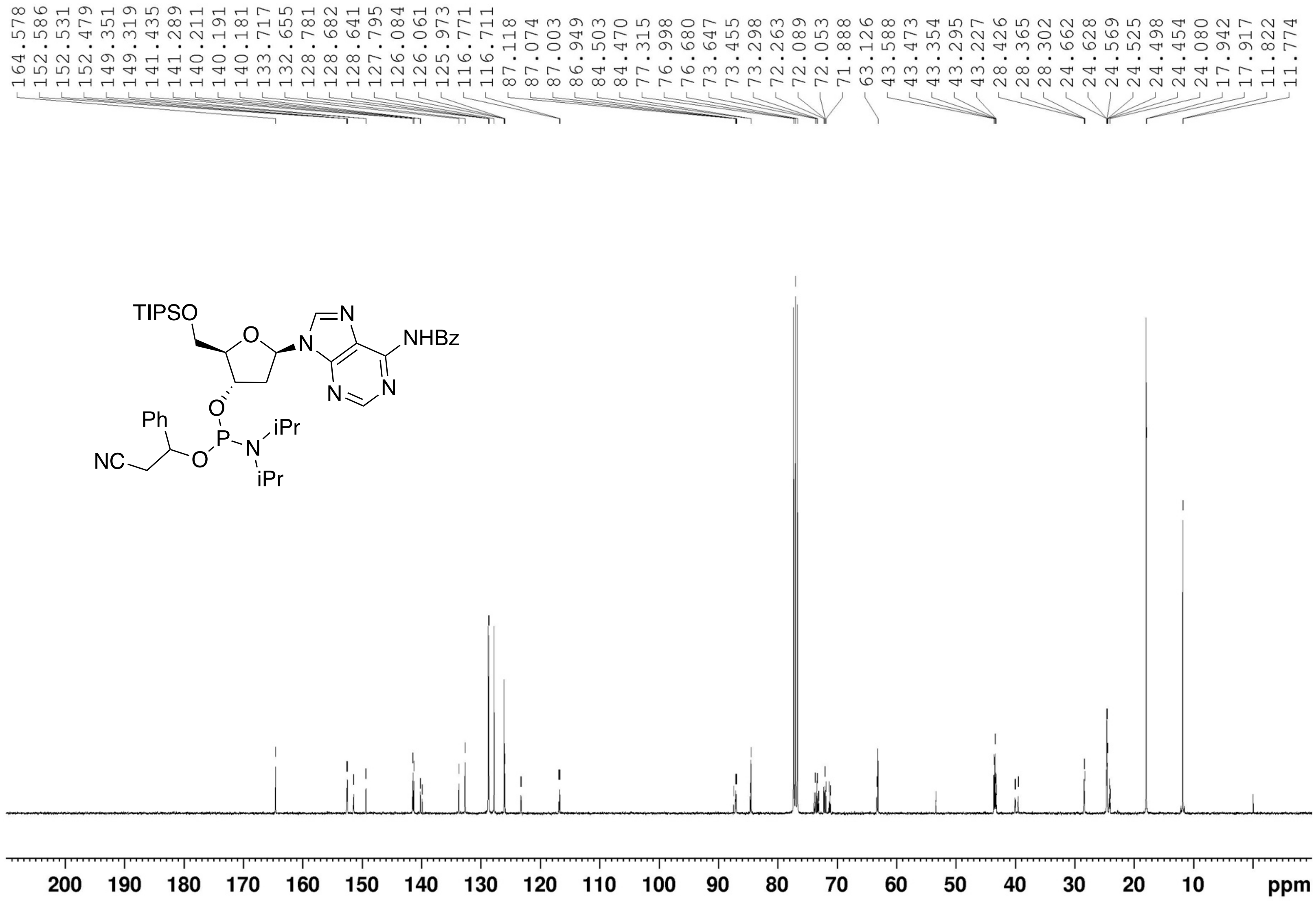


$^{13}$C NMR (100.6 MHz, $CDCl_3$) spectrum of ***N*$^{6}$-Benzoyl-5'-*O*-TIPS-2'-deoxyadenosine Phosphoramidite**

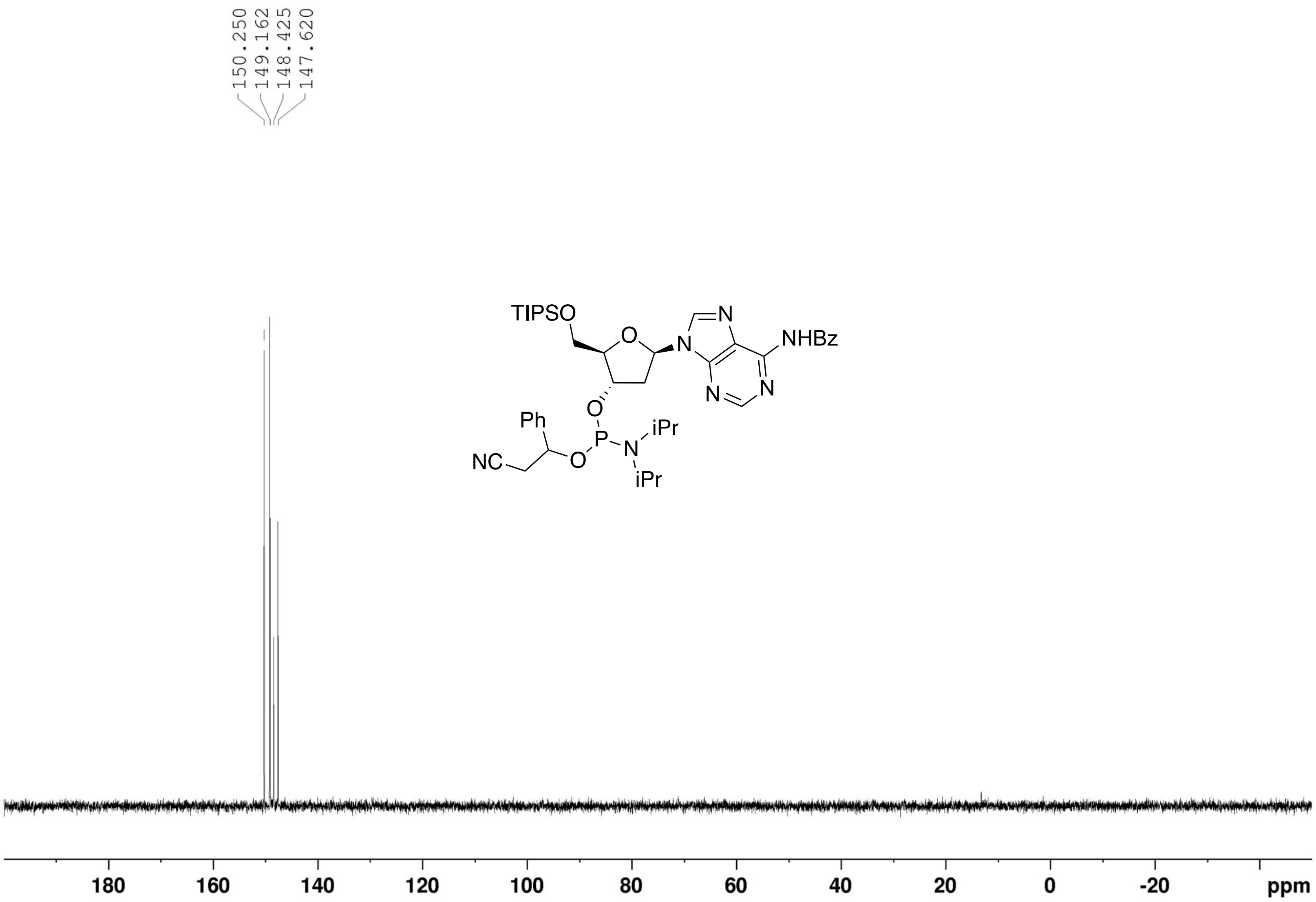


$^{31}P$ NMR (162 MHz, $CDCl_3$) spectrum of ***N*[6]-Benzoyl-5'-*O*-TIPS-2'-deoxyadenosine Phosphoramidite**

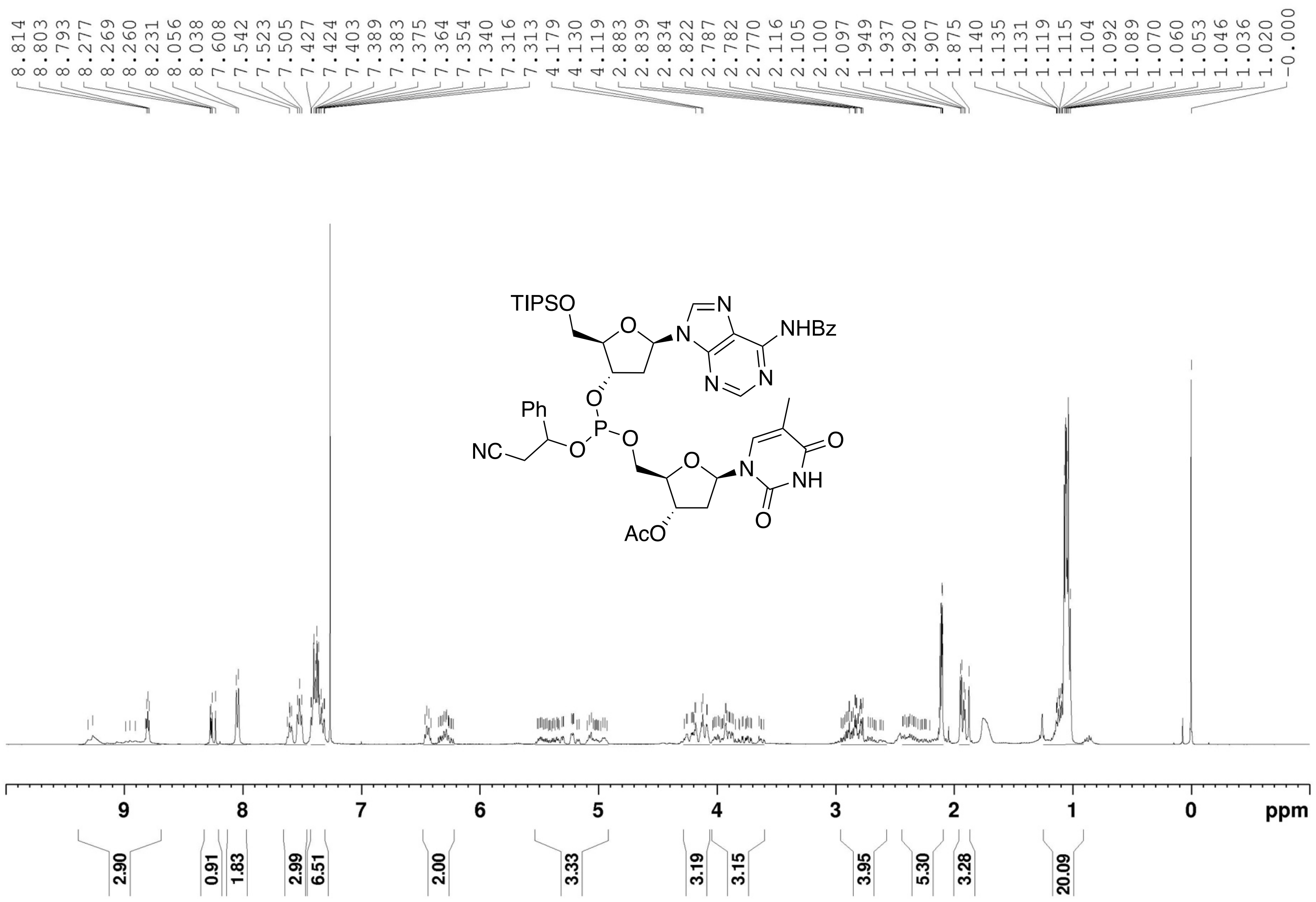

$^{1}H$ NMR (400 MHz, $CDCl_3$) spectrum of **1e**

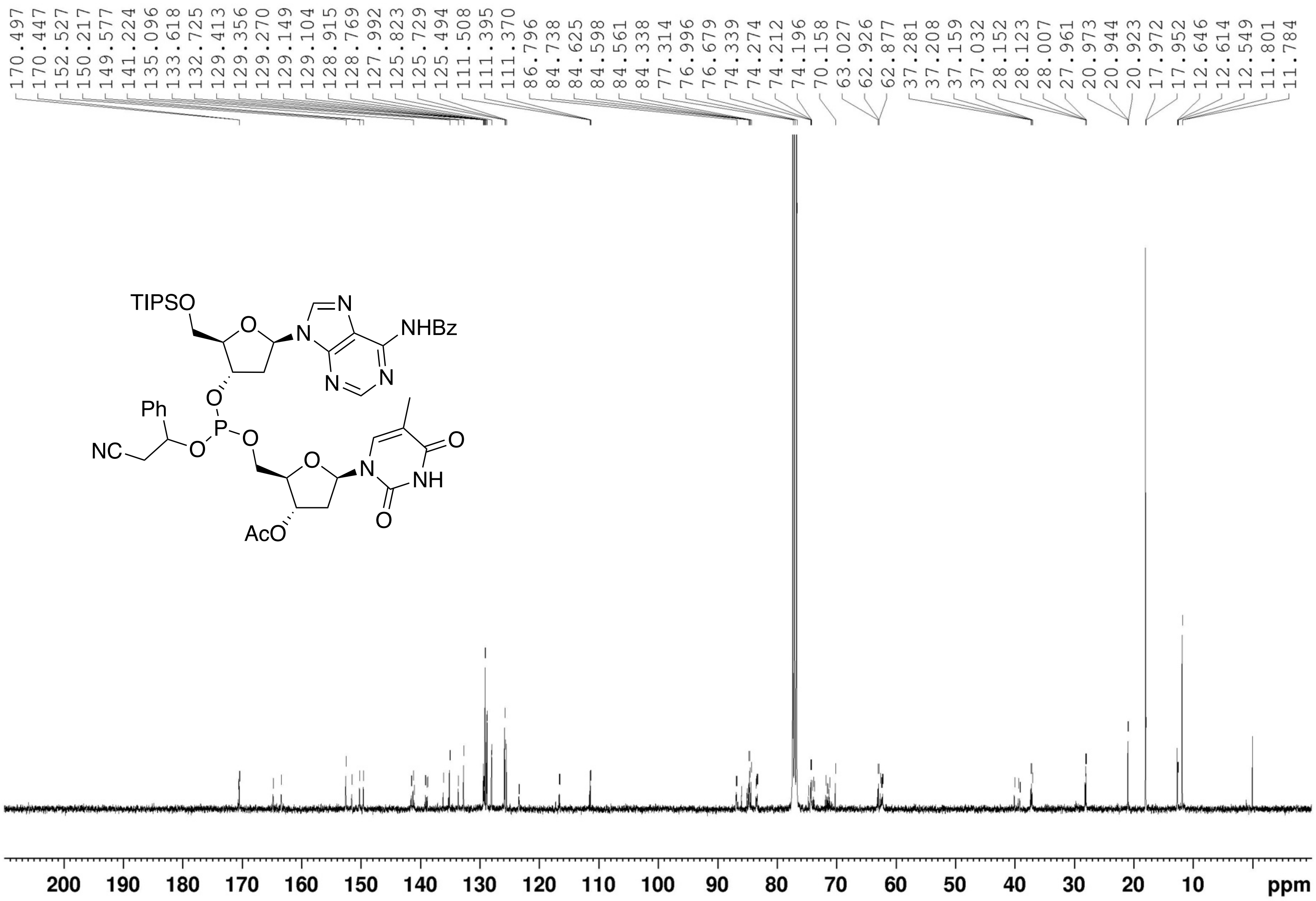


$^{13}C$ NMR (100.6 MHz, $CDCl_3$) spectrum of **1e**

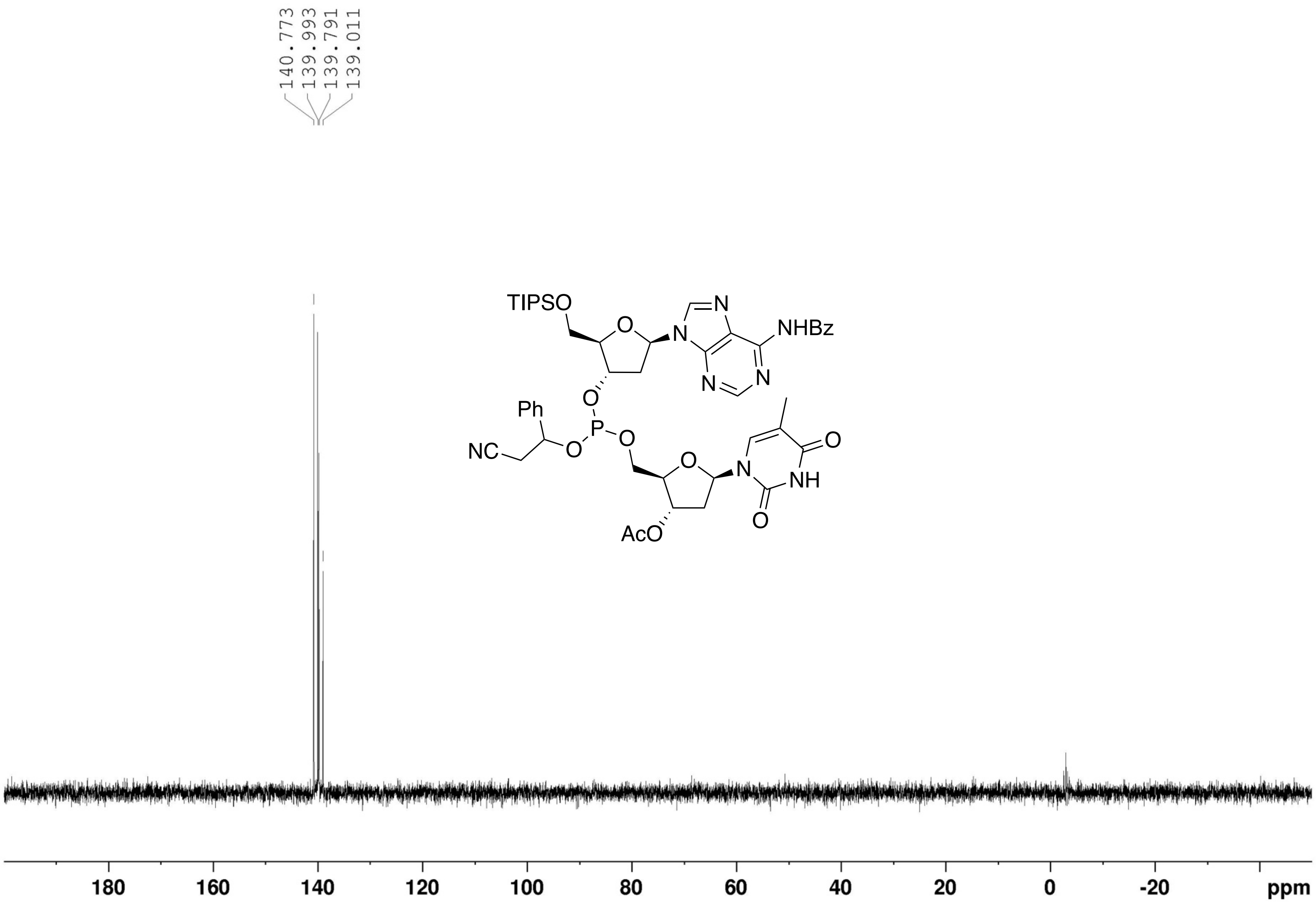


$^{31}$P NMR (162 MHz, $CDCl_3$) spectrum of **1e**

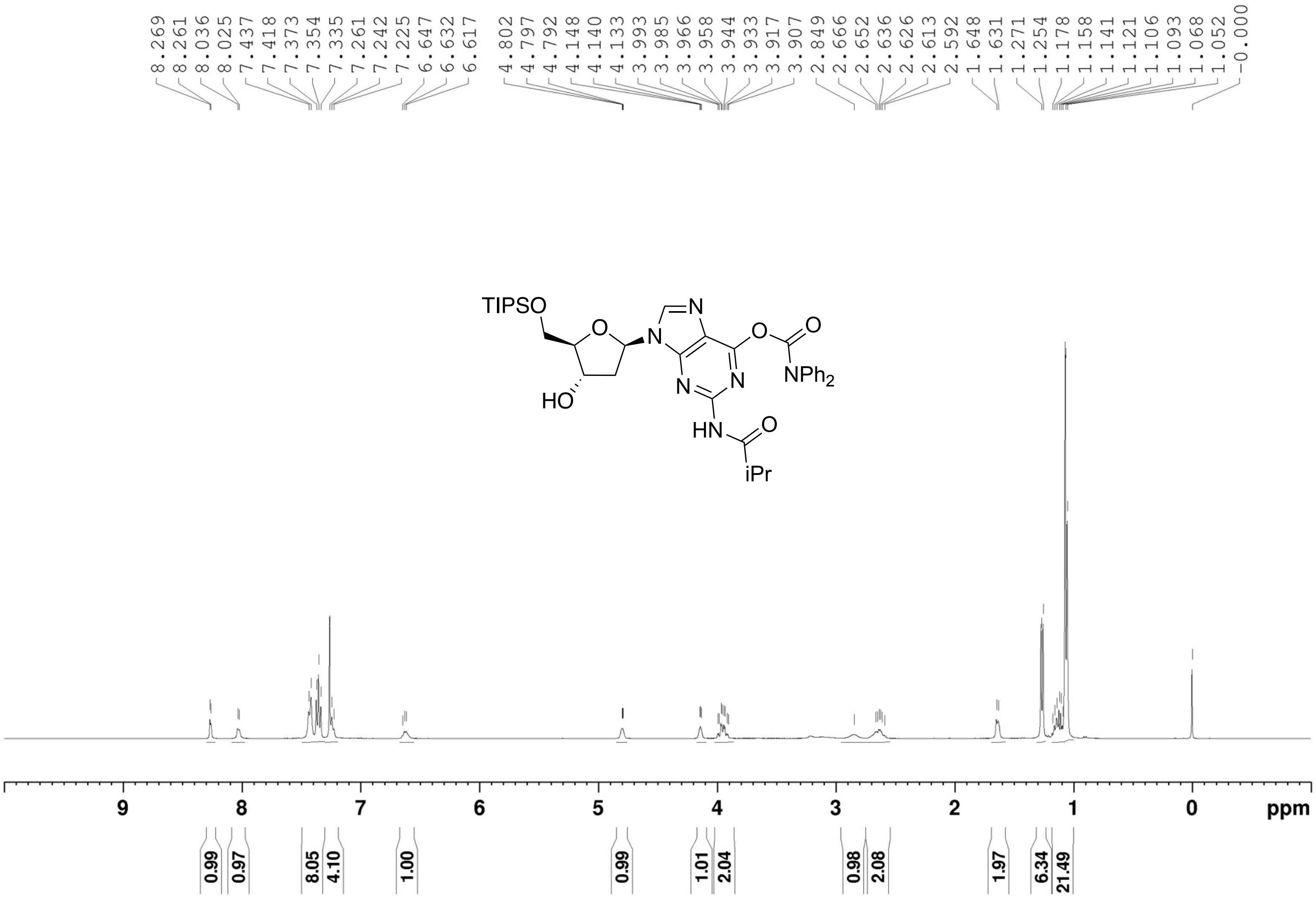


$^{1}$H NMR (400 MHz, $CDCl_3$) spectrum of ***N*²-Isobutyryl-*O*⁶-(diphenylcarbamoyl)-5'-*O*-TIPS-2'-deoxyguanosine**

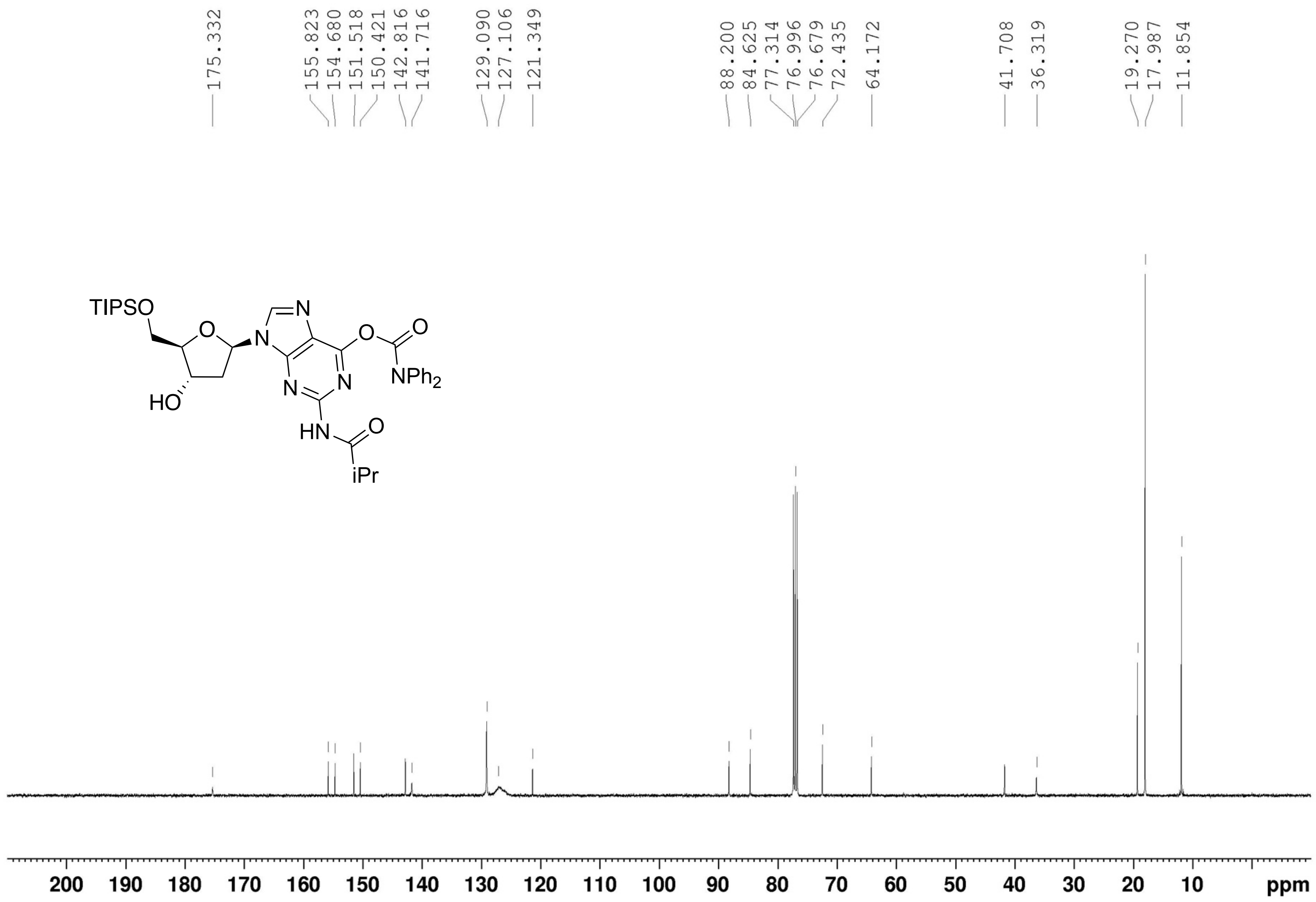


$^{13}$C NMR (100.6 MHz, $CDCl_3$) spectrum of ***N*²-Isobutyryl-*O*⁶-(diphenylcarbamoyl)-5'-*O*-TIPS-2'-deoxyguanosine**

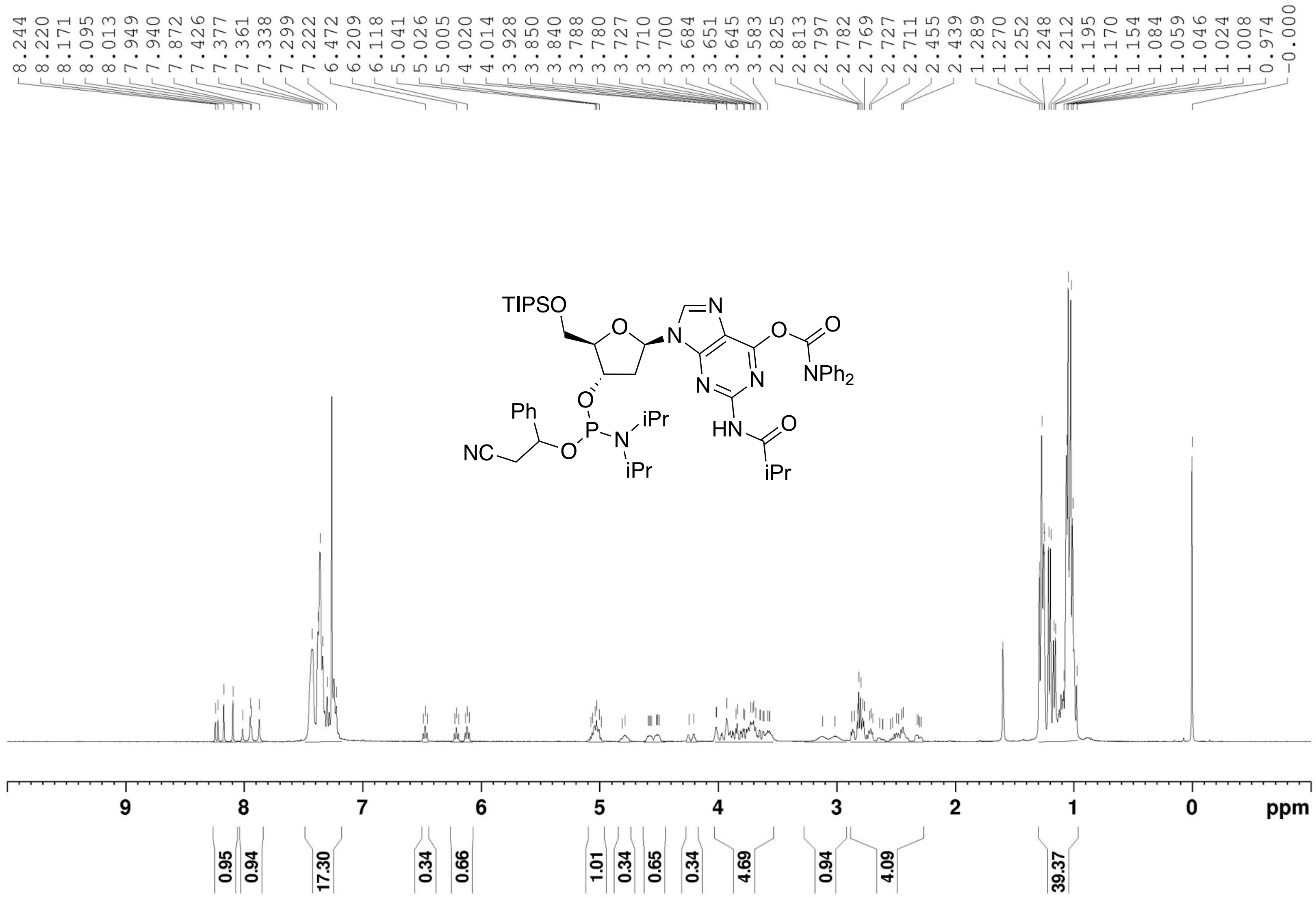

$^{1}$H NMR (400 MHz, CDCl₃) spectrum of ***N*²-Isobutyryl-*O*⁶-(diphenylcarbamoyl)-5'-*O*-TIPS-2'-deoxyguanosine Phosphoramidite**

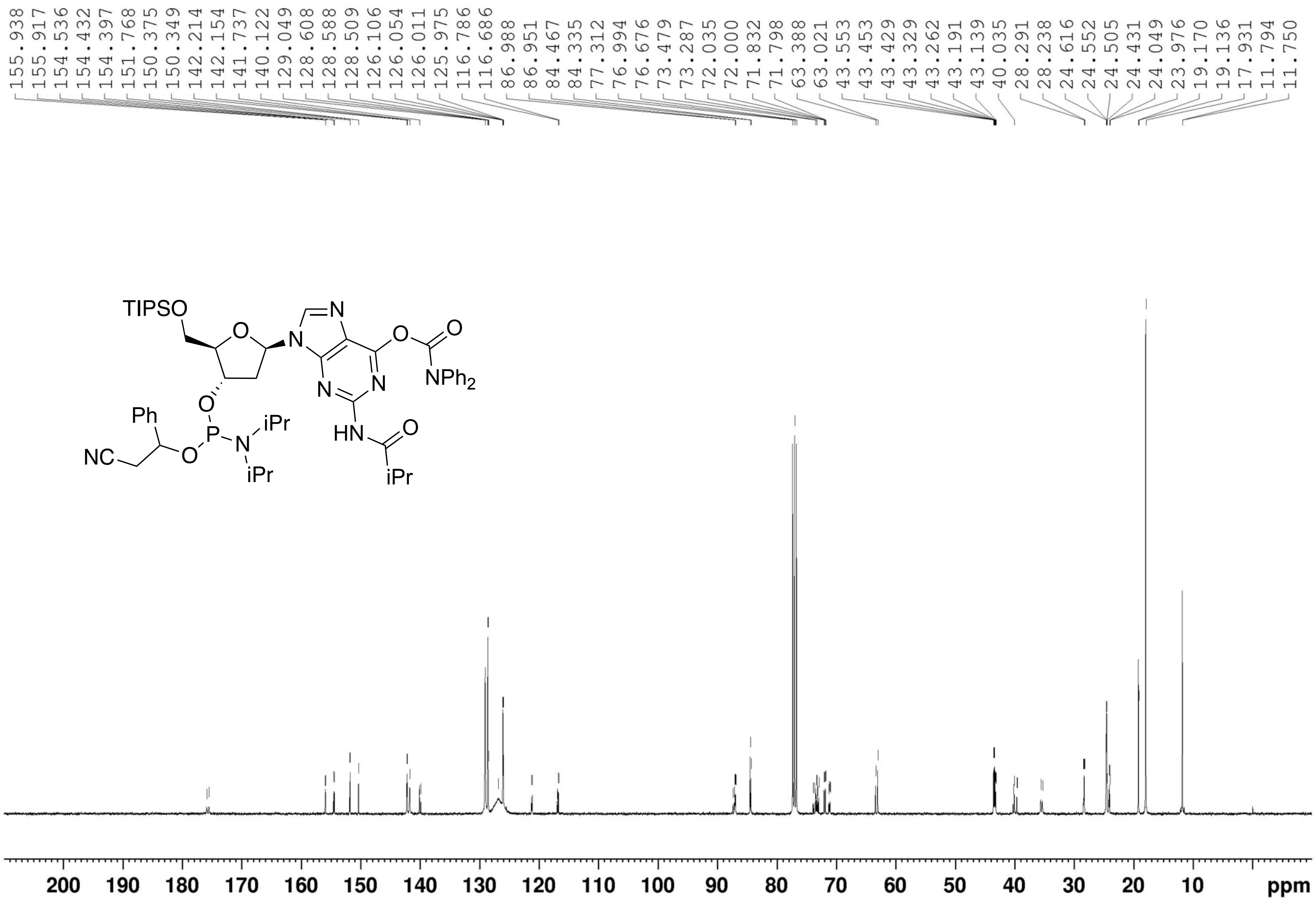


$^{13}C$ NMR (100.6 MHz, $CDCl_3$) spectrum of ***N*[2]-Isobutyryl-*O*[6]-(diphenylcarbamoyl)-5’-*O*-TIPS-2’-deoxyguanosine Phosphoramidite**

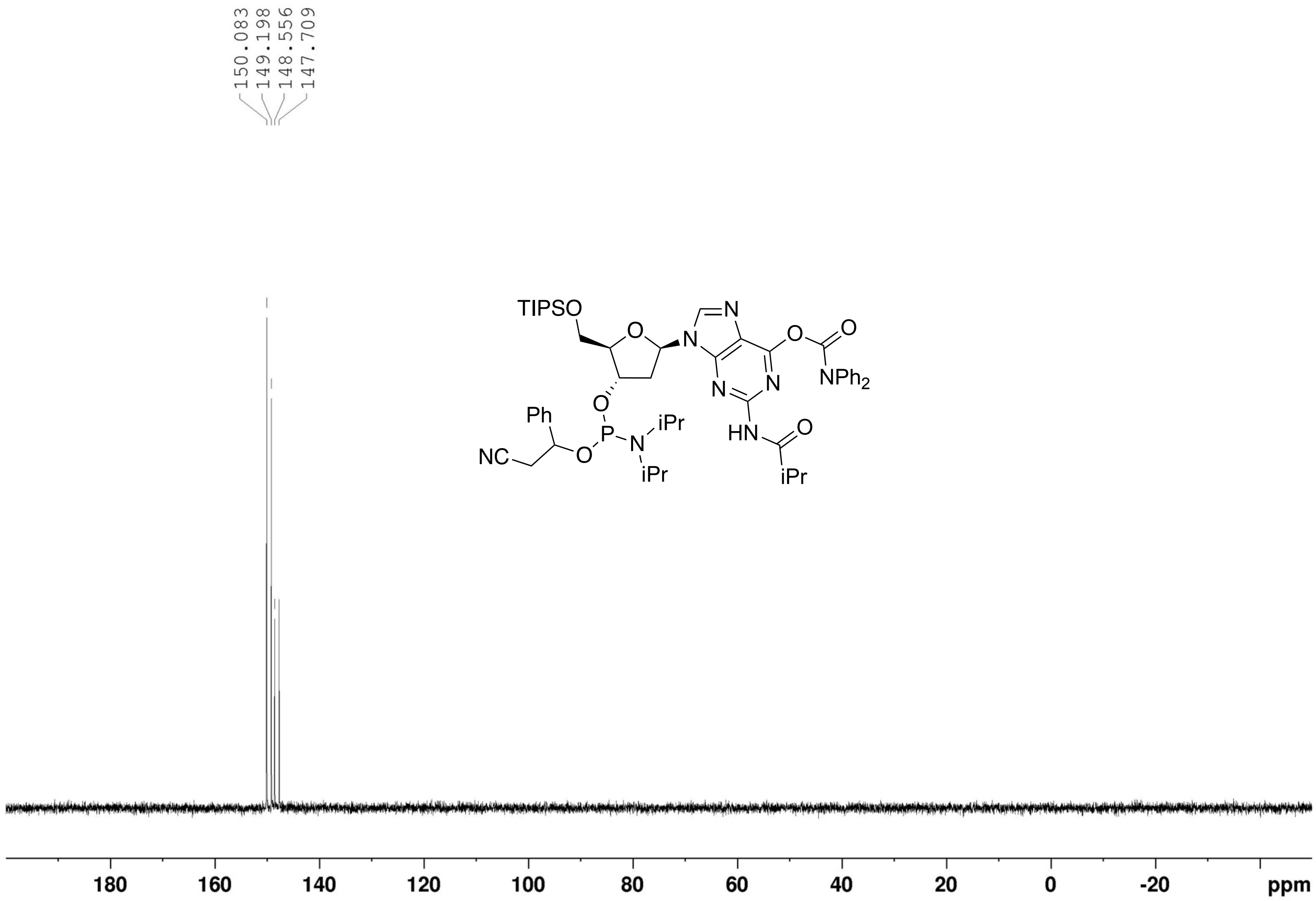


$^{31}$P NMR (162 MHz, $CDCl_3$) spectrum of ***N*^2^-Isobutyryl-*O*^6^-(diphenylcarbamoyl)-5'-*O*-TIPS-2'-deoxyguanosine Phosphoramidite**

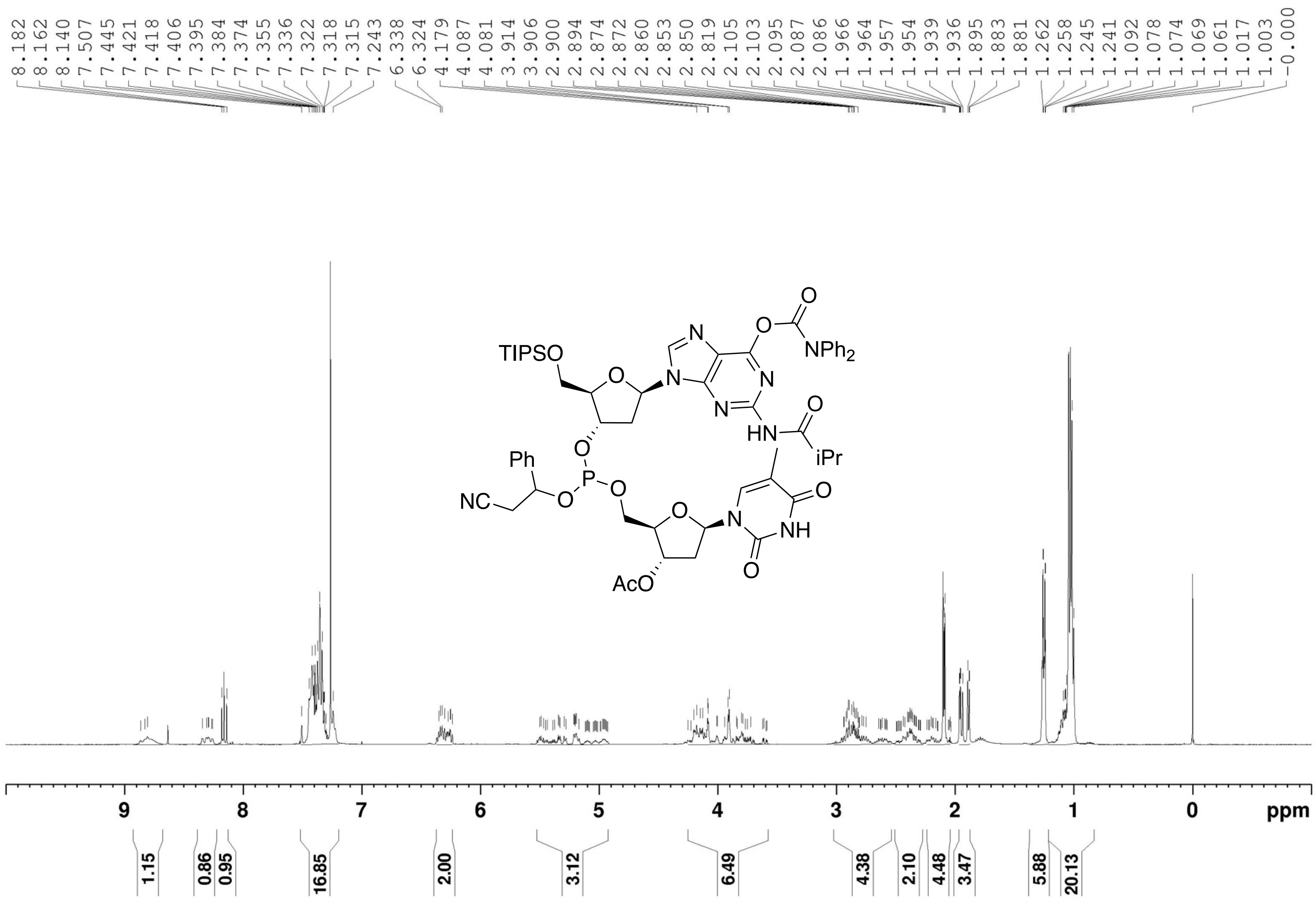


$^{1}$H NMR (400 MHz, $CDCl_3$) spectrum of **1f**

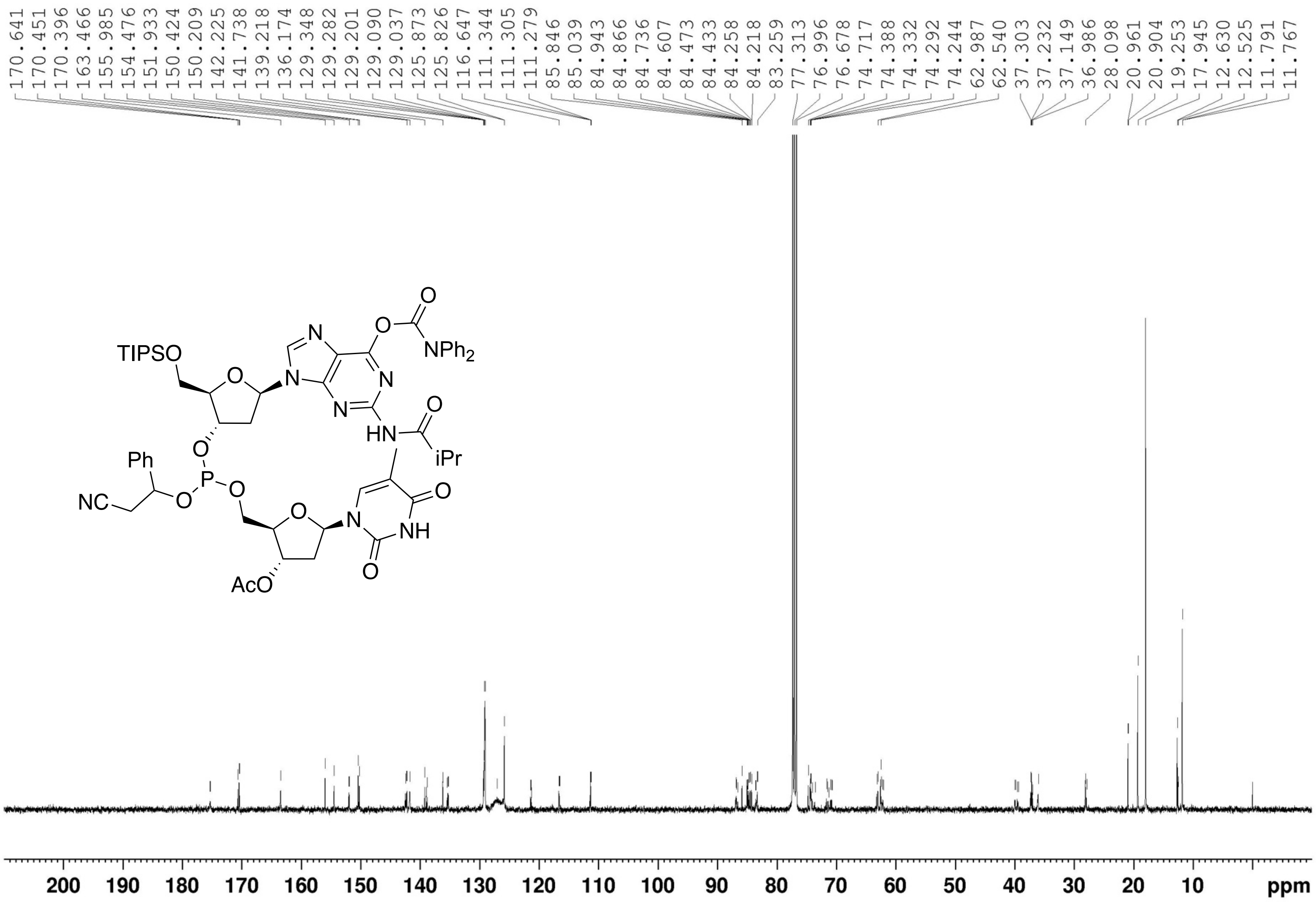


$^{13}C$ NMR (100.6 MHz, $CDCl_3$) spectrum of **1f**

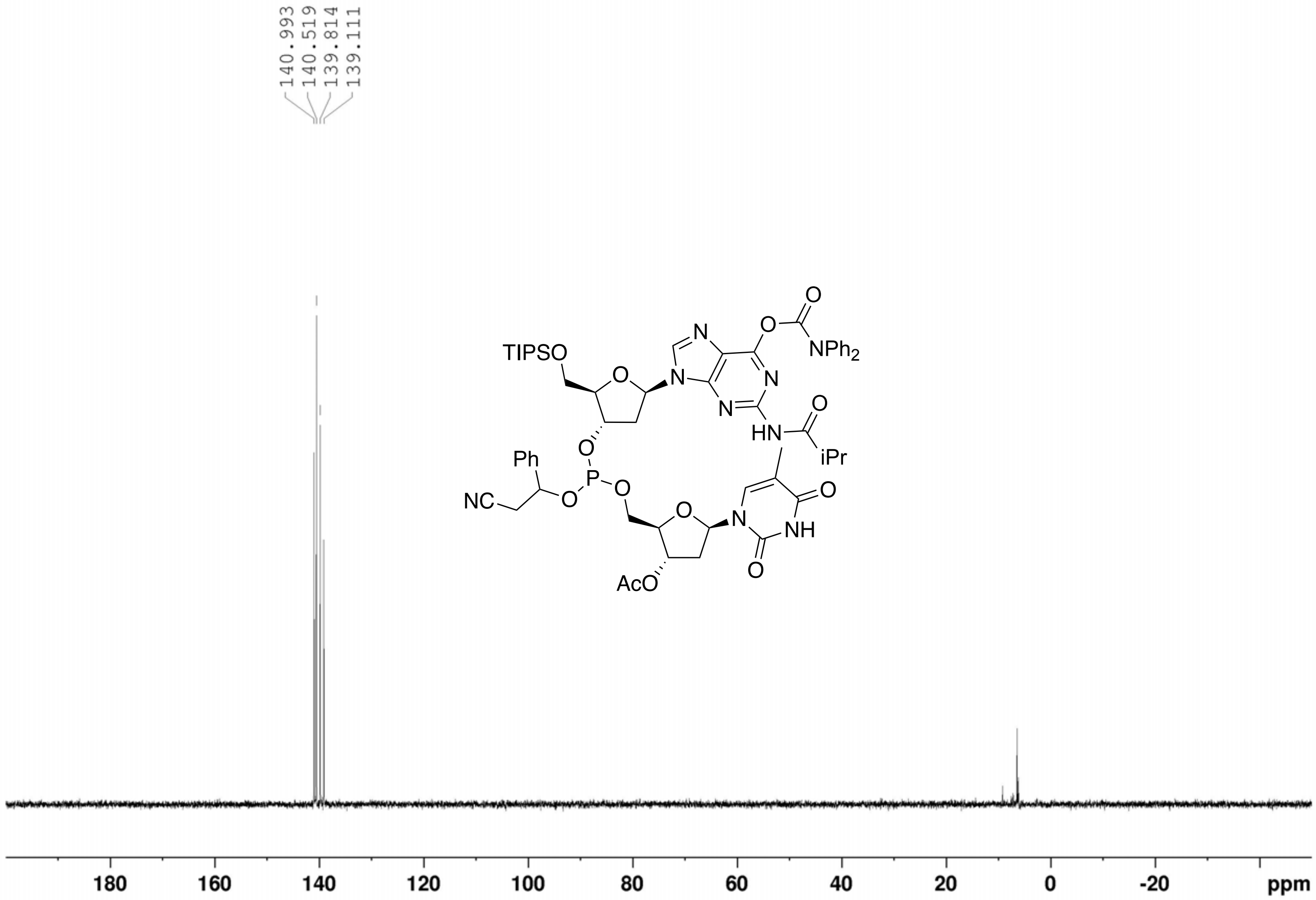


$^{31}$P NMR (162 MHz, $CDCl_3$) spectrum of **1f**

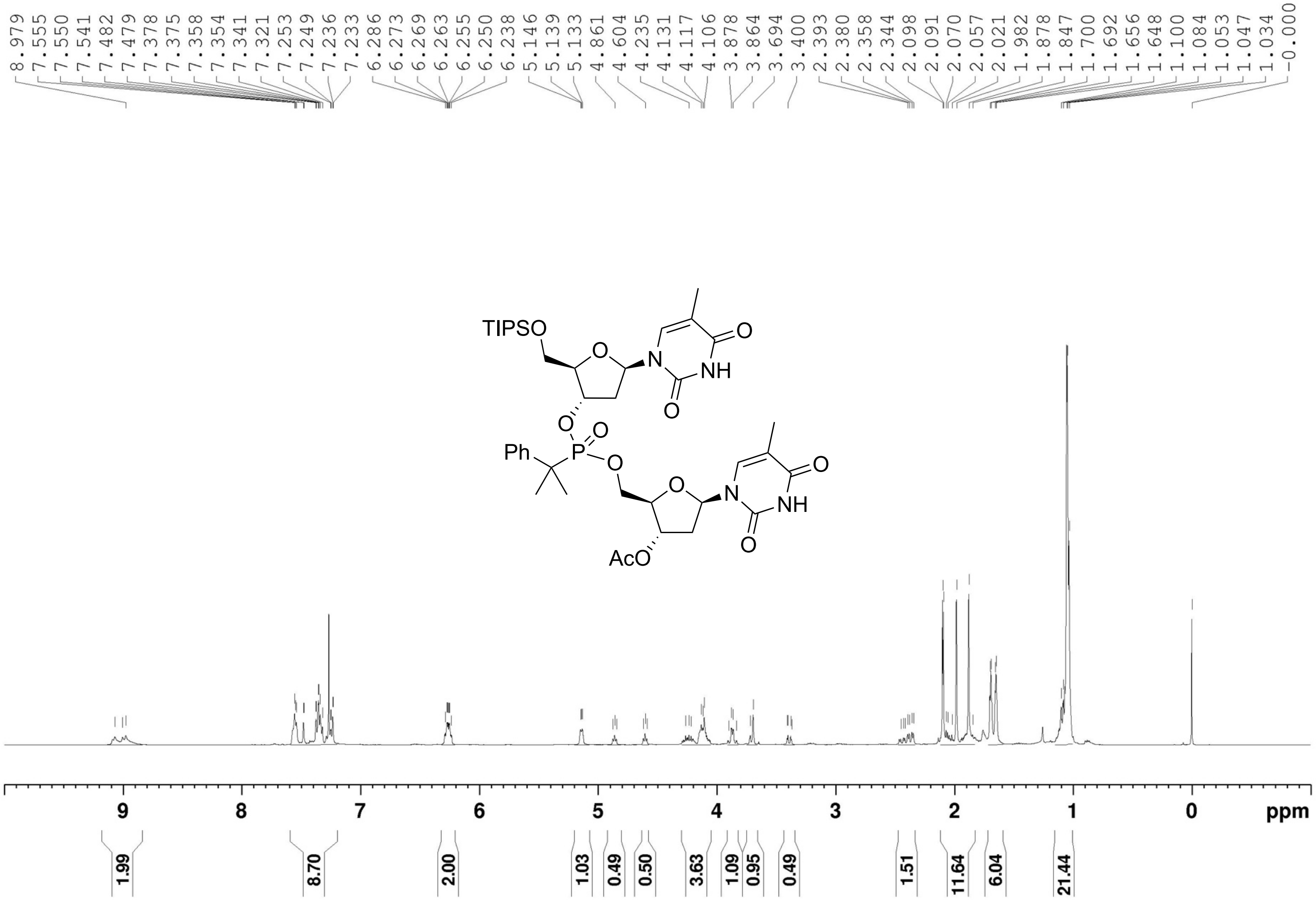


$^1$H NMR (400 MHz, $CDCl_3$) spectrum of **3aa**

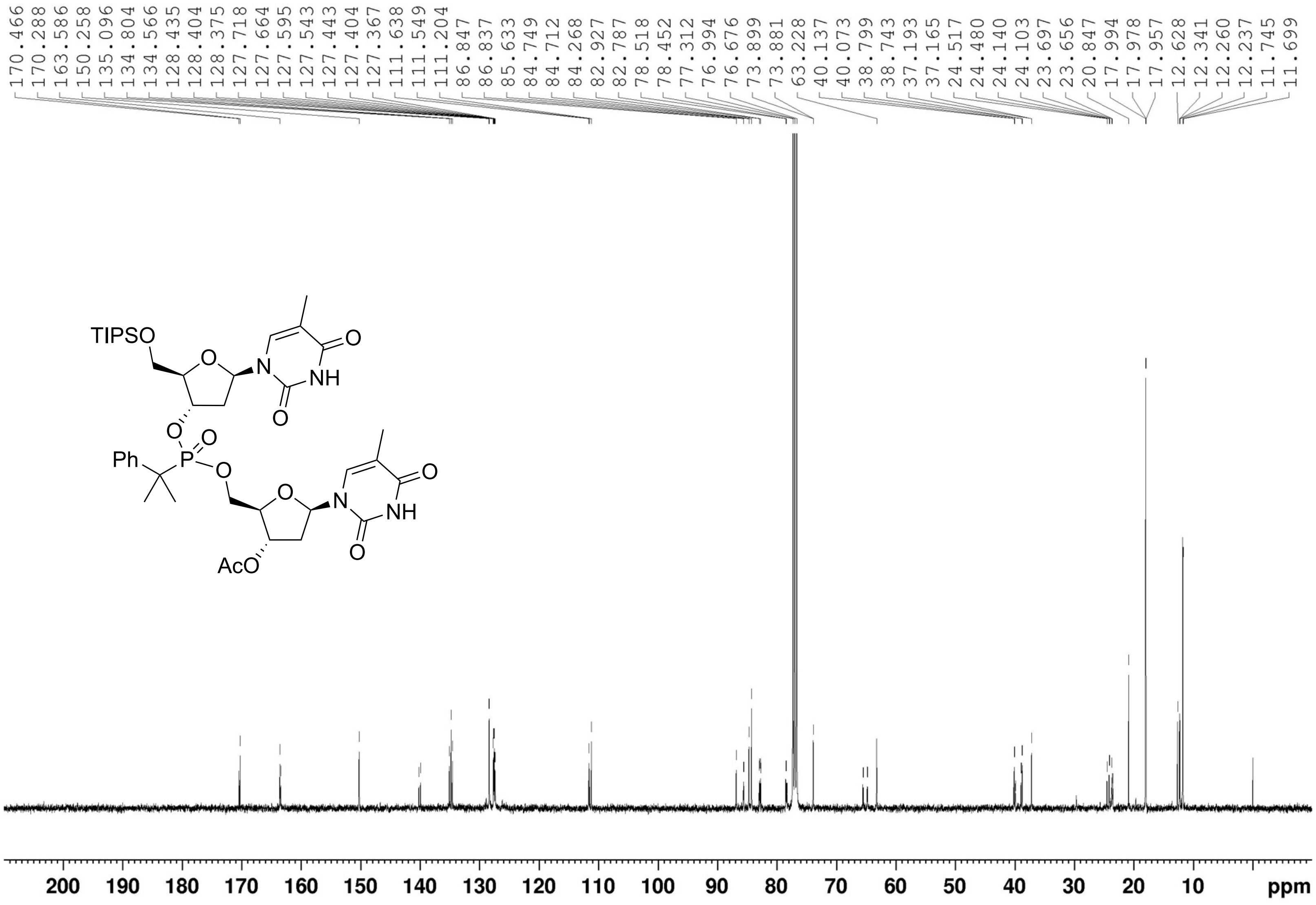


$^{13}C$ NMR (100.6 MHz, $CDCl_3$) spectrum of **3aa**

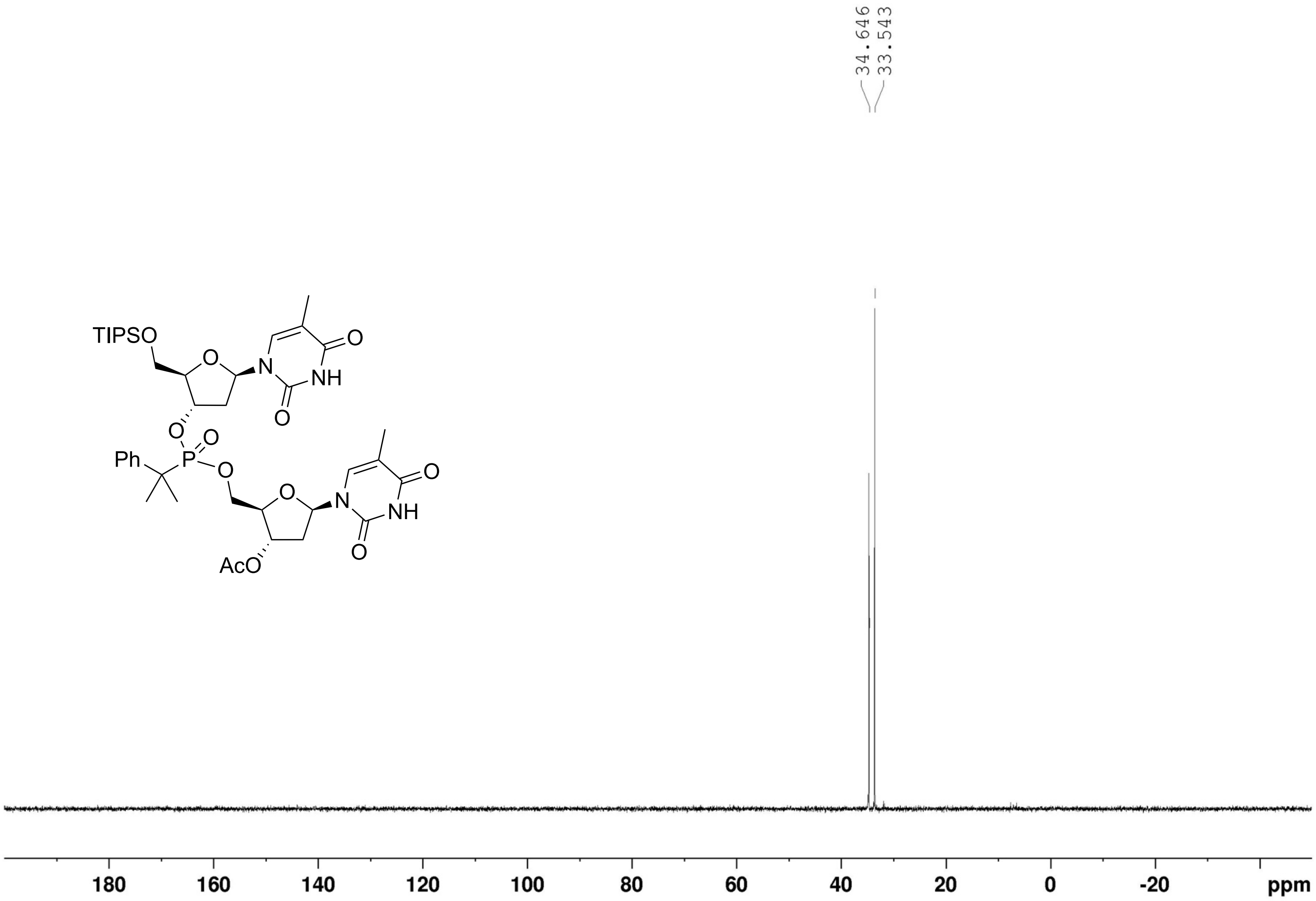


$^{31}$P NMR (162 MHz, $CDCl_3$) spectrum of **3aa**

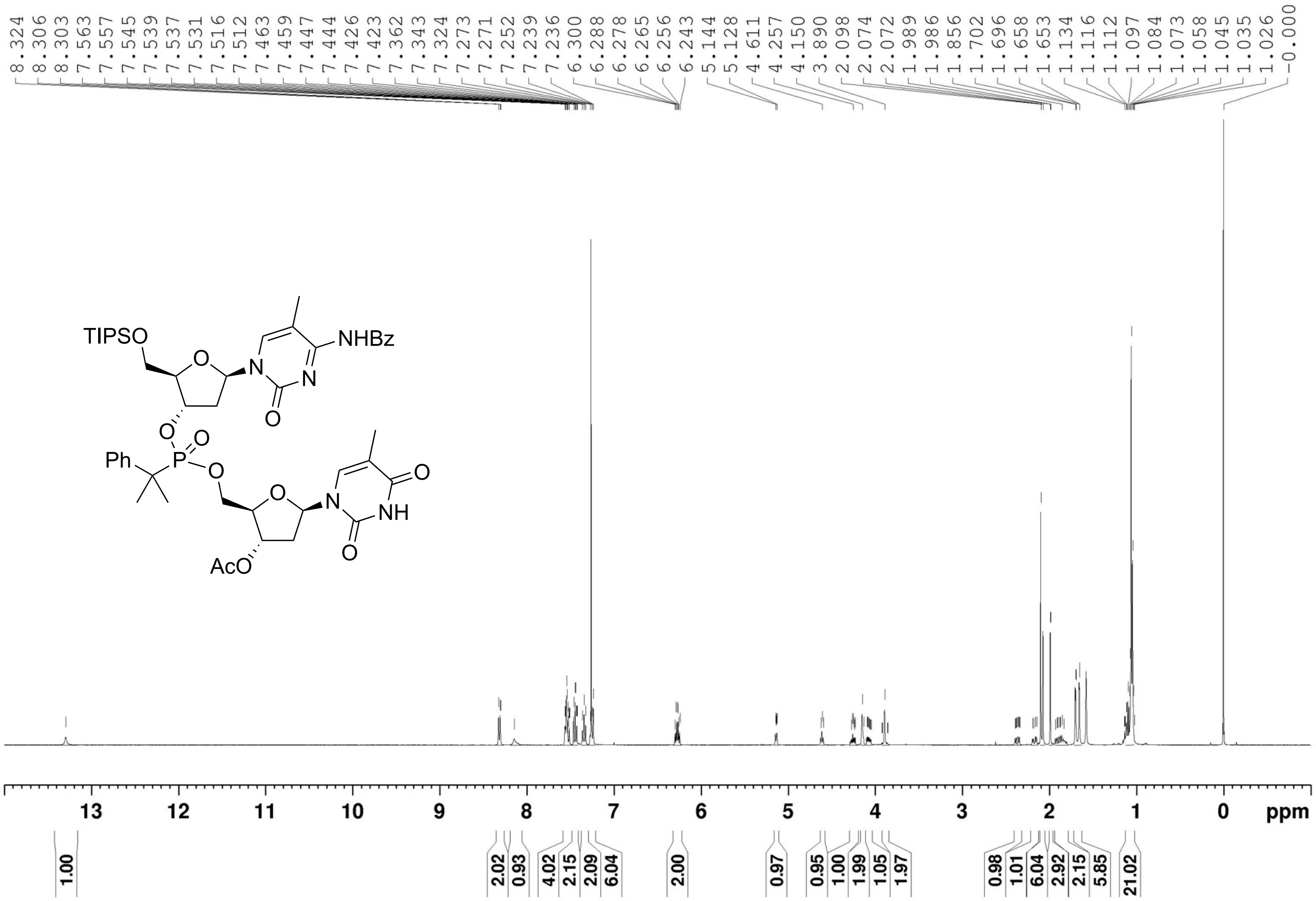


$^1$H NMR (400 MHz, $CDCl_3$) spectrum of **3ba-d1**

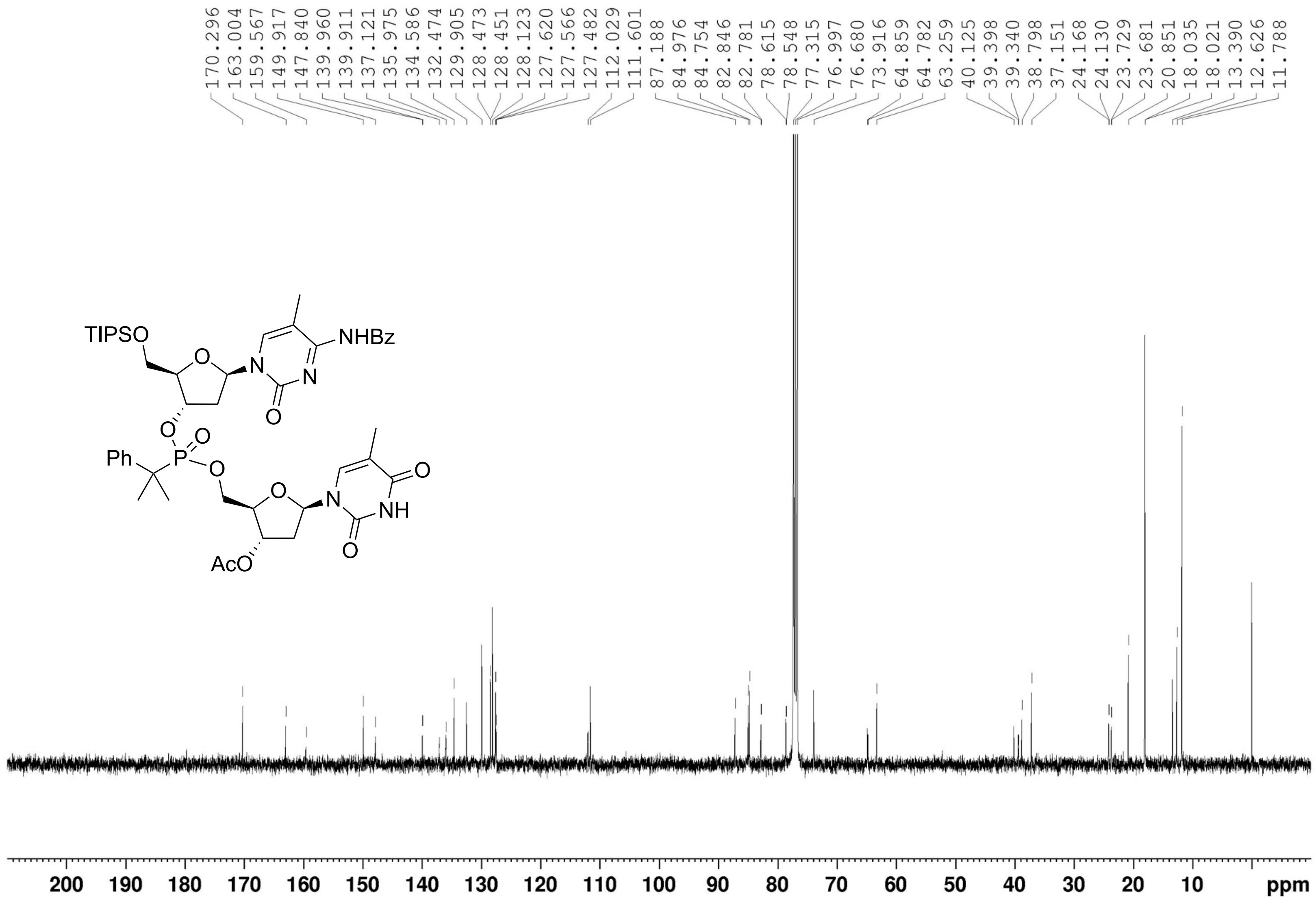


$^{13}$C NMR (100.6 MHz, $CDCl_3$) spectrum of **3ba-d1**

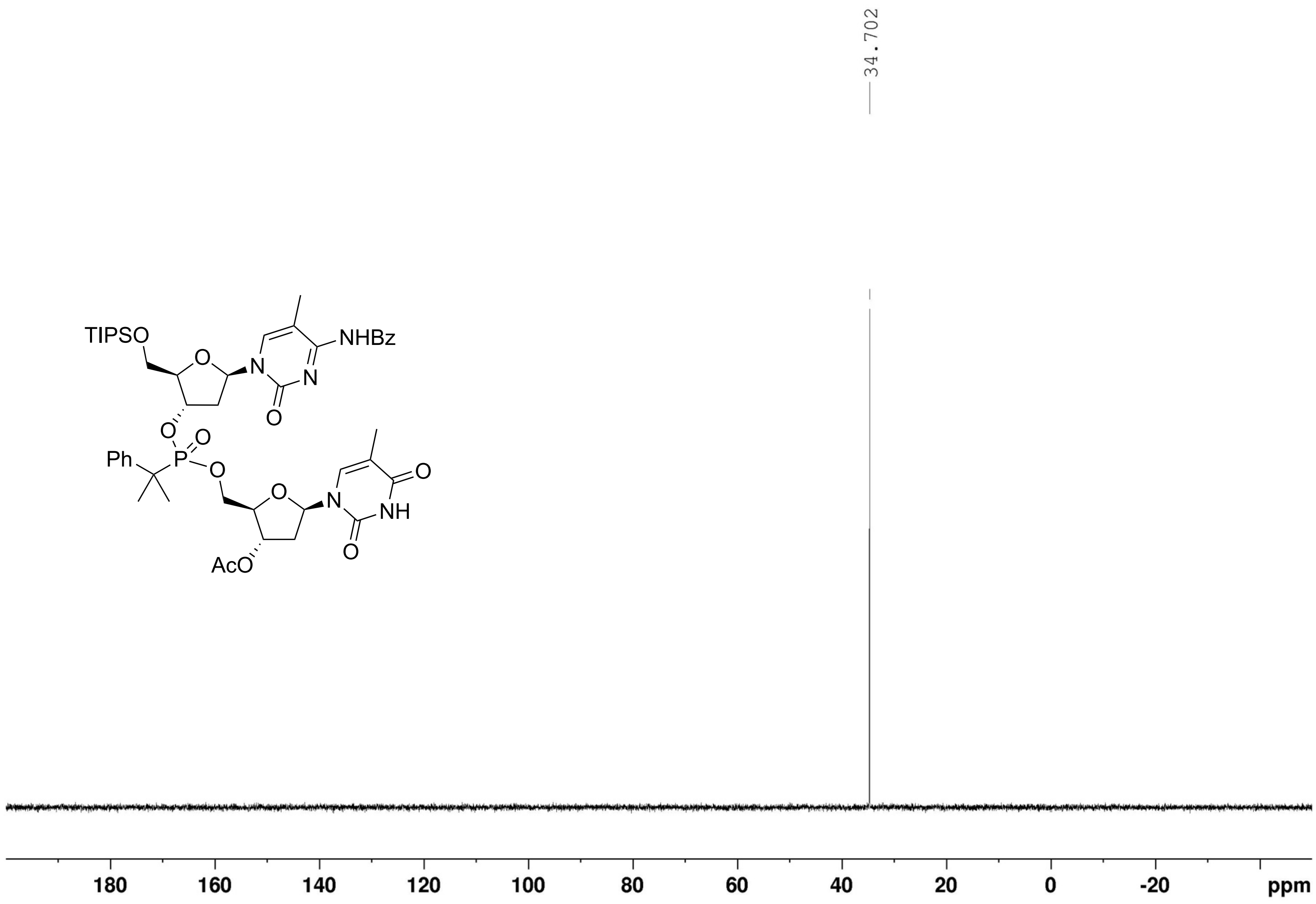


$^{31}P$ NMR (162 MHz, $CDCl_3$) spectrum of **3ba-d1**

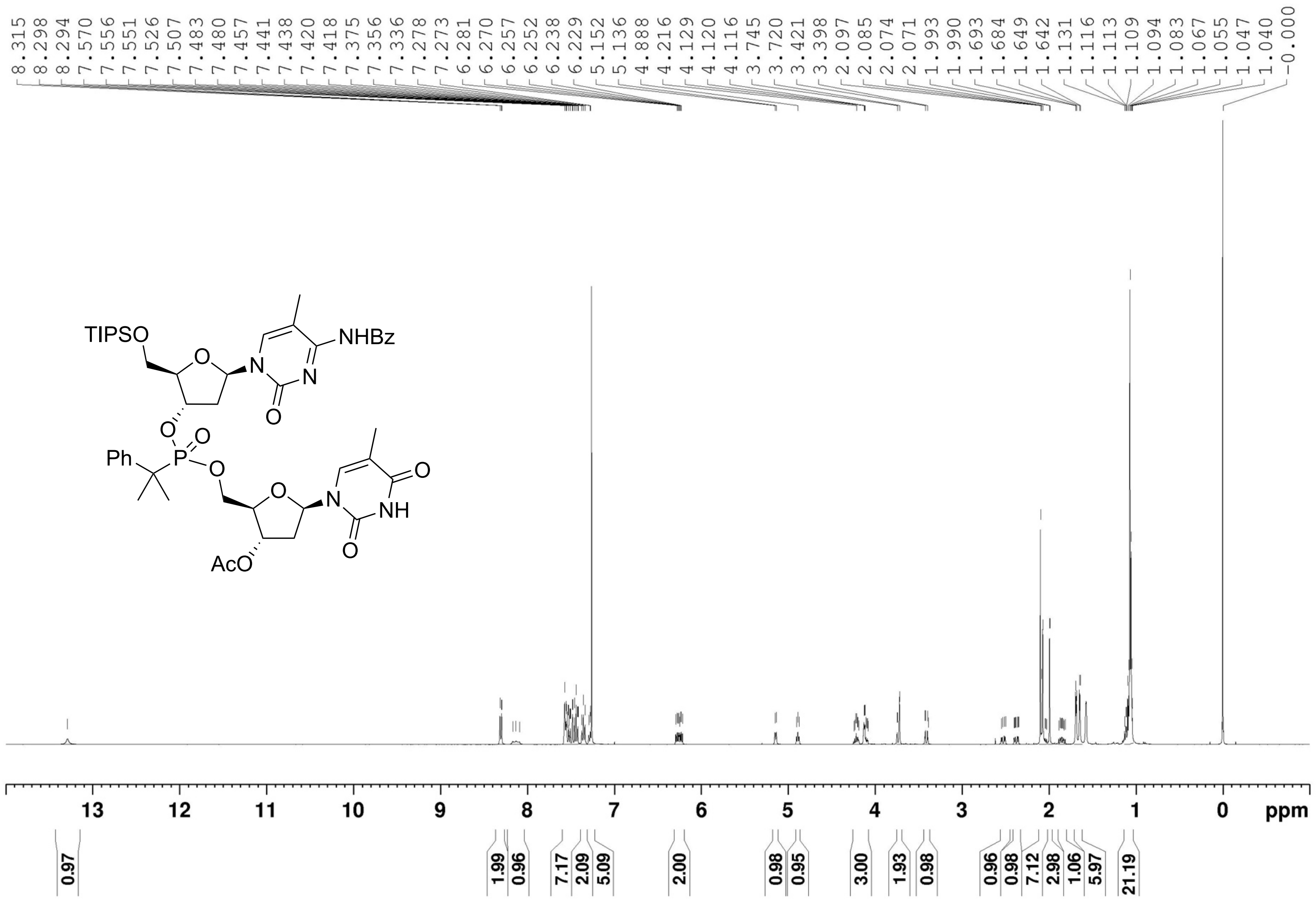


$^1$H NMR (400 MHz, $CDCl_3$) spectrum of **3ba-d2**

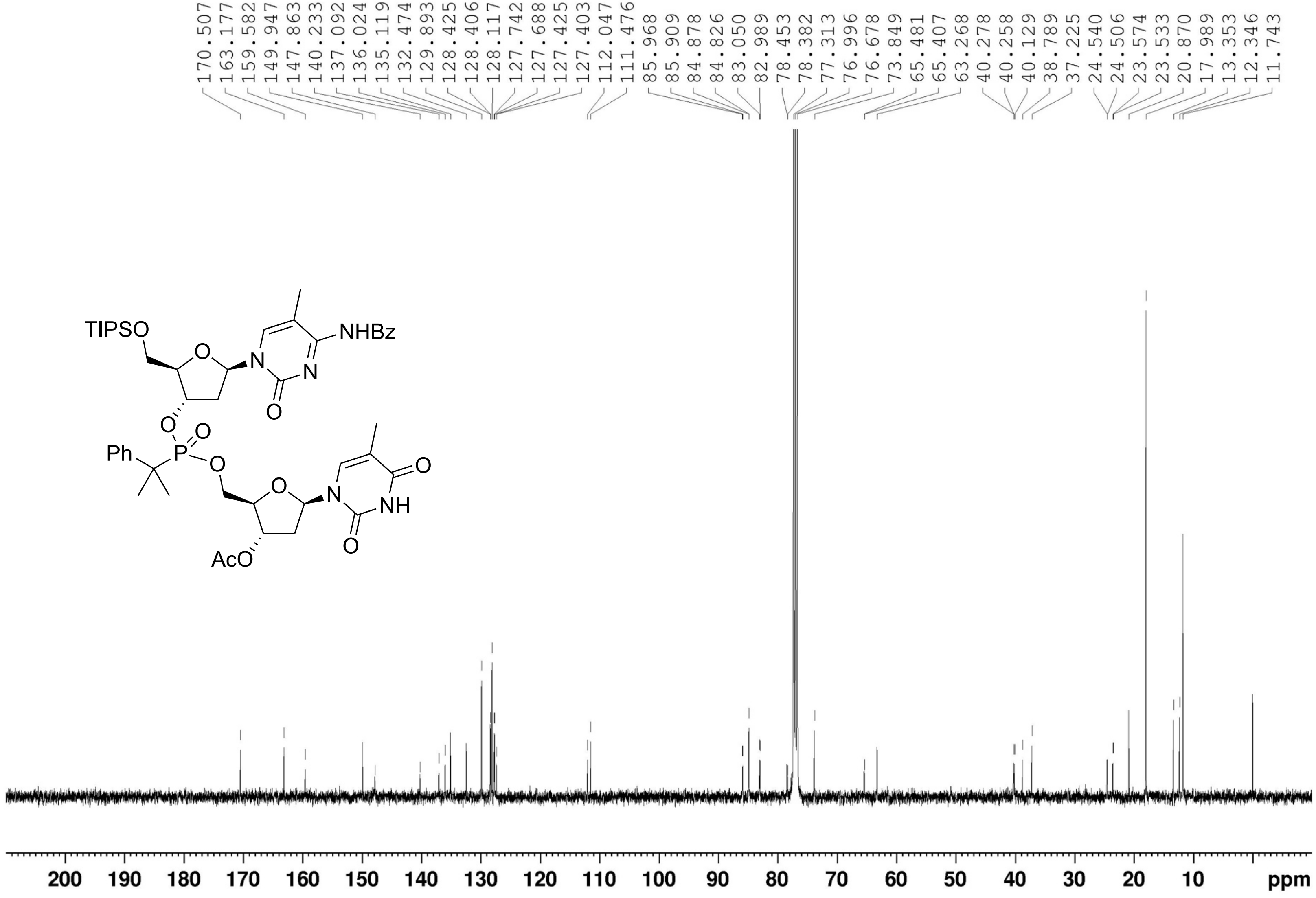


$^{13}C$ NMR (100.6 MHz, $CDCl_3$) spectrum of **3ba-d2**

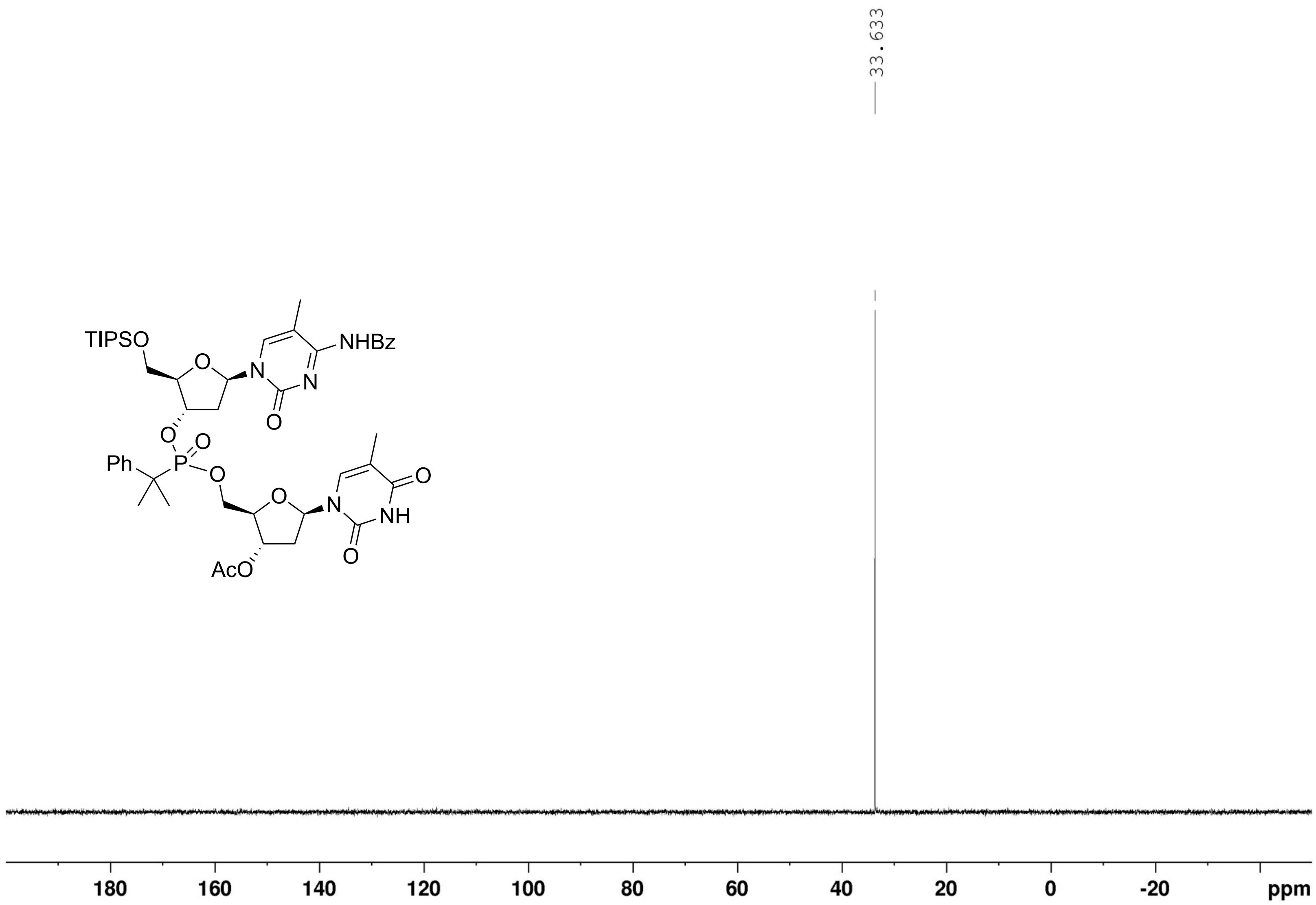


$^{31}$P NMR (162 MHz, $CDCl_3$) spectrum of **3ba-d2**

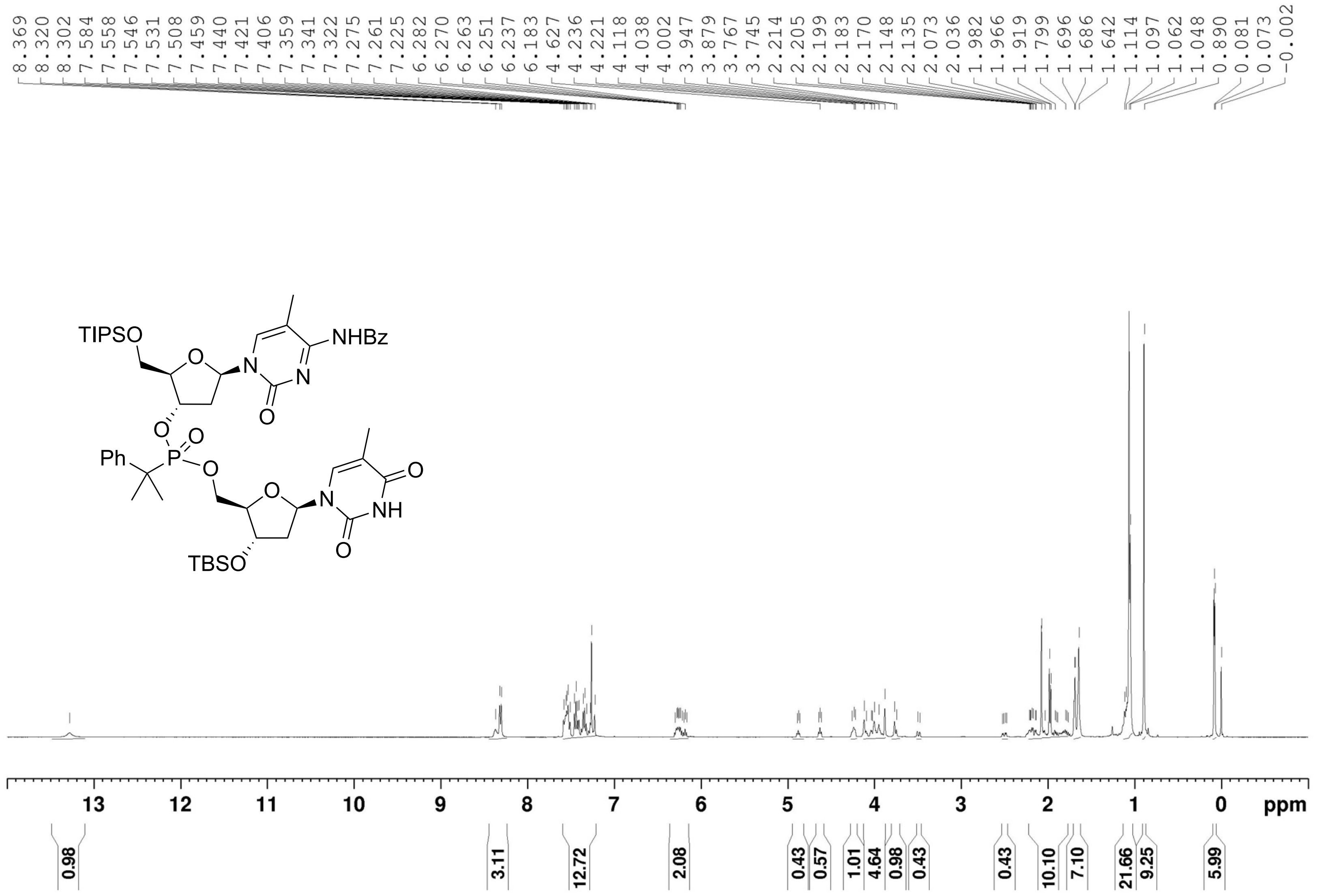


$^{1}$H NMR (400 MHz, $CDCl_3$) spectrum of **3ca**

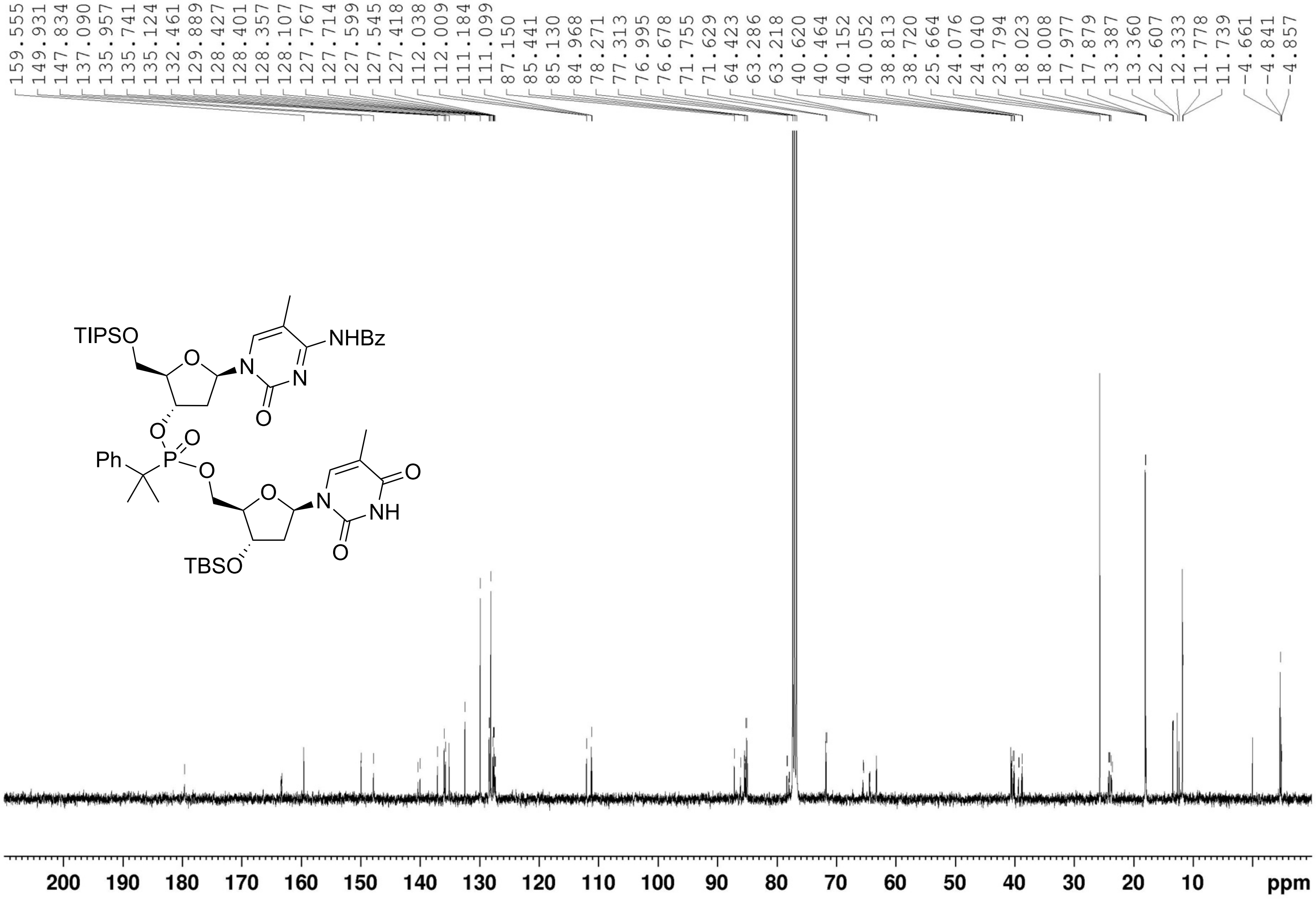


$^{13}$C NMR (100.6 MHz, $CDCl_3$) spectrum of **3ca**

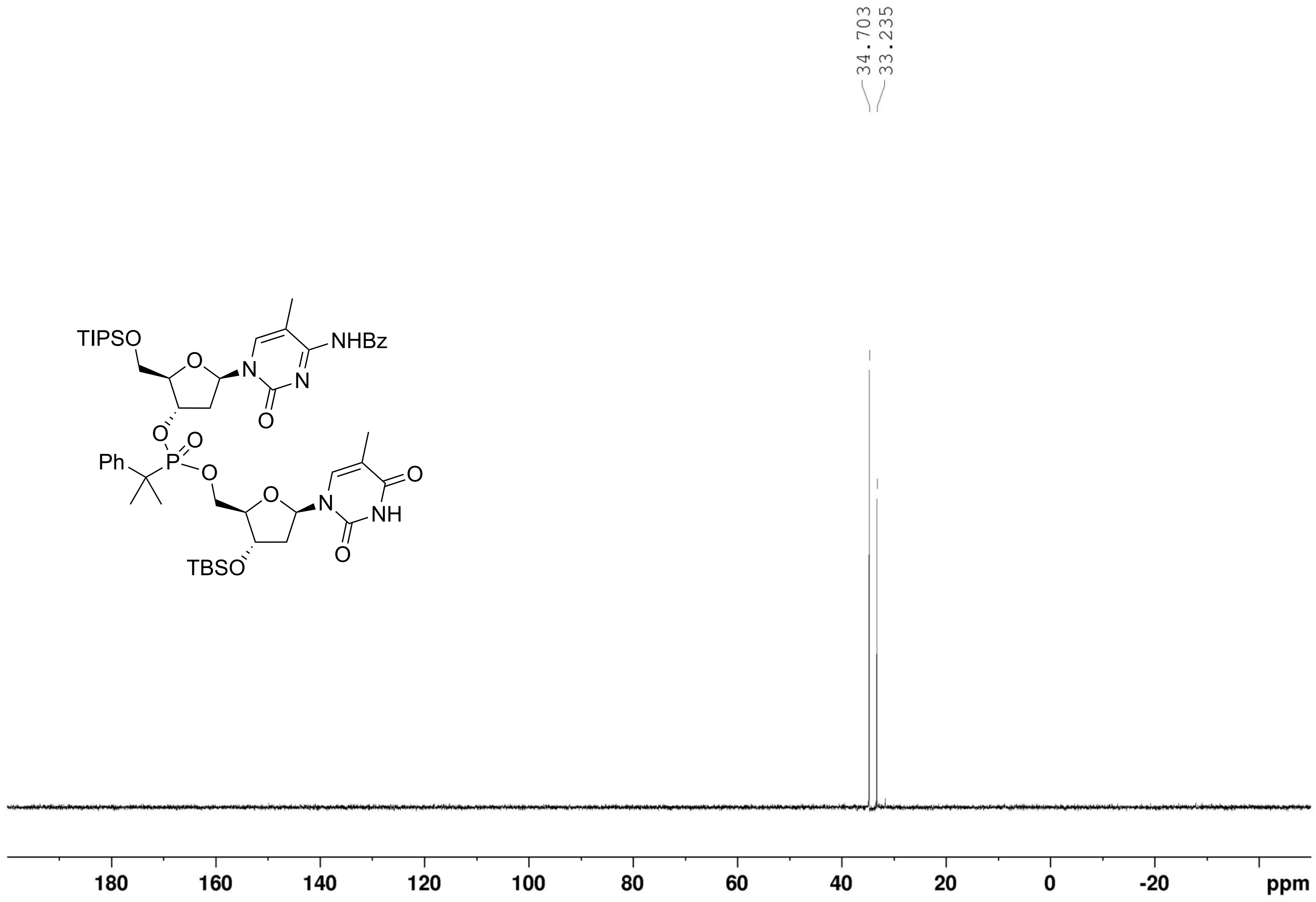


$^{31}$P NMR (162 MHz, $CDCl_3$) spectrum of **3ca**

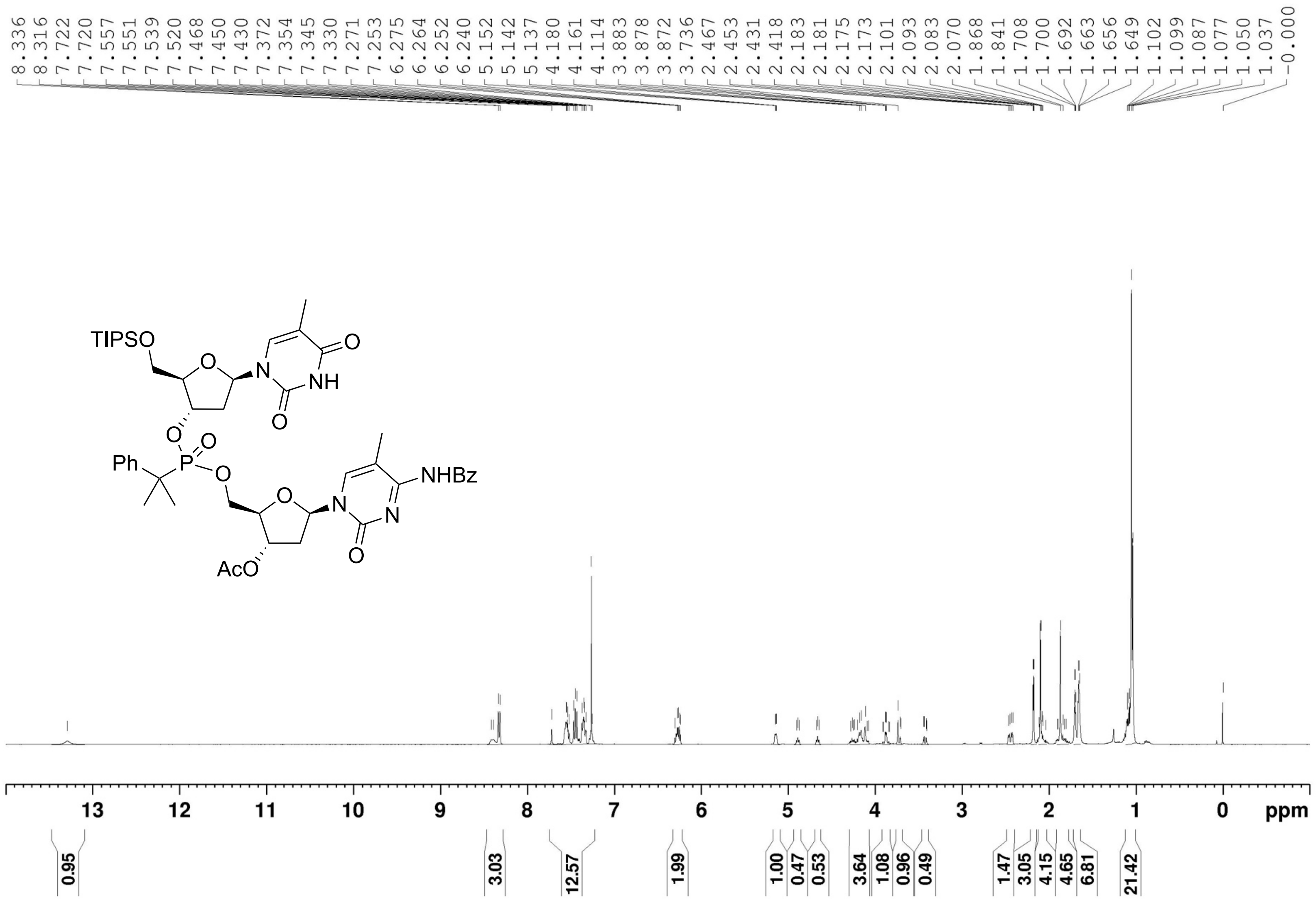

$^{1}H$ NMR (400 MHz, $CDCl_3$) spectrum of **3da**

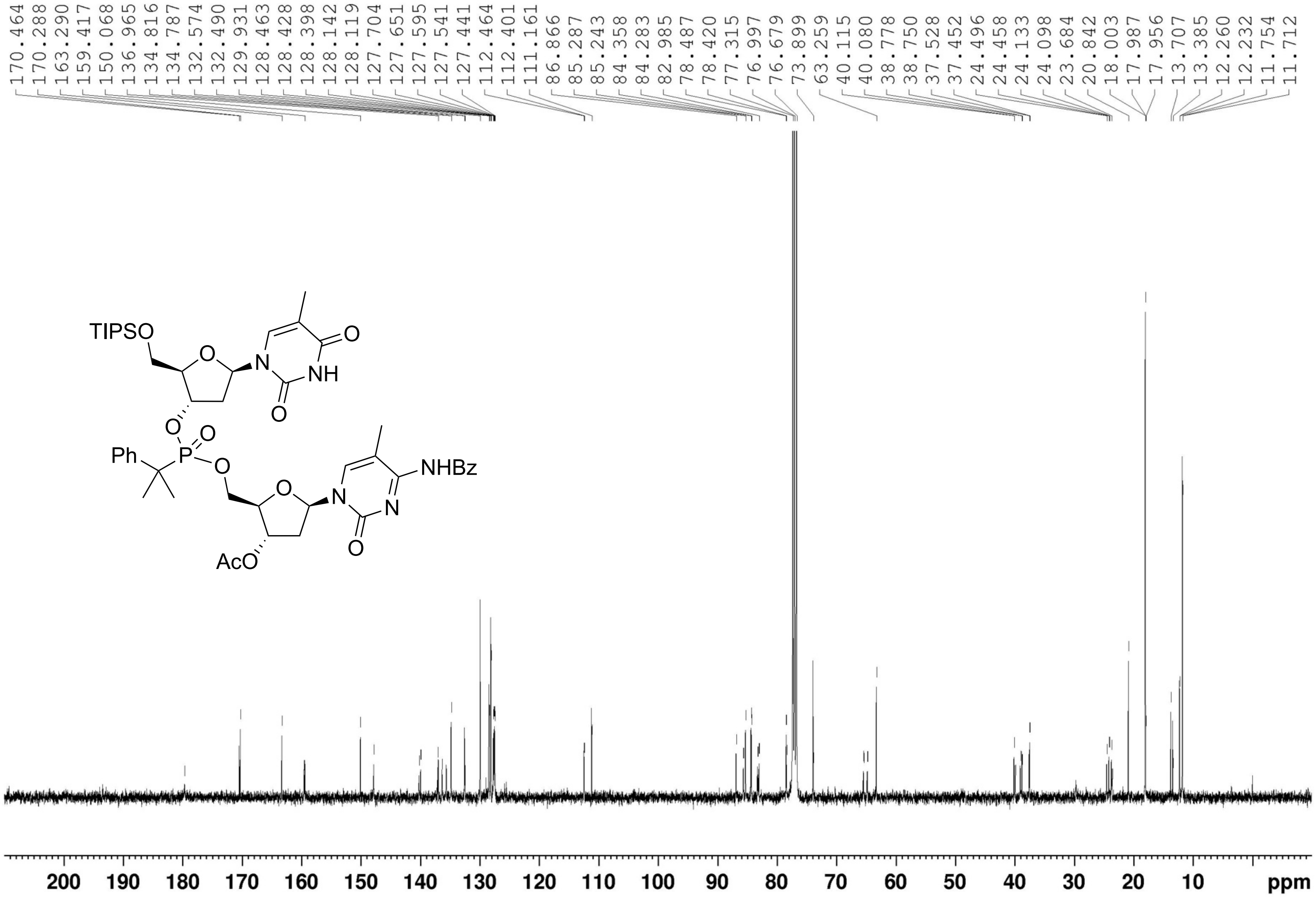


$^{13}C$ NMR (100.6 MHz, $CDCl_3$) spectrum of **3da**

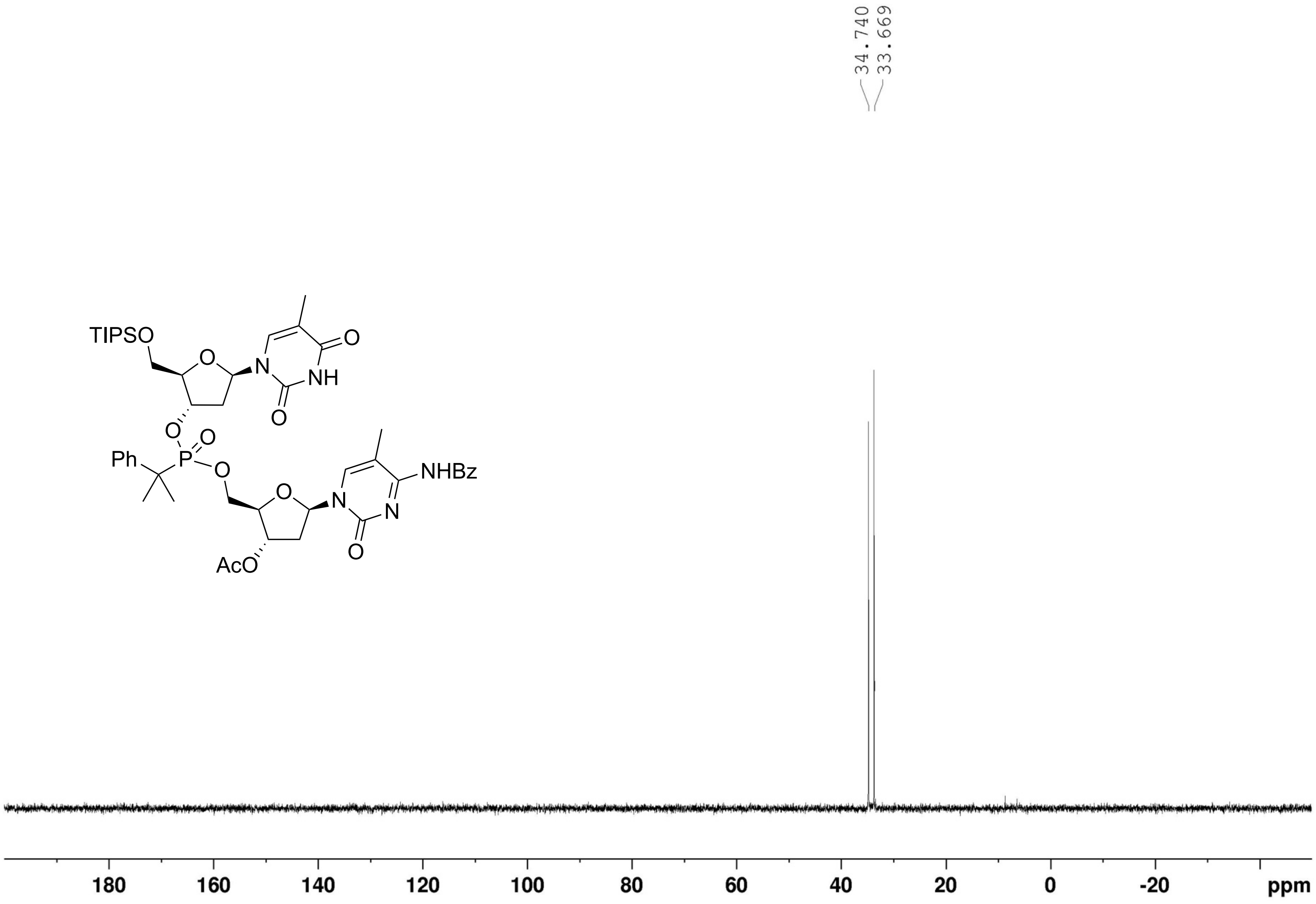


$^{31}$P NMR (162 MHz, $CDCl_3$) spectrum of **3da**

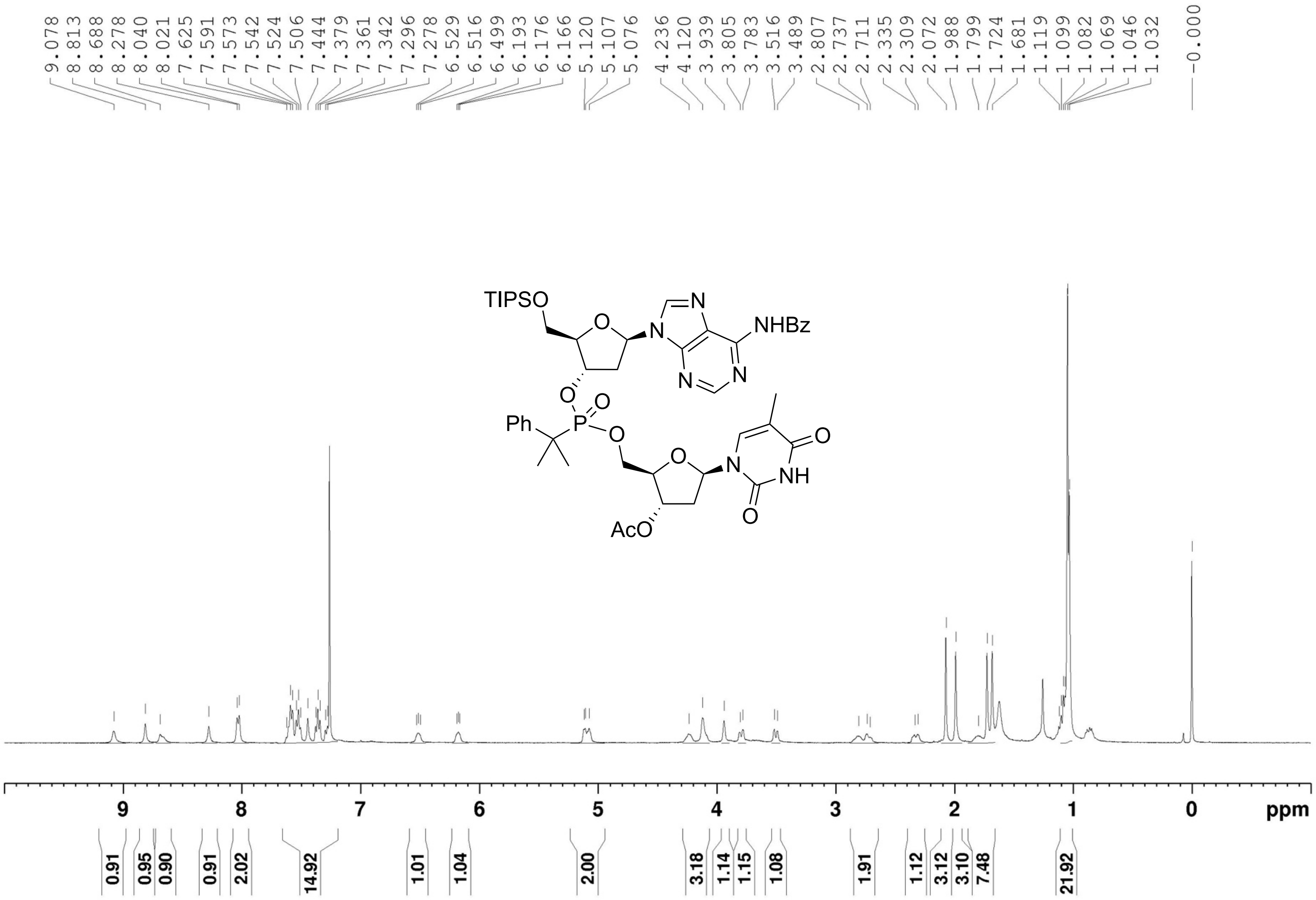

$^{1}$H NMR (400 MHz, $CDCl_3$) spectrum of **3ea-d1**

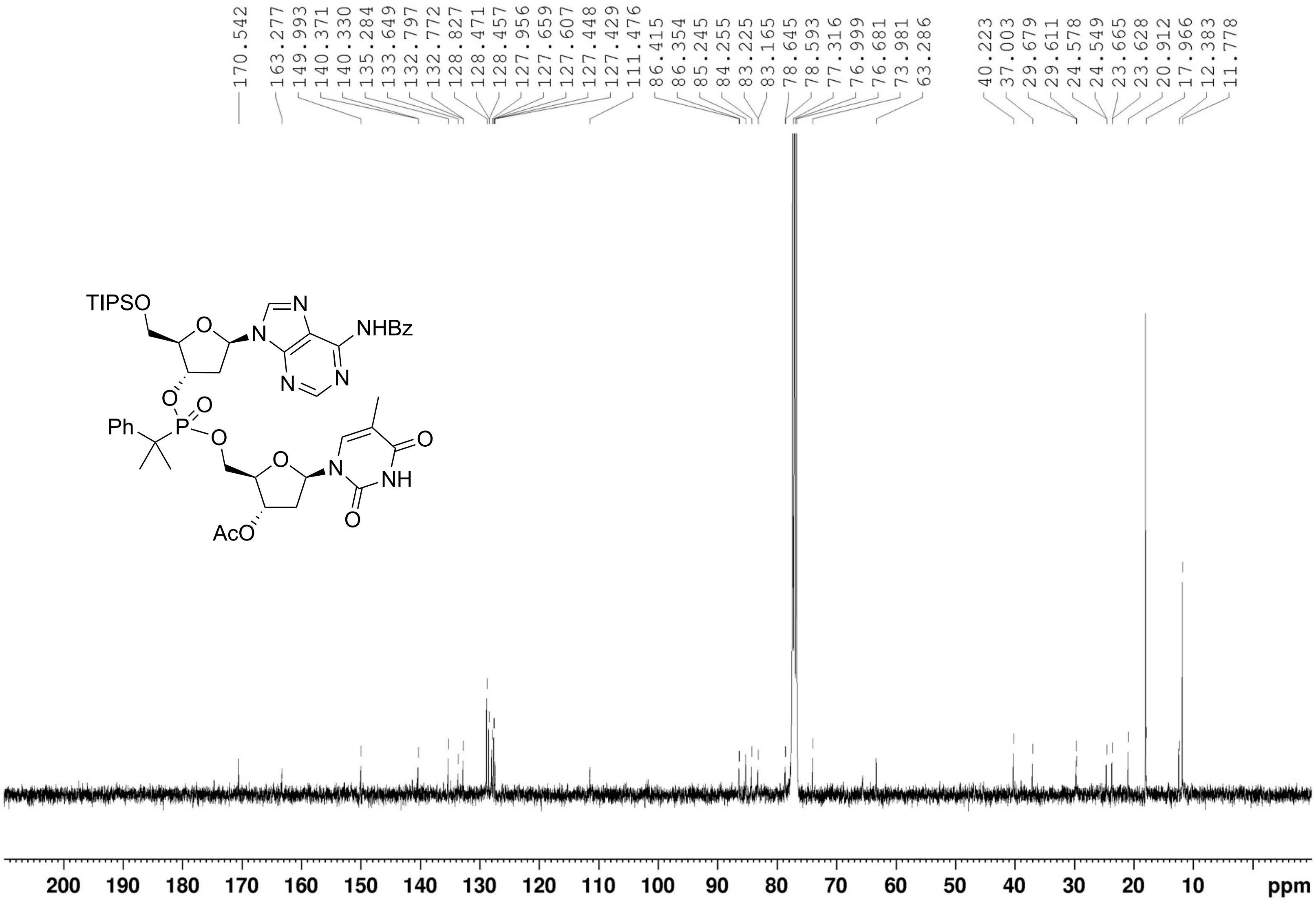


$^{13}C$ NMR (100.6 MHz, $CDCl_3$) spectrum of **3ea-d1**

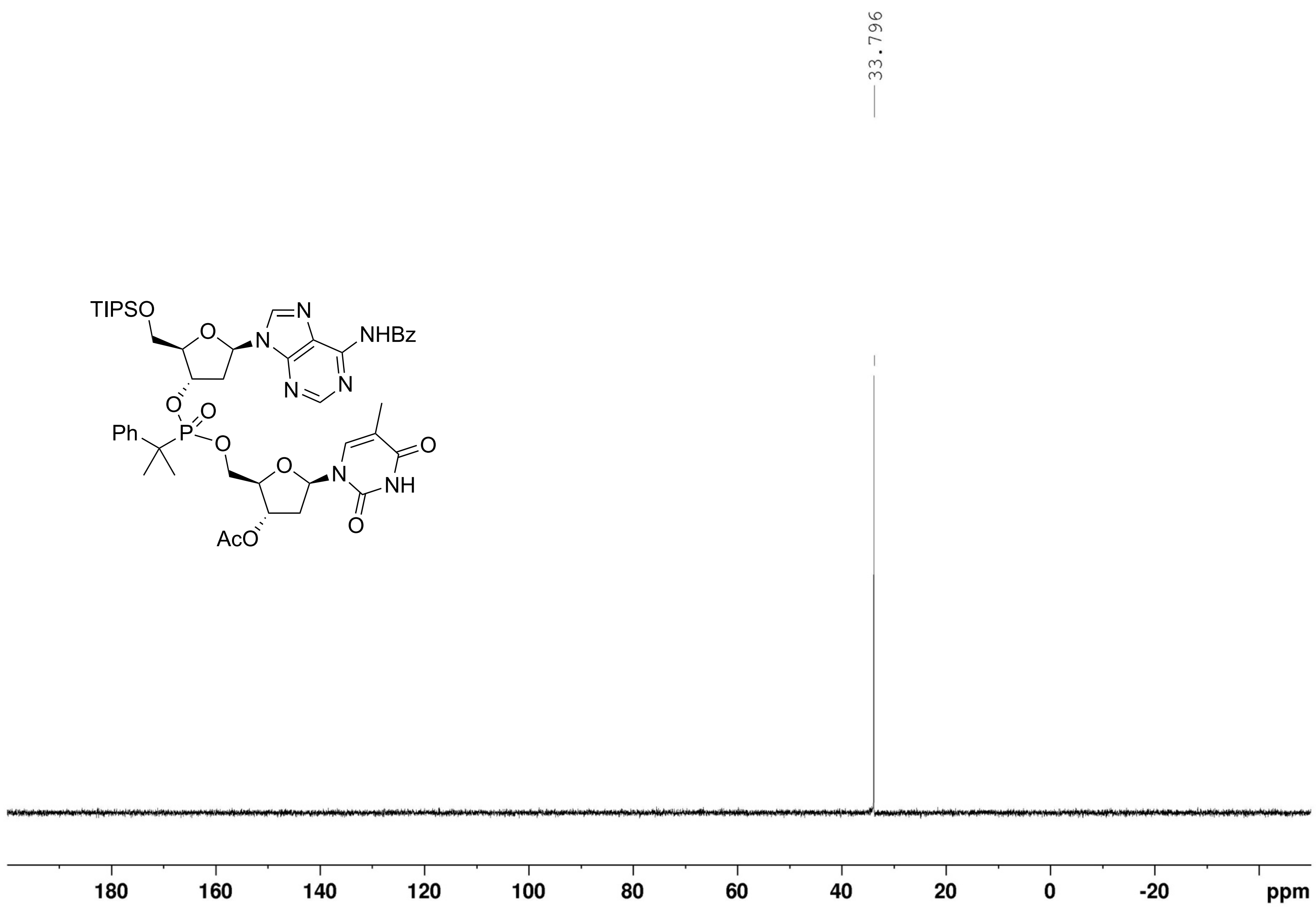


$^{31}P$ NMR (162 MHz, $CDCl_3$) spectrum of **3ea-d1**

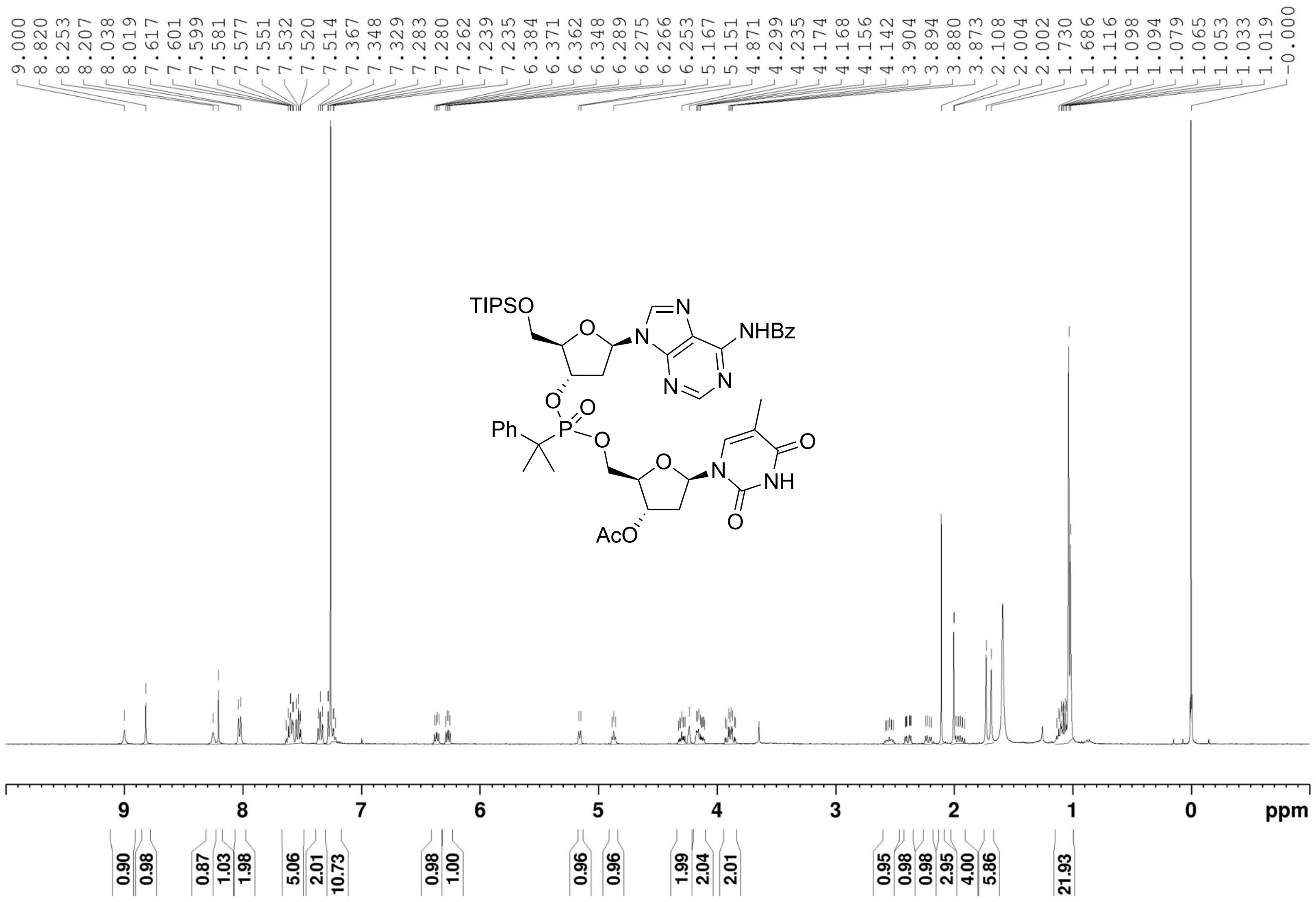

$^{1}$H NMR (400 MHz, $CDCl_3$) spectrum of **3ea-d2**

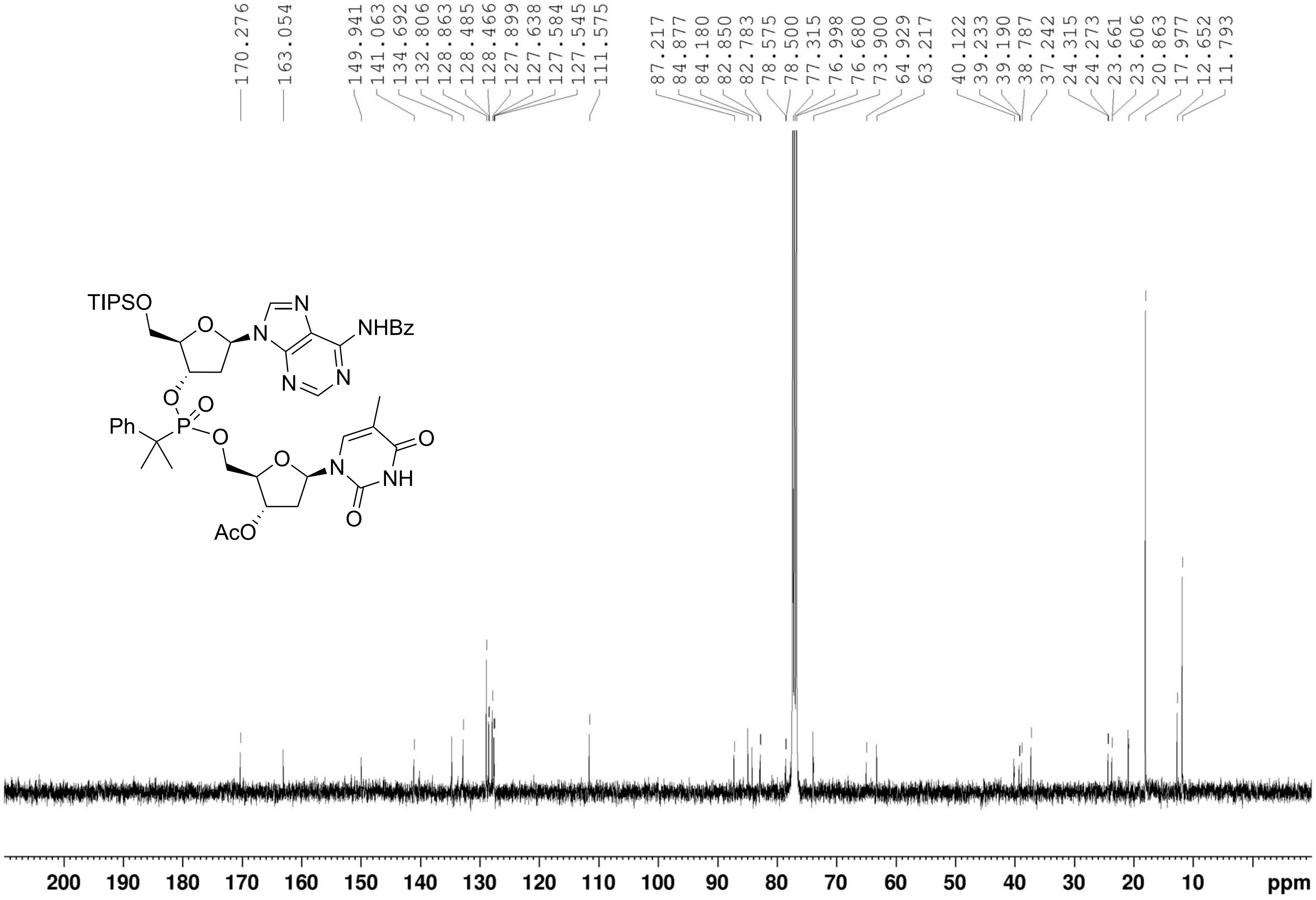


$^{13}C$ NMR (100.6 MHz, $CDCl_3$) spectrum of **3ea-d2**

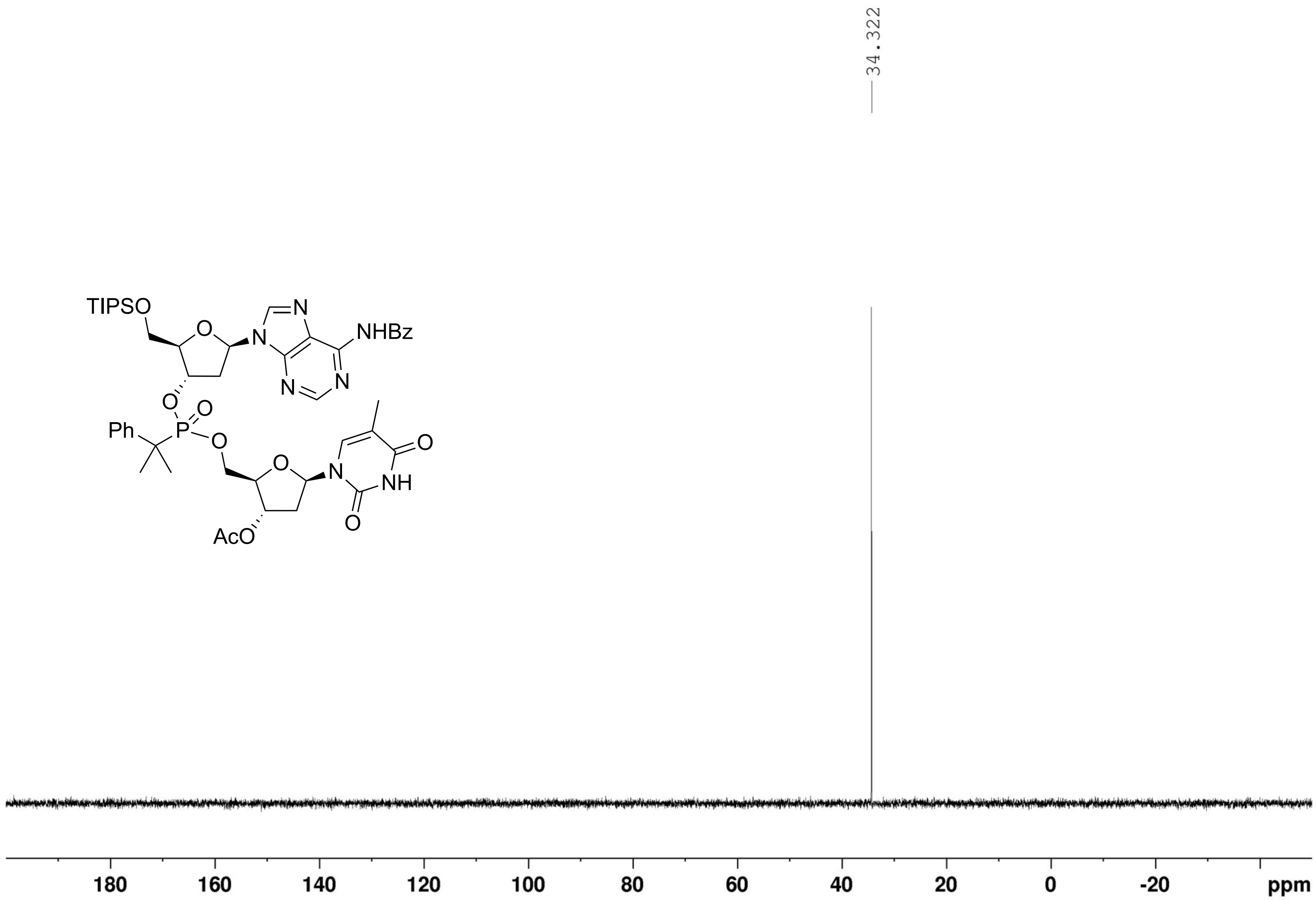


$^{31}P$ NMR (162 MHz, $CDCl_3$) spectrum of **3ea-d2**

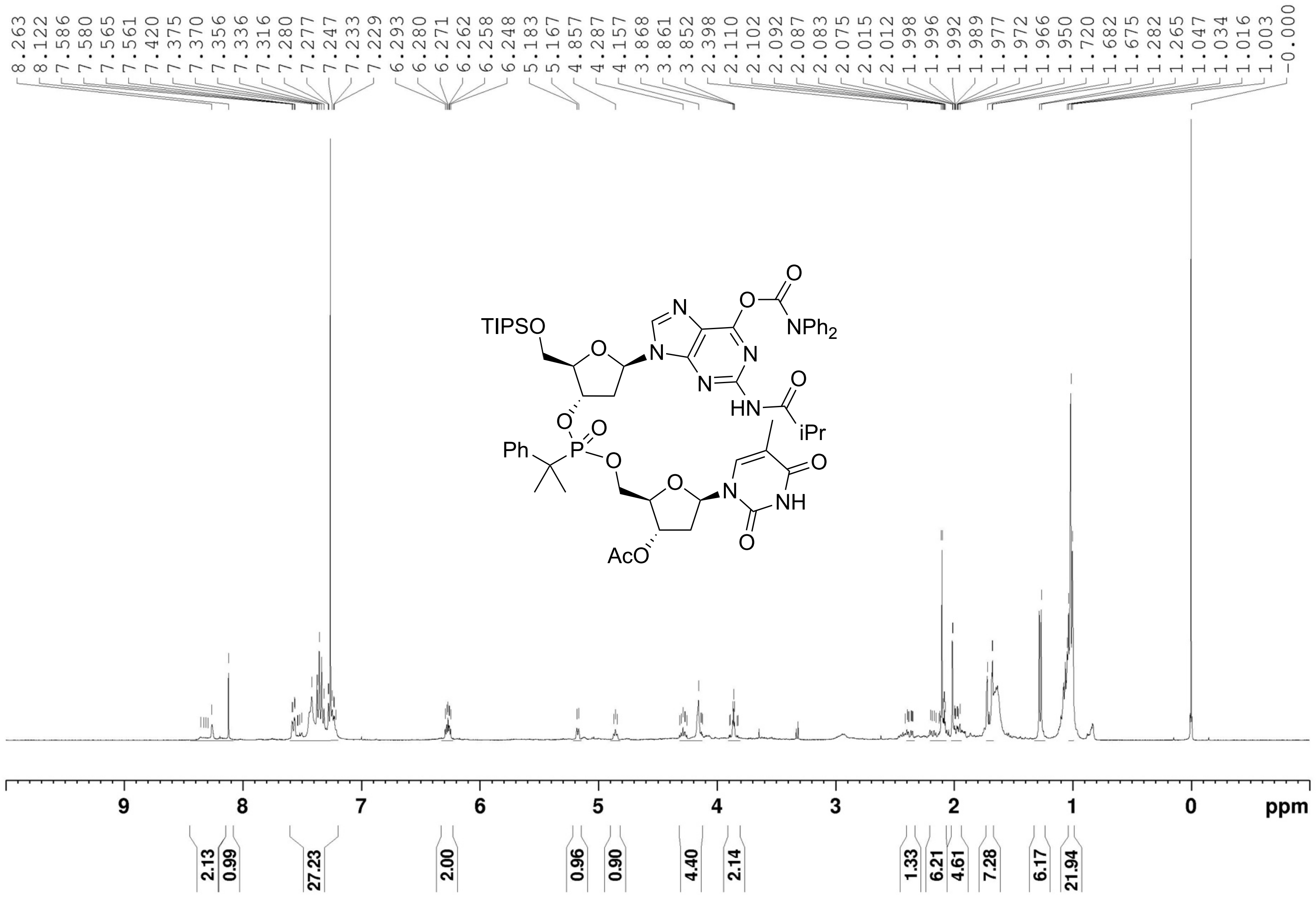


$^{1}$H NMR (400 MHz, $CDCl_3$) spectrum of **3fa-d1**

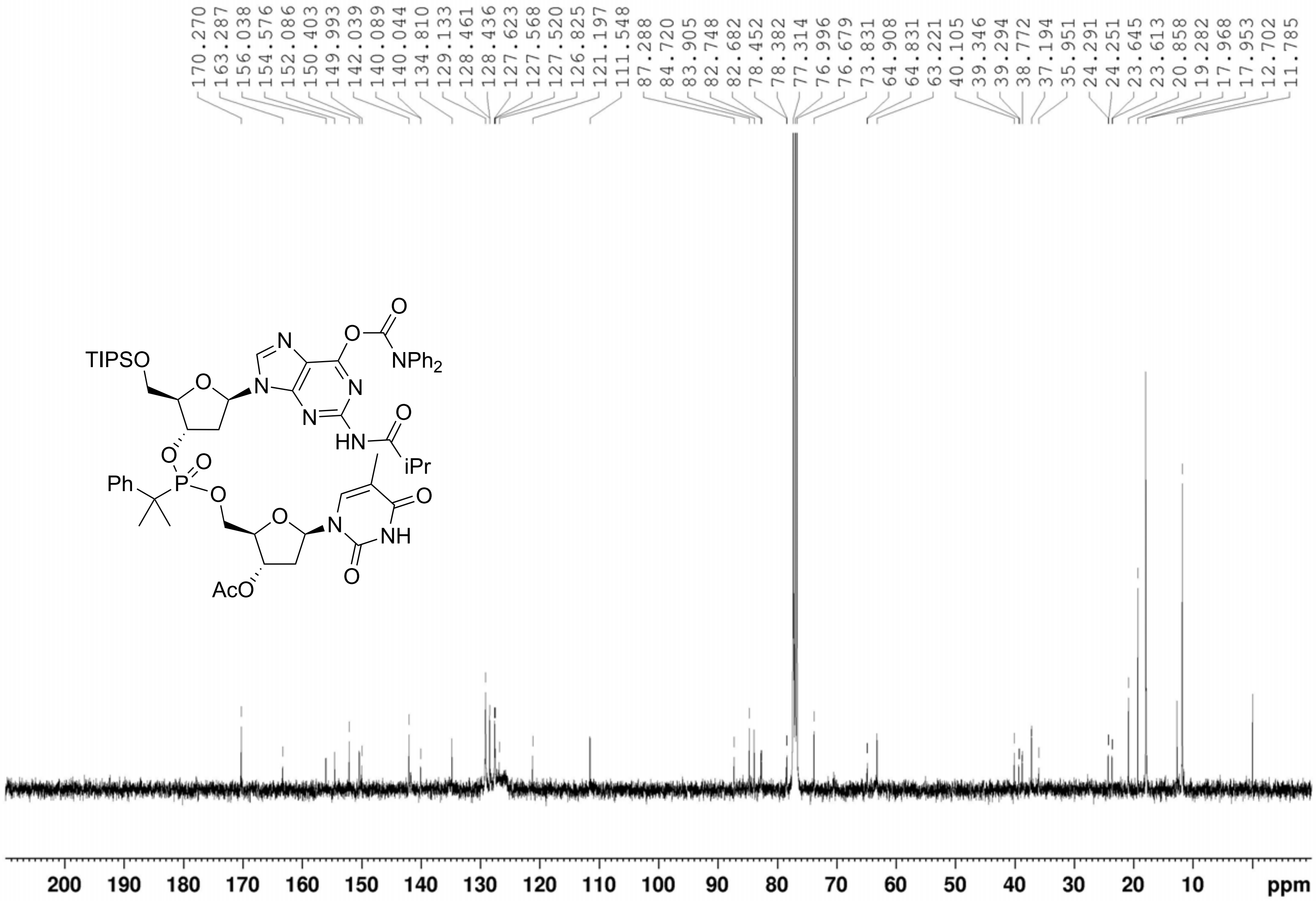


$^{13}$C NMR (100.6 MHz, CDCl$_3$) spectrum of **3fa-d1**

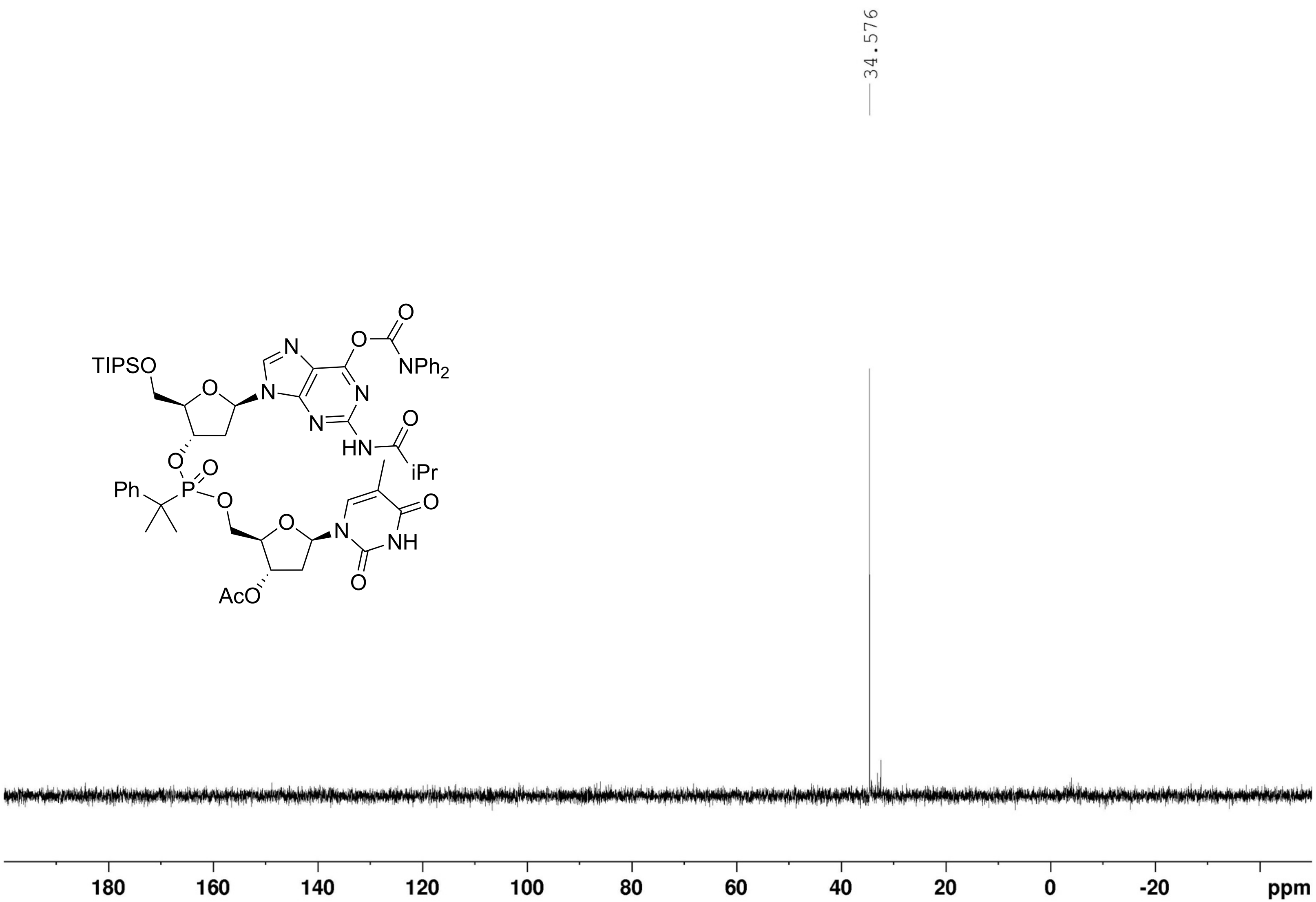


$^{31}P$ NMR (162 MHz, $CDCl_3$) spectrum of **3fa-d1**

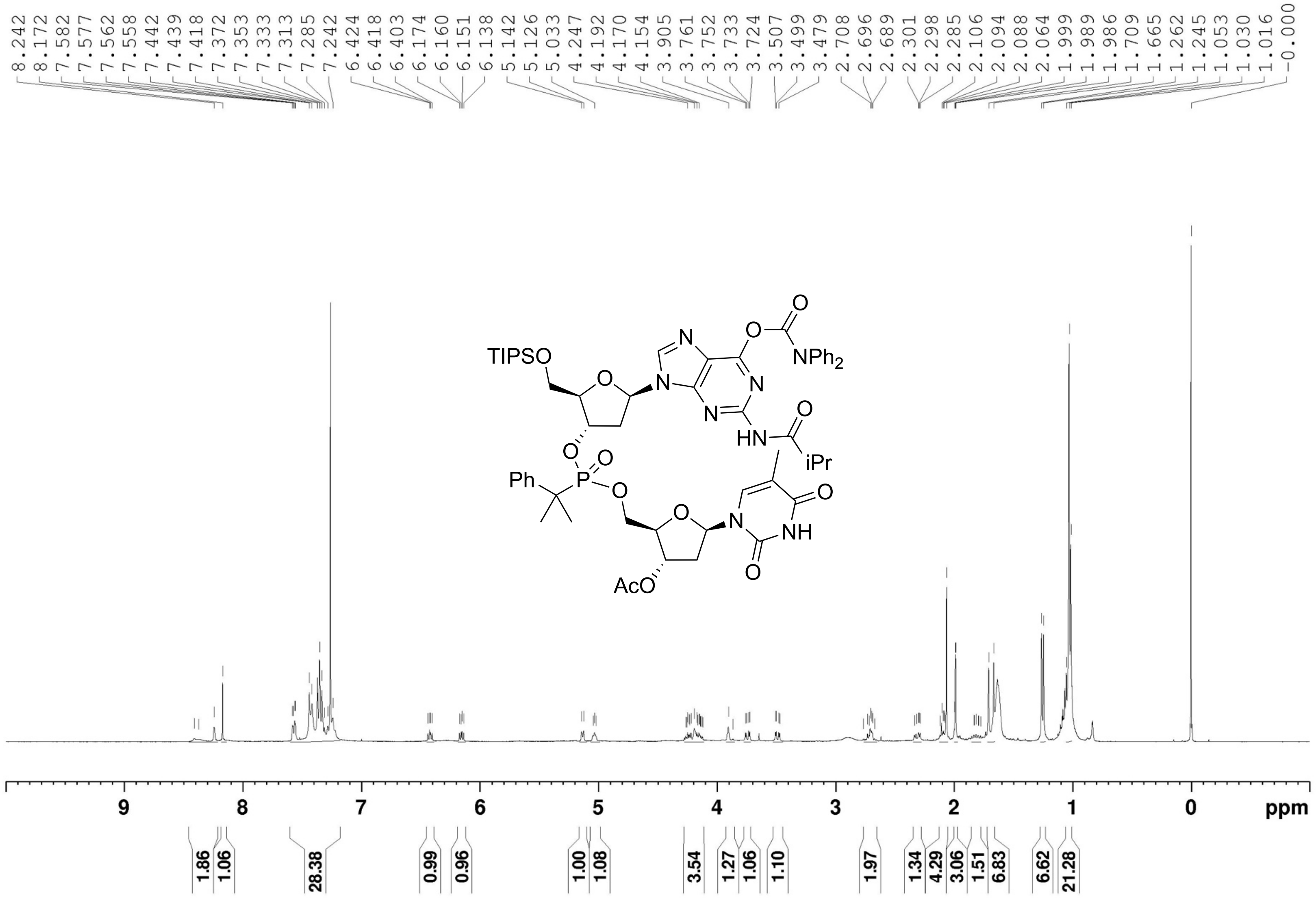


$^{1}$H NMR (400 MHz, $CDCl_3$) spectrum of **3fa-d2**

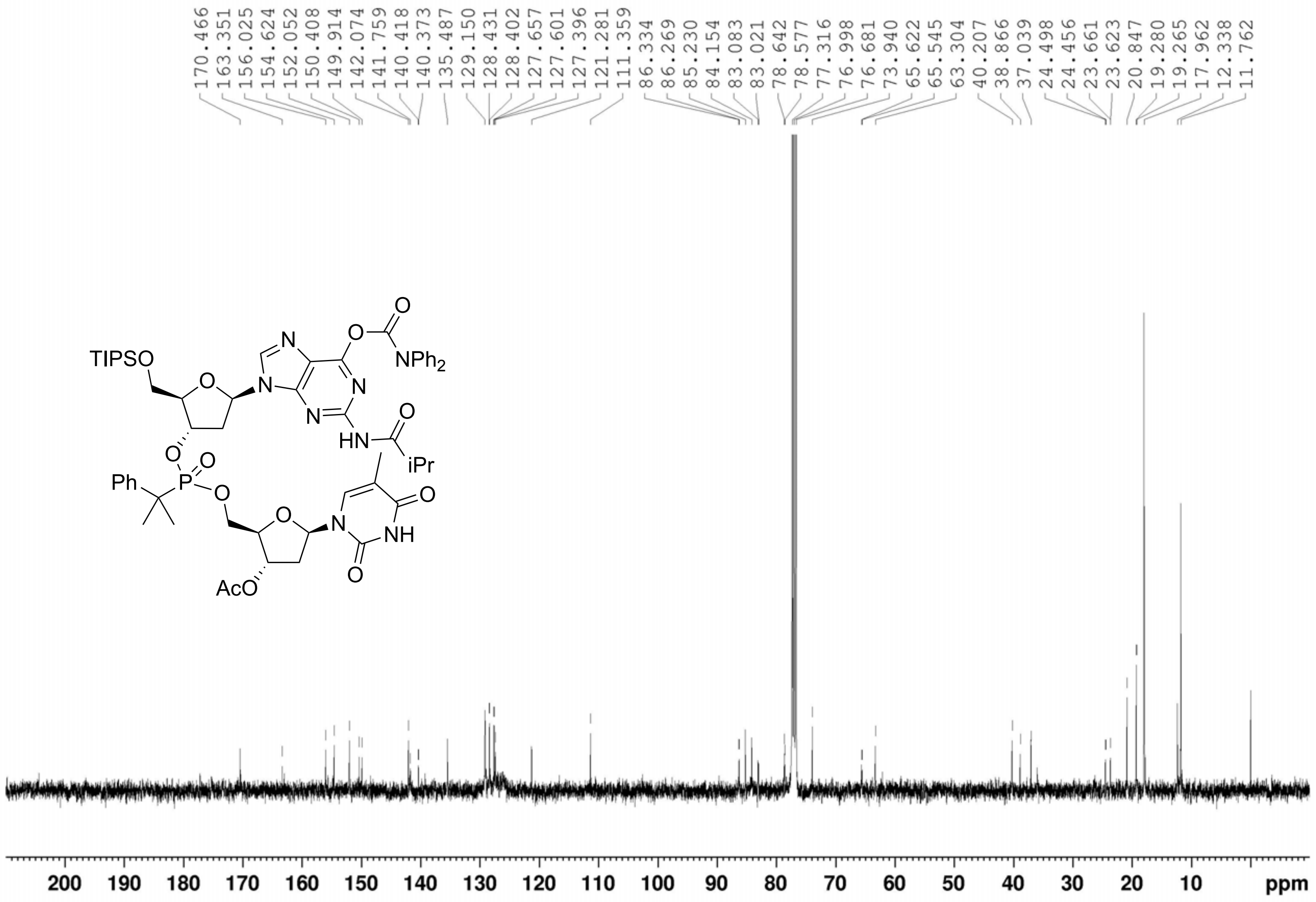


$^{13}$C NMR (100.6 MHz, $CDCl_3$) spectrum of **3fa-d2**

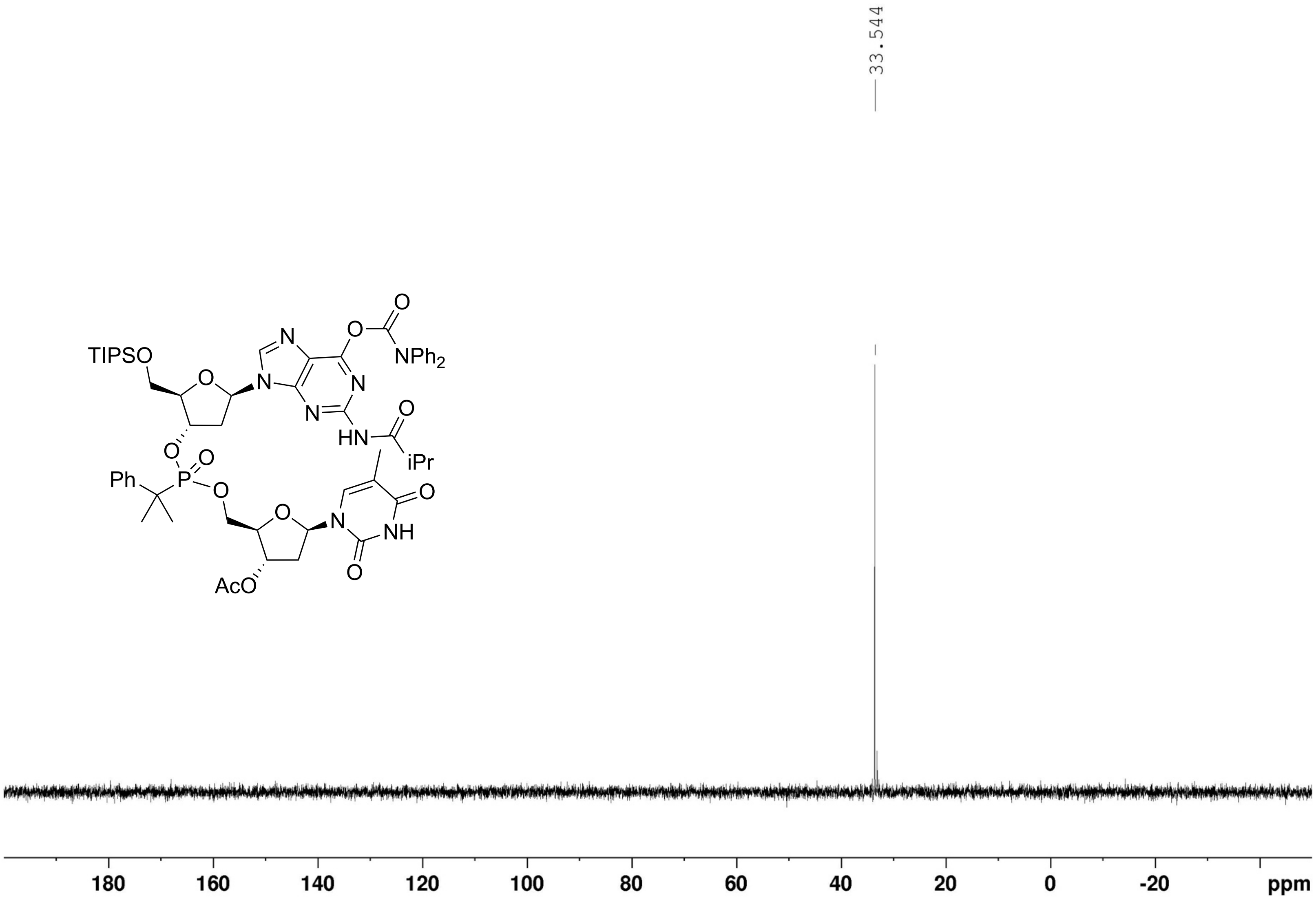


$^{31}$P NMR (162 MHz, $CDCl_3$) spectrum of **3fa-d2**

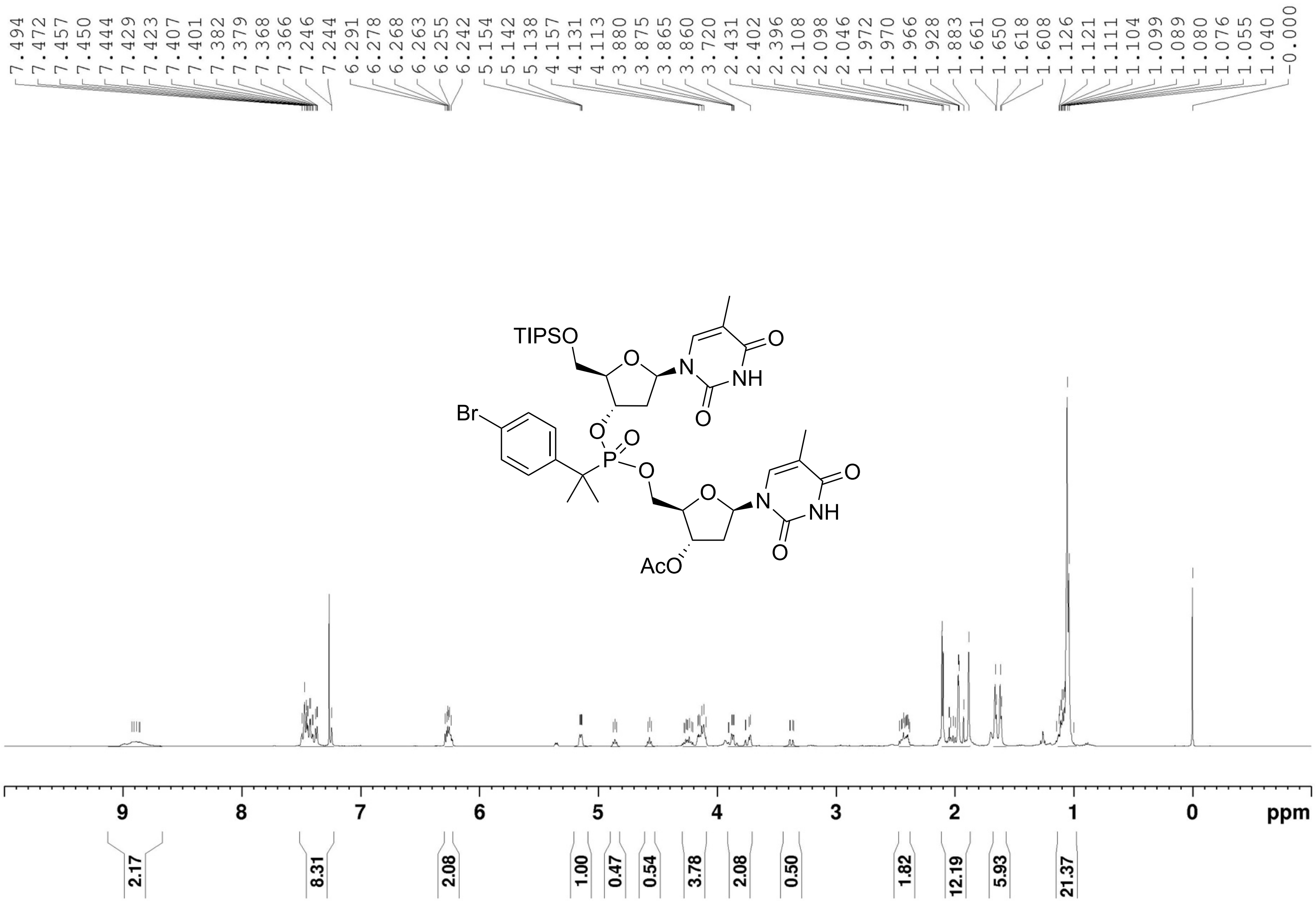


$^{1}H$ NMR (400 MHz, $CDCl_3$) spectrum of **3ab**

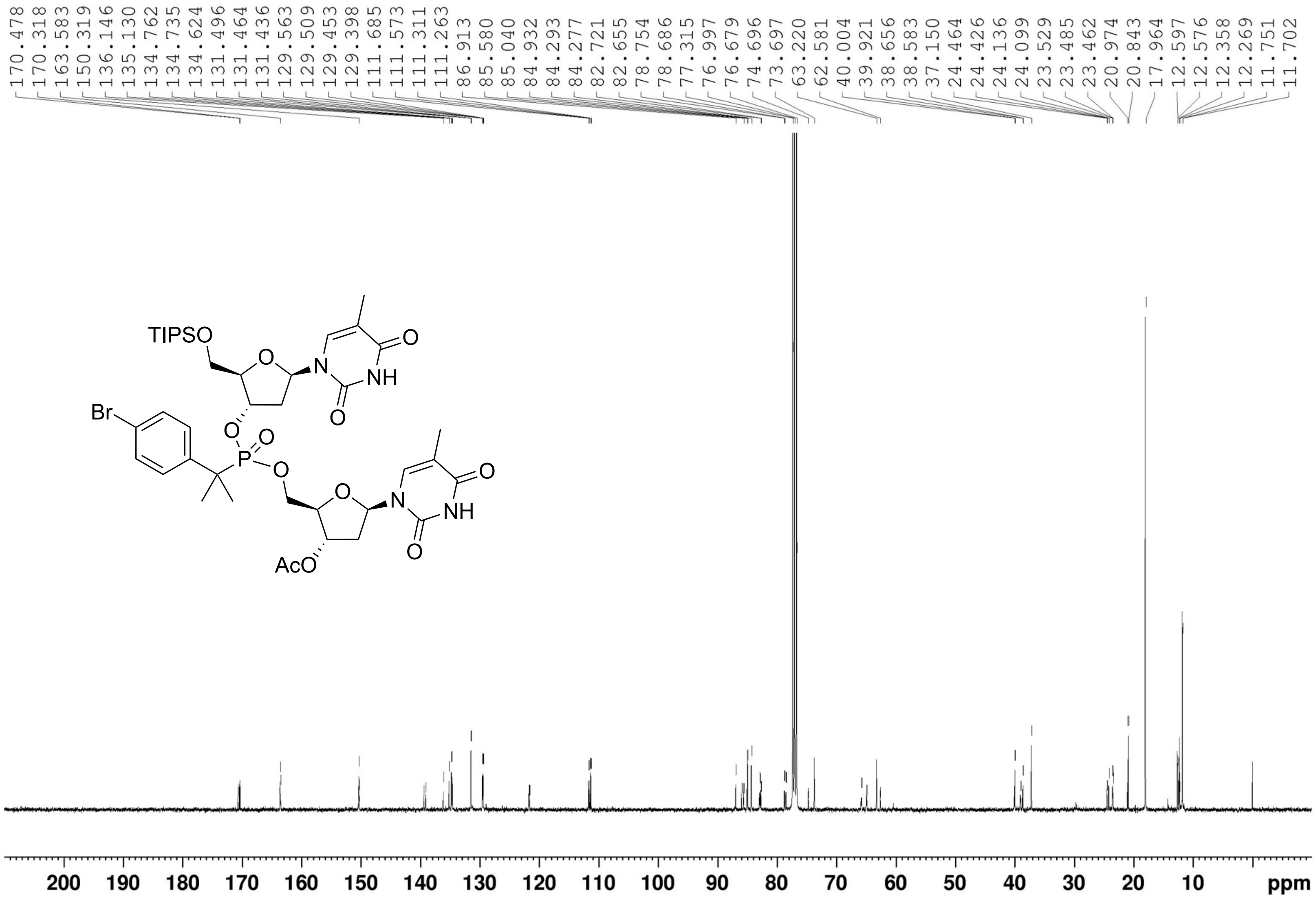


$^{13}C$ NMR (100.6 MHz, $CDCl_3$) spectrum of **3ab**

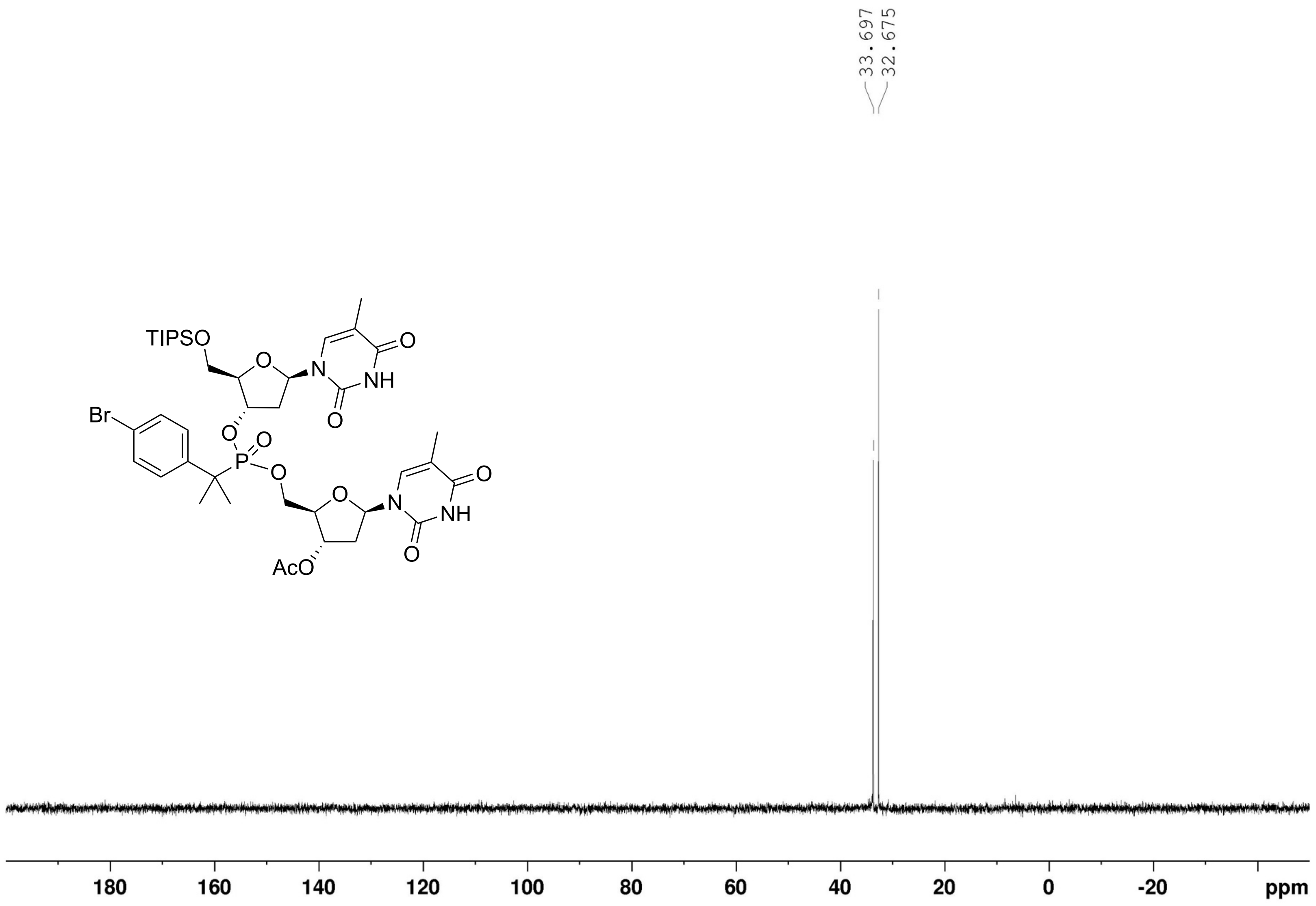


$^{31}P$ NMR (162 MHz, $CDCl_3$) spectrum of **3ab**

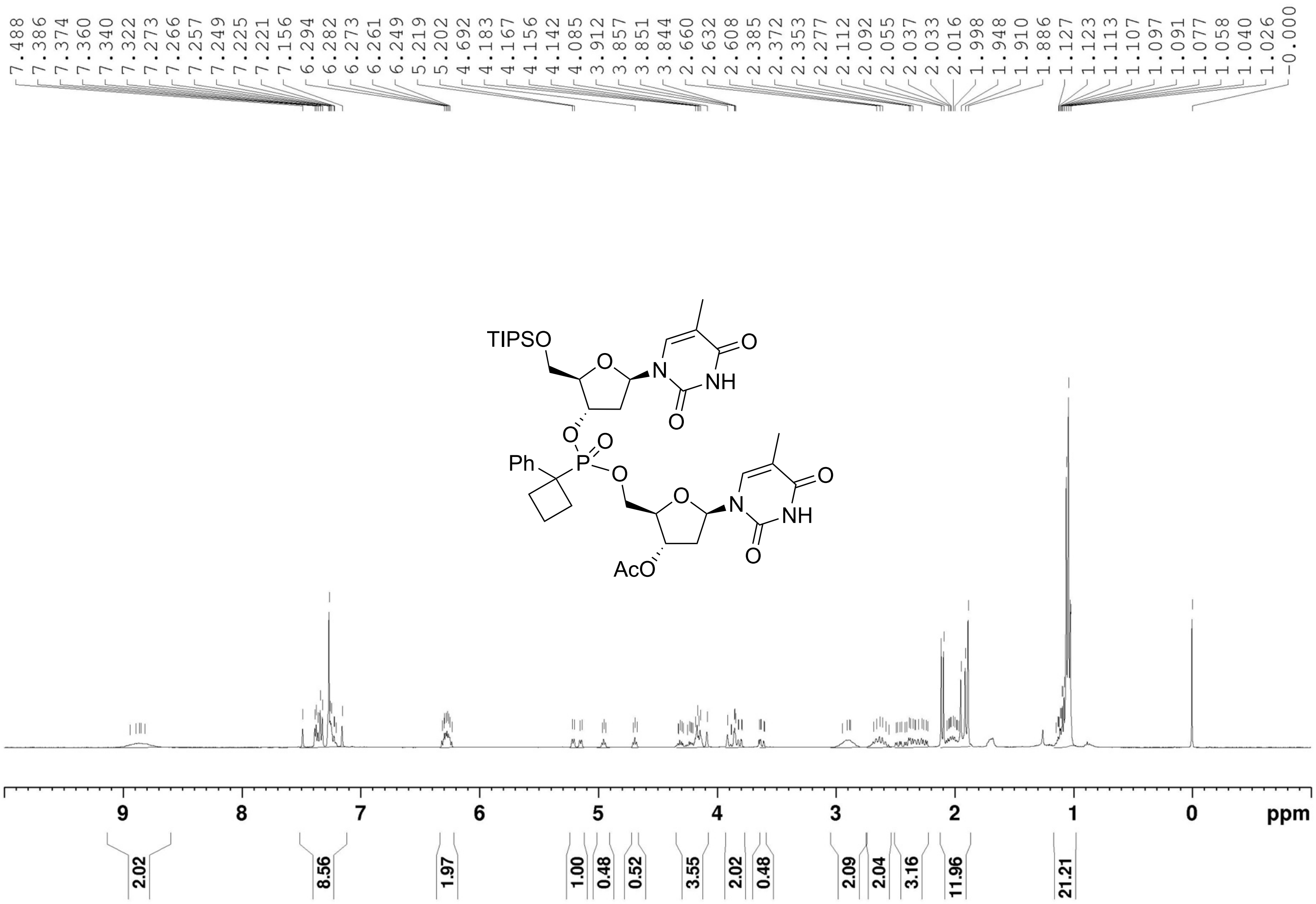

$^{1}$H NMR (400 MHz, $CDCl_3$) spectrum of **3ac**

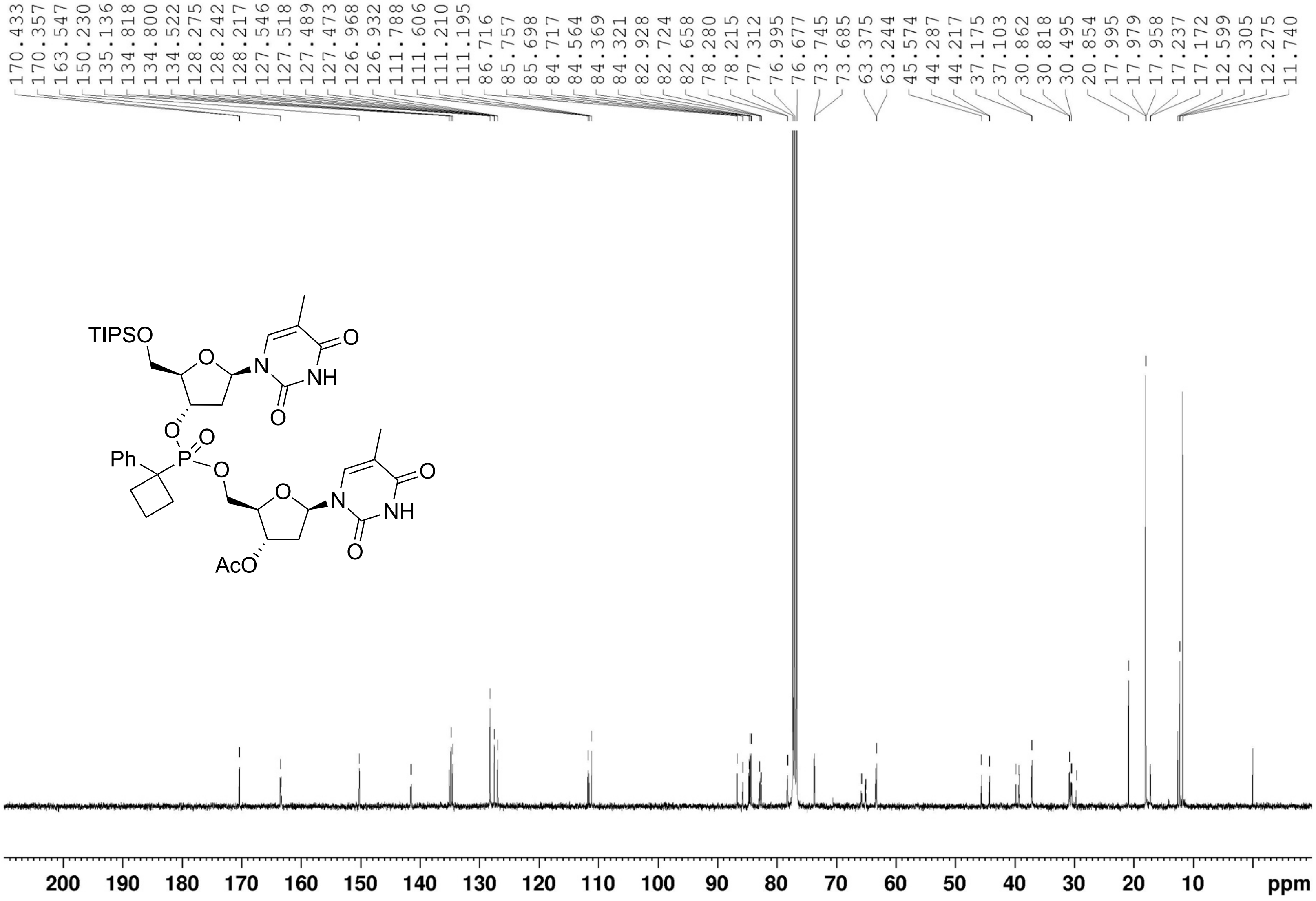


$^{13}$C NMR (100.6 MHz, $CDCl_3$) spectrum of **3ac**

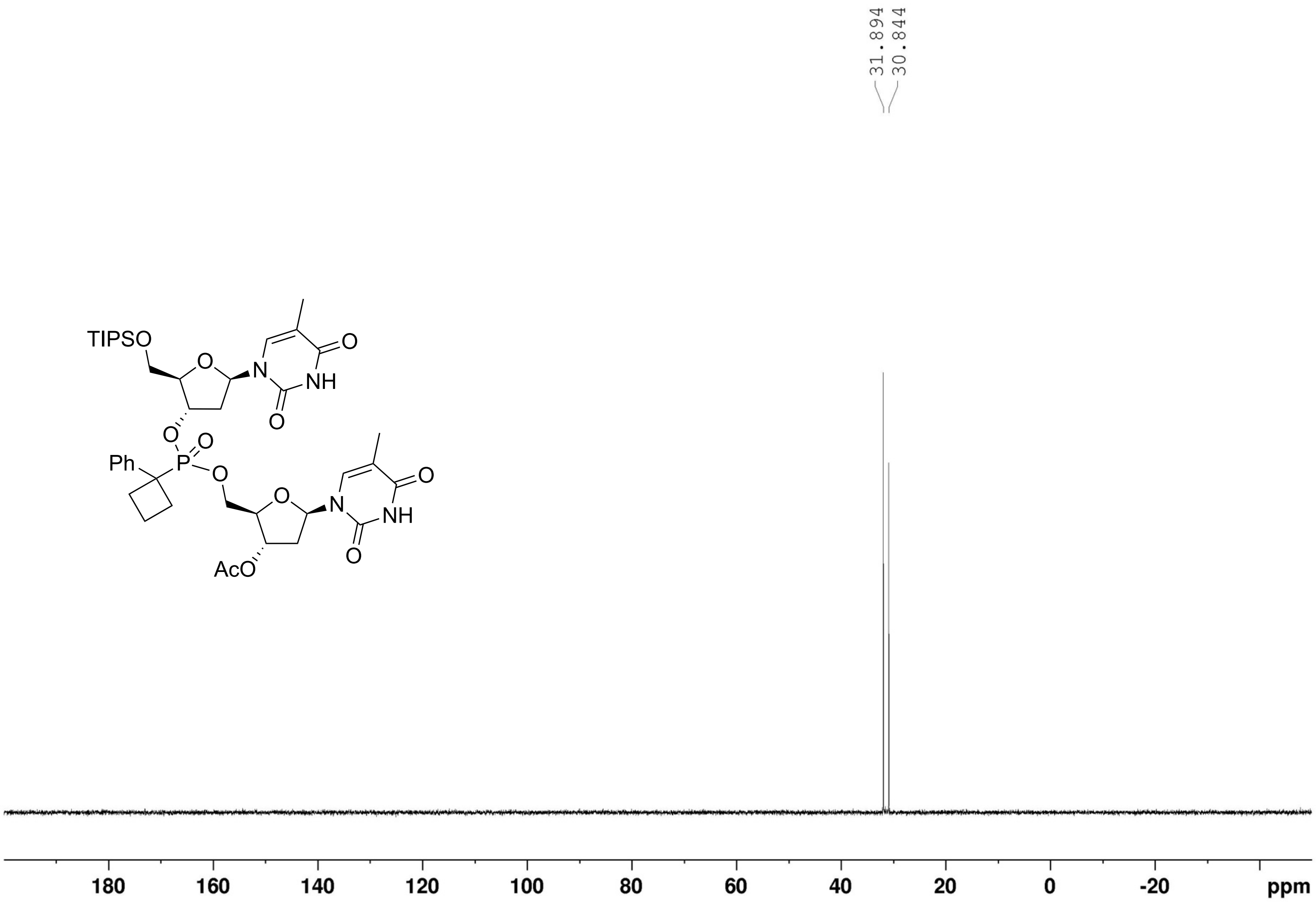


$^{31}$P NMR (162 MHz, $CDCl_3$) spectrum of **3ac**

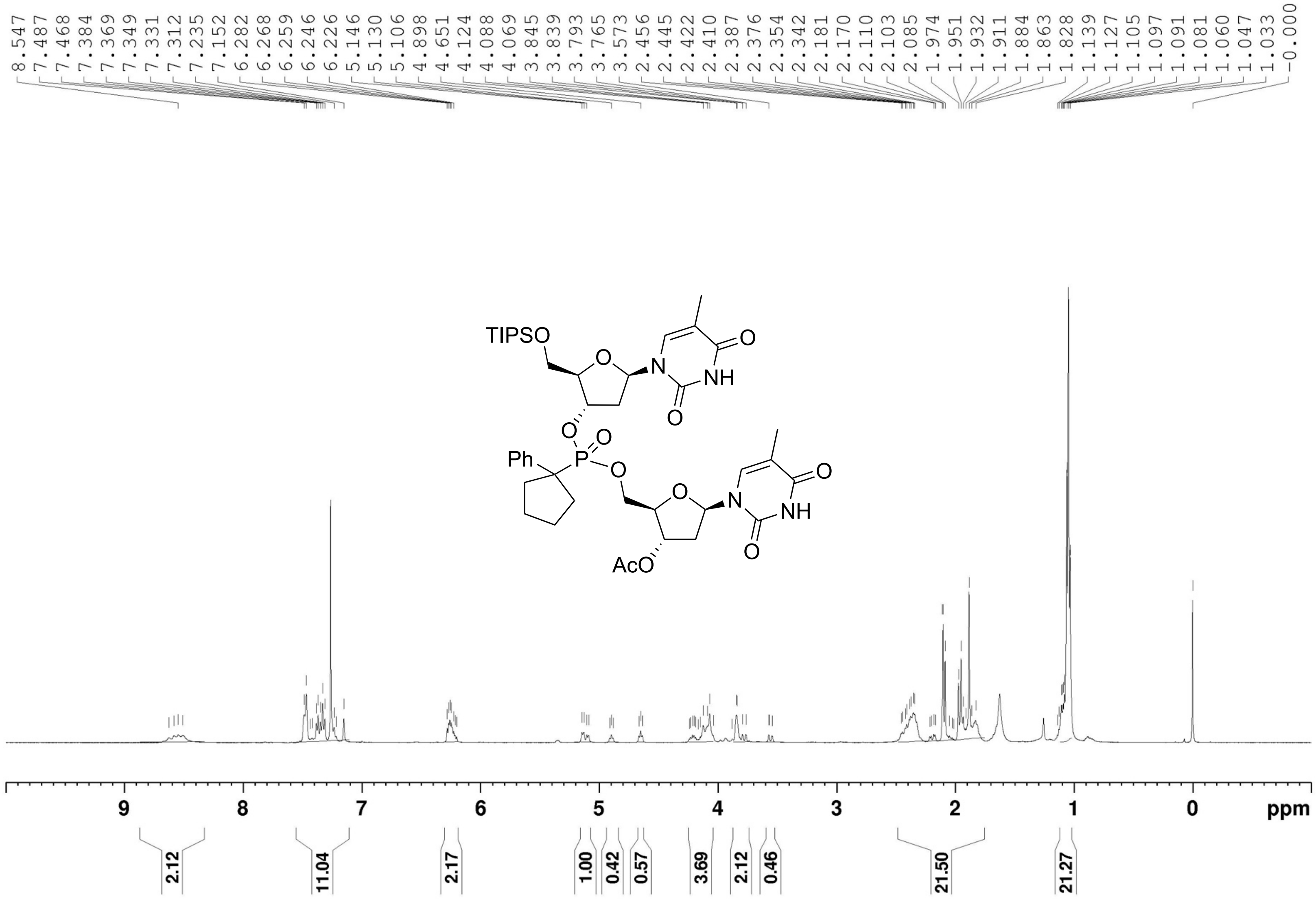


$^{1}$H NMR (400 MHz, $CDCl_3$) spectrum of **3ad**

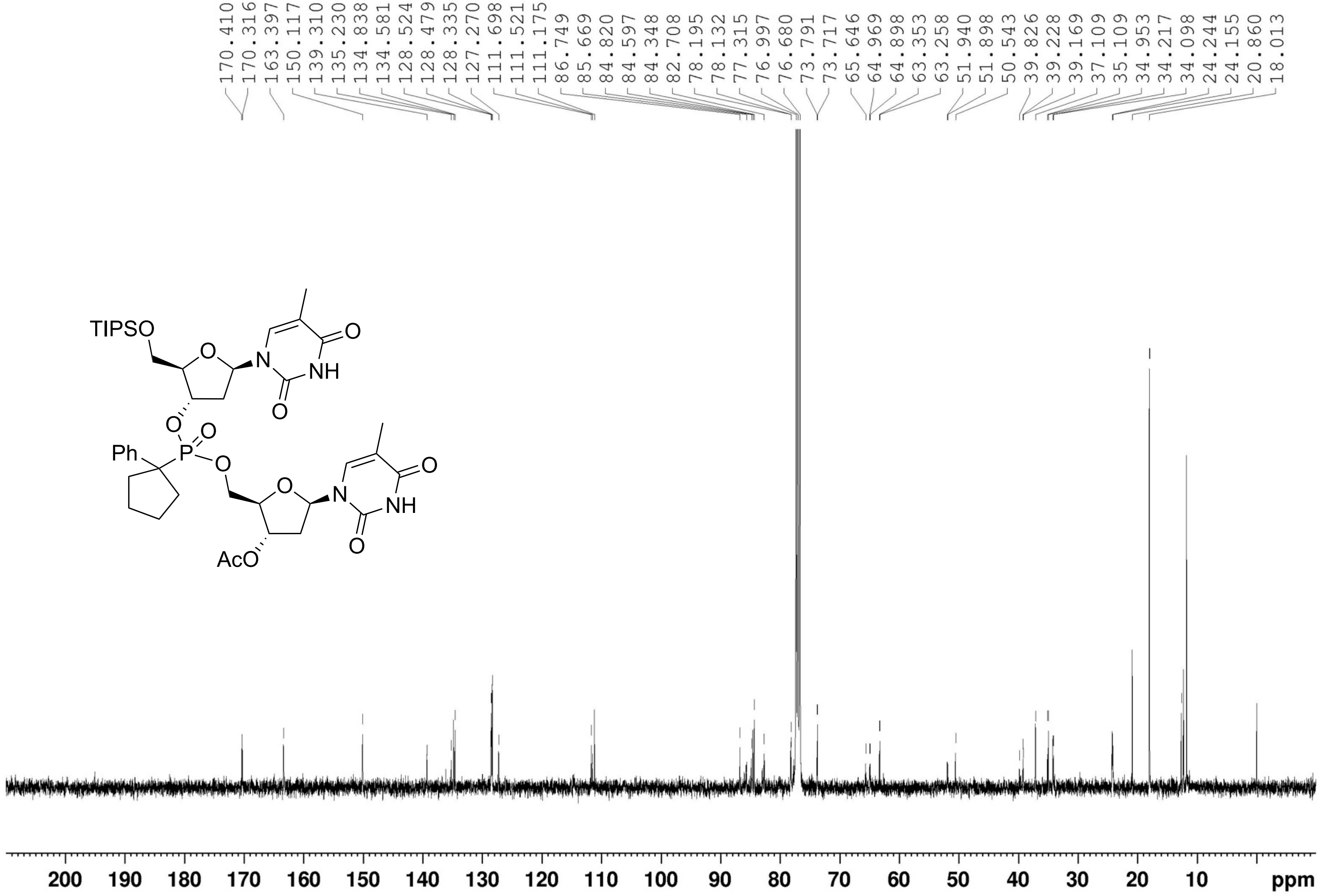


$^{13}$C NMR (100.6 MHz, $CDCl_3$) spectrum of **3ad**

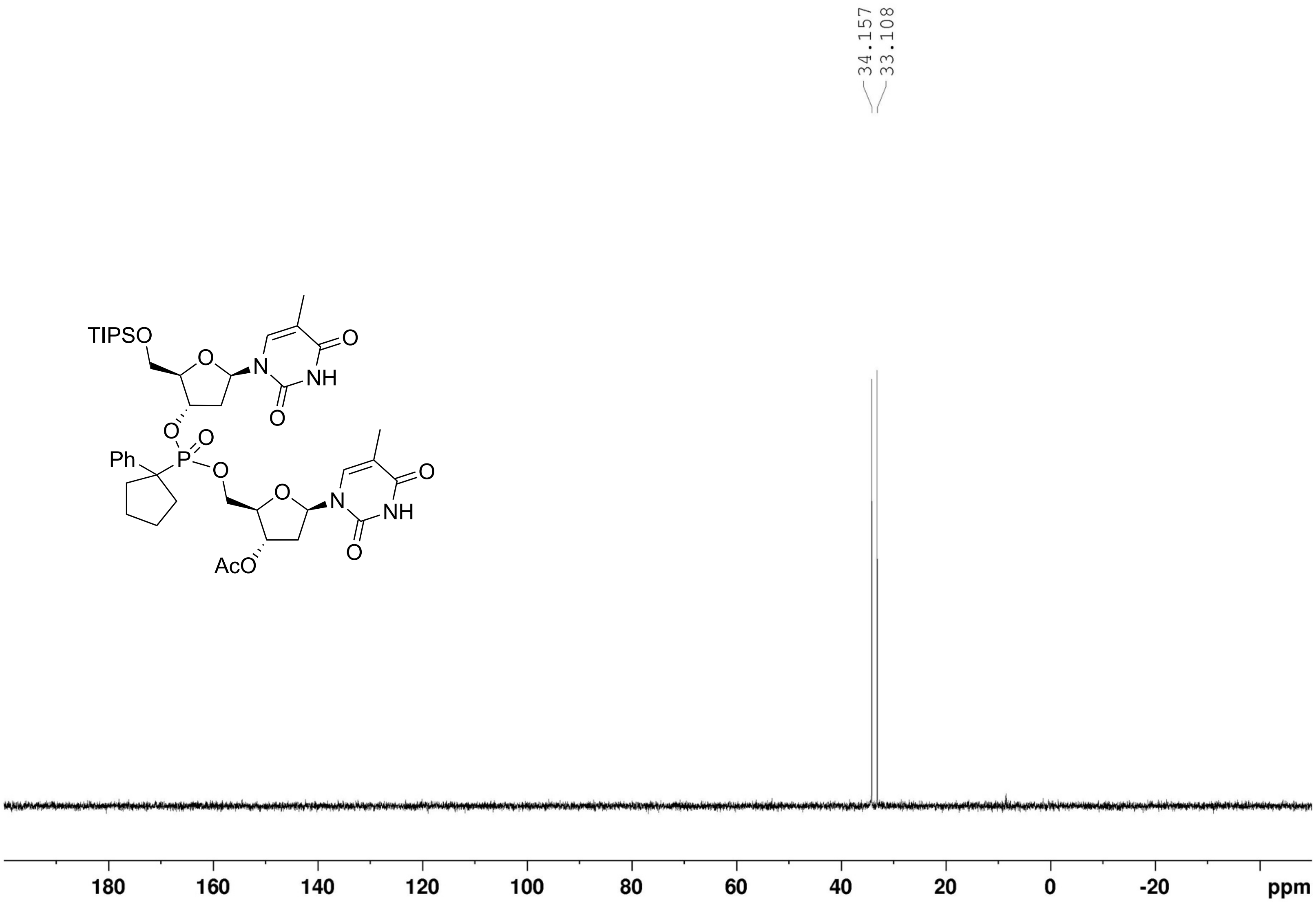


$^{31}$P NMR (162 MHz, $CDCl_3$) spectrum of **3ad**

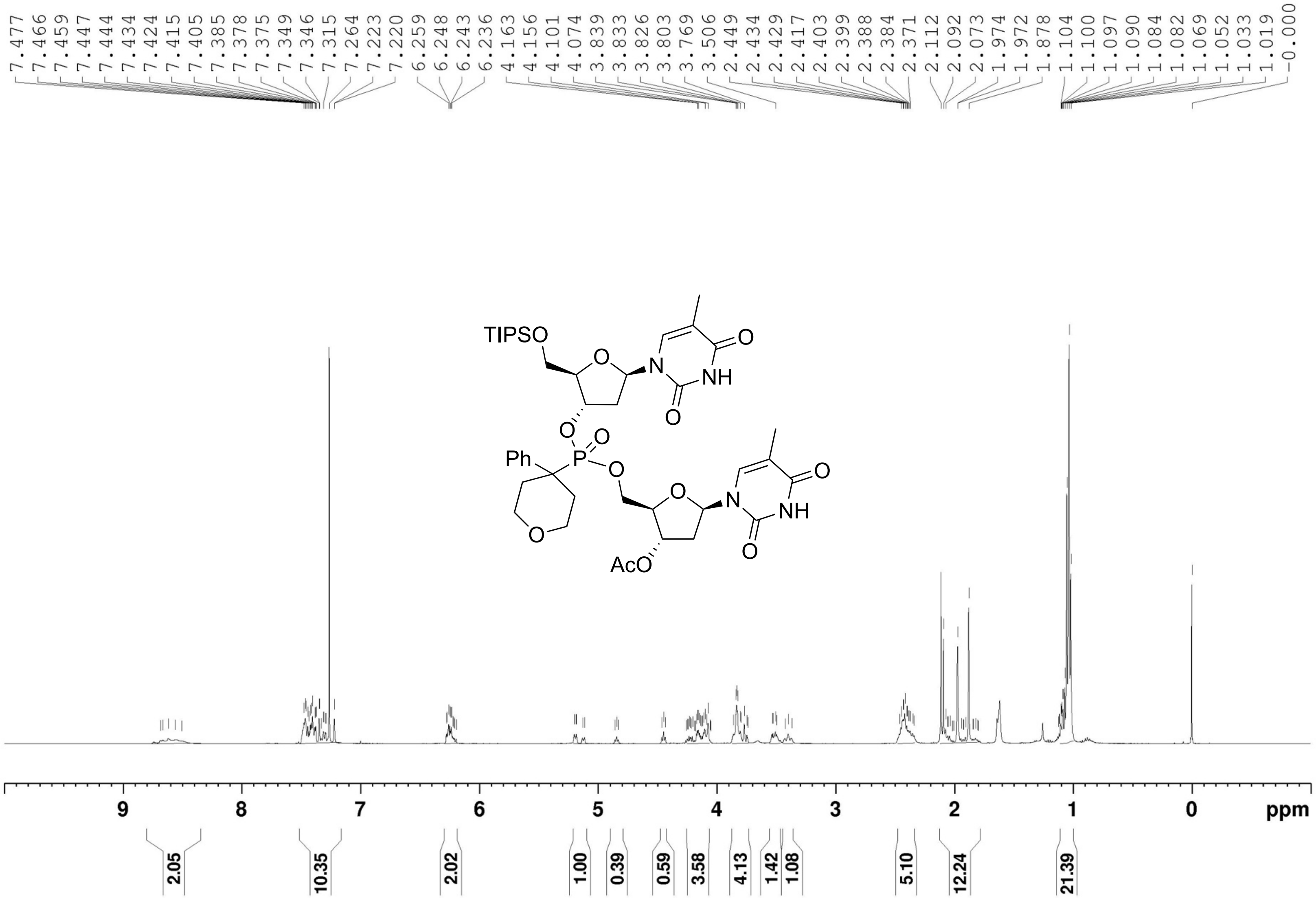


$^{1}$H NMR (400 MHz, $CDCl_3$) spectrum of **3ae**

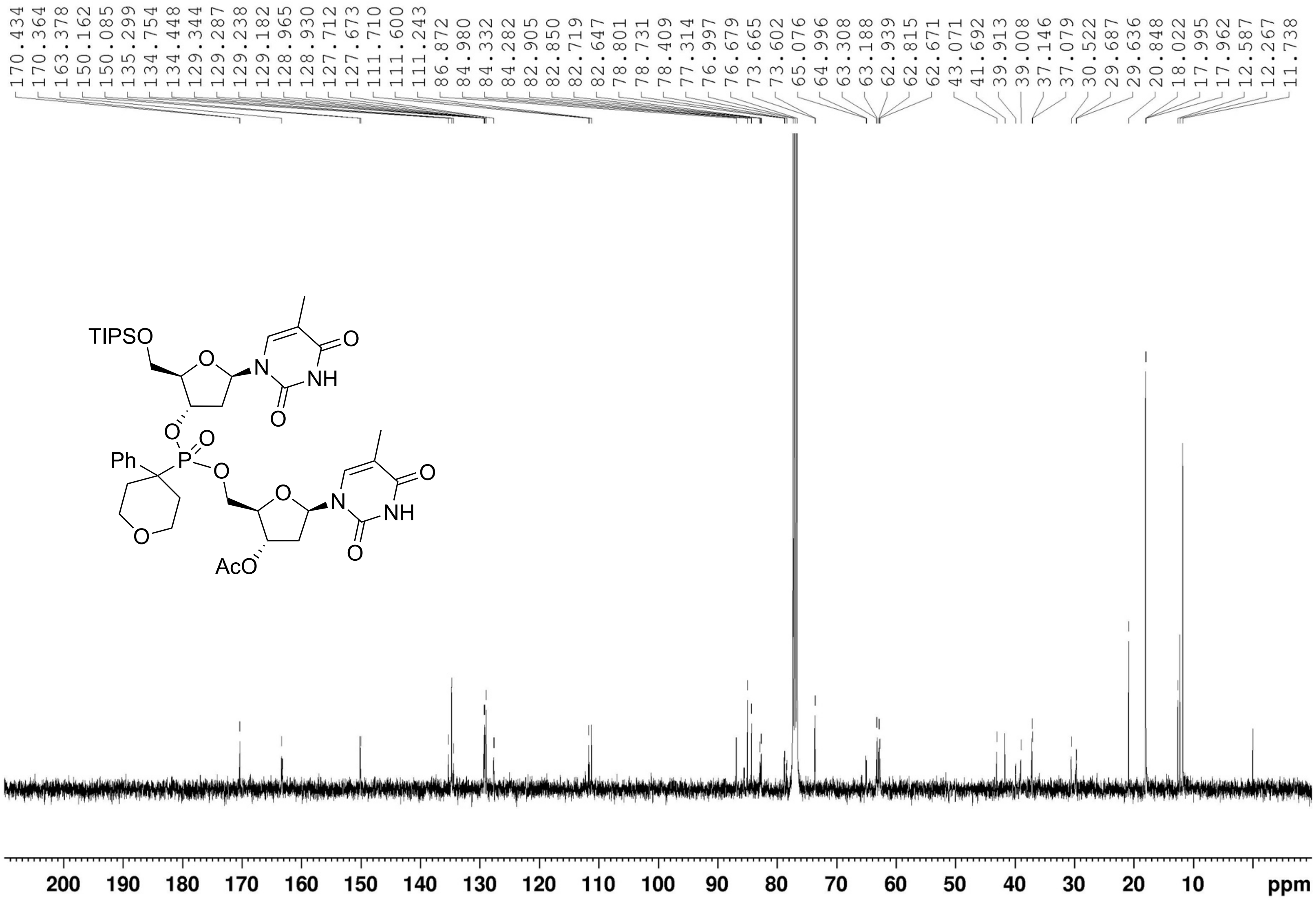


$^{13}C$ NMR (100.6 MHz, $CDCl_3$) spectrum of **3ae**

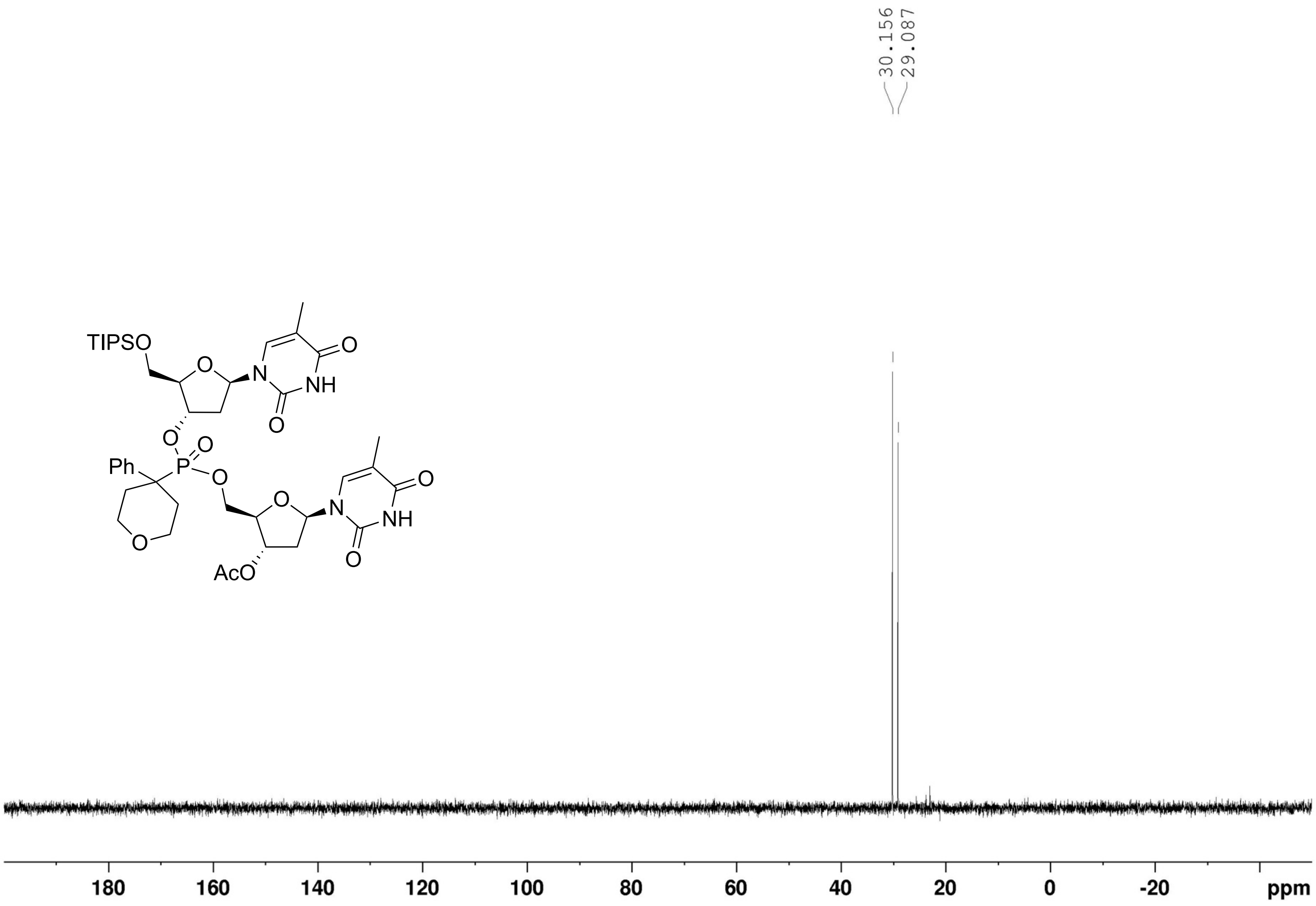


$^{31}$P NMR (162 MHz, $CDCl_3$) spectrum of **3ae**

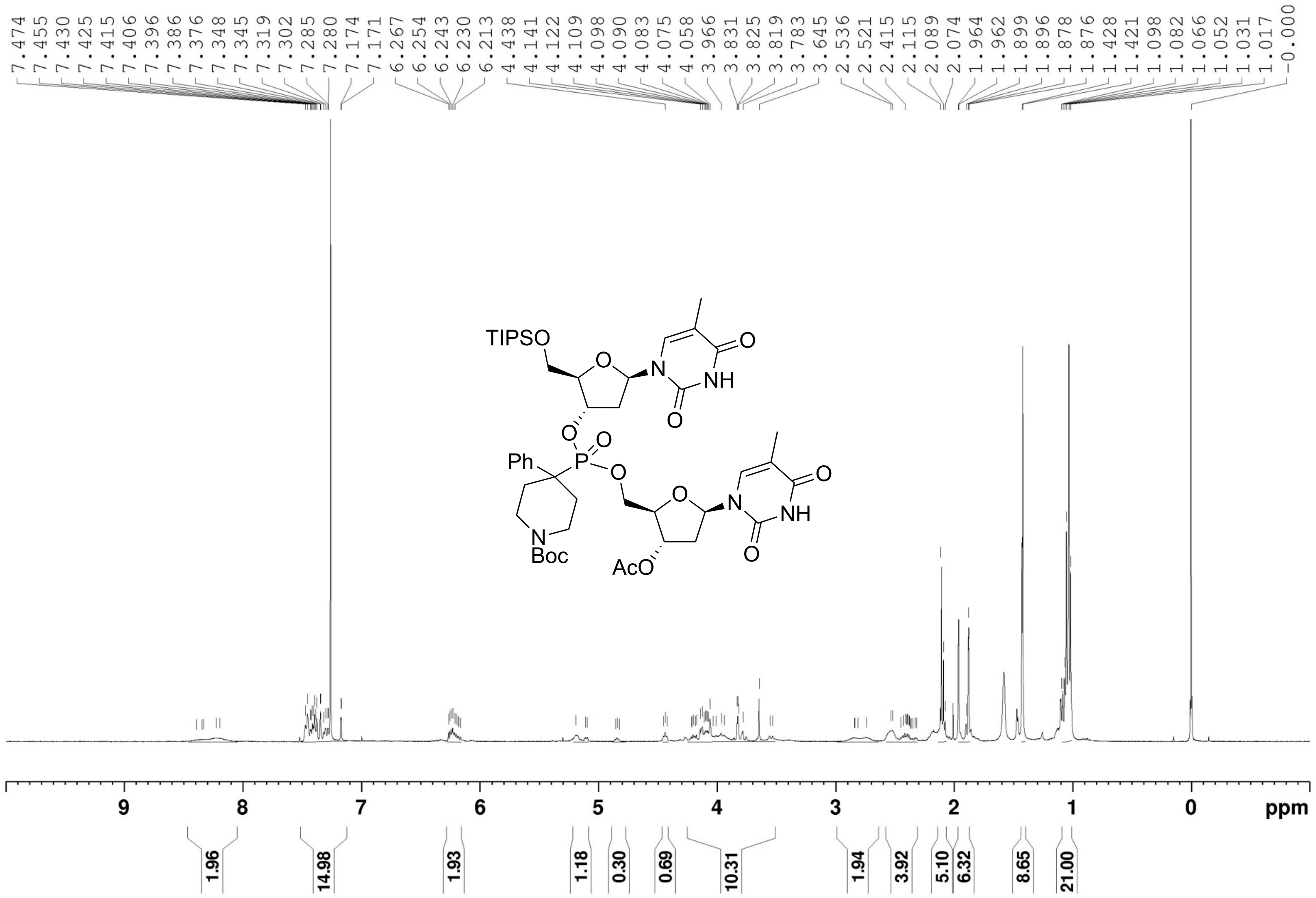

$^{1}$H NMR (400 MHz, $CDCl_3$) spectrum of **3af**

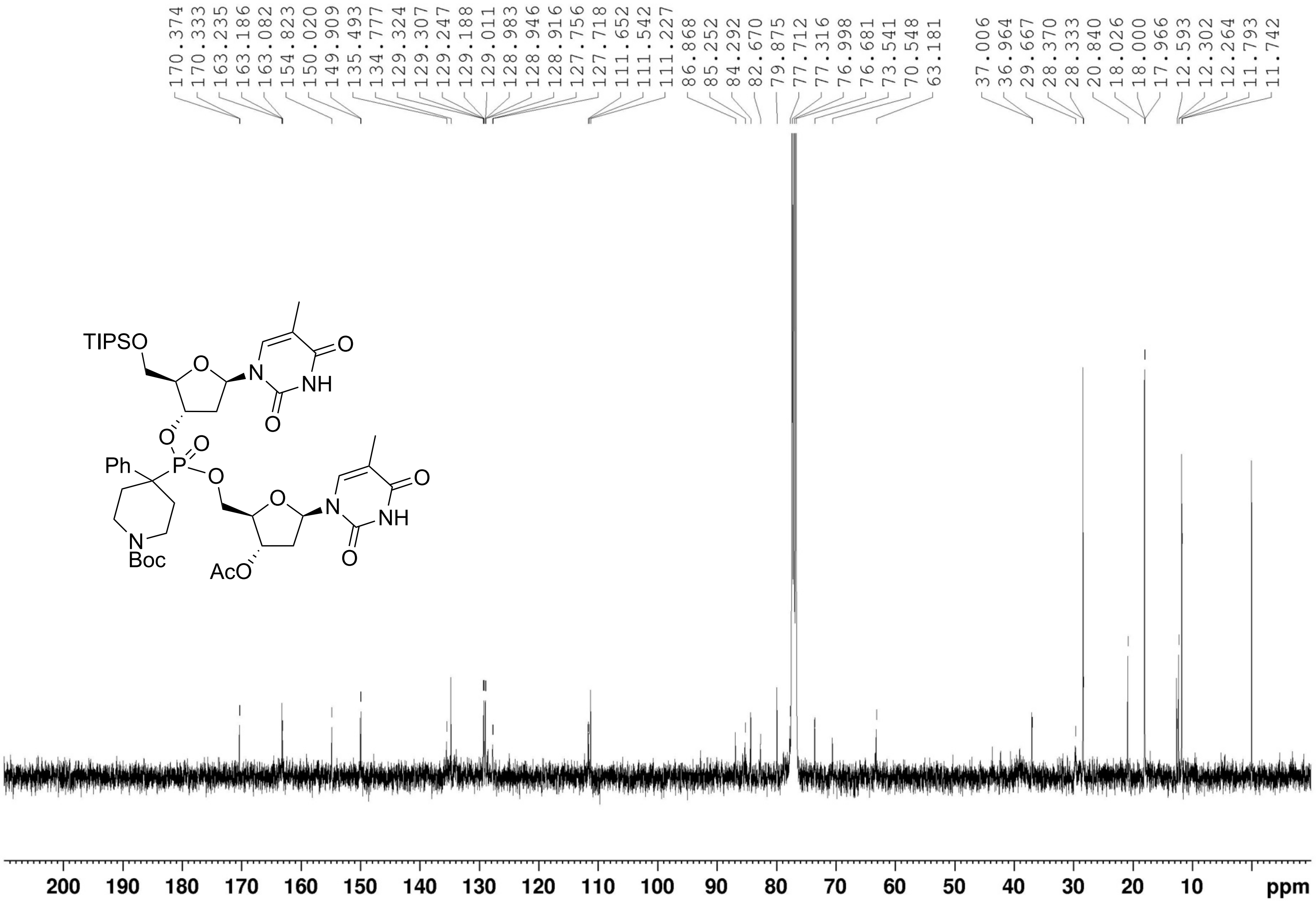


$^{13}$C NMR (100.6 MHz, $CDCl_3$) spectrum of **3af**

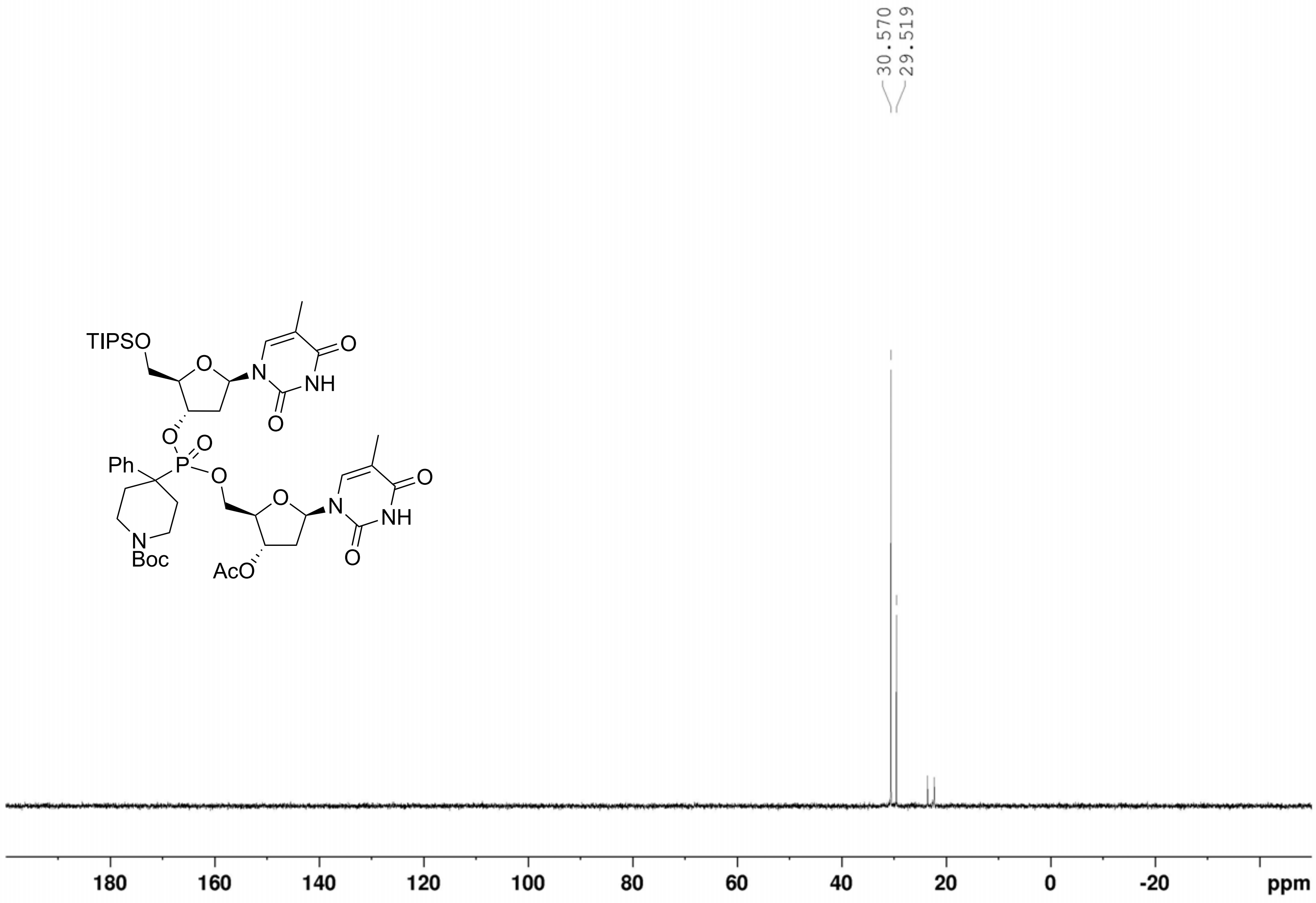


$^{31}P$ NMR (162 MHz, $CDCl_3$) spectrum of **3af**

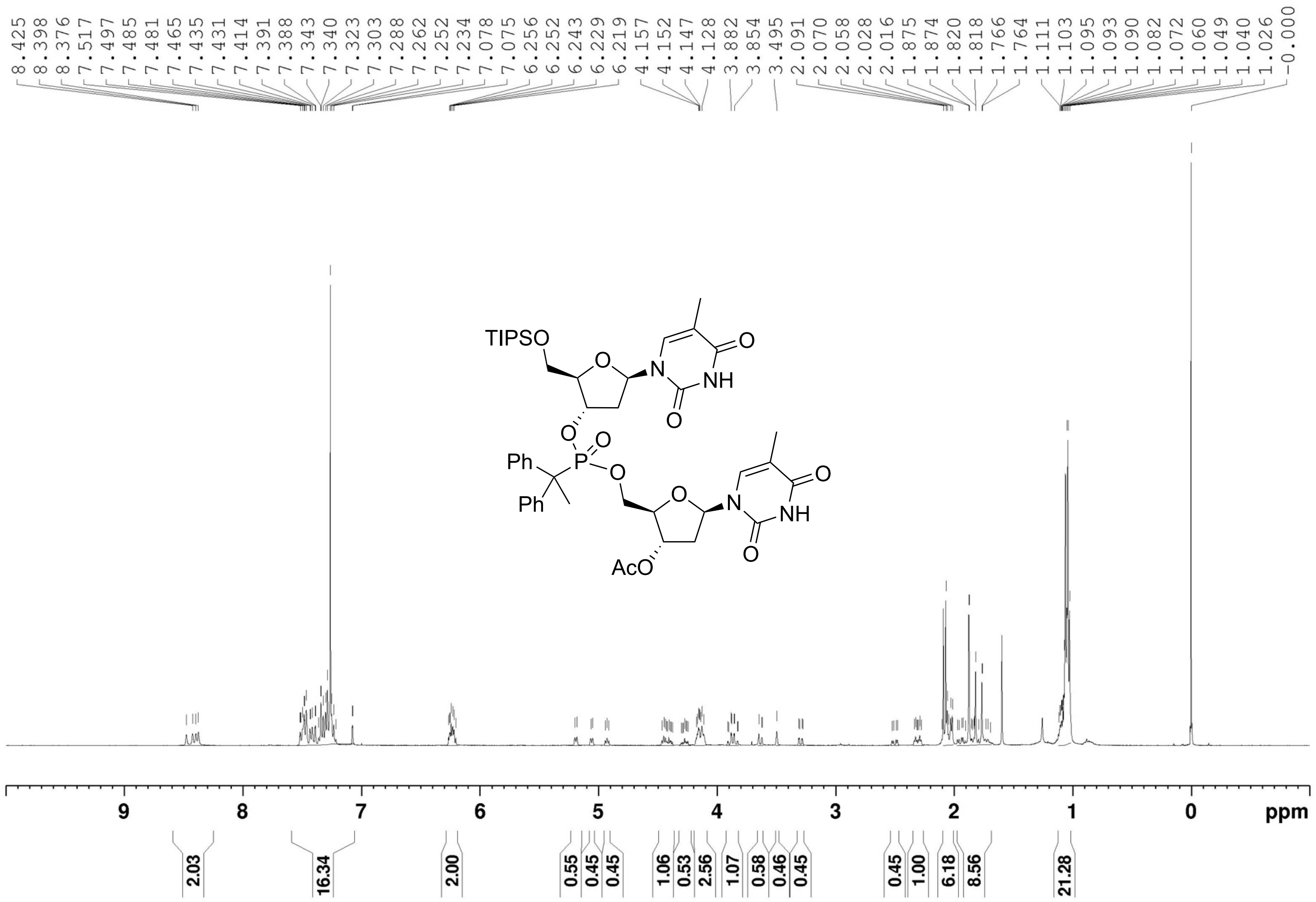


$^1$H NMR (400 MHz, CDCl$_3$) spectrum of **3ag**

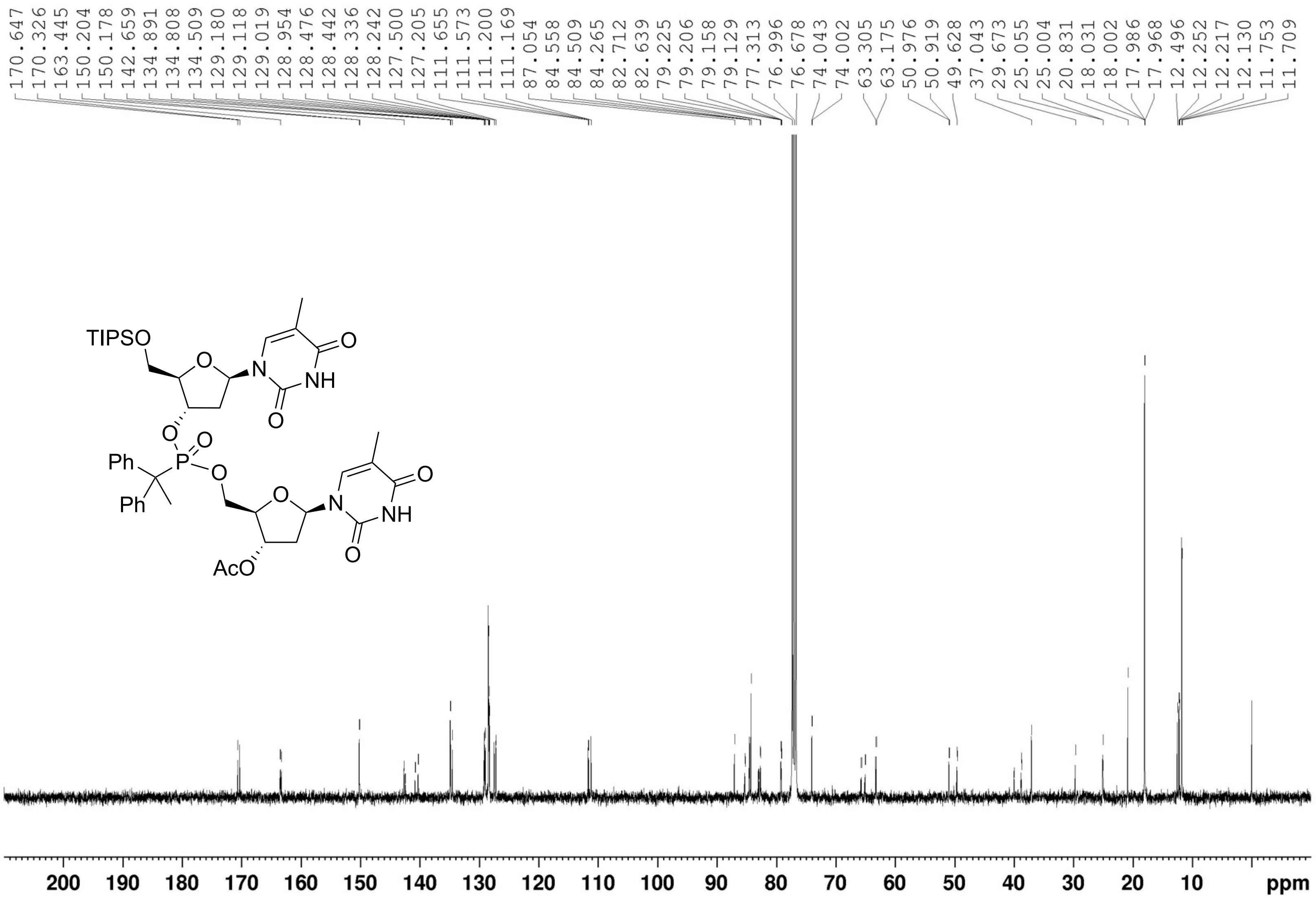


$^{13}C$ NMR (100.6 MHz, $CDCl_3$) spectrum of **3ag**

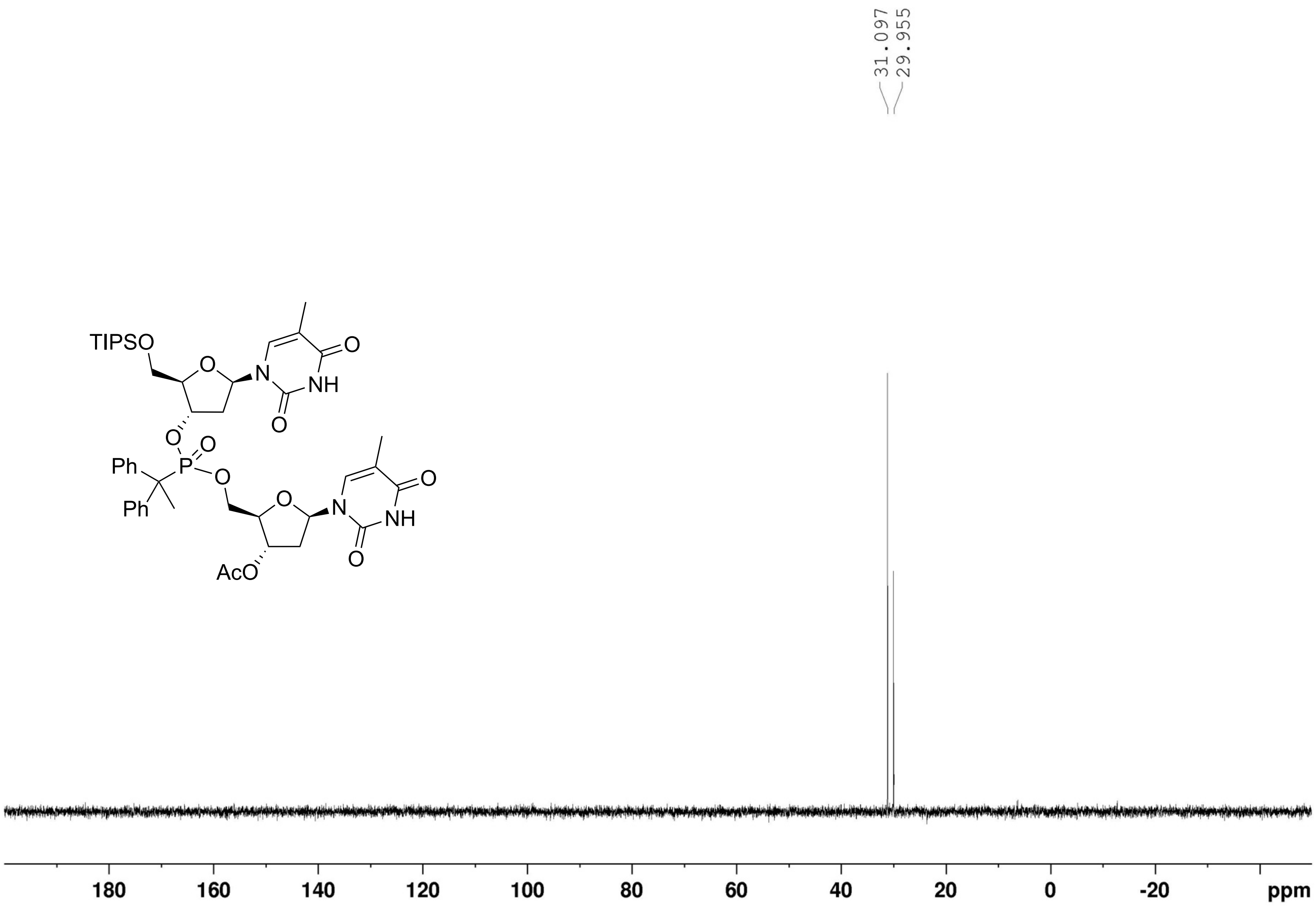


$^{31}P$ NMR (162 MHz, $CDCl_3$) spectrum of **3ag**

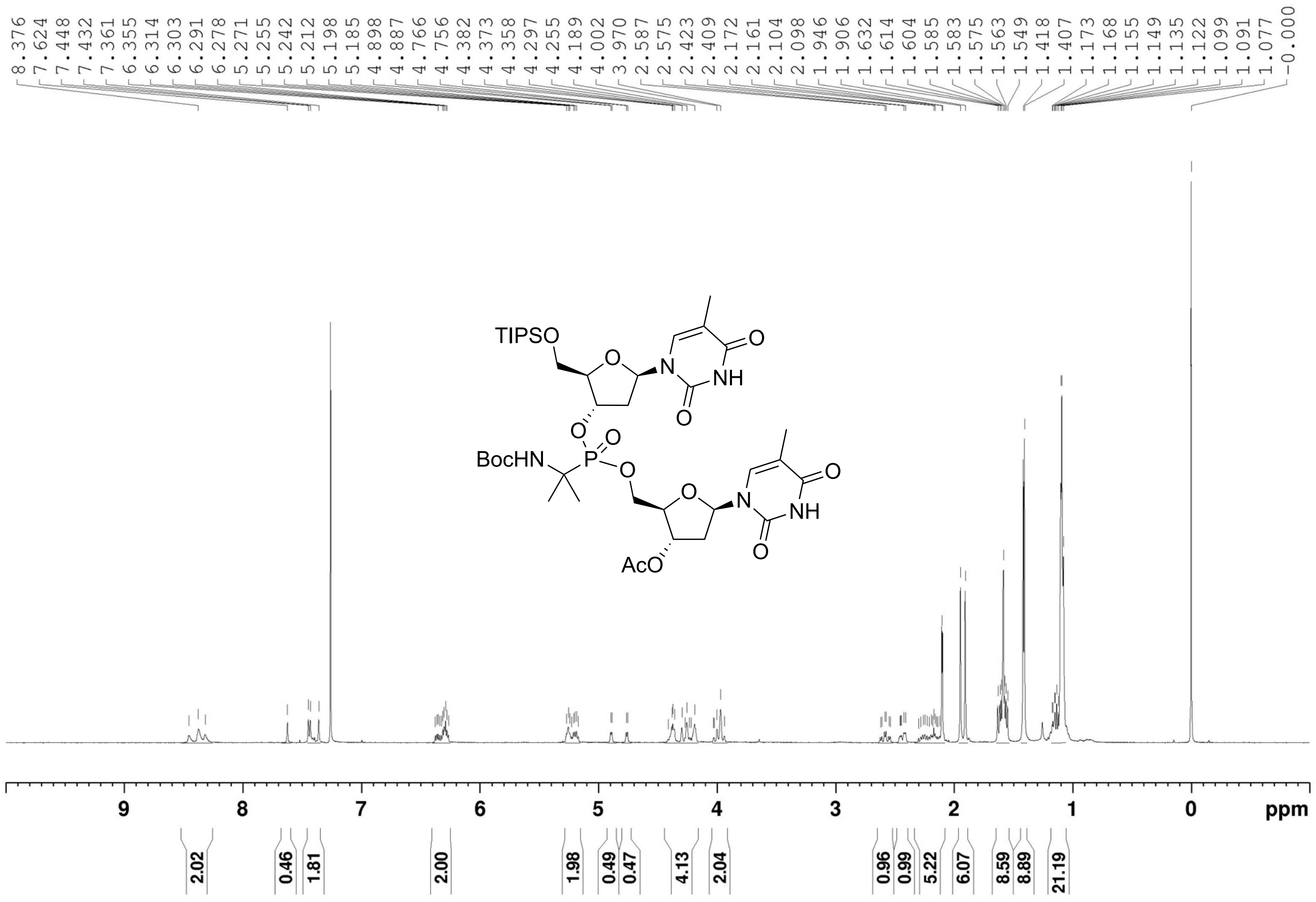

$^{1}$H NMR (400 MHz, CDCl3) spectrum of **3ah**

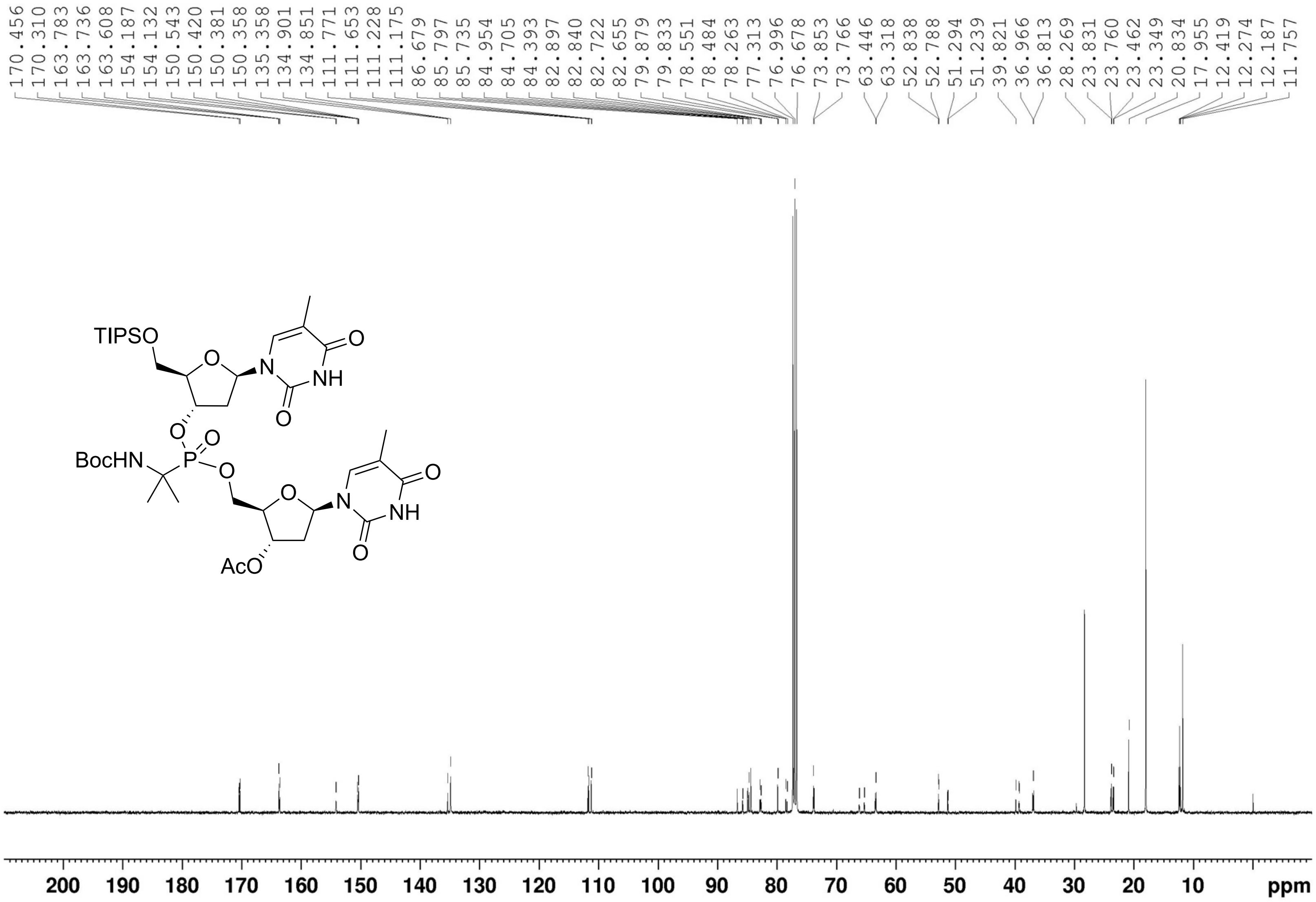


$^{13}C$ NMR (100.6 MHz, $CDCl_3$) spectrum of **3ah**

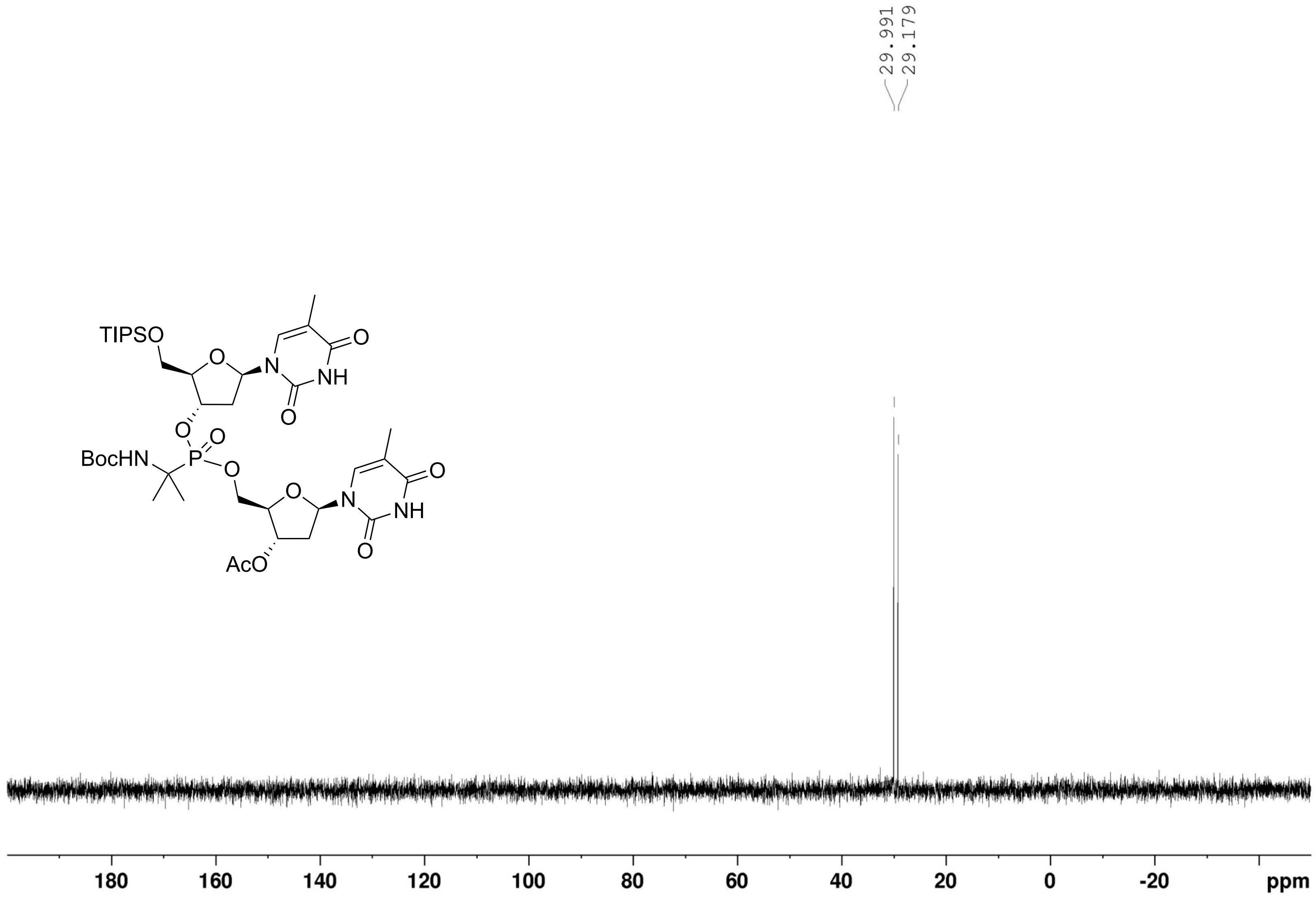


$^{31}P$ NMR (162 MHz, $CDCl_3$) spectrum of **3ah**

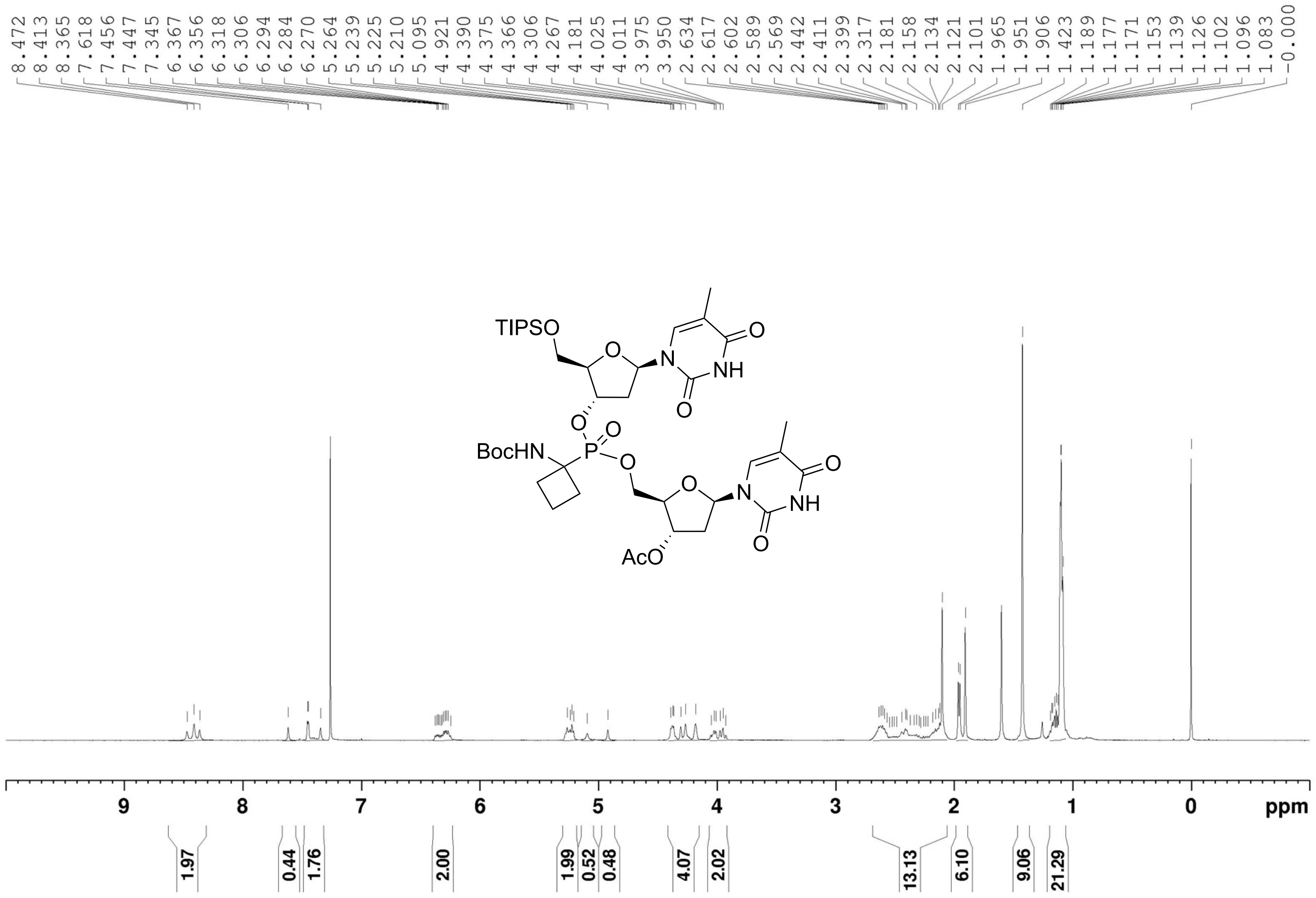


$^{1}$H NMR (400 MHz, $CDCl_3$) spectrum of **3ai**

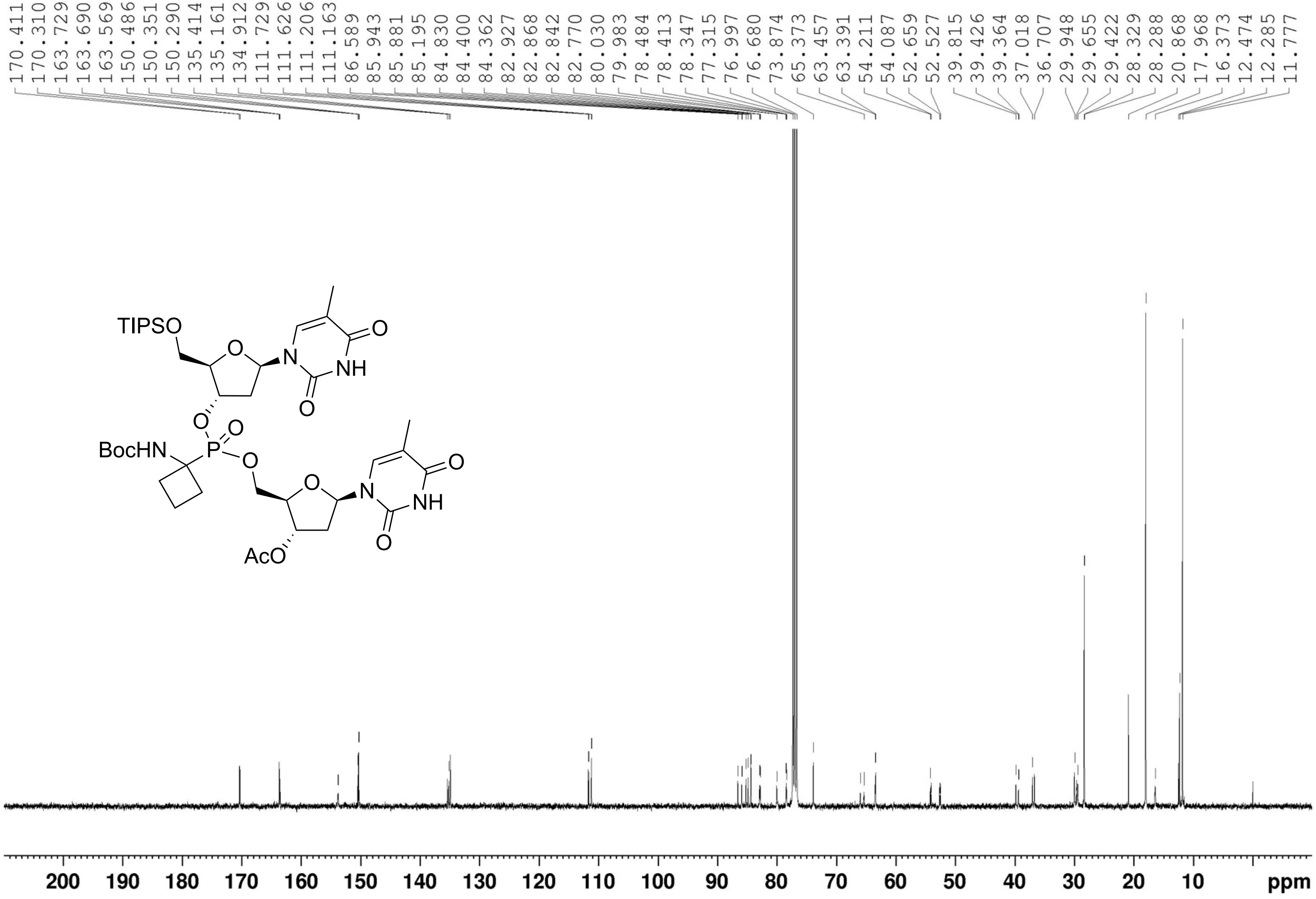


$^{13}$C NMR (100.6 MHz, $CDCl_3$) spectrum of **3ai**

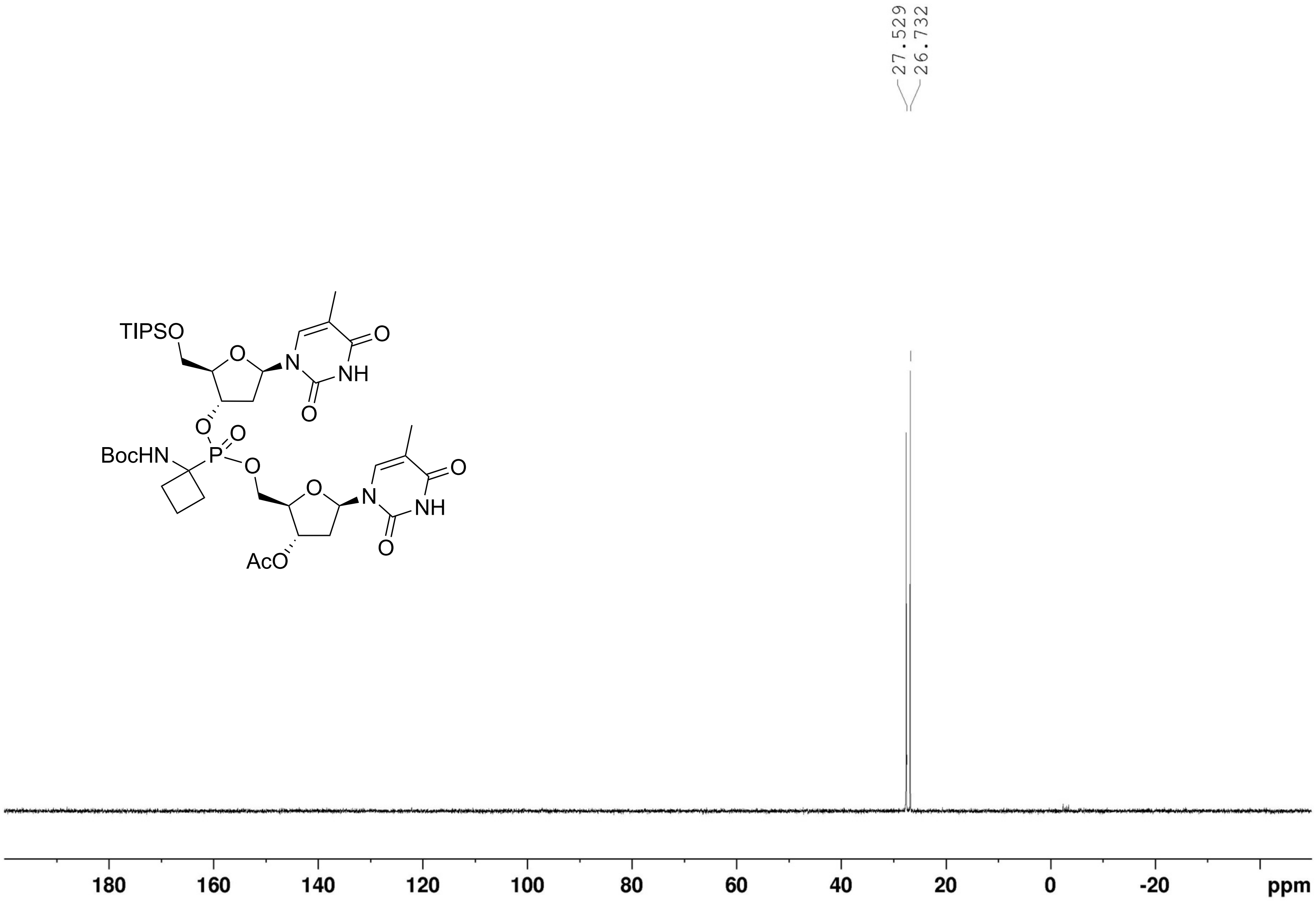


$^{31}P$ NMR (162 MHz, $CDCl_3$) spectrum of **3ai**

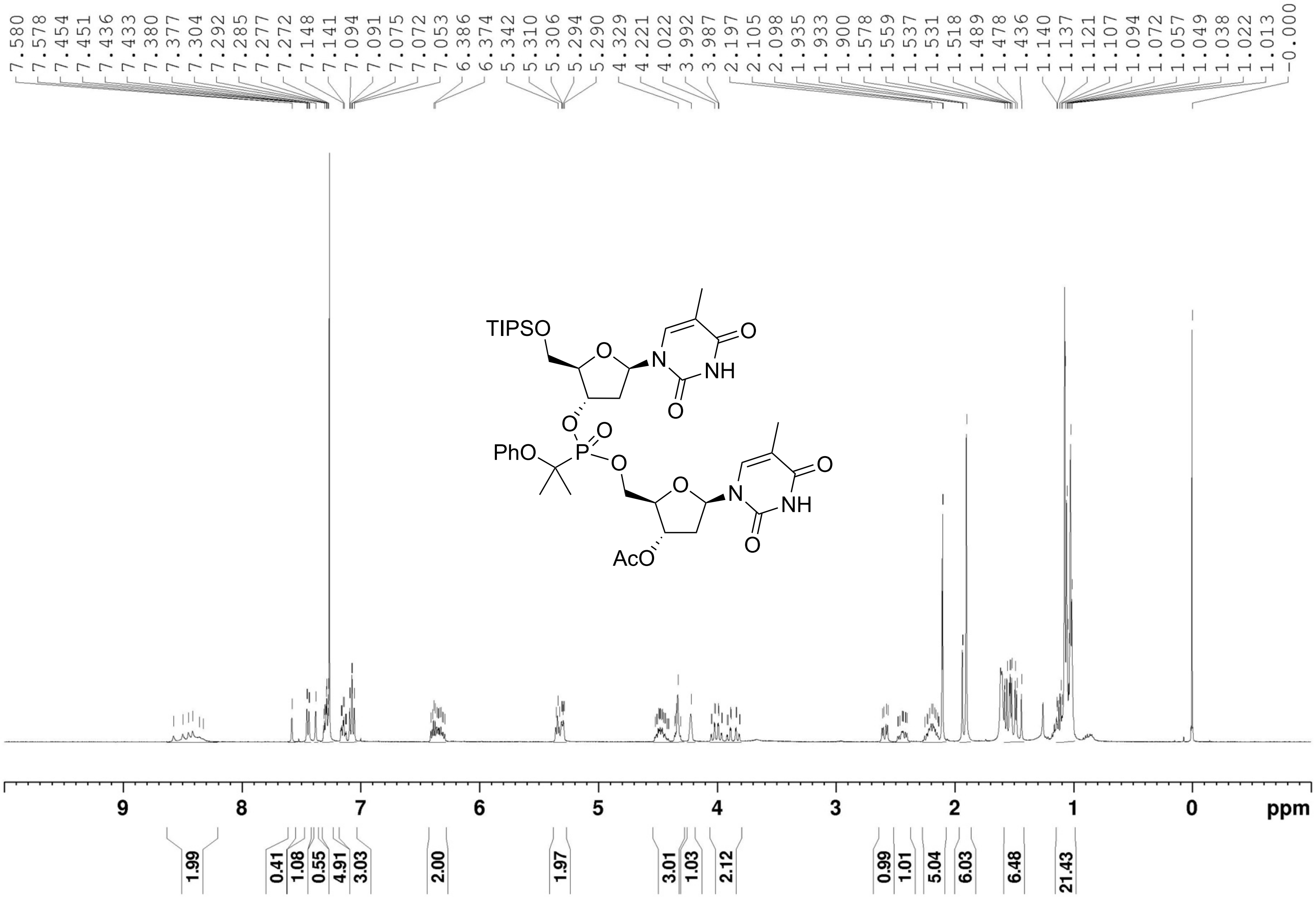


$^{1}$H NMR (400 MHz, $CDCl_3$) spectrum of **3aj**

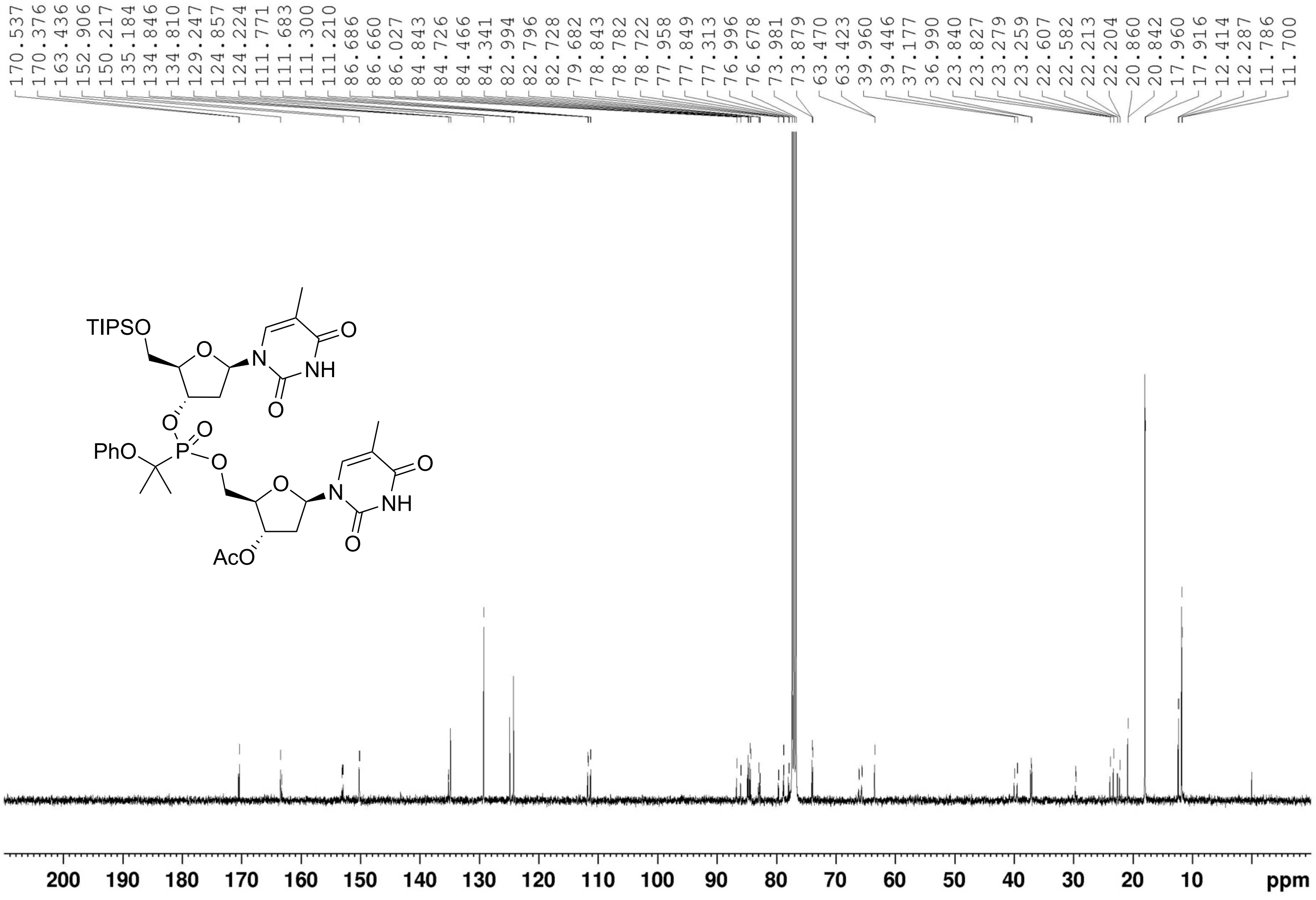


$^{13}$C NMR (100.6 MHz, $CDCl_3$) spectrum of **3aj**

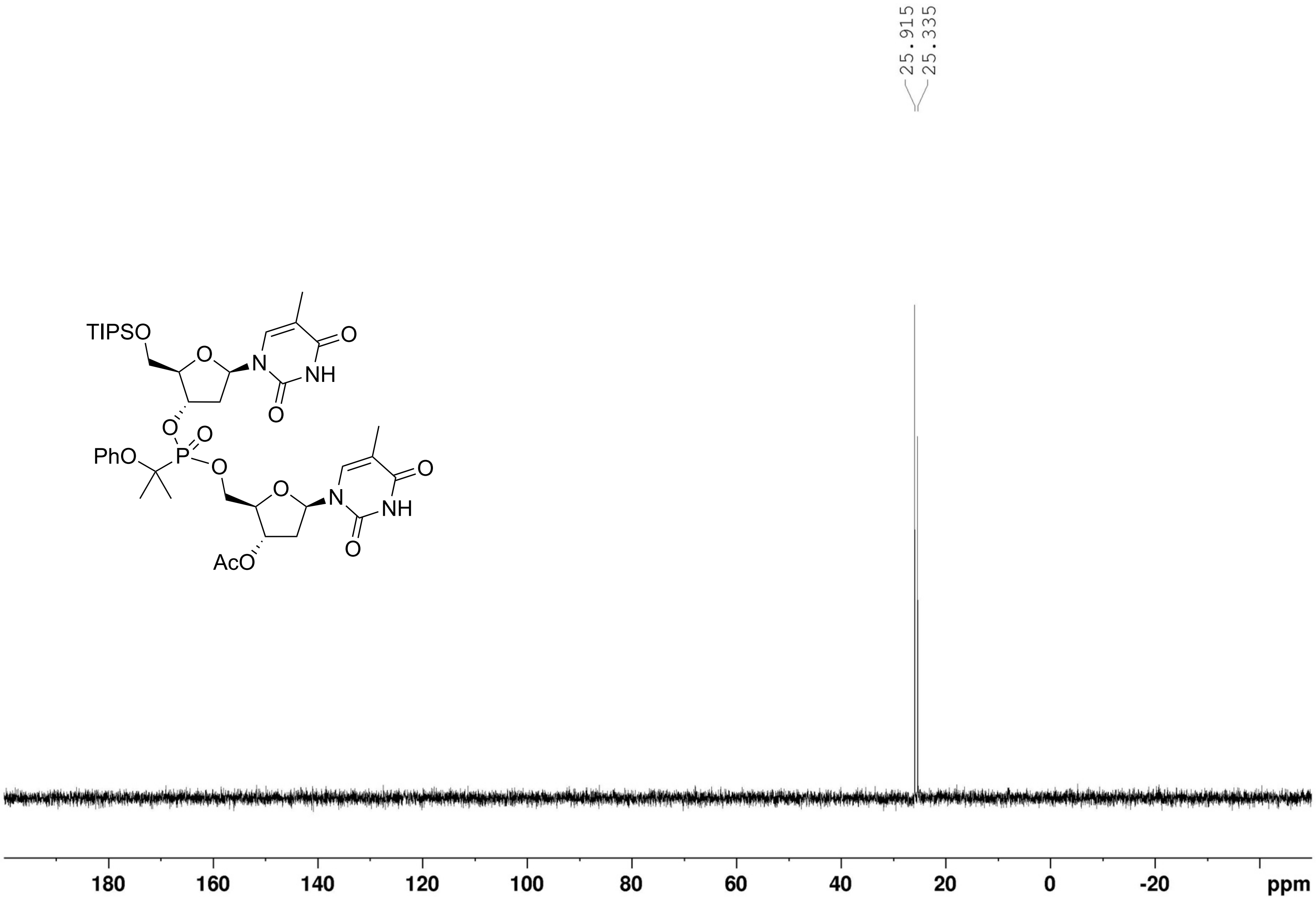


$^{31}$P NMR (162 MHz, $CDCl_3$) spectrum of **3aj**

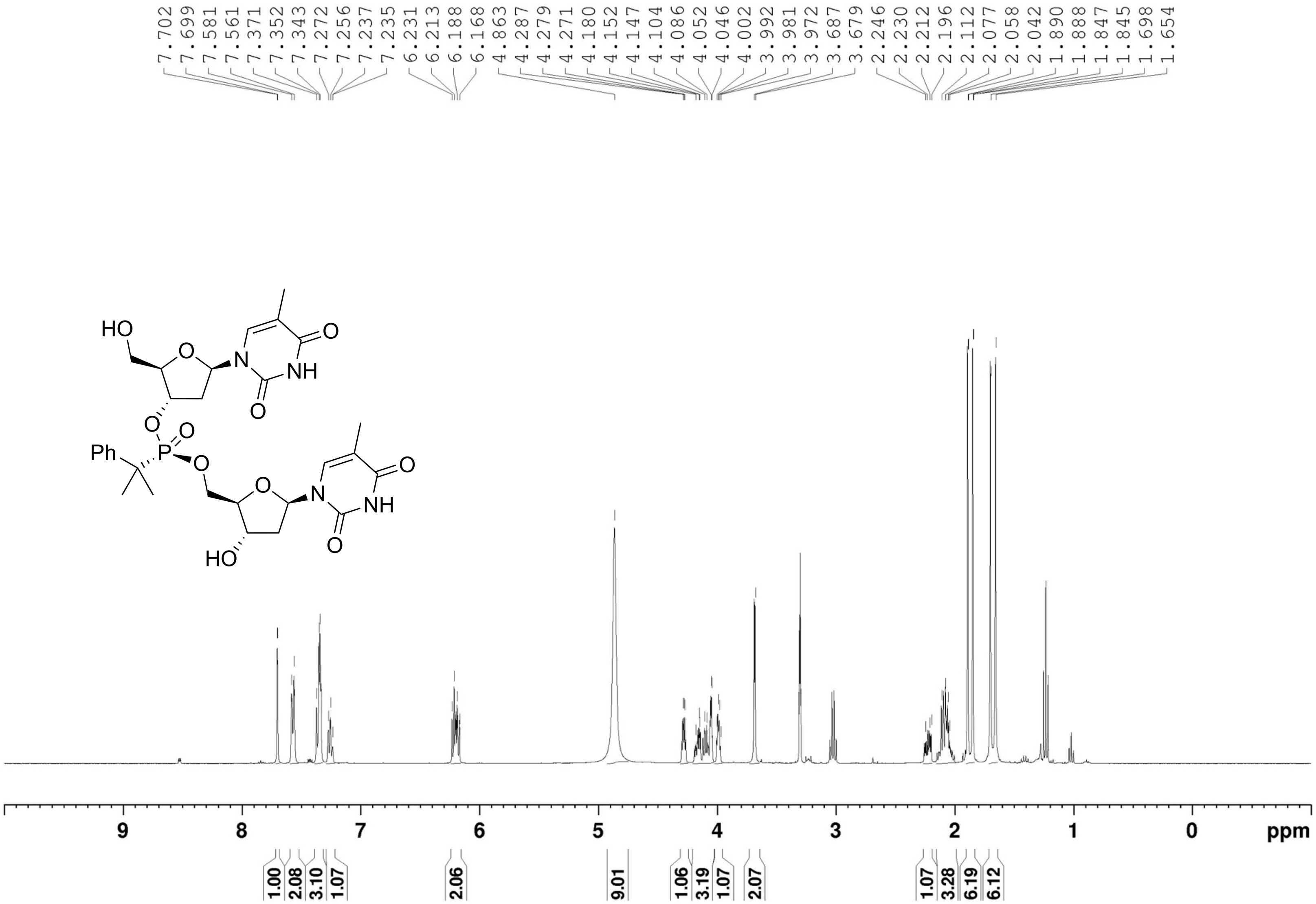


$^{1}$H NMR (400 MHz, $CD_3OD$) spectrum of **($S_P$)-3aa-1**

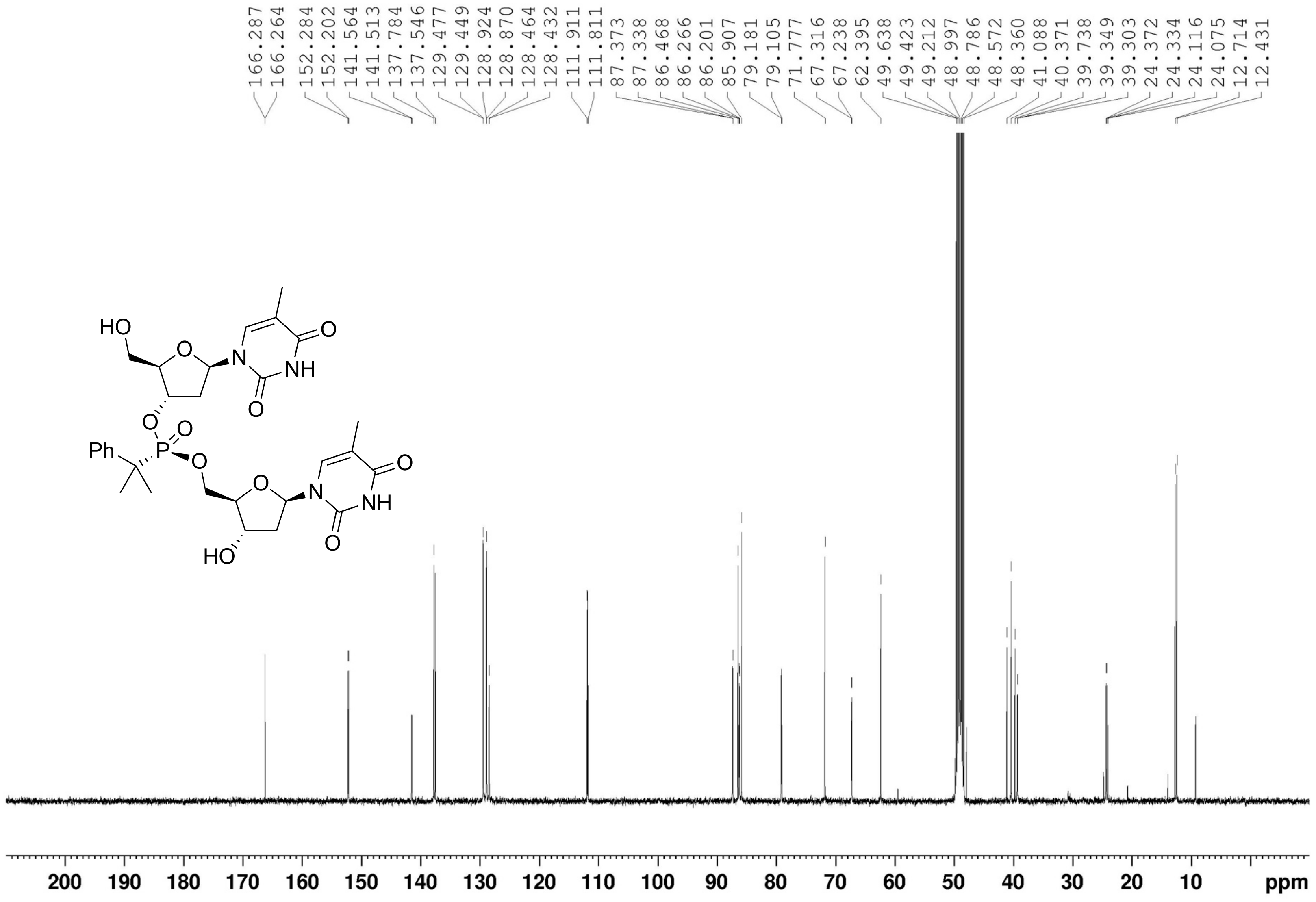


$^{13}$C NMR (100.6 MHz, $CD_3OD$) spectrum of **($S_P$)-3aa-1**

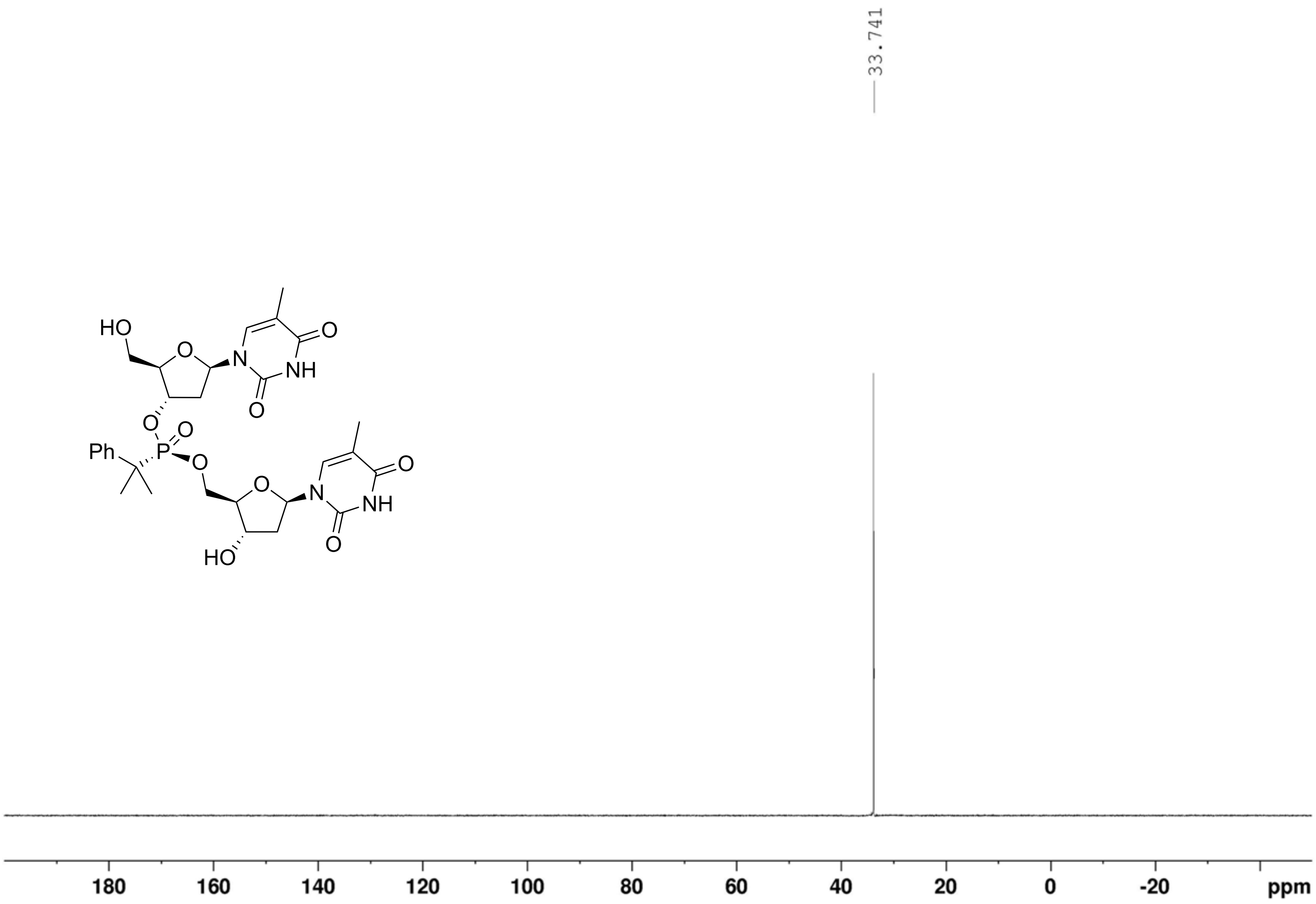


$^{31}$P NMR (162 MHz, $CD_3OD$) spectrum of **($S_P$)-3aa-1**

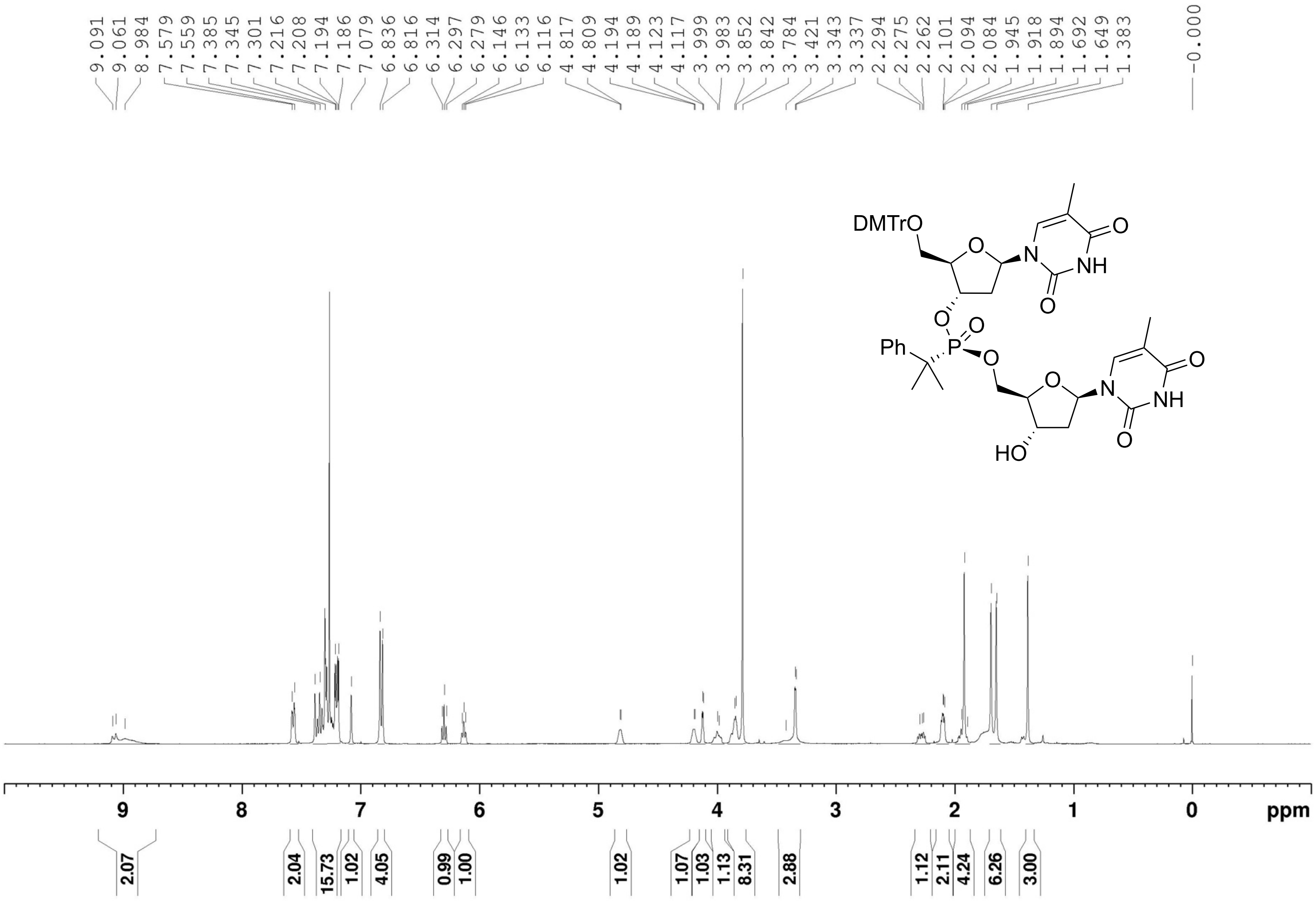


$^{1}$H NMR (400 MHz, $CDCl_3$) spectrum of **($S_P$)-3aa-2**

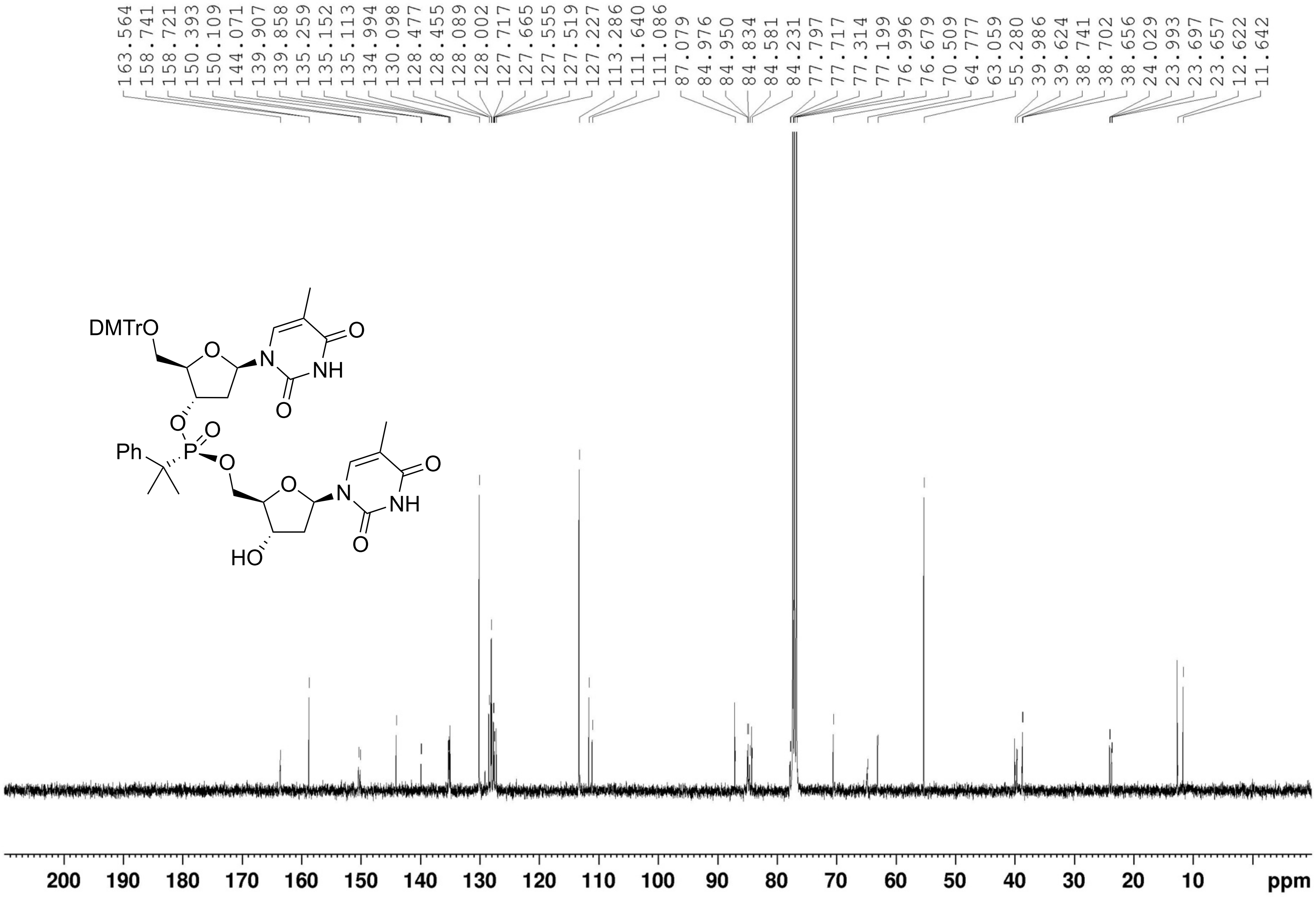


$^{13}$C NMR (100.6 MHz, $CDCl_3$) spectrum of **($S_P$)-3aa-2**

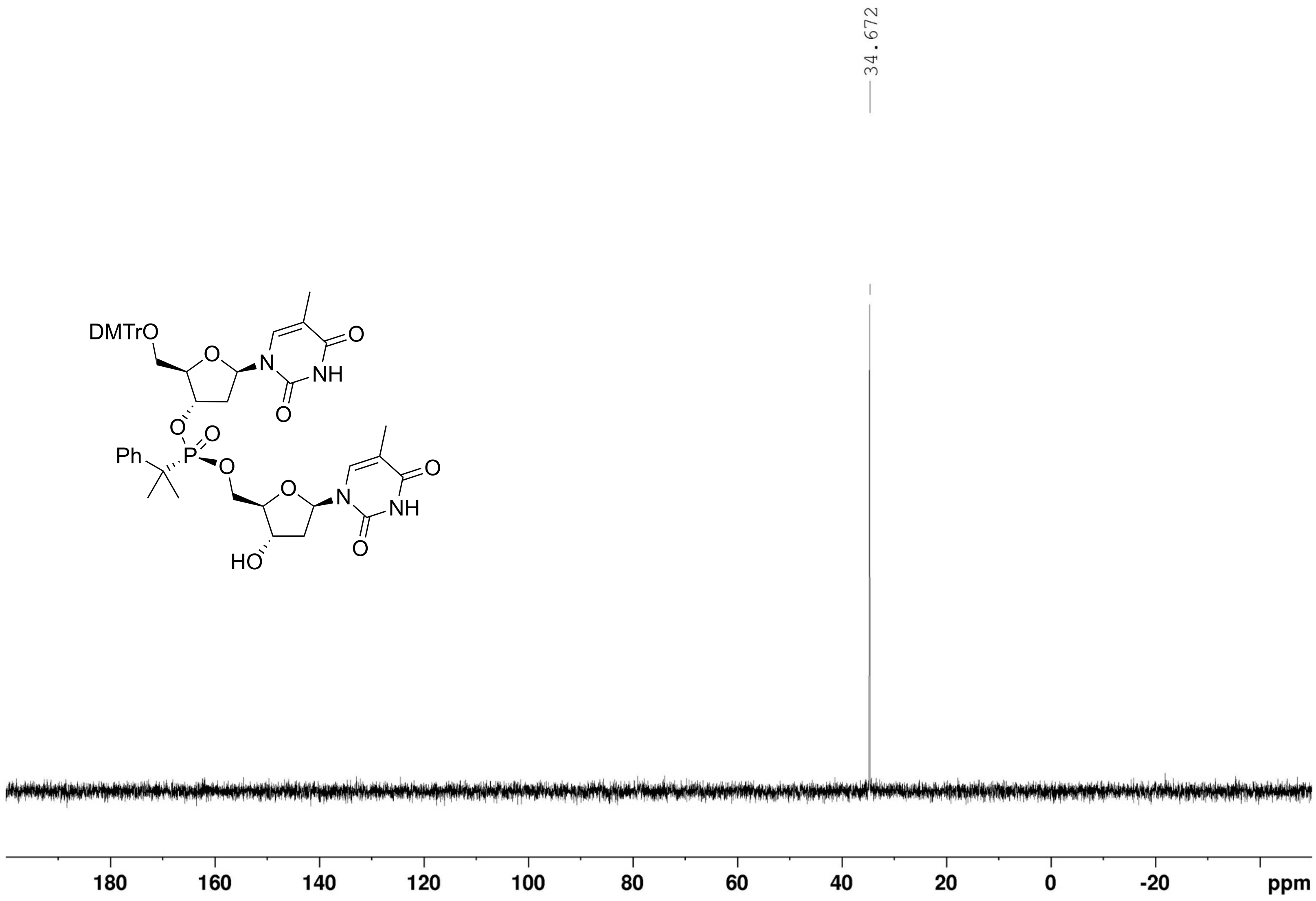


$^{31}P$ NMR (162 MHz, $CDCl_3$) spectrum of **($S_P$)-3aa-2**

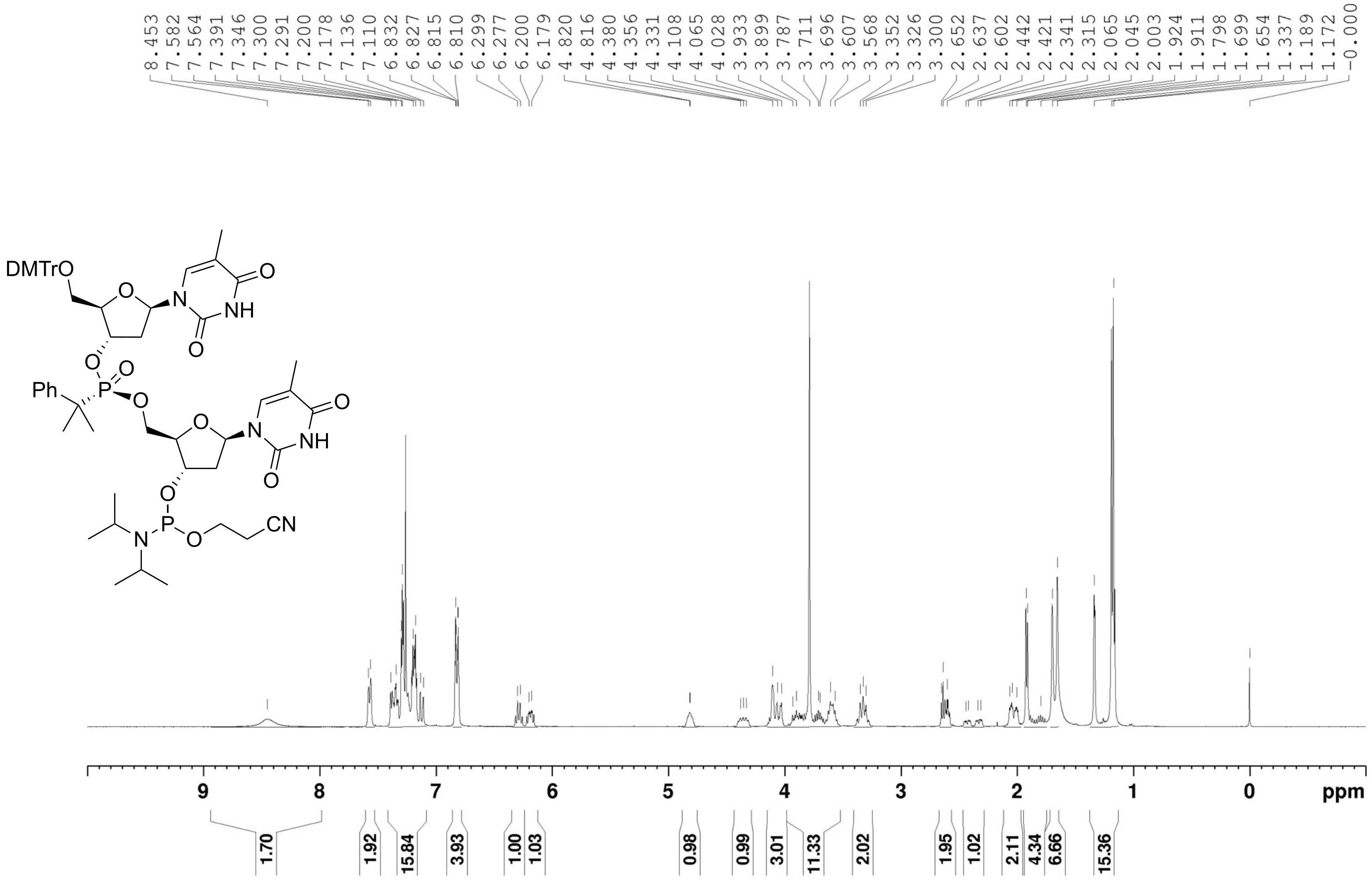


$^{1}H$ NMR (400 MHz, $CDCl_3$) spectrum of **($S_P$)-3aa-3**

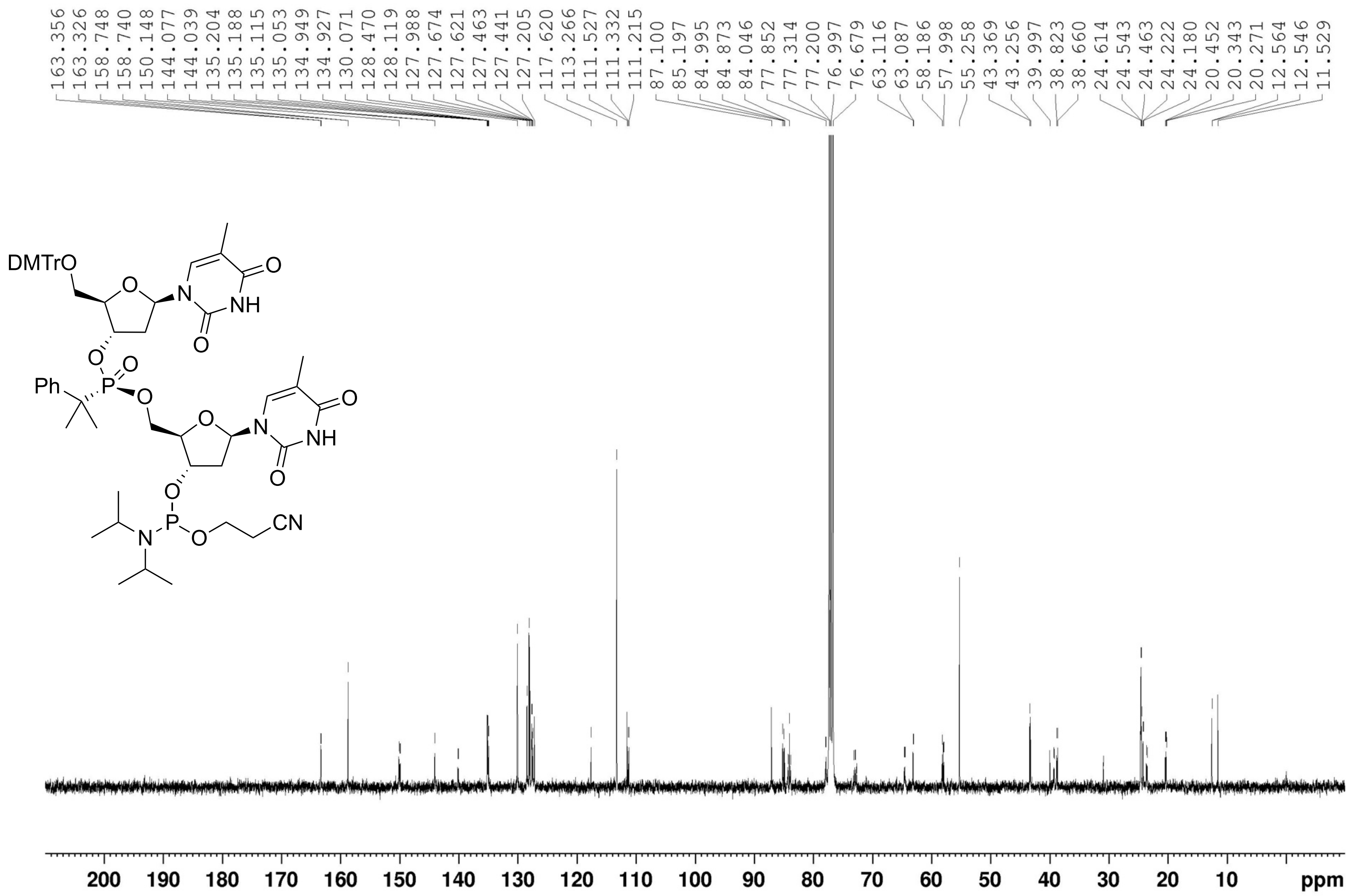


$^{13}C$ NMR (100.6 MHz, $CDCl_3$) spectrum of **($S_P$)-3aa-3**

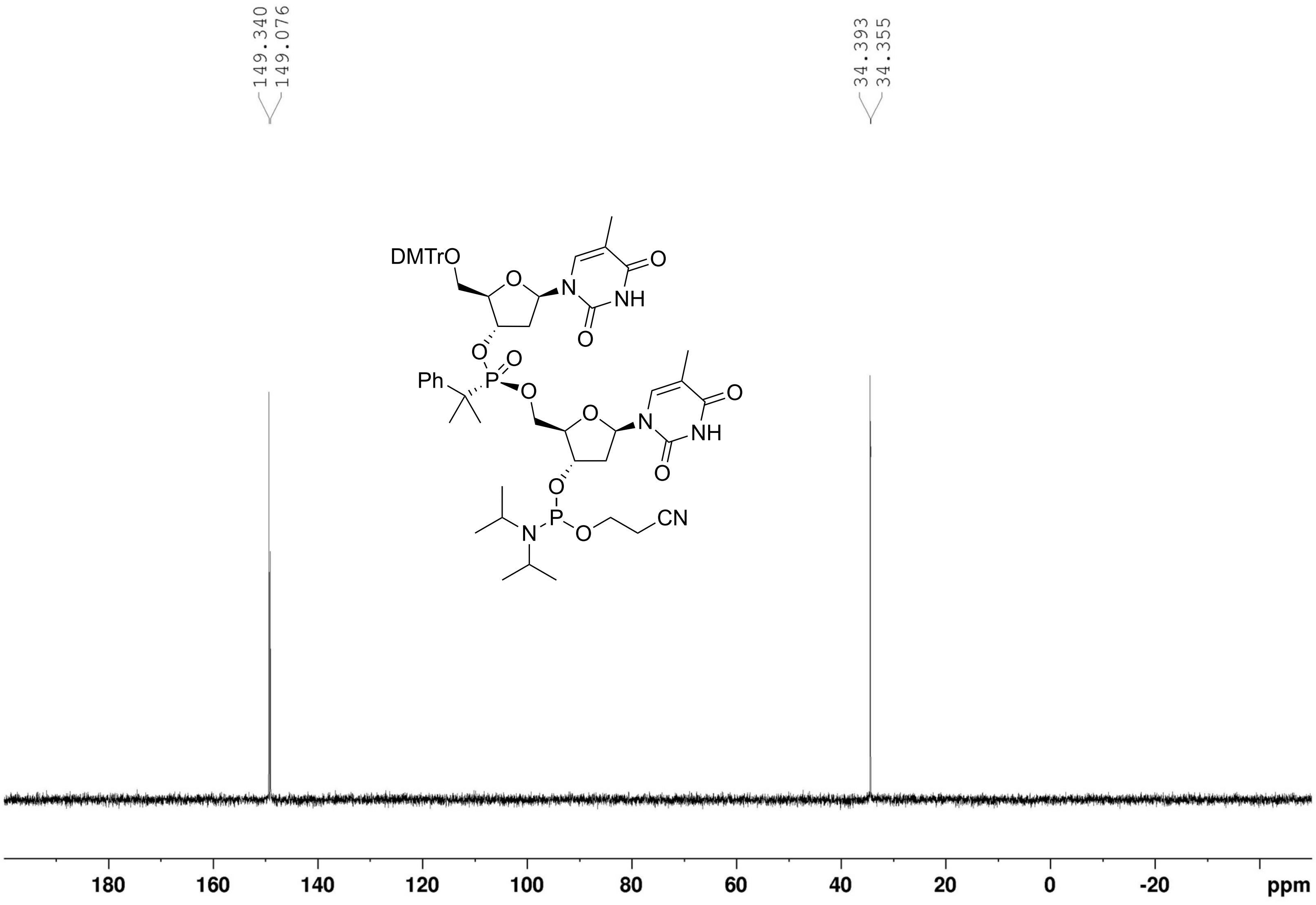


$^{31}P$ NMR (162 MHz, $CDCl_3$) spectrum of **($S_P$)-3aa-3**

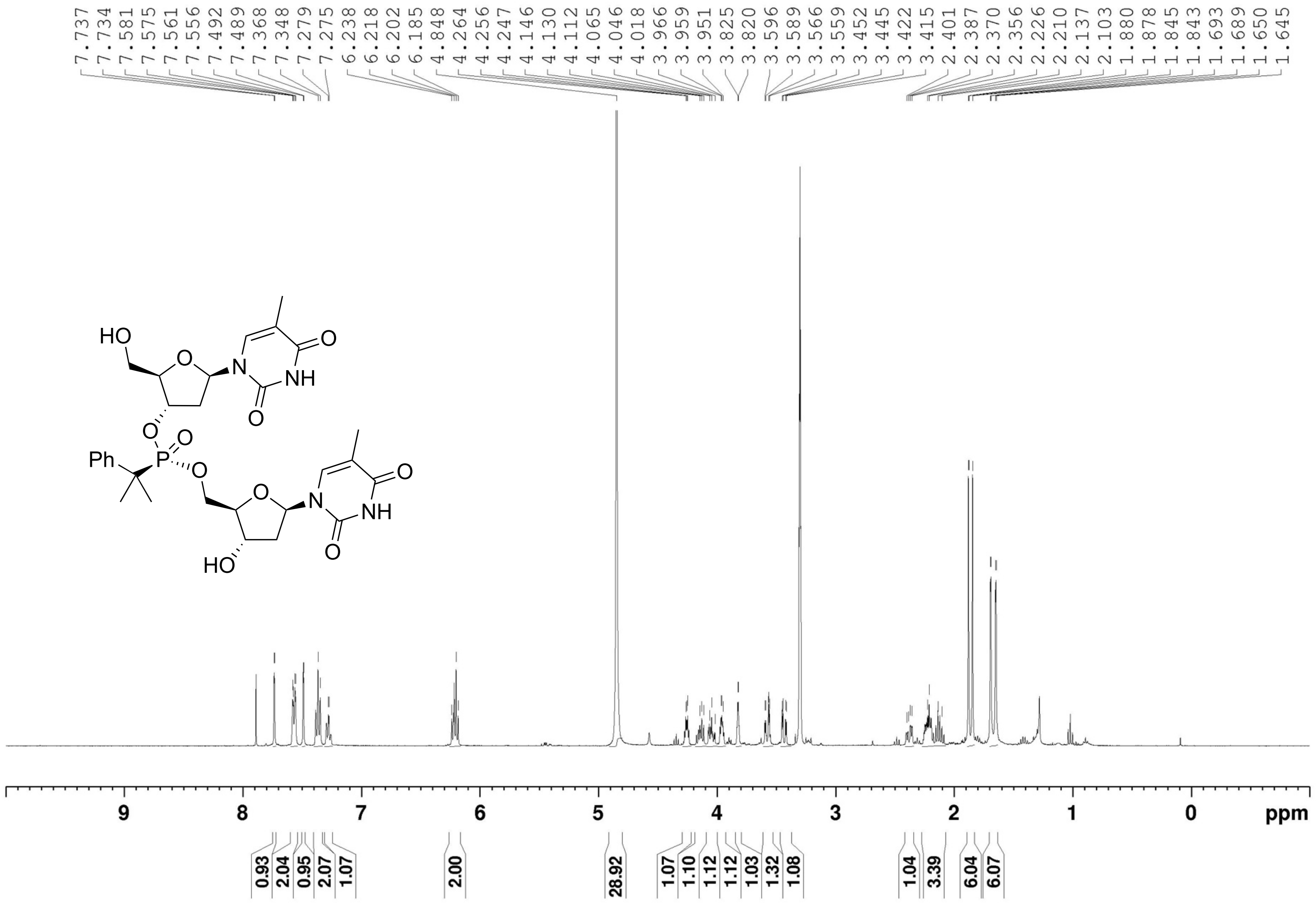


$^{1}$H NMR (400 MHz, $CD_3OD$) spectrum of **(*R*$_P$)-3aa-1**

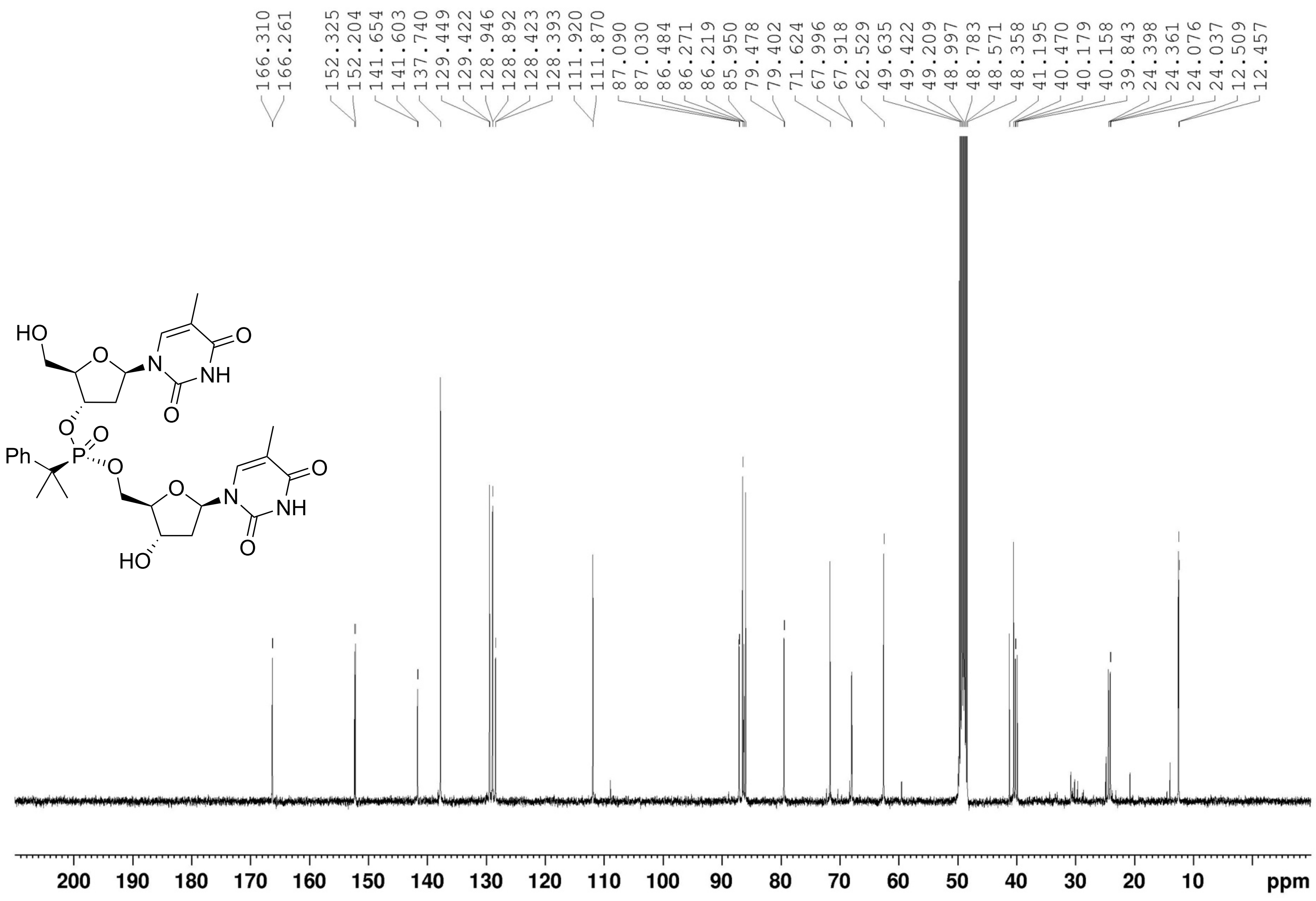


$^{13}$C NMR (100.6 MHz, CD$_3$OD) spectrum of **(*R*$_P$)-3aa-1**

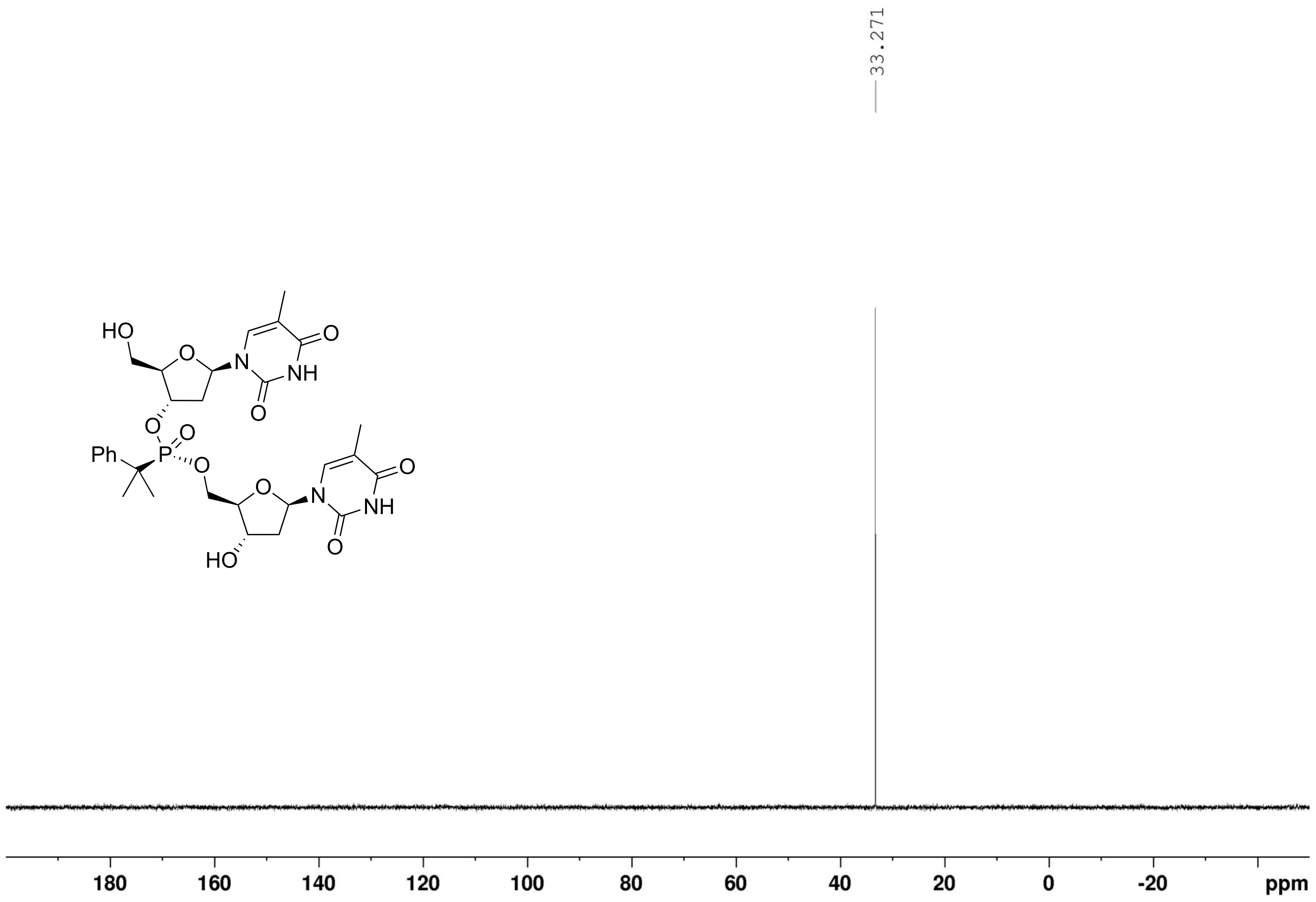


$^{31}$P NMR (162 MHz, $CD_3OD$) spectrum of **(*R*P)-3aa-1**

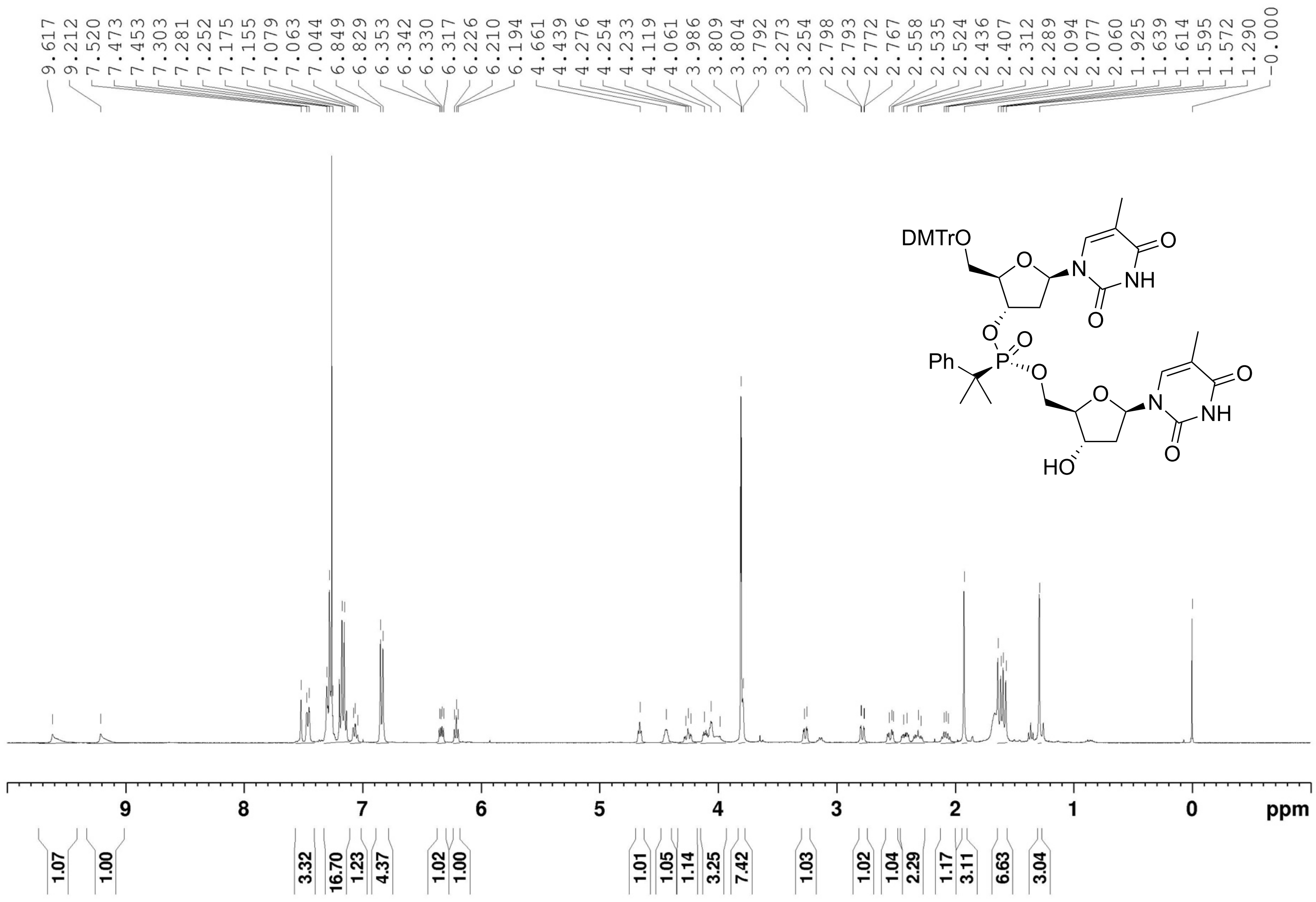

$^{1}$H NMR (400 MHz, CDCl$_3$) spectrum of **($R_P$)-3aa-2**

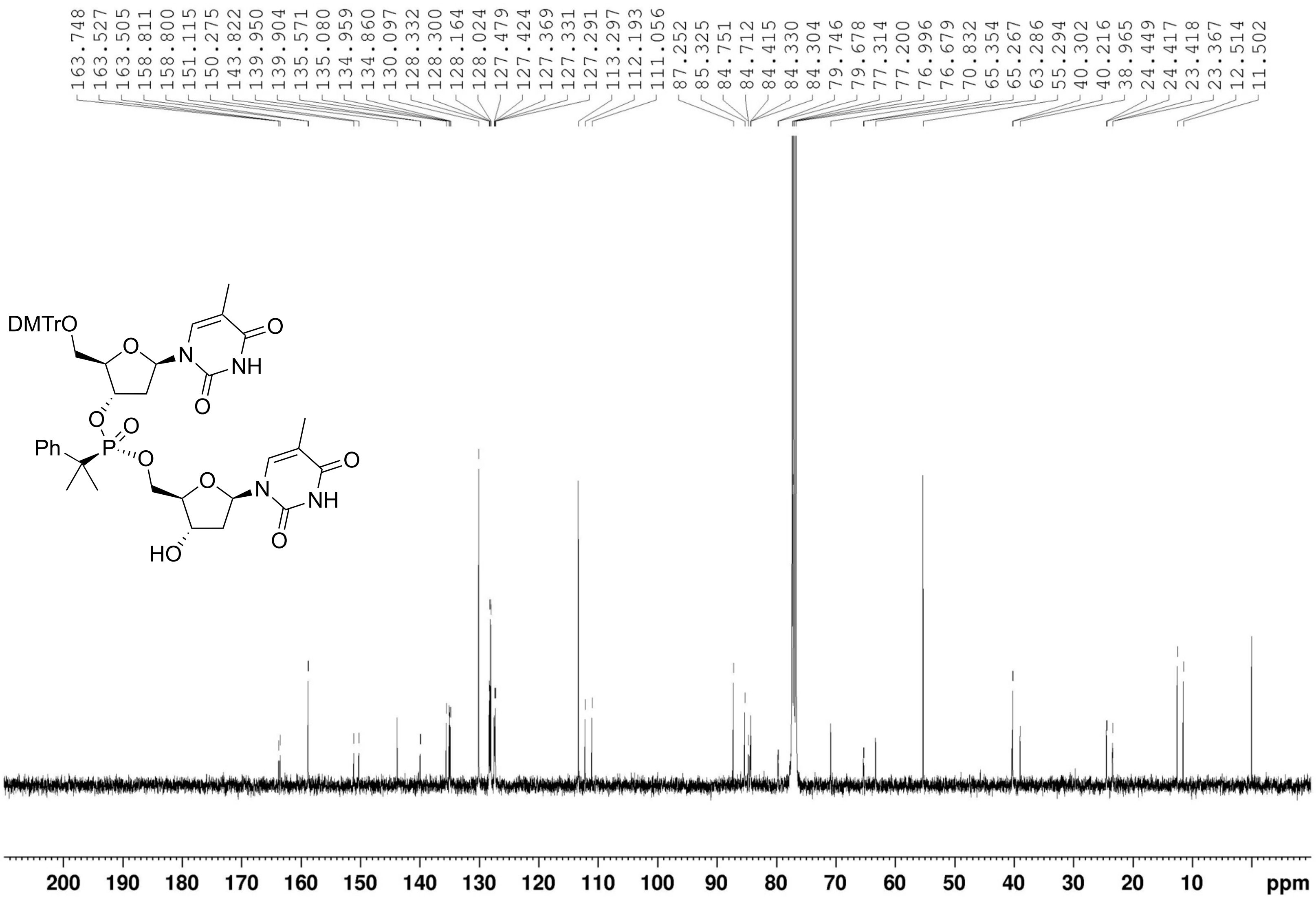


$^{13}$C NMR (100.6 MHz, $CDCl_3$) spectrum of **($R_P$)-3aa-2**

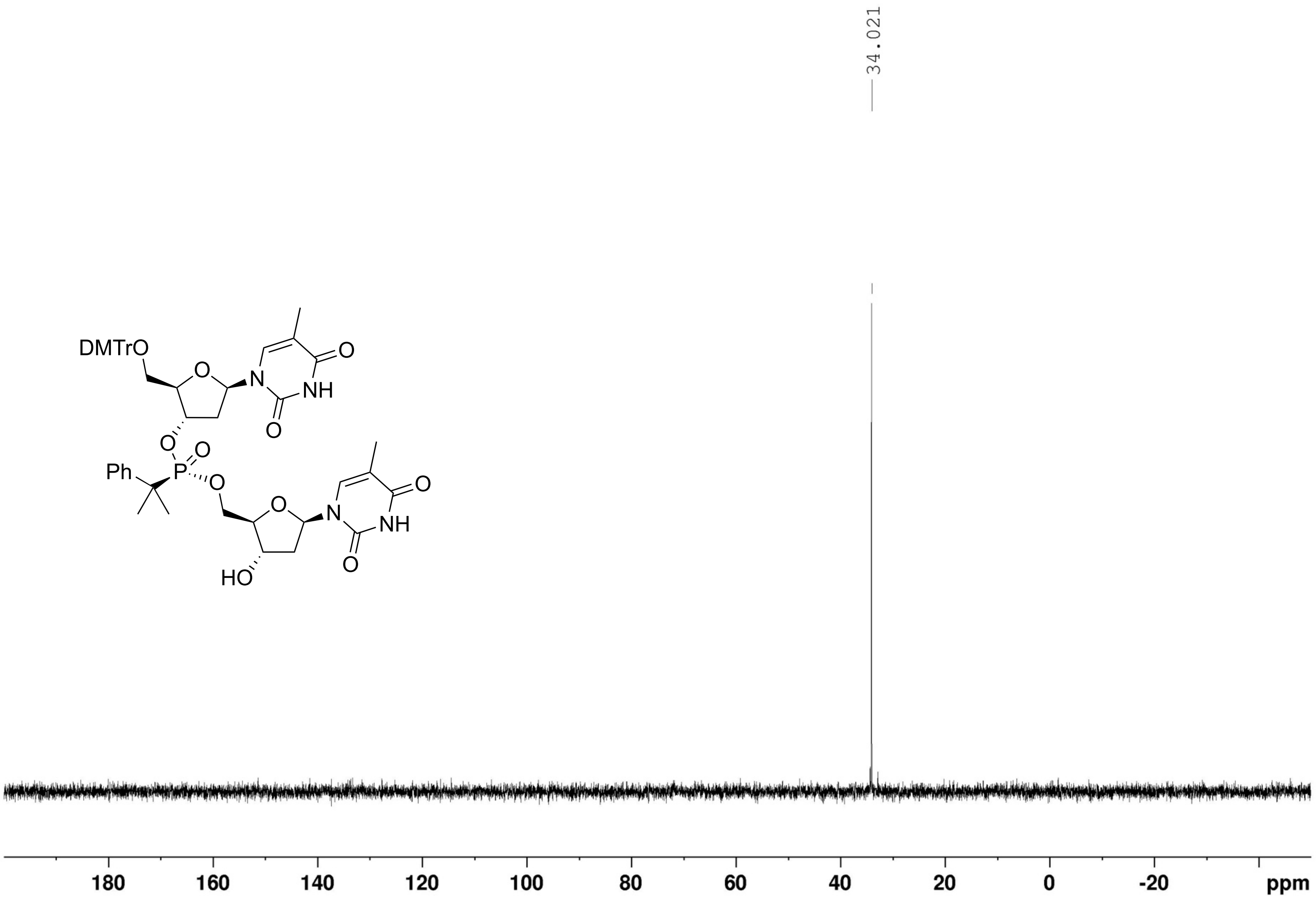


$^{31}P$ NMR (162 MHz, $CDCl_3$) spectrum of **(*R*P)-3aa-2**

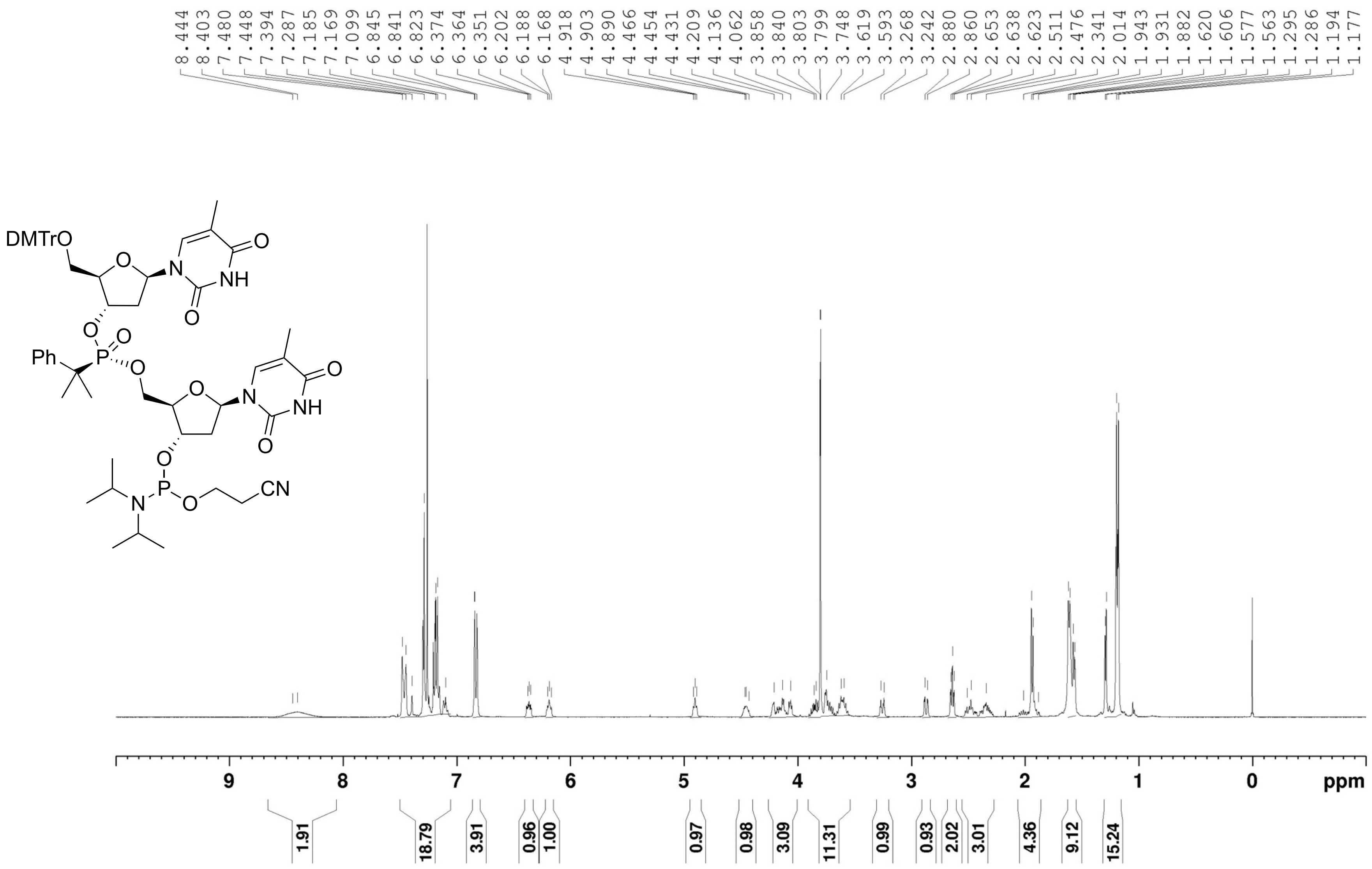

$^{1}$H NMR (400 MHz, $CDCl_3$) spectrum of **($R_P$)-3aa-3**

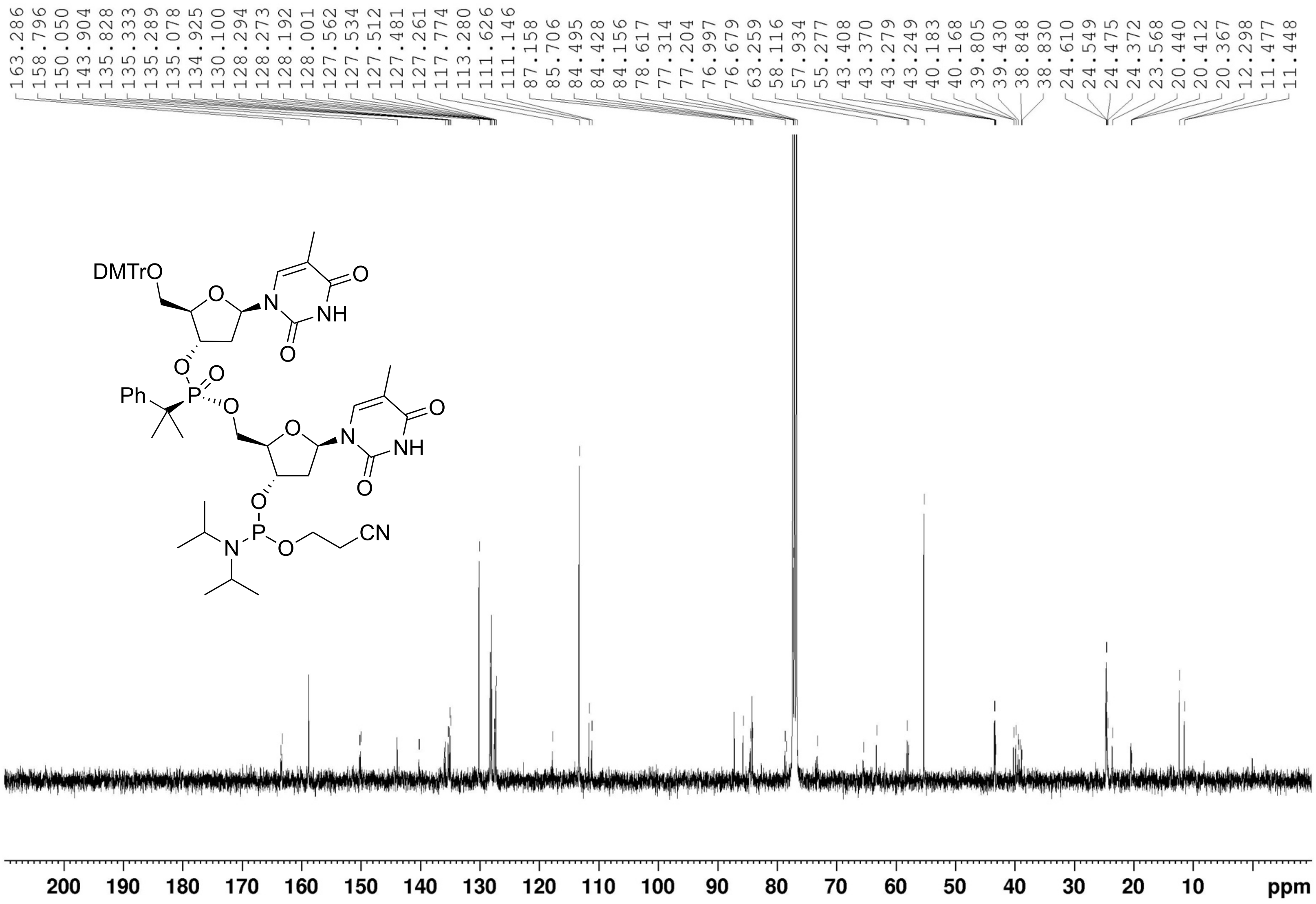


$^{13}$C NMR (100.6 MHz, $CDCl_3$) spectrum of **($R_P$)-3aa-3**

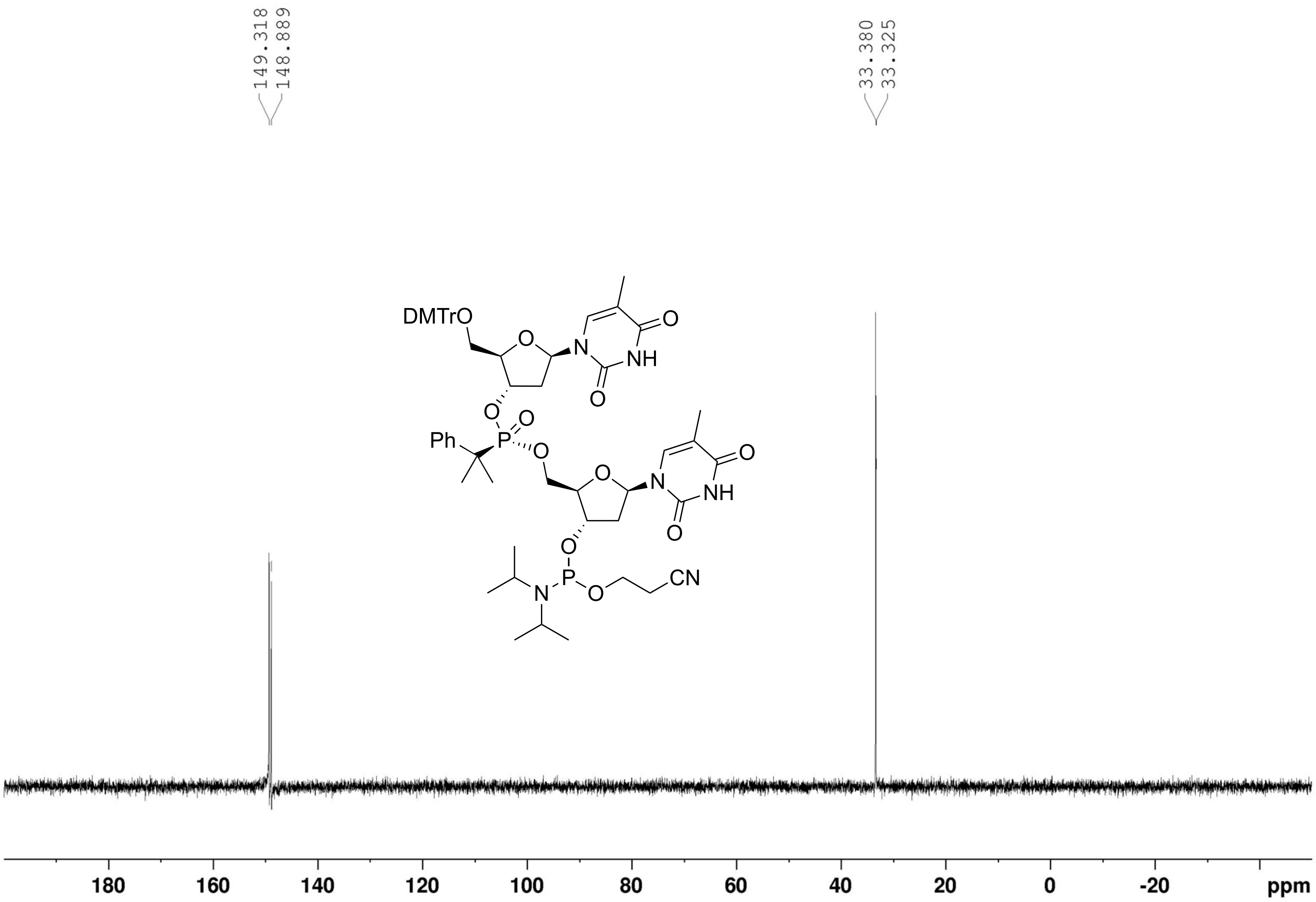


$^{31}$P NMR (162 MHz, $CDCl_3$) spectrum of **(*R*P)-3aa-3**

## ■ Supplementary References ■


(1) Dadashi-Silab, S., Pan, X. & Matyjaszewski, K. Phenyl Benzo[*b*]phenothiazine as a Visible Light Photoredox Catalyst for Metal-Free Atom Transfer Radical Polymerization. *Chem. Eur. J.* **23**, 5972–5977 (2017).

(2) Watanabe, M. et al. Spacer effects in metal-free organic dyes for visible-light-driven dye-sensitized photocatalytic hydrogen production. *J. Mater. Chem. A*, **2**, 12952–12961 (2014).

(3) Ding, X. et al. Highly efficient phenothiazine 5,5-dioxide-based hole transport materials for planar perovskite solar cells with a PCE exceeding 20%. *J. Mater. Chem. A*, **7**, 9510–9516 (2019).

(4) Münch, U. & Pfleiderer, W. Base-Labile Protecting Groups for the Oligoribonucleotide Synthesis. *Helv. Chim. Acta*, **84**, 1504–1517 (2001).

(5) Sekine, M. et al. Studies on Steric and Electronic Control of 2'−3' Phosphoryl Migration in 2'-Phosphorylated Uridine Derivatives and Its Application to the Synthesis of 2'-Phosphorylated Oligouridylates. *J. Org. Chem.* **61**, 4087–4100 (1996).

(6) Boers, R. B. et al. Synthesis and Spectroscopic Characterization of 1-$^{13}$C- and 4-$^{13}$C-Plastoquinone-9. *Eur. J. Org. Chem.* 2094–2108 (2002).

(7) Gao, L. et al. Lewis Acid-Catalyzed Selective Reductive Decarboxylative Pyridylation of *N*-Hydroxyphthalimide Esters: Synthesis of Congested Pyridine-Substituted Quaternary Carbons. *ACS Catal.* **9**, 10142–10151 (2019).

(8) Luo, J. & Zhang, J. Donor–Acceptor Fluorophores for Visible-Light-Promoted Organic Synthesis: Photoredox/Ni Dual Catalytic C(sp$^3$)–C(sp$^2$) Cross-Coupling. *ACS Catal.* **6**, 873–877 (2016).

(9) Stemper, J. et al. Development of Chiral Phosphoric Acids based on Ferrocene-Bridged Paracyclophane Frameworks. *Adv. Synth. Catal.* **355**, 3613–3624 (2013).

(10) Suzuki, M., Sekido, T., Matsuoka, S. & Takagi, K. Syntheses of Aliphatic Polycarbonates from 2′-Deoxyribonucleosides. *Biomacromolecules* **12**, 1449–1459 (2011).

(11) Sheldrick, G. M. SHELXT - integrated space-group and crystal-structure determination. *Acta Crystallogr.* **A71**, 3–8 (2015).

(12) Sheldrick, G. M. Crystal structure refinement with SHELXL. *Acta Crystallogr*. **C71**, 3–8 (2015).

(13) Dolomanov, O. V., Bourhis, L. J., Gildea, R. J., Howard, J. A. K. & Puschmann, H. Olex2: A complete structure solution, refinement and analysis program. *J. Appl. Crystallogr.* **42**, 339–341 (2009).

(14) Reich, D., Noble A., & Aggarwal V. K. Facile Conversion of α-Amino Acids into α-Amino Phosphonates by Decarboxylative Phosphorylation using Visible-Light Photocatalysis. *Angew. Chem.-Int. Ed.* **61**, e202207063 (2022).

(15) Qing, X., TingTing, Z. & Libiao, H. Preparation method of large-steric-hindrance alkyl substituted phosphite diester. Patent no. CN110922427A (2020).

(16) Meng, X. & Kim, S. Palladium(II)-Catalyzed Ortho-Arylation of Benzylic Phosphonic Monoesters Using Potassium Aryltrifluoroborates. *J. Org. Chem.* **78**, 11247–11254 (2013).

(17) Eagon, S. et al. Enantioselective reduction of α-substituted ketones mediated by the boronate ester TarB-$NO_2$. *Tetrahedron Letters.* **51**, 6418–6421 (2010).

(18) Hayashi, R., Shimizu, A. & Yoshida, J. The Stabilized Cation Pool Method: Metal- and Oxidant-Free Benzylic C–H/Aromatic C–H Cross-Coupling. *J. Am. Chem. Soc.* **138**, 8400–8403 (2016).
